# Design, development and applications of copper-catalyzed regioselective (4 + 2) annulations between diaryliodonium salts and alkynes

**DOI:** 10.1038/s42004-022-00768-3

**Published:** 2022-11-08

**Authors:** Weilin Wang, Junrui Zhou, Chao Wang, Congdi Zhang, Xiao-Qian Zhang, Youliang Wang

**Affiliations:** School of Chemistry, MOE Key Laboratory for Nonequilibrium Synthesis and Modulation of Condensed Matter, Xi’an Key Laboratory of Sustainable Energy Materials Chemistry, Xi’an Jiaotong University (XJTU), 710049 Xi’an, P. R. China

**Keywords:** Synthetic chemistry methodology, Natural product synthesis

## Abstract

Diaryliodonium salts have been extensively applied in organic synthesis as aryl cation equivalents. However, in the electrophilic reactions with alkenes or alkynes, only the electrophilic carbon of the diaryliodonium salts was involved while the other part of the aryl ring was not utilized. Herein, a reaction pattern of diaryliodonium was reported as oxa-1,4-dipoles to undergo (4 + 2) cycloaddition reactions with alkynes. Broad spectrum of the two reaction partners could be utilized in this protocol, enabling an operationally simple, high yielding, and regioselective synthetic approach to isocoumarins. Particularly, good to excellent regioselectivities were achieved for the sterically unbiased unsymmetrical diaryl acetylenes, which was challenging for other transition metal-catalyzed processes. The reaction could be scaled up with the ideal 1:1 stoichiometry and the isocoumarin type natural products Oospolactone and Thunberginol A could be obtained in one or three steps through this methodology.

## Abstract (web summary)

Intermolecular (4 + 2) cycloaddition with alkynes is a powerful approach to construct isocoumarin moieties, however, regioselectivity remains challenging for the transition metal-catalyzed approach. Here, the authors report copper-catalyzed regioselective (4 + 2) annulations between diaryliodonium salts and alkynes to synthesize isocoumarin-type natural products.

## Introduction

**Fig. 1:**
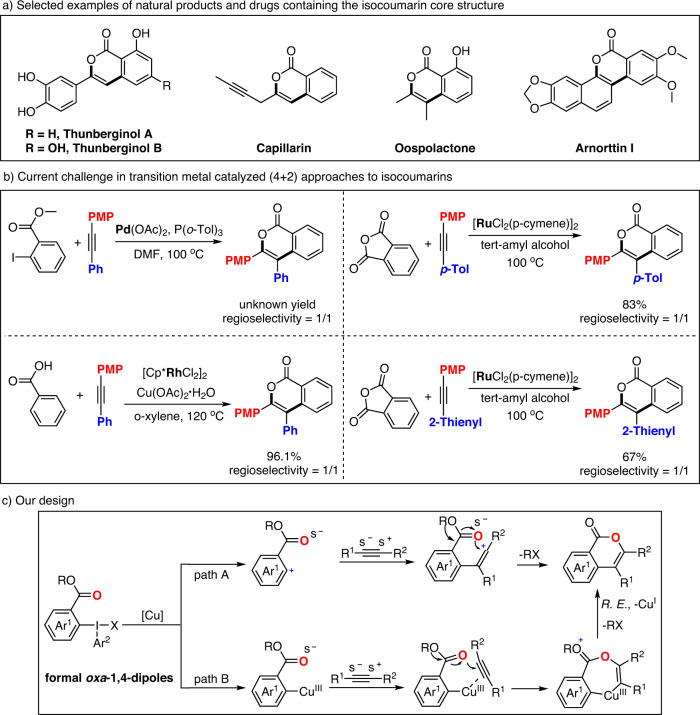
Formal oxa-1,4-dipoles based on diaryliodonium salts and the challenge in the synthesis of isocoumarins. **a** Selected examples of natural products and drugs containing the isocoumarin core structure. **b** Current challenge in transition metal catalyzed (4 + 2) approaches to isocoumarins. **c** Our design.

Isocoumarins are not only core structures of numerous biologically active natural products^1–5^ and clinically relevant molecules^6–9^ but also key intermediates in complex heterocycle synthesis^10,11^ (Fig. 1a). Great efforts from synthetic and medicinal chemists have been devoted to the construction of such structures^12^. Among them, the intermolecular (4 + 2) cycloaddition approaches with alkynes^13–22^ are undoubtedly desirable due to their convergency and flexibility. However, one significant problem for these reactions is the lack of regioselective control of sterically unbiased unsymmetrical diaryl acetylenes, which generally causing 1/1 regioselectivity (Fig. 1b)^20–22^. Herein, we attempted to employ *ortho*-ester substituted aryliodonium salts to establish a general and regioselective (4 + 2) cycloaddition reaction with alkynes to isocoumarins and, particularly, solve the regioselective challenge of sterically unbiased unsymmetrical diaryl acetylenes (Fig. 1c). It was envisioned that, under copper-catalysis conditions, the *ortho*-ester functionalized aryliodonium salt would first react with the alkyne substrate to generate a vinyl cation or its equivalent^23–34^, which then might be intercepted by the preinstalled ester entity to realize a formal 1,4-dipolar cycloaddition reaction. Thanks to the ionic nature of our proposed copper-catalyzed (4 + 2) annulations between diaryliodonium salts and alkynes, such regioselective challenge might be facilely solved via the site selective recognition of the electronically polarized acetylenic bond of the unsymmetrical diaryl acetylenes. Herein, we developed a highly efficient copper-catalyzed regioselective (4 + 2) annulations between diaryliodonium salts and alkynes to furnish a series isocoumarins with good to excellent regioselectivities. Furthermore, the isocoumarin type natural products Oospolactone and Thunberginol A could be obtained in one or three steps through this methodology.

## Results and discussion

### Reaction discovery and optimization of the reaction conditions

With the above design in mind, we directly probed the prospect of the proposal using the commercial 1-phenyl-1-hexyne (**1a**), mesityl(2-(methoxycarbonyl)phenyl)iodonium hexafluorophosphate (**2a**), Li_2_CO_3_ and 5 Å molecular sieves (Table 1). After mixing these components with 10 mol% Cu(CH_3_CN)_4_PF_6_ at 70 °C, the desired (4 + 2) cycloaddition products **3a** and **4a** could be identified by careful examination of the crude ^1^H NMR (Table 1, entry 1). While the yield and regioselectivity was poor, it indeed justified the viability of our original proposal in Fig. 1. Afterwards, the counterions for the copper(I) catalyst and the mesityl(2-(methoxycarbonyl)phenyl) iodonium salt 2 were routinely screened (entry 2–9), where the chloride proved optimal for the copper(I) salt and trifluoromethanesulfonate (OTf) for the iodonium salt **2** and a satisfactory moderate 73% yield with 11/1 regioselectivity was achieved. Efforts were then turned to investigate the influence of the ester entity of **2**. Compared to the methyl ester **2b**, the ethyl and isopropyl esters **2d** and **2e** also participated in the (4 + 2) reaction albeit not as efficient (entry 10-11). Surprisingly, adding ligands such as 2, 2´-bipyridine and bathocuproine completely terminated the reaction (see Supplementary Information, page S3). Various organic and inorganic bases were then examined and conditions without base afforded the best results (entry 12–14, see Supplementary Information, page S3).

**Table 1:** Conditions optimization^a^.


Entry	Catalyst	Additive	2	Yield^b^	3/4^c^
1	Cu(CH_3_CN)_4_PF_6_	Li_2_CO_3_	**2a**	25%	1.5/1
2	CuOTf$\cdot$PhH	Li_2_CO_3_	**2a**	48%	4/1
3	Cu(OTf)_2_	Li_2_CO_3_	**2a**	51%	1/1
4	CuTc	Li_2_CO_3_	**2a**	40%	3/1
5	CuI	Li_2_CO_3_	**2a**	45%	2/1
6	CuBr	Li_2_CO_3_	**2a**	55%	4/1
7	CuCl	Li_2_CO_3_	**2a**	57%	5/1
8	CuCl	Li_2_CO_3_	**2b**	73%	11/1
9	CuCl	Li_2_CO_3_	**2c**	23%	2/1
10	CuCl	Li_2_CO_3_	**2d**	64%	7/1
11	CuCl	Li_2_CO_3_	**2e**	65%	8/1
12	CuCl	DTBP	**2b**	41%	9/1
13	CuCl	K_2_CO_3_	**2b**	56%	10/1
**14**	**CuCl**		**2b**	**78% (84%)**^d^	**12/1**


^a^Conditions: **1a** (0.05 mmol), **2** (0.06 mmol), additive (0.06 mmol), catalyst (10 mol%), 5 Å MS (10 mg), anhydrous solvent (1 mL), under air, 70 °C.

^b^The yields of **3a** were determined by crude ^1^H NMR using dibutyl phthalate as internal standard.

^c^The ratios of **3a/4a** were determined by crude ^1^H NMR.

^d^Isolated yield for 0.2 mmol scale reaction.

The bolded values highlighted the best reaction conditions and the best reaction results.

### Substrate scope study

**Fig. 2:**
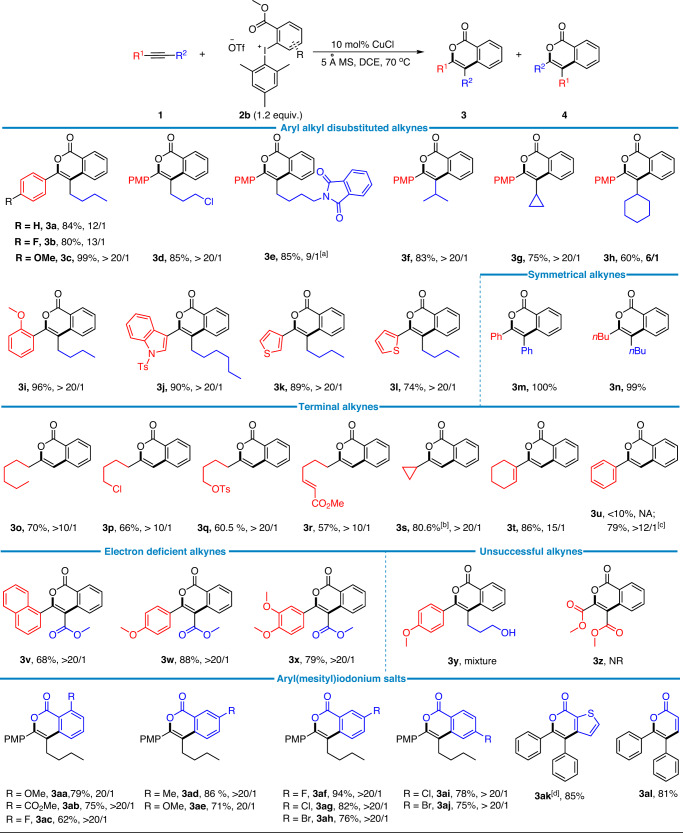
Scope of the alkynes and diaryliodonium salts. Reactions were performed on 0.2 mmol scale. Isolated yields were given. The ratio of **3** and **4** was determined by crude ^1^H NMR. ^[a]^The ratio of **3e** and **4e** was determined by ^1^H NMR of the purified product. ^[b]^1.5 equiv. of cyclopropylacetylene was used. ^[c]^1-Phenyl-2-(trimethylsilyl)acetylene was used. ^[d]^0.1 mmol scale.

With the optimal conditions in hands, the reaction scope for this regioselective (4 + 2) annulation of alkynes was systematically investigated (Fig. 2). In general, aryl/alkyl substituted unsymmetrical internal alkynes were suitable substrates with the aryl group being electronically either rich, neutral, or deficient. Notably, when the aryl group was *p*-methoxyphenyl (PMP), the corresponding isocoumarin product **3c** was generated in near quantitative yield (99%) and excellent regioselectivity (>20/1). For the alkyl chain of the aryl/alkyl substituted internal alkyne **1**, synthetically essential functional groups such as protected amine and chlorine could be tolerated and enabled good yields and regioselectivities (**3d**–**3e**). Besides the primary alkyl groups, sterically more demanding isopropyl, cyclopropyl, and cyclohexyl groups were not detrimental and all led to the corresponding isocoumarins (**3f**–**3h**) in moderate to good yields and moderate to excellent regioselectivities. Steric hinderance could also be tolerated on the aryl part of the aryl/alkyl alkyne (**3i**). Moreover, medicinally relevant and electron rich heterocycles such as the indole and thiophene units survived from the known copper-catalyzed arylations of arenes with diaryliodonium salts^35,36^ and led to comparably good results (**3g**–**3l**). Besides the aryl/alkyl substituted internal alkynes, the aryl/aryl and alkyl/alkyl ones were also suitable reaction partners (**3m**, **3n**). It turned out the substrate was not restricted to internal alkynes. Terminal alkynes worked as well in the (4 + 2) annulations. Again, various functional groups such as the protected hydroxyl group, chlorine, alkene, and cyclopropane groups could be tolerated (**3o**–**3t**). Interestingly, the phenylacetylene substrate merely afforded the isocoumarin product **3u** in low yield but the TMS protected phenylacetylene, 1-phenyl-2-(trimethylsilyl)acetylene, enabled an efficient synthesis of **3u** with good regioselectivity (>12/1). To further expand the substrate scope, another common type of alkynes, propiolates, were examined. While they have not been employed in the reported copper-catalyzed reactions of diaryliodonium salts, the expected (4 + 2) reactions could proceed smoothly, furnishing cycloadducts **3v**–**3x** in good results. Unfortunately, the more electron deficient alkyne, dimethyl acetylenedicarboxylate, and the substrate with a free hydroxyl group, 6-(4-methoxyphenyl)hex-5-yn-1-ol, were not as successful.

To further illustrate the generality of this regioselective (4 + 2) annulation reaction of alkynes, a series of readily synthesizable aryl(mesityl)iodonium salts were investigated (Fig. 2). It was demonstrated that various electron donating and withdrawing groups and halogens could be tolerated at the *ortho*-, *meta*-, and *para*-positions on the phenyl ring of the iodonium salt **2**, all leading to good yields and excellent regioselectivities (**3aa**–**3aj**). In addition, heterocycles such as thienyl and even the simple vinyl substituted iodonium salts were also suitable cycloaddition partners (**3ak** and **3al**).

**Fig. 3:**
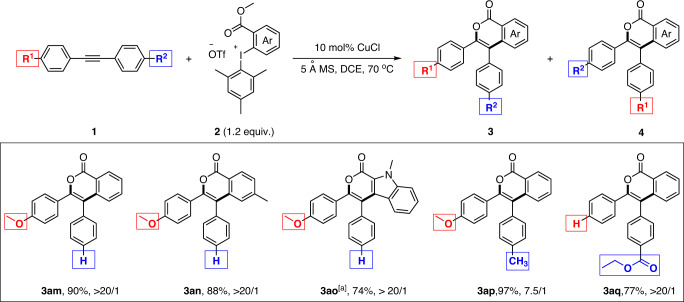
Representative sterically unbiased unsymmetrical diaryl acetylenes. Reactions were performed on 0.2 mmol scale. Isolated yields were given. The ratio of **3** and **4** was determined by crude ^1^H NMR. ^[a]^ Performed on 0.1 mmol scale.

Compared to other (4 + 2) annulation reactions of alkynes to isocoumarins^20–22^, one key feature of this copper-catalyzed reaction was the high-level of regioselective control of sterically unbiased unsymmetrical diaryl acetylenes (Fig. 3). For instance, when 1-(4-methoxyphenyl)−2-phenylacetylene was subjected to the standard copper catalysis conditions, perfect regioselectivities (>20/1) were achieved with several different iodonium salts. In sharp contrast, using the same substrate, the reported palladium−2and rhodium-^21^ catalyzed reactions resulted in no regioselectivity (1/1). It is worth highlighting that the steric environment around the two *sp* carbons of the acetylenic bond was essentially identical, which made it extremely challenging to control the regioselectivity by steric discrimination. On the other hand, its acetylenic bond was polarized by the *para*-methoxyl group and, thanks to the ionic nature of this copper-catalyzed reaction, the regioselective control was made possible by recognizing this inherent electronic difference. Indeed, when two competing electron donating groups (MeO and Me) were installed at the *para*-positions of the two phenyl rings of the parent diphenyl acetylene, i. e. 1-(4-methoxyphenyl)-2-(4-methylphenyl)acetylene, the acetylenic bond was less polarized compared to 1-(4-methoxyphenyl)-2-phenylacetylene and, consequently, the regioselectivity dropped to 7.5/1 under the same copper catalysis conditions (**3ap**). Again, the utilization of this sterically unbiased substrate in the reported ruthenium-catalyzed reaction^22^ only caused 1/1 mixture of two regio-isomers. Notably, besides the isocoumarin forming reactions, the regioselective control of sterically unbiased unsymmetrical diaryl acetylenes was currently a general challenge in transition metal-catalyzed cycloaddition reactions of alkynes^37–45^. Finally, an electron withdrawing group (-CO_2_Et) polarized diphenyl acetylene (ethyl 4-(phenylethynyl)benzoate) also showed excellent regioselectivity in this copper-catalyzed (4 + 2) reaction, albeit with less yield than the electron rich substrates.

### Synthetic applications

**Fig. 4:**
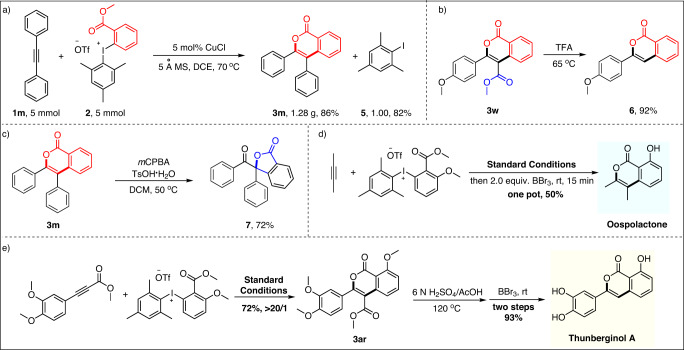
Synthetic applications. **a** The scale-up reaction. **b**, **c** Diverse transformations of the isocoumarin products. **d** Total synthesis of Oospolactone. **e** Total synthesis of Thunberginol A.

To highlight the practicability of this methodology, a scaled up reaction was performed with the ideal 1:1 stoichiometry for the two cycloaddition partners (Fig. 4a). Gratifyingly, the desired product **3m** could be isolated in 86% yield and 2, 4, 6-trimethyliodobenzene **5** could be recycled in 82% yield, which could be used to regenerate the starting diaryliodonium salt **2a**^46^. Beyond the scalability, the synthetic potential of this methodology could be further highlighted by the versatile downstream transformations of the (4 + 2) cycloadducts. For instance, the methyl ester entity (-CO_2_Me) of isocoumarin **3w** could be easily removed to get isocoumarin **6** (Fig. 4b). Moreover, isocoumarin **3m** could be isomerized into γ-lactone **7** efficiently via an oxidative rearrangement process^47^, which constituted the core structures of nature products such as Arnottin II^48^, Azaphilone and its derivatives^49^ (Fig. 4c).Thanks to the above copper-catalyzed regioselective (4 + 2) annulation reaction, the isocoumarin type natural product Oospolactone could be prepared in one step and 50% yield from 2-butyne (Fig. 4d). Furthermore, under the same copper catalysis conditions, methyl 3-(3,4-dimethoxyphenyl)propiolate could be facilely converted into isocoumarin **3ar** in 72% yield, which could be further elaborated into Thunberginol A in two successive steps in 93% yield (Fig. 4e), highlighting the power of this new methodology for streamline and diverse synthesis of molecules for function.

## Conclusion

In summary, a novel and practical copper-catalyzed regioselective (4 + 2) annulation reaction between diaryliodonium salts and alkynes to isocoumarins was developed with a broad spectrum of substrate scope. Particularly, good to excellent regioselectivities were achieved for the sterically unbiased unsymmetrical diaryl acetylenes, which was challenging for other transition metal-catalyzed processes. The reaction could be scaled up with the ideal 1:1 stoichiometry and the (4 + 2) cycloadducts could be facilely transformed into elaborated structures and natural products. The synthesis of other bioactive and complex natural products based on this methodology is underway in our laboratory.

## Methods

Alkyne **1** (1.00 equiv., 0.2 mmol), aryl(mesityl)iodonium trifluoromethanesulfonate **2** (1.20 equiv., 0.24 mmol), copper(I) chloride (0.10 equiv., 0.02 mmol, 3.8 mg) and 5 Å MS (20 mg) were added to a dried vial sequentially. The reaction mixture was stirred at 70 °C in anhydrous DCE (0.05 M) until complete consumption of the alkyne was observed by TLC. Then it was concentrated and purified on silica gel to get the target product **3** (*Z*/*E* selectivity was determined by crude NMR.) Full experimental details and compound characterizations are given in the Supplementary Information and Supplementary Data 1.

## Supplementary information

Supplementary Information

Description of Additional Supplementary Files

Supplementary Data 1

## Data Availability

The data supporting the findings of this study are available within this article and its Supplementary Information and Supplementary Data 1.
