# Supplementary material for: Design, development and applications of copper-catalyzed regioselective (4 + 2) annulations between diaryliodonium salts and alkynes
Source: Commun Chem. 2022 Nov 8;5:145. doi: 10.1038/s42004-022-00768-3 (PMC9814649; doi:10.1038/s42004-022-00768-3)
Supplement: Supplementary file 1 — Supplementary Information [file 42004_2022_768_MOESM1_ESM.pdf]

# *Supplementary Information*

## Design, development and applications of copper-catalyzed regioselective (4+2) annulations between diaryliodonium salts and alkynes

Weilin Wang,* Junrui Zhou, Chao Wang, Congdi Zhang, Xiao-Qian Zhang, Youliang Wang*

[a] School of Chemistry, MOE Key Laboratory for Nonequilibrium Synthesis and Modulation of Condensed Matter, Xi'an Key Laboratory of Sustainable Energy Materials Chemistry, Xi'an Jiaotong University (XJTU), Xi'an 710049, P. R. China

*Corresponding Author, email: weilinwang@xjtu.edu.cn; youliangwang@xjtu.edu.cn


| Supplementary Methods | Page number |
|---|---|
| **General** | S2 |
| **Reaction discovery and optimizations** | S3 |
| **Preparation of the unknown diaryliodonium trifluoromethanesulfonate** | S4 |
| **Scope of the alkynes** | S17 |
| **Scope of the aryl(mesityl)iodonium salts** | S41 |
| **Synthetic applications** | S59 |
| **References** | S65 |

# Supplementary Methods

## General

NMR spectra were recorded on a Bruker-400 MHz . $^1H$ NMR spectra were recorded at 400 MHz and data are reported as follows: chemical shift in ppm using residue solvent peak as internal standard ($CDCl_3$ δ 7.26 ppm), multiplicity (s = singlet, d = doublet, t = triplet, q = quartet, m = multiplet or overlap of nonequivalent resonances), integration. $^{13}C$ NMR spectra were recorded at 101 MHz and data are reported as follows: chemical shift in ppm using solvent residue peak as internal indicator ($CDCl_3$ δ 77.15 ppm). $^{19}F$ NMR spectra were recorded at 376 MHz. High resolution mass spectra were performed on a WATERS I-Class VION IMS QTof at the Instrumental Analysis Center of Xi'an Jiaotong University and are given in m/z. GCMS were performed on Agilent 8860 GC/5977B GC/MSD System and are given in m/z. All reactions were carried out in glassware dried overnight in an oven at 110 °C. All reactions were performed under air. Commercial reagents and solvents were used without further purification unless stated otherwise. TLC was performed on pre-coated glass plates visualized either with a UV lamp (254 nm), or using solutions of $KMnO_4$–$K_2CO_3$ in water followed by heating. Flash chromatography was performed on silica gel (230-400 mesh).

# Reaction discovery and optimizations

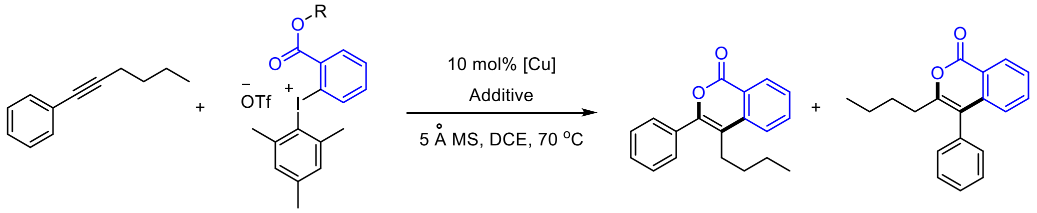


1a 2 3a 4a

| Entry | Catalyst | Additive | Solvent | 2 | Yield [b] | 3/4 [c] |
|---|---|---|---|---|---|---|
| 1 | $Cu(CH_3CN)_4PF_6$ | $Li_2CO_3$ | DCE | **2a** | 25% | 1.5/1 |
| 2 | CuOTf·PhH | $Li_2CO_3$ | DCE | **2a** | 48% | 4/1 |
| 3 | $Cu(OTf)_2$ | $Li_2CO_3$ | DCE | **2a** | 51% | 1/1 |
| 4 | CuTc | $Li_2CO_3$ | DCE | **2a** | 40% | 3/1 |
| 5 | CuI | $Li_2CO_3$ | DCE | **2a** | 45% | 2/1 |
| 6 | CuBr | $Li_2CO_3$ | DCE | **2a** | 55% | 4/1 |
| 7 | CuCl | $Li_2CO_3$ | DCE | **2a** | 57% | 5/1 |
| 8 | CuCl | $Li_2CO_3$ | DCE | **2b** | 73% | 11/1 |
| 9 | CuCl | $Li_2CO_3$ | DCE | **2c** | 23% | 2/1 |
| 10 | CuCl | $Li_2CO_3$ | DCE | **2d** | 64% | 7/1 |
| 11 | CuCl | $Li_2CO_3$ | DCE | **2e** | 65% | 8/1 |
| 12 | CuCl | $Li_2CO_3$ | $Ph\text{-}CF_3$ | **2b** | 59% | 5/1 |
| 13 | CuCl | $Li_2CO_3$ | Dioxane | **2b** | 0 | 0 |
| 14 | CuCl | $Li_2CO_3$ | $THF/CH_3CN$ | **2b** | 0 | 0 |
| 15[d] | CuCl | $Li_2CO_3$ | DCE | **2b** | 0 | 0 |
| 16 | CuCl | DTBP | DCE | **2b** | 41% | 9/1 |
| 17 | CuCl | $K_2CO_3$ | DCE | **2b** | 56% | 10/1 |
| 18 | CuCl | $Cs_2CO_3$ | DCE | **2b** | 0 | 0 |
| 19 | CuCl | 2,6-Dicloropyridine | DCE | **2b** | 56% | 10/1 |
| 20 | CuCl | 2,6-Dimethylpyridine | DCE | **2b** | 0 | 0 |
| **21** | **CuCl** | | **DCE** | **2b** | **78% (84%)[e]** | **12/1** |

[a] Conditions: **1a** (0.05 mmol), **2** (0.06 mmol), addtive (0.06 mmol), catalyst (10 mol%), 5 Å MS (10 mg), anhydrous solvent (1 mL), under air, 70 °C. [b] The yields of **3a** were determined by crude $^1H$ NMR using dibutyl phthalate as internal standard. [c] The ratios of **3a**/**4a** were determined by crude $^1H$ NMR. [d] the ligand Bipyridine and Bathocuproine were added respectively. [e] Isolated yield for 0.2 mmol scale reaction.

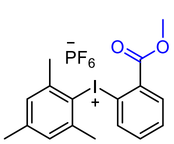


2a

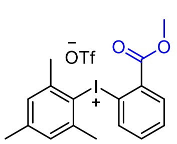


2b

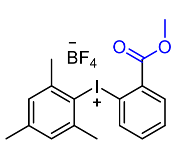


2c

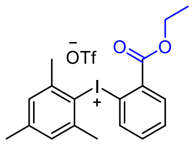


2d

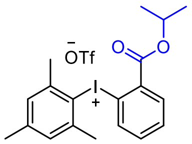


2e

# Preparation of the unknown diaryliodonium trifluoromethanesulfonate

## Mesityl(3-methoxy-2-(methoxycarbonyl)phenyl)iodonium trifluoromethanesulfonate (2aa)

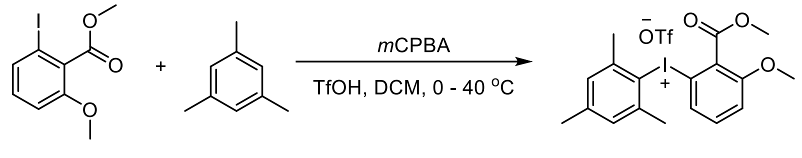


6-Methoxy-2-iodobenzoic acid methyl ester (730 mg, 2.5 mmol,), *m*CPBA (665 mg, 84% active oxidant, 3.2 mmol) and mesitylene (0.7 mL, 5 mmol) were dissolved in DCM (25 mL). The mixture was cooled to 0 °C, and TfOH (442 uL, 5 mmol) was added dropwise. The reaction was stirred for another 10 minutes at 0 °C, then warmed to 40 °C and stirred until 6-methoxy-2-iodobenzoic acid methyl ester disappeared. Subsequently, the phases were separated and the aqueous phase was extracted with DCM (3 x 15 mL). The combined organic phases were dried ($MgSO_4$) and concentrated under reduced pressure. The target compound was isolated by repeated recrystallization from diethylether as a white solid (882 mg, 63%).

$R_f$ 0.38 (EtOAc/EtOH = 20/1).

**$^1$H NMR** (400 MHz, Chloroform-*d*) δ 7.43 (t, *J* = 8.4 Hz, 1H), 7.23 (d, *J* = 8.4 Hz, 1H), 7.17 (s, 2H), 6.45 (d, *J* = 8.0 Hz, 1H), 4.10 (s, 3H), 3.98 (s, 3H), 2.54 (s, 6H), 2.41 (s, 3H).

**$^{13}$C NMR** (101 MHz, $CDCl_3$) *δ* 169.0, 163.4, 144.8, 143.5, 137.1, 130.3, 120.4, 120.0, 118.4, 116.7, 115.3, 57.1, 54.7, 26.9, 21.5.

**$^{19}$F NMR** (376 MHz, $CDCl_3$) *δ* -78.2 (s, 3F).

**HRMS** (ESI) m/z: $[M - OTf]^+$ Calcd for $C_{18}H_{20}IO_3^+$ 411.0452; Found 411.0448.

**(2,3-Bis(methoxycarbonyl)phenyl)(mesityl)iodonium trifluoromethanesulfonate (2ab)**

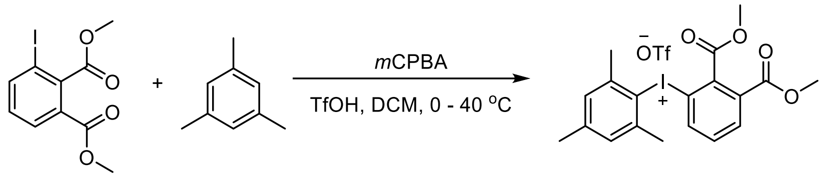


3-Iodo-dimethylphthalate (800 mg, 2.5 mmol), *m*CPBA (665 mg, 84% active oxidant, 3.2 mmol) and mesitylene (0.7 mL, 5 mmol) were dissolved in DCM (25 mL). The mixture was cooled to 0 °C, and TfOH (442 uL, 5 mmol) was added dropwise. The reaction was stirred for another 10 minutes at 0 °C, then warmed to 40 °C and stirred until 3-Iodo-dimethylphthalate disappeared. Subsequently, the phases were separated and the aqueous phase was extracted with DCM (3 x 15 mL). The combined organic phases were dried ($MgSO_4$) and concentrated under reduced pressure. The target compound was isolated by repeated recrystallization from diethylether as a white solid (745 mg, 50.7%).

$R_f$ 0.40 (EtOAc/EtOH = 20/1).

**$^1$H NMR** (400 MHz, Chloroform-*d*) δ 7.71 (d, *J* = 9.2 Hz, 1H), 7.57 (t, *J* = 8.0 Hz, 1H), 7.20 (s, 2H), 6.96 (d, *J* = 8.0 Hz, 1H), 4.10 (s, 3H), 3.96 (s, 3H), 2.55 (s, 6H), 2.42 (s, 3H).

**$^{13}$C NMR** (101 MHz, $CDCl_3$) *δ* 167.1, 167.0, 145.7, 143.8, 138.6, 136.2, 131.0, 130.7, 130.4, 126.5, 118.5, 113.7, 55.4, 53.6, 26.9, 21.5.

**$^{19}$F NMR** (376 MHz, $CDCl_3$) *δ* -78.4 (s, 3F).

**HRMS** (ESI) m/z: $[M - OTf]^+$ Calcd for $C_{19}H_{22}IO_4^+$ 439.0401; Found 439.0403.

**(3-Fluoro-2-(methoxycarbonyl)phenyl)(mesityl)iodonium trifluoromethanesulfonate (2ac)**

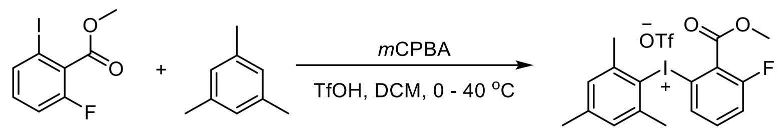


Methyl 2-fluoro-6-iodobenzoate (700 mg, 2.5 mmol,), *m*CPBA (665 mg, 84% active oxidant, 3.2 mmol) and mesitylene (0.7 mL, 5 mmol) were dissolved in DCM (25 mL). The mixture was cooled to 0 °C, and TfOH (442 uL, 5 mmol) was added dropwise. The reaction was stirred for another 10 minutes at 0 °C, then warmed to 40 °C and stirred until methyl 2-fluoro-6-iodobenzoate disappeared. Subsequently, the phases were separated and the aqueous phase was extracted with DCM (3 x 15 mL). The combined organic phases were dried ($MgSO_4$) and concentrated under reduced pressure. The target compound was isolated by repeated recrystallization from diethylether as a white solid (836 mg, 61%).

$R_f$ 0.38 (EtOAc/EtOH = 20/1).

**$^1$H NMR** (400 MHz, Chloroform-*d*) δ 7.57 – 7.52 (m, 1H), 7.42 (q, *J* = 8.4 Hz, 1H), 7.19 (s, 2H), 6.68 (d, *J* = 8.0 Hz, 1H), 4.14 (s, 3H), 2.54 (s, 7H), 2.42 (s, 3H).

**$^{13}$C NMR** (101 MHz, $CDCl_3$) *δ* 167.0, 164.5 (d, *J* = 270.3 Hz), 145.6, 143.9, 138.0 (d, *J* = 9.6 Hz), 130.6, 124.6 (d, *J* = 3.5 Hz), 120.9, 120.7 ,118.3, 114.9, 55.2, 26.8, 21.5.

**$^{19}$F NMR** (376 MHz, $CDCl_3$) *δ* -78.4 (s, 3F), -96.7 (q, J = 4.5 Hz, 1F).

**HRMS** (ESI) m/z: $[M - OTf]^+$ Calcd for $C_{17}H_{17}FIO_2^+$ 399.0252; Found 399.0258.

**Mesityl (2-(methoxycarbonyl)-4-methylphenyl) iodonium trifluoromethanesulfonate (2ad)**

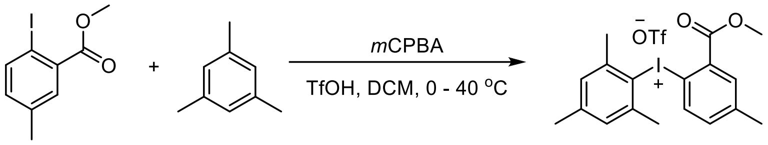


5-Methyl-2-iodobenzoic acid methyl ester (690 mg, 2.5 mmol,), *m*CPBA (665 mg, 84% active oxidant, 3.2 mmol) and mesitylene (0.7 mL, 5 mmol) were dissolved in DCM (25 mL). The mixture was cooled to 0 °C, and TfOH (442 uL, 5 mmol) was added dropwise. The reaction was stirred for another 10 minutes at 0 °C, then warmed to 40 °C and stirred until 5-methyl-2-iodobenzoic acid methyl ester disappeared. Subsequently, the phases were separated and the aqueous phase was extracted with DCM (3 x 15 mL). The combined organic phases were dried ($MgSO_4$) and concentrated under reduced pressure. The target compound was isolated by repeated recrystallization from diethylether as a white solid (1105 mg, 81.3%).

$R_f$ 0.38 (EtOAc/EtOH = 20/1).

**$^1$H NMR** (400 MHz, Chloroform-*d*) δ 8.14 (d, *J* = 2.0 Hz, 1H), 7.37 (dd, *J* = 8.4, 2.0 Hz, 1H), 7.18 (s, 2H), 6.67 (d, *J* = 8.4 Hz, 1H), 4.10 (s, 3H), 2.53 (s, 7H), 2.44 (s, 3H), 2.41 (s, 3H).

**$^{13}$C NMR** (101 MHz, $CDCl_3$) *δ* 167.9, 145.2, 143.8, 142.5, 138.0, 134.2, 130.4, 128.1, 127.1, 117.1, 108.6, 54.7, 26.8, 21.4, 20.8.

**$^{19}$F NMR** (376 MHz, $CDCl_3$) *δ* -78.2 (s, 3F)

**HRMS** (ESI) m/z: $[M - OTf]^+$ Calcd for $C_{18}H_{20}IO_2^+$ 395.0503; Found 395.0502.

**Mesityl(4-methoxy-2-(methoxycarbonyl)phenyl)iodonium trifluoromethanesulfonate (2ae)**

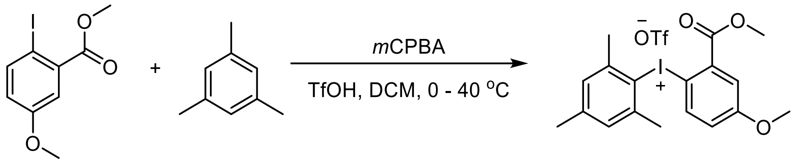


5-Methoxy-2-iodobenzoic acid methyl ester (730 mg, 2.5 mmol,), *m*CPBA (665 mg, 84% active oxidant, 3.2 mmol) and mesitylene (0.7 mL, 5 mmol) were dissolved in DCM (25 mL). The mixture was cooled to 0 °C, and TfOH (442 uL, 5 mmol) was added dropwise. The reaction was stirred for another 10 minutes at 0 °C, then warmed to 40 °C and stirred until 5-methoxy-2-iodobenzoic acid methyl ester disappeared. Subsequently, the phases were separated and the aqueous phase was extracted with DCM (3 x 15 mL). The combined organic phases were dried ($MgSO_4$) and concentrated under reduced pressure. The target compound was isolated by repeated recrystallization from diethylether as a white solid (924 mg, 66%).

$R_f$ 0.38 (EtOAc/EtOH = 20/1).

**$^1$H NMR** (400 MHz, Chloroform-*d*) δ 7.81 (d, *J* = 2.8 Hz, 1H), 7.17 (s, 2H), 7.08 (dd, *J* = 9.2, 2.8 Hz, 1H), 6.66 (d, *J* = 9.2 Hz, 1H), 4.11 (s, 3H), 3.87 (s, 3H), 2.55 (s, 6H), 2.41 (s, 3H).

**$^{13}$C NMR** (101 MHz, $CDCl_3$) *δ* 167.6, 162.1, 145.1, 143.7, 130.4, 129.4, 128.5, 123.1, 118.6, 117.7, 101.4, 56.3, 54.8, 26.9, 21.5.

**$^{19}$F NMR** (376 MHz, $CDCl_3$) *δ* -78.2 (s, 3F).

**HRMS** (ESI) m/z: [M - OTf]$^+$ Calcd for $C_{18}H_{20}IO_3^+$ 411.0452; Found 411.0447.

**(4-Fluoro-2-(methoxycarbonyl)phenyl)(mesityl)iodonium trifluoromethanesulfonate (2af)**

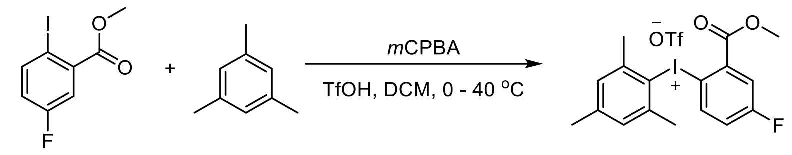


5-Fluoro-2-iodobenzoic acid methyl ester (700 mg, 2.5 mmol,), *m*CPBA (665 mg, 84% active oxidant, 3.2 mmol) and mesitylene (0.7 mL, 5 mmol) were dissolved in DCM (25 mL). The mixture was cooled to 0 °C, and TfOH (442 uL, 5 mmol) was added dropwise. The reaction was stirred for another 10 minutes at 0 °C, then warmed to 40 °C and stirred until 5-fluoro-2-iodobenzoic acid methyl ester disappeared. Subsequently, the phases were separated and the aqueous phase was extracted with DCM (3 x 15 mL). The combined organic phases were dried ($MgSO_4$) and concentrated under reduced pressure. The target compound was isolated by repeated recrystallization from diethylether as a white solid (973 mg, 71%).

$R_f$ 0.38 (EtOAc/EtOH = 20/1).

**$^1$H NMR** (400 MHz, Chloroform-*d*) δ 8.05 – 8.02 (m, 1H), 7.33 – 7.28 (m, 1H), 7.20 (s, 2H), 6.81 – 6.78 (m, 1H), 4.13 (s, 3H), 2.55 (s, 6H), 2.42 (s, 3H).

**$^{13}$C NMR** (101 MHz, $CDCl_3$) $\delta$ 166.8 (d, $J$ = 2.7 Hz), 164.3 (d, $J$ = 254.5 Hz), 145.5, 143.9, 130.6, 130.3 (d, $J$ = 7.7 Hz), 129.6 (d, $J$ = 7.8 Hz), 124.3, 120.8 (d, $J$ = 24.8 Hz), 117.5,106.0 (d, $J$ = 3.0 Hz), 55.1, 26.9, 21.0.

**$^{19}$F NMR** (376 MHz, $CDCl_3$) $\delta$ -78.4 (s, 3F), 107.5 – 107.6 (m, 1F)

**HRMS** (ESI) m/z: [M - OTf]$^+$ Calcd for $C_{17}H_{17}FIO_2^+$ 399.0252; Found 399.0258.

**(5-Chloro-2-(methoxycarbonyl)phenyl)(mesityl)iodonium trifluoromethanesulfonate (2ag)**

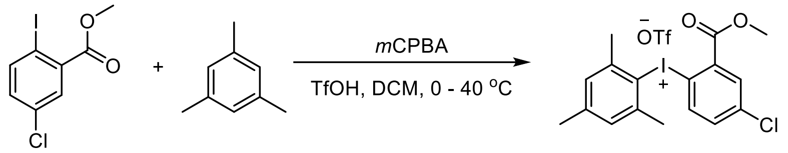


5-Chloro-2-iodobenzoic acid methyl ester (740 mg, 2.5 mmol,), *m*CPBA (665 mg, 84% active oxidant, 3.2 mmol) and mesitylene (0.7 mL, 5 mmol) were dissolved in DCM (25 mL). The mixture was cooled to 0 °C, and TfOH (442 uL, 5 mmol) was added dropwise. The reaction was stirred for another 10 minutes at 0 °C, then warmed to 40 °C and stirred until 5-chloro-2-iodobenzoic acid methyl ester disappeared. Subsequently, the phases were separated and the aqueous phase was extracted with DCM (3 x 15 mL). The combined organic phases were dried ($MgSO_4$) and concentrated under reduced pressure. The target compound was isolated by repeated recrystallization from diethylether as a white solid (967 mg, 68.6%).

$R_f$ 0.38 (EtOAc/EtOH = 20/1).

**$^1$H NMR** (400 MHz, Chloroform-*d*) δ 8.28 (d, *J* = 1,8 Hz, 1H), 7.52 (dd, *J* = 8.8, 2.4 Hz, 1H), 7.19 (s, 2H), 6.73 (d, *J* = 8.8 Hz, 1H), 4.12 (s, 3H), 2.53 (s, 6H), 2.41 (s, 3H).

**$^{13}$C NMR** (101 MHz, $CDCl_3$) *δ* 166.8, 145.6, 143.9, 138.5, 136.9, 133.3, 130.6, 129.7, 128.9, 117.4, 109.8, 55.1, 26.8, 21.4.

**$^{19}$F NMR** (376 MHz, $CDCl_3$) *δ* -78.6 (s, 3F).

**HRMS** (ESI) m/z: $[M - OTf]^+$ Calcd for $C_{17}H_{17}ClIO_2^+$ 414.9956 ; Found 414.9961.

**(4-Bromo-2-(methoxycarbonyl)phenyl)(mesityl)iodonium trifluoromethanesulfonate (2ah)**

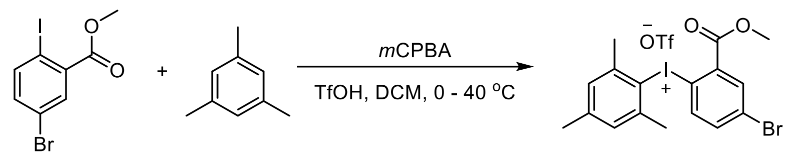


5-Bromo-2-iodobenzoic acid methyl ester (850 mg, 2.5 mmol,), *m*CPBA (665 mg, 84% active oxidant, 3.2 mmol) and mesitylene (0.7 mL, 5 mmol) were dissolved in DCM (25 mL). The mixture was cooled to 0 °C, and TfOH (442 uL, 5 mmol) was added dropwise. The reaction was stirred for another 10 minutes at 0 °C, then warmed to 40 °C and stirred until 5-bromo-2-iodobenzoic acid methyl ester disappeared. Subsequently, the phases were separated and the aqueous phase was extracted with DCM (3 x 15 mL). The combined organic phases were dried ($MgSO_4$) and concentrated under reduced pressure. The target compound was isolated by repeated recrystallization from diethylether as a white solid (1307 mg, 86%).

$R_f$ 0.40 (EtOAc/EtOH = 20/1).

**$^1$H NMR** (400 MHz, Chloroform-*d*) δ 8.41 (d, *J* = 2.4 Hz, 1H), 7.64 (dd, *J* = 8.8, 2.43 Hz, 1H), 7.18 (s, 2H), 6.64 (d, *J* = 8.8 Hz, 1H), 4.11 (s, 3H), 2.53 (s, 6H), 2.40 (s, 3H).

**$^{13}$C NMR** (101 MHz, $CDCl_3$) *δ* 166.8, 145.4, 143.7, 139.8, 136.2, 130.5, 129.9, 129.0, 126.1, 117.9, 111.8, 55.0, 26.8, 21.4.

**$^{19}$F NMR** (376 MHz, $CDCl_3$) *δ* -78.4 (s, 3F).

**HRMS** (ESI) m/z: $[M - OTf]^+$ Calcd for $C_{17}H_{17}BrIO_2^+$ 458.9451 ; Found 458.9454.

**(5-Chloro-2-(methoxycarbonyl)phenyl)(mesityl)iodonium trifluoromethanesulfonate (2ai)**

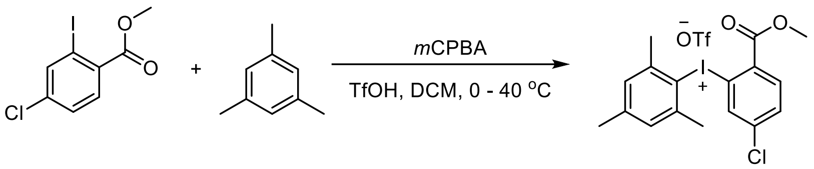


4-Chloro-2-iodobenzoic acid methyl ester (740 mg, 2.5 mmol,), *m*CPBA (665 mg, 84% active oxidant, 3.2 mmol) and mesitylene (0.7 mL, 5 mmol) were dissolved in DCM (25 mL). The mixture was cooled to 0 °C, and TfOH (442 uL, 5 mmol) was added dropwise. The reaction was stirred for another 10 minutes at 0 °C, then warmed to 40 °C and stirred 4-chloro-2-iodobenzoic acid methyl ester disappeared. Subsequently, the phases were separated and the aqueous phase was extracted with DCM (3 x 15 mL). The combined organic phases were dried ($MgSO_4$) and concentrated under reduced pressure. The target compound was isolated by repeated recrystallization from diethylether as a white solid (1018 mg, 72.2%).

$R_f$ 0.37 (EtOAc/EtOH = 20/1).

**$^1$H NMR** (400 MHz, Chloroform-*d*) δ 8.25 (d, *J* = 8.4 Hz, 1H), 7.64 (dd, *J* = 8.4, 2.0 Hz, 1H), 7.22 (s, 2H), 6.69 (d, *J* = 2.0 Hz, 1H), 4.11 (s, 3H), 2.56 (s, 6H), 2.43 (s, 3H).

**$^{13}$C NMR** (101 MHz, $CDCl_3$) *δ* 167.3, 145.7, 143.8, 134.0, 131.8, 130.6, 128.3, 126.1, 117.6, 113.7, 54.9, 26.9, 21.5.

**$^{19}$F NMR** (376 MHz, $CDCl_3$) *δ* -78.4 (s, 3F).

**HRMS** (ESI) m/z: $[M - OTf]^+$ Calcd for $C_{17}H_{17}ClIO_2^+$ 414.9956 ; Found 414.9961.

**(5-Bromo-2-(methoxycarbonyl)phenyl)(mesityl)iodonium trifluoromethanesulfonate (2aj)**

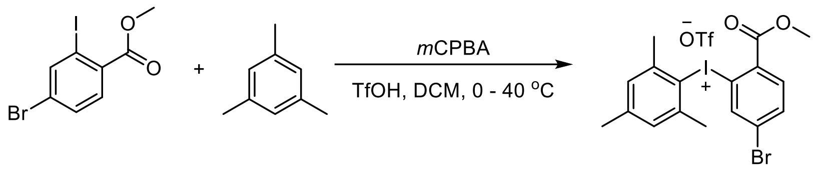


4-Bromo-2-iodobenzoic acid methyl ester (850 mg, 2.5 mmol,), *m*CPBA (665 mg, 84% active oxidant, 3.2 mmol) and mesitylene (0.7 mL, 5 mmol) were dissolved in DCM (25 mL). The mixture was cooled to 0 °C, and TfOH (442 uL, 5 mmol) was added dropwise. The reaction was stirred for another 10 minutes at 0 °C, then warmed to 40 °C and stirred until 4-bromo-2-iodobenzoic acid methyl ester disappeared. Subsequently, the phases were separated and the aqueous phase was extracted with DCM (3 x 15 mL). The combined organic phases were dried ($MgSO_4$) and concentrated under reduced pressure. The target compound was isolated by repeated recrystallization from diethylether as a white solid (1056 mg, 69.5%).

$R_f$ 0.39 (EtOAc/EtOH = 20/1).

**$^1$H NMR** (400 MHz, Chloroform-*d*) δ 8.17 (d, *J* = 8.4 Hz, 1H), 7.79 (dd, *J* = 8.4, 1.6 Hz, 1H), 7.22 (s, 2H), 6.84 (d, *J* = 2.0 Hz, 1H), 4.10 (s, 3H), 2.56 (s, 6H), 2.44 (s, 3H).

**$^{13}$C NMR** (101 MHz, $CDCl_3$) *δ* 167.4, 145.7, 143.8, 134.8, 134.1, 132.2, 131.2, 130.6, 125.8, 117.6, 113.8, 55.0, 26.9, 21.5.

**$^{19}$F NMR** (376 MHz, $CDCl_3$) *δ* -80.9 (s, 3F).

**HRMS** (ESI) m/z: $[M - OTf]^+$ Calcd for $C_{17}H_{17}BrIO_2^+$ 458.9451 ; Found 458.9454.

**Mesityl(2-(methoxycarbonyl)thiophen-3-yl)iodonium trifluoromethanesulfonate (2ak)**

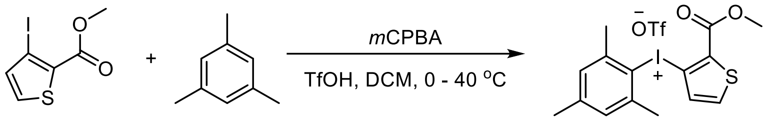


Methyl-3-iodothiophene-2-carboxylate (670 mg, 2.5 mmol), *m*CPBA (665 mg, 84% active oxidant, 3.2 mmol) and mesitylene (0.7 mL, 5 mmol) were dissolved in DCM (25 mL). The mixture was cooled to 0 °C, and TfOH (442 uL, 5 mmol) was added dropwise. The reaction was stirred for another 10 minutes at 0 °C, then warmed to 40 °C and stirred until 6-fluoro-2-iodobenzoic acid methyl ester disappeared. Subsequently, the phases were separated and the aqueous phase was extracted with DCM (3 x 15 mL). The combined organic phases were dried ($MgSO_4$) and concentrated under reduced pressure. The target compound was isolated by repeated recrystallization from diethylether as a brown solid (442 mg, 33%).

$R_f$ 0.42 (EtOAc/EtOH = 20/1).

**$^1$H NMR** (400 MHz, Chloroform-*d*) δ 7.74 (d, *J* = 5.2 Hz, 1H), 7.17 (s, 2H), 6.18 (d, *J* = 5.2 Hz, 1H), 4.04 (s, 3H), 2.57 (s, 6H), 2.40 (s, 3H).

**$^{13}$C NMR** (101 MHz, $CDCl_3$) *δ* 163.2, 145.1, 142.9, 136.9, 130.5, 129.5, 127.3, 119.8, 106.0, 54.3, 27.1, 21.3.

**$^{19}$F NMR** (376 MHz, $CDCl_3$) *δ* -78.3 (s, 3F).

**HRMS** (ESI) m/z: [M - OTf]$^+$ Calcd for $C_{15}H_{16}IO_2S^+$ 386.9910 ; Found 386.9912.

**(*Z*)-Mesityl(3-methoxy-3-oxoprop-1-en-1-yl)iodonium trifluoromethanesulfonate (2al)**

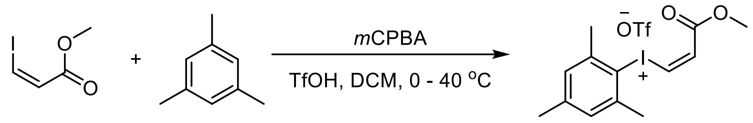


Methyl (*Z*)-3-iodoacrylate (530 mg, 2.5 mmol), *m*CPBA (665 mg, 84% active oxidant, 3.2 mmol) and mesitylene (0.7 mL, 5 mmol) were dissolved in DCM (25 mL). The mixture was cooled to 0 °C, and TfOH (442 uL, 5 mmol) was added dropwise. The reaction was stirred for another 10 minutes at 0 °C, then warmed to 40 °C and stirred until methyl (Z)-3-iodoacrylate disappeared. Subsequently, the phases were separated and the aqueous phase was extracted with DCM (3 x 15 mL). The combined organic phases were dried ($MgSO_4$) and concentrated under reduced pressure. The target compound was isolated by repeated recrystallization from diethylether as a gray solid (727 mg, 60.6%).

$R_f$ 0.36 (EtOAc/EtOH = 20/1).

**$^1$H NMR** (400 MHz, Chloroform-*d*) δ 7.52 (d, *J* = 6.8 Hz, 1H), 7.29 (d, *J* = 6.8 Hz, 1H), 7.10 (s, 2H), 3.97 (s, 3H), 2.57 (s, 6H), 2.35 (s, 3H).

**$^{13}$C NMR** (101 MHz, $CDCl_3$) *δ* 167.8, 144.6, 142.3, 130.3, 128.3, 121.5, 120.0, 54.6, 27.1, 21.2.

**$^{19}$F NMR** (376 MHz, $CDCl_3$) *δ* -78.5 (s, 3F).

**HRMS** (ESI) m/z: $[M - OTf]^+$ Calcd for $C_{13}H_{16}IO_2^+$ 331.0190 ; Found 331.0196.

**Mesityl(2-(methoxycarbonyl)-1-methyl-1H-indol-3-yl)iodonium trifluoromethanesulfonate (2ao)**

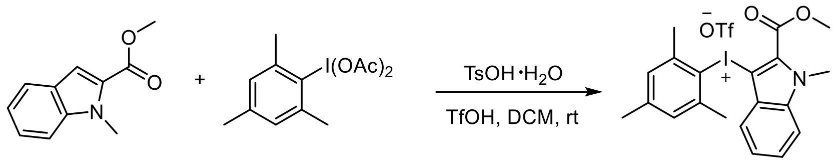


To a solution of mesityl-$\lambda^3$-iodanediyl diacetate (382 mg, 1.05 mmol) in DCM (10 mL) was added TsOH·$H_2O$ (228 mg, 1.2 mmol) and the resulting suspension was stirred for 5 min at room temperature. Then, a solution of indole (189 mg, 1.0 mmol) in DCM (5 mL) was added rapidly to the stirred suspension. The reaction mixture was monitored by TLC until indole disappeared. The solution of $CF_3SO_3H$ (5 mL, 1 mol/L aqueous solution ) was added to the reaction mixture and stirred with another 1 h.Subsequently, the phases were separated and the aqueous phase was extracted with DCM (3 x 15 mL). The combined organic phases were dried (MgSO4) and concentrated under reduced pressure. The target compound was isolated by repeated recrystallization from diethylether as a gray solid (380 mg, 65.5%).

$R_f$ 0.45 (EtOAc/EtOH = 20/1).

**$^1$H NMR** (400 MHz, Chloroform-*d*) δ 7.50 (d, *J* = 8.8 Hz, 1H), 7.45 – 7.39 (m, 1H), 7.11 (s, 2H), 7.08 – 7.04 (m, 1H), 6.52 (m, 1H), 4.17 (s, 3H), 4.16 (s, 3H), 2.62 (s, 6H), 2.38 (s, 3H).

**$^{13}$C NMR** (101 MHz, $CDCl_3$) *δ* 160.9, 144.6, 143.3, 140.0, 130.4, 128.6, 127.3, 126.2, 123.8, 119.5, 118.3, 112.1, 79.3, 53.9, 33.5, 27.2, 21.3.

**$^{19}$F NMR** (376 MHz, $CDCl_3$) *δ* -78.3 (s, 3F).

**HRMS** (ESI) m/z: $[M - OTf]^+$ Calcd for $C_{20}H_{21}INO_2^+$ 434.0612 ; Found 434.0618.

## Scope of the alkynes

### 4-Butyl-3-phenyl-1*H*-isochromen-1-one (3a)

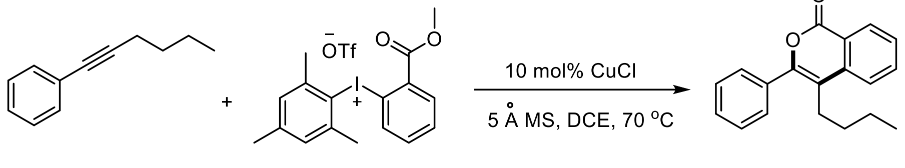


0.2 mmol 0.24 mmol 84%, 12/1

1-Phenyl-1-hexyne (31.6 mg, 0.2 mmol), mesityl (2-(methoxycarbonyl)phenyl) iodonium trifluoromethanesulfonate (127.2 mg, 0.24 mmol), copper(I) chloride (2 mg, 0.02 mmol) and 5 Å MS (20 mg) were added to a dried vial sequentially. The reaction was stirred at 70 ºC after added anhydrous DCE (4 mL). The reaction mixture was stirred until complete consumption of starting material was observed by TLC. Then the reaction mixture was concentrated in vacuum and separated on silica gel by PE/EA gradient (10:1) to get the target product (46 mg, 84% yield, regioselectivity 12/1). (Regioselectivity was determined by crude sample NMR.)

$R_f$ 0.36 (Petroleum ether/EtOAc = 10/1).

**$^1$H NMR** (400 MHz, Chloroform-*d*) δ 8.38 (d, *J* = 8.0 Hz, 1H), 7.79 (t, *J* = 8.0 Hz, 1H), 7.65 (d, *J* = 8.0 Hz, 1H), 7.58 – 7.42 (m, 6H), 2.70 – 2.66 (m, 2H), 1.68 – 1.59 (m, 2H), 1.41 – 1.32 (m, 2H), 0.89 (t, *J* = 7.2 Hz, 3H).

The titled compound is known. It’s $^1$H-NMR is in accordance with the known literature[1].

**4-Butyl-3-(4-fluorophenyl)-1*H*-isochromen-1-one (3b)**

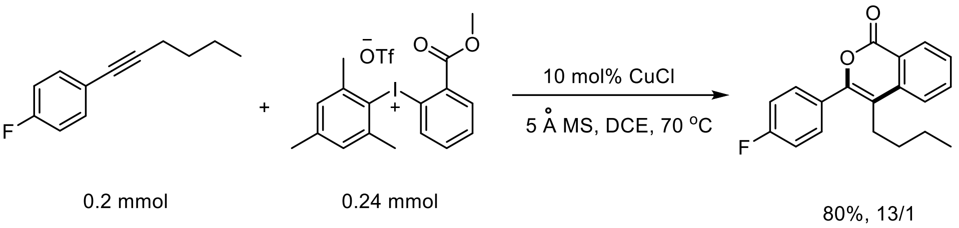


1-Fluoro-4-(hex-1-yn-1-yl)benzene (35.2 mg, 0.2 mmol), mesityl (2-(methoxycarbonyl)phenyl) iodonium trifluoromethanesulfonate (127.2 mg, 0.24 mmol), copper(I) chloride (2 mg, 0.02 mmol) and 5 Å MS (20 mg) were added to a dried vial sequentially. The reaction was stirred at 70 °C after added anhydrous DCE (4 mL). The reaction mixture was stirred until complete consumption of starting material was observed by TLC. Then the reaction mixture was concentrated in vacuum and separated on silica gel by PE/EA gradient (10:1) to get the target product (48 mg, 80% yield, regioselectivity 13/1). (Regioselectivity was determined by crude sample NMR.)

$R_f$ 0.65 (Petroleum ether/EtOAc = 5/1).

**$^1$H NMR** (400 MHz, Chloroform-*d*) δ 8.38 (dd, *J* = 1.6 Hz, 8.0 Hz, 1H), 7.78-7.82 (m, 1H),7.64 (d, *J* = 8.0 Hz, 1H), 7.57-7.52 (m, 3H ), 7.19-7.12 (m, 2H ), 2.67-2.63 (m, 2H), 1.65-1.58 (m, 2H), 1.41-1.31 (m, 2H), 0.89 (t, *J* = 7.6 Hz, 3H).

**$^{13}$C NMR** (101 MHz, $CDCl_3$) *δ* 164.5, 162.5, 150.6, 137.9, 134.9, 131.2 (d, *J* = 8.6 Hz), 130.2, 129.8 (d, *J* = 3.1 Hz), 128.1, 123.6, 121.4, 115.6 (d, *J* = 21.8 Hz), 114.4, 32.2, 26.7, 22.8, 13.9.

**$^{19}$F NMR** (376 MHz, $CDCl_3$) *δ* -110.8 ~ -110.9 (m, 1 F).

**HRMS** (ESI) m/z: $[M + H]^+$ Calcd for $C_{19}H_{18}FO_2^+$ 297.1285 ; Found 297.1283.

**4-Butyl-3-(4-methoxyphenyl)-1*H*-isochromen-1-one (3c)**

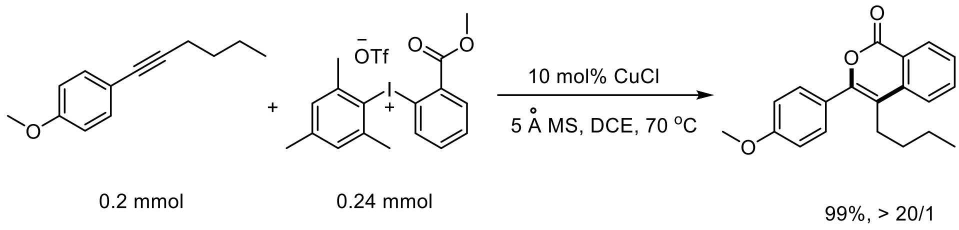


1-Methoxy-4-(hex-1-yn-1-yl)benzene (37.6 mg, 0.2 mmol), mesityl (2-(methoxycarbonyl)phenyl) iodonium trifluoromethanesulfonate (127.2 mg, 0.24 mmol), copper(I) chloride (2 mg, 0.02 mmol) and 5 Å MS (20 mg) were added to a dried vial sequentially. The reaction was stirred at 70 °C after added anhydrous DCE (4 mL). The reaction mixture was stirred until complete consumption of starting material was observed by TLC. Then the reaction mixture was concentrated in vacuum and separated on silica gel by PE/EA gradient (10:1) to get the target product (61 mg, 99% yield, regioselectivity > 20/1). (Regioselectivity was determined by crude sample NMR.)

$R_f$ 0.52 (Petroleum ether/EtOAc = 5/1).

**$^1$H NMR** (400 MHz, Chloroform-*d*) δ 8.37 (d, *J* = 8.0 Hz, 1H), 7.78 (t, *J* = 8.4 Hz, 1H), 7.63 (d, *J* = 8.0 Hz, 1H), 7.53 – 7.48 (m, 3H), 6.99 – 6.96 (m, 2H), 3.86 (s, 3H), 2.70 – 2.65 (m, 2H), 1.66 – 1.59 (m, 2H), 1.43 – 1.33 (m, 2H), 0.90 (t, *J* = 7.6 Hz, 3H).

**$^{13}$C NMR** (101 MHz, $CDCl_3$) *δ* 162.7, 160.4, 151.6, 138.2, 134.7, 130.6, 130.0, 127.7, 126.1, 123.5, 121.3, 113.8, 113.7, 55.5, 32.3, 26.8, 22.9, 13.9.

**HRMS** (ESI) m/z: $[M + H]^+$ Calcd for $C_{20}H_{21}O_3^+$ 309.1485 ; Found 309.1481.

**4-(3-Chloropropyl)-3-(4-methoxyphenyl)-1*H*-isochromen-1-one (3d)**

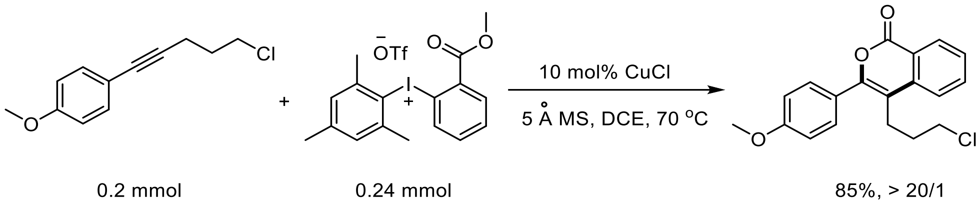


1-(5-Chloropent-1-yn-1-yl)-4-methoxybenzene (41.6 mg, 0.2 mmol), mesityl (2-(methoxycarbonyl)phenyl) iodonium trifluoromethanesulfonate (127.2 mg, 0.24 mmol), copper(I) chloride (2 mg, 0.02 mmol) and 5 Å MS (20 mg) were added to a dried vial sequentially. The reaction was stirred at 70 °C after added anhydrous DCE (4 mL). The reaction mixture was stirred until complete consumption of starting material was observed by TLC. Then the reaction mixture was concentrated in vacuum and separated on silica gel by PE/EA gradient (10:1) to get the target product (56 mg, 85% yield, regioselectivity > 20/1). (Regioselectivity was determined by crude sample NMR.)

$R_f$ 0.35 (Petroleum ether/EtOAc = 5/1).

**$^1$H NMR** (400 MHz, Chloroform-*d*) δ 8.37 (d, *J* = 8.0 Hz, 1H), 7.79 (t, *J* = 7.2 Hz, 1H),7.66 (d, *J* = 8.0 Hz, 1H), 7.54 – 7.47 (m, 3H), 6.97 (d, *J* = 8.4 Hz, 2H), 3.86 (s, 3H), 3.55 (t, *J* = 6.4 Hz, 2H), 2.89 – 2.85 (m, 2H), 2.11 – 2.04 (m, 2H).

**$^{13}$C NMR** (101 MHz, $CDCl_3$) *δ* 162.5, 160.6, 152.3, 137.7, 134.9, 130.6, 130.2, 127.9, 125.6, 123.3, 121.2, 114.0, 112.1, 55.5, 44.7, 32.6, 24.6.

**HRMS** (ESI) m/z: $[M + H]^+$ Calcd for $C_{19}H_{18}ClO_3^+$ 329.0939 ; Found 329.0937.

**2-(4-(3-(4-Methoxyphenyl)-1-*oxo*-1*H*-isochromen-4-yl)butyl)isoindoline-1,3-dione (3e)**

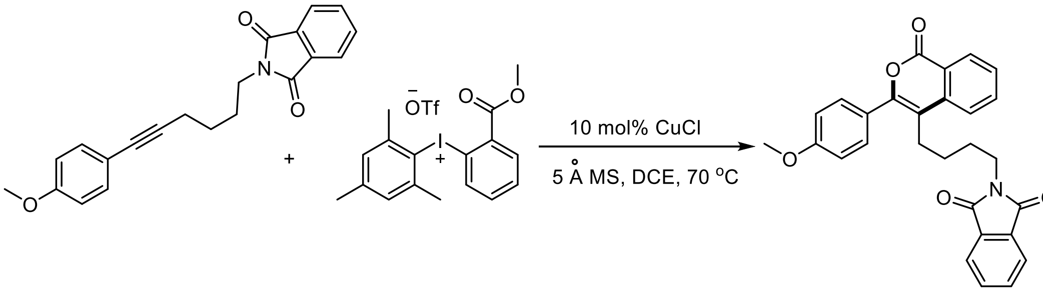


2-(6-(4-Methoxyphenyl)hex-5-yn-1-yl)isoindoline-1,3-dione (66.6 mg, 0.2 mmol), mesityl (2-(methoxycarbonyl)phenyl) iodonium trifluoromethanesulfonate (127.2 mg, 0.24 mmol), copper(I) chloride (2 mg, 0.02 mmol) and 5 Å MS (20 mg) were added to a dried vial sequentially. The reaction was stirred at 70 °C after added anhydrous DCE (4 mL). The reaction mixture was stirred until complete consumption of starting material was observed by TLC. Then the reaction mixture was concentrated in vacuum and separated on silica gel by PE/EA gradient (5:1) to get the target product (77 mg, 85% yield, regioselectivity 9/1). (Regioselectivity was determined by $^{1}$H NMR of the purified product. )

$R_f$ 0.37 (Petroleum ether/EtOAc = 2/1).

**$^{1}$H NMR** (400 MHz, Chloroform-*d*) δ 8.32 (d, *J* = 7.6 Hz, 1H), 7.81 – 7.68 (m, 5H), 7.58 (d, *J* = 8.0 Hz, 1H), 7.49 – 7.43 (m, 3H), 6.93 (d, *J* = 8.8 Hz, 2H), 3.85 (s, 3H), 3.63 (t, *J* = 6.8 Hz, 2H), 2.71 (t, *J* = 7.6 Hz, 2H), 1.75 – 1.61 (m, 4H).

**$^{13}$C NMR** (101 MHz, $CDCl_3$) *δ* 168.4, 162.5, 160.4, 151.8, 137.8, 134.8, 134.0, 132.0, 130.5, 130.0, 127.7, 125.8, 123.3, 123.3, 121.1, 113.8, 113.0, 55.4, 37.6, 28.5, 27.2, 26.6.

**HRMS** (ESI) m/z: $[M + H]^+$ Calcd for $C_{28}H_{24}NO_5^+$ 454.1649 ; Found 454.1647.

**4-Isopropyl-3-(4-methoxyphenyl)-1*H*-isochromen-1-one (3f)**

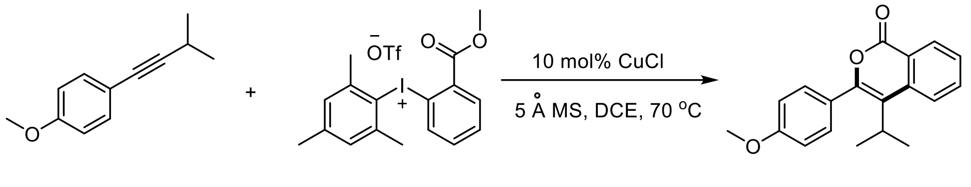


1-Methoxy-4-(3-methylbut-1-yn-1-yl)benzene (34.8 mg, 0.2 mmol), mesityl (2-(methoxycarbonyl)phenyl) iodonium trifluoromethanesulfonate (127.2 mg, 0.24 mmol), copper(I) chloride (2 mg, 0.02 mmol) and 5 Å MS (20 mg) were added to a dried vial sequentially. The reaction was stirred at 70 °C after added anhydrous DCE (4 mL). The reaction mixture was stirred until complete consumption of starting material was observed by TLC. Then the reaction mixture was concentrated in vacuum and separated on silica gel by PE/EA gradient (10:1) to get the target product (49 mg, 83% yield, regioselectivity > 20/1). (Regioselectivity was determined by crude sample NMR.)

$R_f$ 0.30 (Petroleum ether/EtOAc = 8/1).

**$^1$H NMR** (400 MHz, Chloroform-*d*) δ 8.40 (dd, *J* = 7.9, 1.5 Hz, 1H), 7.93 (d, *J* = 8.0 Hz, 1H), 7.74 (m, 1H), 7.56 – 7.35 (m, 3H), 7.08 – 6.79 (m, 2H), 3.87 (s, 3H), 3.33 (m, 1H), 1.43 (s, 3H), 1.41 (s, 3H).

**$^{13}$C NMR** (101 MHz, $CDCl_3$) *δ* 162.7, 160.5, 151.5, 137.4, 134.0, 130.8, 130.5, 127.6, 126.7, 125.1, 122.0, 118.5, 113.8, 55.5, 28.6, 21.9.

**HRMS** (ESI) m/z: $[M + H]^+$ Calcd for $C_{19}H_{19}O_3^+$ 295.1329 ; Found 295.1323.

**4-Cyclopropyl-3-(4-methoxyphenyl)-1*H*-isochromen-1-one (3g)**

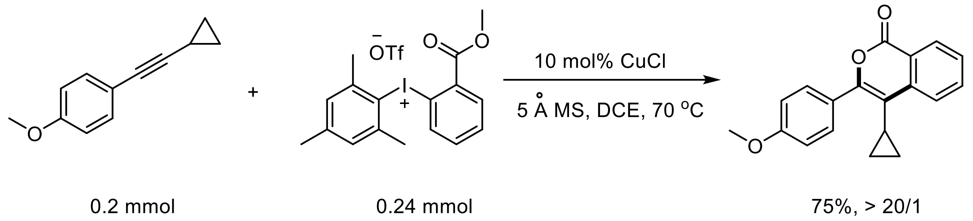


1-(Cyclopropylethynyl)-4-methoxybenzene (34.4 mg, 0.2 mmol), mesityl (2-(methoxycarbonyl)phenyl) iodonium trifluoromethanesulfonate (127.2 mg, 0.24 mmol), copper(I) chloride (2 mg, 0.02 mmol) and 5 Å MS (20 mg) were added to a dried vial sequentially. The reaction was stirred at 70 °C after added anhydrous DCE (4 mL). The reaction mixture was stirred until complete consumption of starting material was observed by TLC. Then the reaction mixture was concentrated in vacuum and separated on silica gel by PE/EA gradient (10:1) to get the target product (44 mg, 75% yield, regioselectivity > 20/1). (Regioselectivity was determined by crude sample NMR.)

$R_f$ 0.41 (Petroleum ether/EtOAc = 5/1).

**$^1$H NMR** (400 MHz, Chloroform-*d*) δ 8.32 (d, *J* = 8.0 Hz, 1H), 8.09 (d, *J* = 8.0 Hz, 1H), 7.80 – 7.76 (m, 1H), 7.72 – 7.68 (m, 2H), 7.50 (t, *J* = 6.8 Hz, 1H), 6.97 – 6.94 (m, 2H), 3.87 (s, 3H), 1.92 – 1.85 (m, 1H), 0.98 – 0.94 (m, 2H), 0.23 – 0.19 (m, 2H).

**$^{13}$C NMR** (101 MHz, $CDCl_3$) *δ* 162.7, 160.5, 153.6, 140.1, 134.5, 131.1, 129.5, 127.6, 125.8, 124.5, 120.5, 113.6, 113.3, 55.5, 10.2, 9.2.

**HRMS** (ESI) m/z: $[M + H]^+$ Calcd for $C_{19}H_{17}O_3^+$ 293.1172 ; Found 293.1166.

**4-Cyclohexyl-3-(4-methoxyphenyl)-1*H*-isochromen-1-one (3h)**

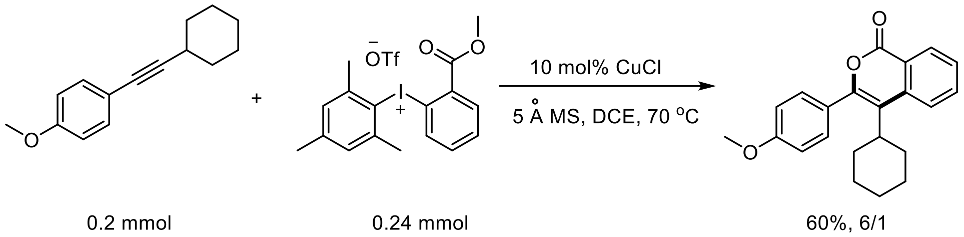


1-(Cyclohexylethynyl)-4-methoxybenzene (42.8 mg, 0.2 mmol), mesityl (2-(methoxycarbonyl)phenyl) iodonium trifluoromethanesulfonate (127.2 mg, 0.24 mmol), copper(I) chloride (2 mg, 0.02 mmol) and 5 Å MS (20 mg) were added to a dried vial sequentially. The reaction was stirred at 70 °C after added anhydrous DCE (4 mL). The reaction mixture was stirred until complete consumption of starting material was observed by TLC. Then the reaction mixture was concentrated in vacuum and separated on silica gel by PE/EA gradient (10:1) to get the target product (40 mg, 60% yield, regioselectivity 6/1). (Regioselectivity was determined by crude sample NMR.)

$R_f$ 0.34 (Petroleum ether/EtOAc = 8/1).

**$^1$H NMR** (400 MHz, Chloroform-*d*) δ 8.39 (dd, *J* = 8.0, 1.6 Hz, 1H), 8.02 (d, *J* = 8.4 Hz, 1H), 7.76 – 7.72 (m, 1H), 7.52 – 7.42 (m, 3H), 6.99 – 6.95 (m, 2H), 3.88 (s, 3H), 2.97 – 2.89 (m, 1H), 2.01 (s, 2H), 1.83 – 1.71 (m, 4H), 1.26 – 1.21 (m, 4H).

**$^{13}$C NMR** (101 MHz, $CDCl_3$) *δ* 162.6, 160.4, 151.9, 137.8, 134.0, 130.7, 130.3, 127.5, 126.8, 125.2, 121.8, 118.0, 113.7, 55.5, 39.9, 31.6, 27.1, 26.1.

**HRMS** (ESI) m/z: $[M + H]^+$ Calcd for $C_{22}H_{23}O_3^+$ 335.1642 ; Found 335.1643.

**4-Butyl-3-(2-methoxyphenyl)-1*H*-isochromen-1-one (3i)**

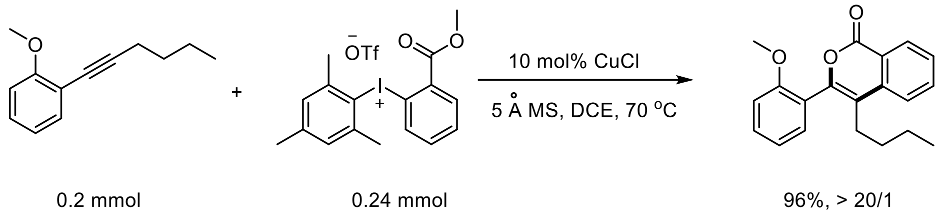


1-(Hex-1-yn-1-yl)-2-methoxybenzene (37.6 mg, 0.2 mmol), mesityl (2-(methoxycarbonyl)phenyl) iodonium trifluoromethanesulfonate (127.2 mg, 0.24 mmol), copper(I) chloride (2 mg, 0.02 mmol) and 5 Å MS (20 mg) were added to a dried vial sequentially. The reaction was stirred at 70 °C after added anhydrous DCE (4 mL). The reaction mixture was stirred until complete consumption of starting material was observed by TLC. Then the reaction mixture was concentrated in vacuum and separated on silica gel by PE/EA gradient (10:1) to get the target product (59 mg, 96% yield, regioselectivity > 20/1). (Regioselectivity was determined by crude sample NMR.)

$R_f$ 0.32 (Petroleum ether/EtOAc = 8/1).

**$^1$H NMR** (400 MHz, Chloroform-*d*) δ 8.40 – 8.37 (m, 1H), 7.80 – 7.76 (m, 1H), 7.64 – 7.62 (m, 1H), 7.55 – 7.51 (m, 1H), 7.45 – 7.40 (m, 1H), 7.32 (dd, *J* = 7.6, 2.0 Hz, 1H), 7.04 – 6.97 (m, 2H), 3.80 (s, 3H), 2.48 (s, 2H), 1.54 (s, 2H), 1.31 – 1.21 (m, 2H), 0.80 (t, *J* = 7.6 Hz, 3H).

**$^{13}$C NMR** (101 MHz, $CDCl_3$) δ 163.0, 157.6, 149.3, 137.9, 134.5, 131.3, 131.1, 130.0, 127.7, 123.5, 122.6, 121.6, 120.4, 115.4, 111.3, 55.6, 31.5, 26.8, 22.6, 13.8.

**HRMS** (ESI) m/z: $[M + H]^+$ Calcd for $C_{20}H_{21}O_3^+$ 309.1485; Found 309.1483.

**4-Hexyl-3-(1-tosyl-1H-indol-3-yl)-1*H*-isochromen-1-one (3j)**

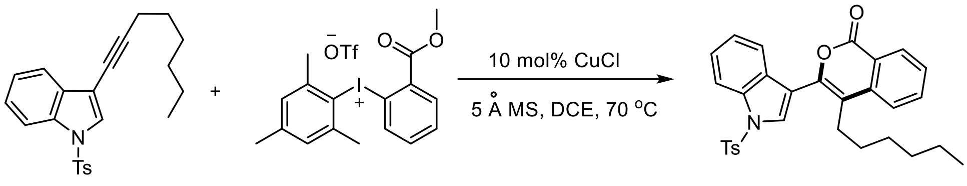


3-(Oct-1-yn-1-yl)-1-tosyl-1H-indole (75.8 mg, 0.2 mmol), mesityl (2-(methoxycarbonyl)phenyl)iodonium trifluoromethanesulfonate (127.2 mg, 0.24 mmol), copper(I) chloride (2 mg, 0.02 mmol) and 5 Å MS (20 mg) were added to a dried vial sequentially. The reaction was stirred at 70 °C after added anhydrous DCE (4 mL). The reaction mixture was stirred until complete consumption of starting material was observed by TLC. Then the reaction mixture was concentrated in vacuum and separated on silica gel by PE/EA gradient (10:1) to get the target product (90 mg, 90% yield, regioselectivity > 20/1). (Regioselectivity was determined by crude sample NMR.)

$R_f$ 0.79 (Petroleum ether/EtOAc = 2/1).

**$^1$H NMR** (400 MHz, Chloroform-*d*) δ 8.40 (dd, *J* = 8.0, 1.2 Hz, 1H), 8.03 (d, *J* = 8.4 Hz, 1H), 7.84 – 7.80 (m, 4H), 7.76 – 7.73 (m, 1H), 7.66 (d, *J* = 8.0 Hz, 1H), 7.58 – 7.54 (m, 1H), 7.41 – 7.36 (m, 1H), 7.32 – 7.25 (m, 3H), 2.75 – 2.71 (m, 2H), 2.37 (s, 3H), 1.67 – 1.60 (m, 2H), 1.40 – 1.33 (m, 2H), 1.28 – 1.24 (m, 4H), 0.90 – 0.80 (t, *J* = 6.8 Hz, 3H).

**$^{13}$C NMR** (101 MHz, $CDCl_3$) δ 162.3, 145.6, 145.1, 137.7, 134.9, 134.9, 134.6, 130.2, 130.1, 129.2, 128.1, 127.0, 126.1, 125.5, 124.1, 123.6, 121.6, 121.3, 116.1, 115.4, 113.6, 31.5, 30.1, 29.3, 27.3, 22.7, 21.7, 14.1.

**HRMS** (ESI) m/z: $[M + H]^+$ Calcd for $C_{30}H_{30}NO_4S^+$ 500.1890; Found 500.1893.

## 4-Butyl-3-(thiophen-3-yl)-1*H*-isochromen-1-one (3k)

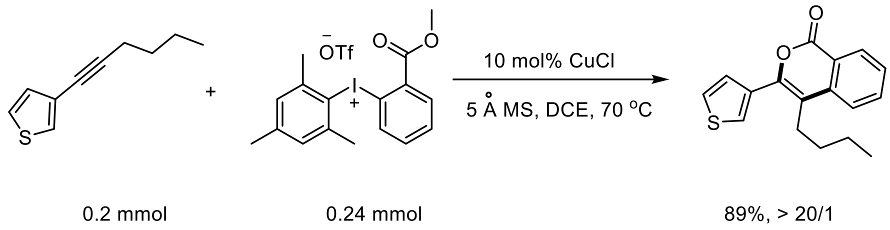


3-(Hex-1-yn-1-yl)thiophene (32.8 mg, 0.2 mmol), mesityl (2-(methoxycarbonyl) phenyl)iodonium trifluoromethanesulfonate (127.2 mg, 0.24 mmol), copper(I) chloride (2 mg, 0.02 mmol) and 5 Å MS (20 mg) were added to a dried vial sequentially. The reaction was stirred at 70 °C after added anhydrous DCE (4 mL). The reaction mixture was stirred until complete consumption of starting material was observed by TLC. Then the reaction mixture was concentrated in vacuum and separated on silica gel by PE/EA gradient (10:1) to get the target product (50 mg, 89% yield, regioselectivity > 20/1). (Regioselectivity was determined by crude sample NMR.)

$R_f$ 0.38 (Petroleum ether/EtOAc = 8/1).

**$^1$H NMR** (400 MHz, Chloroform-*d*) δ 8.37 (dd, *J* = 8.0, 1.6 Hz, 1H), 7.81 – 7.77 (m, 1H), 7.65 – 7.63 (m, 2H), 7.54 – 7.50 (m, 1H), 7.42 – 7.39 (m, 2H), 2.80 – 2.76 (m, 2H), 1.72 – 1.63 (m, 2H), 1.53 – 1.44 (m, 2H), 0.98 (t, *J* = 7.6 Hz, 3H).

**$^{13}$C NMR** (101 MHz, $CDCl_3$) δ 162.4, 147.0, 138.1, 134.8, 134.2, 130.0, 127.8, 126.2, 125.8, 123.5, 121.2, 114.0, 32.1, 26.8, 22.9, 14.0.

**HRMS** (ESI) m/z: $[M + H]^+$ Calcd for $C_{17}H_{17}O_2S^+$ 285.0944; Found 285.0942.

**4-Butyl-3-(thiophen-2-yl)-1*H*-isochromen-1-one (3l)**

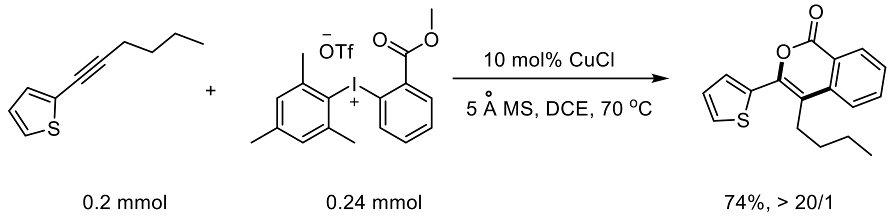


2-(Hex-1-yn-1-yl)thiophene (32.8 mg, 0.2 mmol), mesityl (2-(methoxycarbonyl) phenyl)iodonium trifluoromethanesulfonate (127.2 mg, 0.24 mmol), copper(I) chloride (2 mg, 0.02 mmol) and 5 Å MS (20 mg) were added to a dried vial sequentially. The reaction was stirred at 70 ºC after added anhydrous DCE (4 mL). The reaction mixture was stirred until complete consumption of starting material was observed by TLC. Then the reaction mixture was concentrated in vacuum and separated on silica gel by PE/EA gradient (10:1) to get the target product (42 mg, 74% yield, regioselectivity > 20/1). (Regioselectivity was determined by crude sample NMR.)

$R_f$ 0.40 (Petroleum ether/EtOAc = 8/1).

**$^1$H NMR** (400 MHz, Chloroform-*d*) δ 8.36 (dd, *J* = 8.0, 1.6 Hz, 1H), 7.81 – 7.76 (m, 1H), 7.64 (d, *J* = 8.0 Hz, 1H), 7.54 – 7.48 (m, 3H), 7.14 (q, *J* = 3.6 Hz, 1H), 2.91 – 2.87 (m, 2H), 1.73 – 1.50 (m, 4H), 1.01 (t, *J* = 7.2 Hz, 3H).

**$^{13}$C NMR** (101 MHz, $CDCl_3$) δ 161.8, 145.6, 138.1, 135.0, 134.9, 130.1, 128.6, 128.0, 127.9, 127.3, 123.5, 121.1, 114.1, 31.6, 26.9, 23.0, 14.0.

**HRMS** (ESI) m/z: $[M + H]^+$ Calcd for $C_{17}H_{17}O_2S^+$ 285.0944; Found 285.0948.

**3,4-Diphenyl-1*H*-isochromen-1-one (3m)**

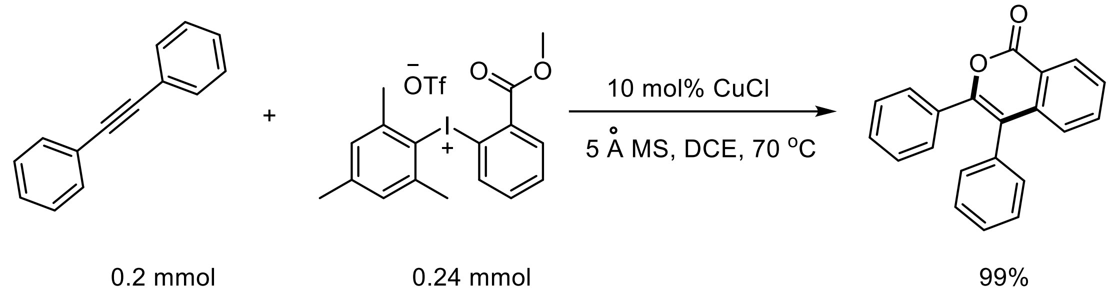


1, 2-Diphenylethyne (32.8 mg, 0.2 mmol), mesityl (2-(methoxycarbonyl) phenyl)iodonium trifluoromethanesulfonate (127.2 mg, 0.24 mmol), copper(I) chloride (2 mg, 0.02 mmol) and 5 Å MS (20 mg) were added to a dried vial sequentially. The reaction was stirred at 70 °C after added anhydrous DCE (4 mL). The reaction mixture was stirred until complete consumption of starting material was observed by TLC. Then the reaction mixture was concentrated in vacuum and separated on silica gel by PE/EA gradient (10:1) to get the target product (59 mg, 99% yield).

$R_f$ 0.35 (Petroleum ether/EtOAc = 10/1).

**$^1$H NMR** (400 MHz, Chloroform-*d*) δ 8.42 (dd, *J* = 1.6, 8.0 Hz, 1H), 7.67 – 7.63 (m, 1H), 7.55 – 7.51 (m, 1H), 7.48 – 7.39 (m, 3H), 7.36 – 7.33 (m, 2H), 7.29 – 7.18 (m, 6H).

The titled compound is known. It’s $^1$H-NMR is in accordance with the known literature.[1]

**3,4-Dibutyl-1*H*-isochromen-1-one (3n)**

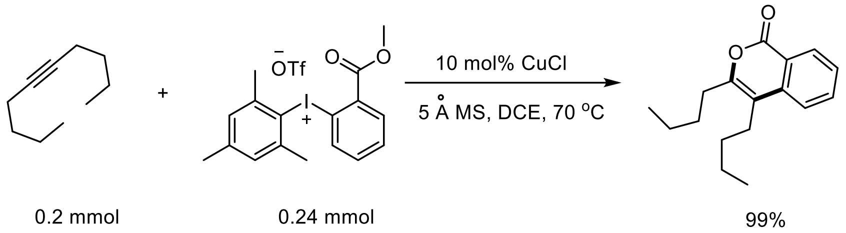


5-Decyne (27.6 mg, 0.2 mmol), mesityl(2-(methoxycarbonyl)phenyl)iodonium trifluoromethanesulfonate (127.2 mg, 0.24 mmol), copper(I) chloride (2 mg, 0.02 mmol) and 5 Å MS (20 mg) were added to a dried vial sequentially. The reaction was stirred at 70 °C after added anhydrous DCE (4 mL). The reaction mixture was stirred until complete consumption of starting material was observed by TLC. Then the reaction mixture was concentrated in vacuum and separated on silica gel by PE/EA gradient (10:1) to get the target product (51 mg, 99% yield).

$R_f$ 0.51 (Petroleum ether/EtOAc = 10/1).

**$^1$H NMR** (400 MHz, Chloroform-*d*) δ 8.31 (dd, *J* = 1.6, 8.0 Hz, 1H), 7.75 – 7.71 (m, 1H), 7.52 (d, *J* = 8.8 Hz, 1H), 7.47 – 7.43 (m, 1H), 2.59 (q, *J* = 7.2 Hz, 4H), 1.73 – 1.65 (m, 2H), 1.58 – 1.38 (m, 6H), 0.96 (m, 6H).

The titled compound is known. It's $^1$H-NMR is in accordance with the known literature.[2]

**3-Hexyl-1*H*-isochromen-1-one (3o)**

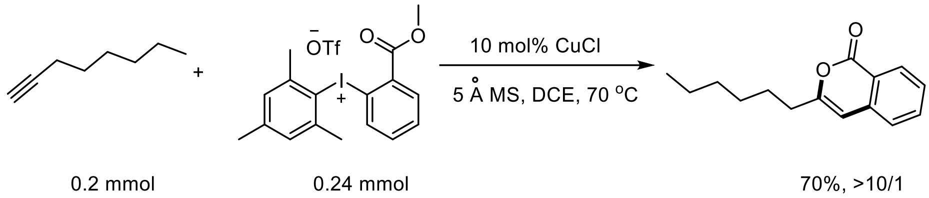


1-Octyne (22.0 mg, 0.2 mmol), mesityl(2-(methoxycarbonyl)phenyl)iodonium trifluoromethanesulfonate (127.2 mg, 0.24 mmol), copper(I) chloride (2 mg, 0.02 mmol) and 5 Å MS (20 mg) were added to a dried vial sequentially. The reaction was stirred at 70 °C after added anhydrous DCE (4 mL). The reaction mixture was stirred until complete consumption of starting material was observed by TLC. Then the reaction mixture was concentrated in vacuum and separated on silica gel by PE/EA gradient (10:1) to get the target product (32 mg, 70% yield, regioselectivity > 10/1). (Regioselectivity was determined by crude sample NMR.)

$R_f$ 0.24 (Petroleum ether/EtOAc = 10/1).

**$^1$H NMR** (400 MHz, Chloroform-*d*) δ 8.24 (d, *J* = 8.0 Hz, 1H), 7.66 (t, *J* = 7.6 Hz, 1H), 7.43 (t, *J* = 7.2 Hz, 1H), 7.34 (d, *J* = 8.0 Hz, 1H), 6.24 (s, 1H), 2.51 (t, *J* = 7.6 Hz, 2H), 1.73 – 1.66 (m, 2H), 1.42 – 1.28 (m, 6H), 0.88 (t, *J* = 6.8 Hz, 3H).

The titled compound is known. It's $^1$H-NMR is in accordance with the known literature.[3]

**3-(4-Chlorobutyl)-1*H*-isochromen-1-one (3p)**

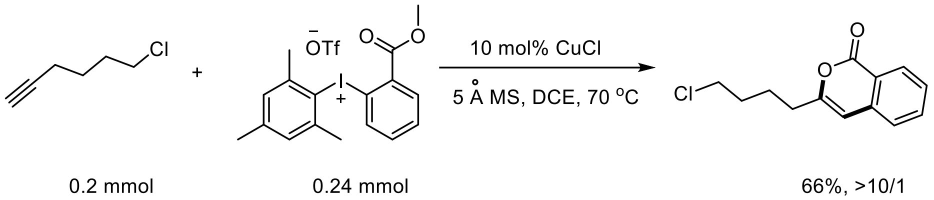


6-chlorohex-1-yne (22.0 mg, 0.2 mmol), mesityl(2-(methoxycarbonyl)phenyl) iodonium trifluoromethanesulfonate (127.2 mg, 0.24 mmol), copper(I) chloride (2 mg, 0.02 mmol) and 5 Å MS (20 mg) were added to a dried vial sequentially. The reaction was stirred at 70 °C after added anhydrous DCE (4 mL). The reaction mixture was stirred until complete consumption of starting material was observed by TLC. Then the reaction mixture was concentrated in vacuum and separated on silica gel by PE/EA gradient (10:1) to get the target product (31 mg, 66% yield, regioselectivity > 10/1). (Regioselectivity was determined by crude sample NMR.)

$R_f$ 0.22 (Petroleum ether/EtOAc = 10/1).

**$^1$H NMR** (400 MHz, Chloroform-*d*) δ 8.27 – 8.24 (m, 1H), 7.70 – 7.66 (m, 1H), 7.48 – 7.44 (m, 1H), 7.36 (d, *J* = 7.6 Hz, 1H), 6.28 (s, 1H), 3.60 – 3.55 (m, 2H), 2.60 – 2.55 (m, 2H), 1.92 – 1.82 (m, 4H).

The titled compound is known. It's $^1$H-NMR is in accordance with the known literature.[4]

**4-(1-*Oxo*-1*H*-isochromen-3-yl)butyl 4-methylbenzenesulfonate (3q)**

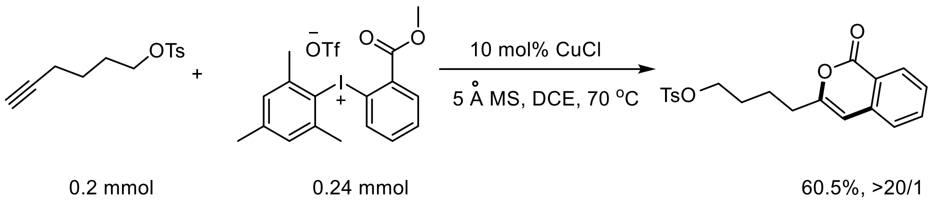


Hex-5-yn-1-yl 4-methylbenzenesulfonate (50.4 mg, 0.2 mmol), mesityl(2-(methoxycarbonyl)phenyl) iodonium trifluoromethanesulfonate (127.2 mg, 0.24 mmol), copper(I) chloride (2 mg, 0.02 mmol) and 5 Å MS (20 mg) were added to a dried vial sequentially. The reaction was stirred at 70 °C after added anhydrous DCE (4 mL). The reaction mixture was stirred until complete consumption of starting material was observed by TLC. Then the reaction mixture was concentrated in vacuum and separated on silica gel by PE/EA gradient (10:1) to get the target product (45 mg, 60.5% yield, regioselectivity > 20/1). (Regioselectivity was determined by crude sample NMR.)

$R_f$ 0.58 (Petroleum ether/EtOAc = 2/1).

**$^1$H NMR** (400 MHz, Chloroform-*d*) δ 8.23 – 8.21 (m, 1H), 7.79 – 7.75 (m, 2H), 7.69 – 7.65 (m, 1H), 7.47 – 7.43 (m, 1H), 7.35 – 7.32 (m, 3H), 6.23 (s, 1H), 4.06 – 4.02 (m, 2H), 2.50 – 2.47 (m, 2H), 2.42 (s, 3H), 1.76 – 1.69 (m, 4H).

**$^{13}$C NMR** (101 MHz, $CDCl_3$) δ 163.0, 157.0, 145.0, 137.4, 135.0, 133.0, 130.0, 129.6, 128.0, 127.9, 125.2, 120.2, 103.5, 70.0, 32.8, 28.2, 23.0, 21.7.

**HRMS** (ESI) m/z: $[M + H]^+$ Calcd for $C_{20}H_{21}O_5S^+$ 373.1104; Found 373.1101.

**Methyl (*E*)-6-(1-*oxo*-1*H*-isochromen-3-yl)hex-2-enoate (3r)**

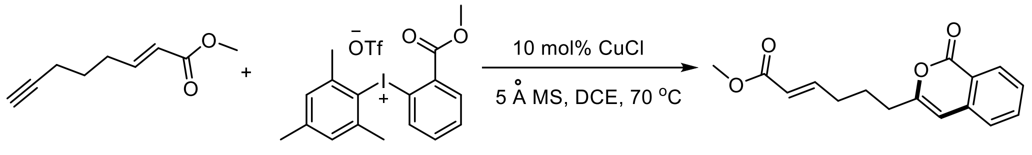


0.2 mmol 0.24 mmol 57%, >20/1

Methyl (*E*)-oct-2-en-7-ynoate (30.4 mg, 0.2 mmol), mesityl(2-(methoxycarbonyl) phenyl) iodonium trifluoromethanesulfonate (127.2 mg, 0.24 mmol), copper(I) chloride (2 mg, 0.02 mmol) and 5 Å MS (20 mg) were added to a dried vial sequentially. The reaction was stirred at 70 °C after added anhydrous DCE (4 mL). The reaction mixture was stirred until complete consumption of starting material was observed by TLC. Then the reaction mixture was concentrated in vacuum and separated on silica gel by PE/EA gradient (10:1) to get the target product (34 mg, 57% yield, regioselectivity > 20/1). (Regioselectivity was determined by crude sample NMR.)

$R_f$ 0.68 (Petroleum ether/EtOAc = 2/1).

**$^1$H NMR** (400 MHz, Chloroform-*d*) δ 8.25 – 8.23 (m, 1H), 7.69 – 7.65 (m, 1H), 7.47 – 7.43 (m, 1H), 7.35 (d, *J* = 8.0 Hz, 1H), 6.99 – 6.92 (m, 1H), 6.26 (s, 1H), 5.87 – 5.83 (m, 1H), 3.71 (s, 3H), 2.55 (t, *J* = 7.2 Hz, 2H), 2.32 – 2.26 (m, 2H), 1.93 – 1.85 (m, 2H).

**$^{13}$C NMR** (101 MHz, $CDCl_3$) δ 167.0, 163.0, 157.2, 148.2, 137.5, 134.9, 129.7, 127.9, 125.2, 121.9, 120.3, 103.6, 51.6, 32.9, 31.3, 25.3.

**HRMS** (ESI) m/z: $[M + Na]^+$ Calcd for $C_{16}H_{16}O_4Na^+$ 295.0941; Found 295.0941.

**3-Cyclopropyl-1*H*-isochromen-1-one (3s)**

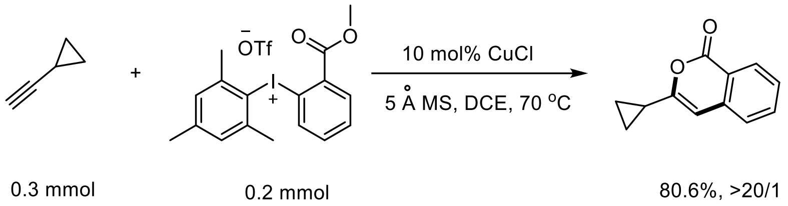


Ethynylcyclopropane (19.8 mg, 0.3 mmol), mesityl(2-(methoxycarbonyl) phenyl) iodonium trifluoromethanesulfonate (106 mg, 0.20 mmol), copper(I) chloride (2 mg, 0.02 mmol) and 5 Å MS (20 mg) were added to a dried vial sequentially. The reaction was stirred at 70 °C after added anhydrous DCE (4 mL). The reaction mixture was stirred until complete consumption of starting material was observed by TLC. Then the reaction mixture was concentrated in vacuum and separated on silica gel by PE/EA gradient (10:1) to get the target product (30 mg, 80.6% yield, regioselectivity > 20/1). (Regioselectivity was determined by crude sample NMR.)

$R_f$ 0.35 (Petroleum ether/EtOAc = 10/1).

**$^1$H NMR** (400 MHz, Chloroform-*d*) δ 8.22 – 8.20 (m, 1H), 7.66 – 7.62 (m, 1H), 7.42 – 7.38 (m, 1H), 7.31 (d, *J* = 8.0 Hz, 1H), 6.30 (s, 1H), 1.77 – 1.84 (m, 1H),1.05-1.09 (m, 2H), 0.90-0.95 (m, 2H).

The titled compound is known. It's $^1$H-NMR is in accordance with the known literature.[5]

**3-(Cyclohex-1-en-1-yl)-1*H*-isochromen-1-one (3t)**

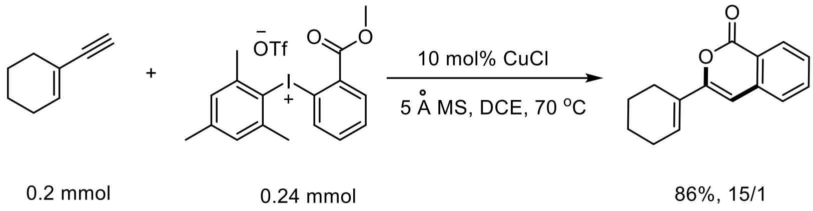


Ethynylcyclohex-1-ene (21.2 mg, 0.2 mmol), mesityl(2-(methoxycarbonyl) phenyl) iodonium trifluoromethanesulfonate (127.2 mg, 0.24 mmol), copper(I) chloride (2 mg, 0.02 mmol) and 5 Å MS (20 mg) were added to a dried vial sequentially. The reaction was stirred at 70 °C after added anhydrous DCE (4 mL). The reaction mixture was stirred until complete consumption of starting material was observed by TLC. Then the reaction mixture was concentrated in vacuum and separated on silica gel by PE/EA gradient (10:1) to get the target product (39 mg, 86% yield, regioselectivity > = 15/1). (Regioselectivity was determined by crude sample NMR.)

$R_f$ 0.65 (Petroleum ether/EtOAc = 5/1).

**$^1$H NMR** (400 MHz, Chloroform-*d*) δ 8.22 (d, *J* = 8.0 Hz, 1H), 7.63 (t, *J* = 7.6 Hz, 1H), 7.42 – 7.36 (q, *J* = 8.0 Hz, 2H), 6.80 (t, *J* = 4.4 Hz, 1H), 6.34 (s, 1H), 2.30 – 2.23 (m, 4H), 1.79 – 1.73 (m, 2H), 1.67 – 1.63 (m, 2H).

The titled compound is known. It's $^1$H-NMR is in accordance with the known literature.[6]

**3-Phenyl-1*H*-isochromen-1-one (3u)**

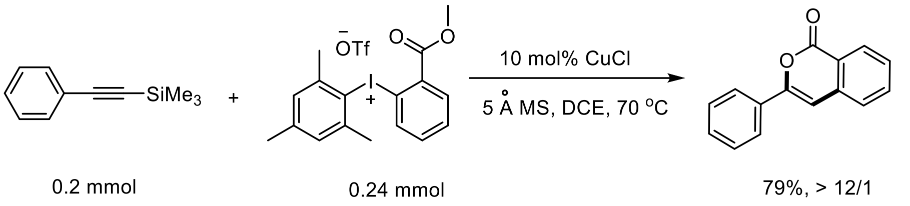


1-Phenyl-2-(trimethylsilyl)acetylene (34.8 mg, 0.2 mmol), mesityl(2-(methoxycarbonyl)phenyl) iodonium trifluoromethanesulfonate (127.2 mg, 0.24 mmol), copper(I) chloride (2 mg, 0.02 mmol) and 5 Å MS (20 mg) were added to a dried vial sequentially. The reaction was stirred at 70 °C after added anhydrous DCE (4 mL). The reaction mixture was stirred until complete consumption of starting material was observed by TLC. Then the reaction mixture was concentrated in vacuum and separated on silica gel by PE/EA gradient (20:1) to get the target product (35 mg, 79% yield, regioselectivity > 12/1). (Regioselectivity was determined by crude sample NMR.)

$R_f$ 0.14 (Petroleum ether/EtOAc = 20/1).

**$^1$H NMR** (400 MHz, Chloroform-*d*) δ 8.31 (d, *J* = 8.0 Hz, 1H), 7.89 (d, *J* = 6.4 Hz, 2H), 7.77 – 7.68 (m, 1H), 7.54 – 7.43 (m, 5H), 6.96 (s, 1H).

The titled compound is known. It's $^1$H-NMR is in accordance with the known literature.[3]

**Methyl 3-(naphthalen-1-yl)-1-*oxo*-1*H*-isochromene-4-carboxylate (3v)**

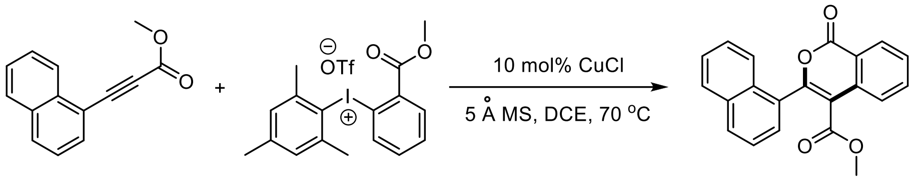


Methyl 3-(naphthalen-1-yl)propiolate (42 mg, 0.2 mmol), mesityl(2-(methoxy carbonyl)phenyl)iodonium trifluoromethanesulfonate (127.2 mg, 0.24 mmol), copper(I) chloride (2 mg, 0.02 mmol) and 5 Å MS (20 mg) were added to a dried vial sequentially. The reaction was stirred at 70 °C after added anhydrous DCE (4 mL). The reaction mixture was stirred until complete consumption of starting material was observed by TLC. Then the reaction mixture was concentrated in vacuum and separated on silica gel by PE/EA gradient (5:1) to get the target product as a white solid (45 mg, 68% yield, regioselectivity > 20/1). (Regioselectivity was determined by crude sample NMR.)

$R_f$ 0.33 (Petroleum ether/EtOAc = 5/1).

**$^1$H NMR** (400 MHz, Chloroform-*d*) δ 8.43 (dd, *J* = 8.4, 2.0 Hz, 1H),7.98 – 7.82 (m, 4H), 7.66 – 7.50 (m, 5H), 3.34 (s, 3H).

**$^{13}$C NMR** (101 MHz, Chloroform-*d*) δ 166.2, 161.2, 156.7, 135.5, 134.6, 133.5, 131.1, 131.0, 130.6, 130.1, 129.2, 128.6, 127.8, 127.4, 126.6, 125.1, 124.9, 124.8, 120.1, 113.2, 52.3.

**HRMS** (ESI) m/z: $[M + H]^+$ Calcd for $C_{21}H_{15}O_4^+$ 331.0965; Found 331.0954.

**Methyl 3-(4-methoxyphenyl)-1-*oxo*-1*H*-isochromene-4-carboxylate (3w)**

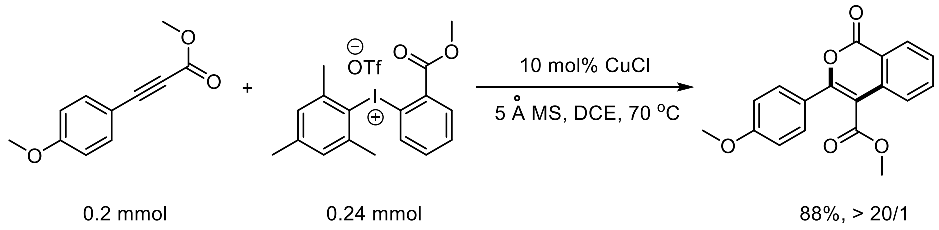


Methyl 3-(4-methoxyphenyl)propiolate (38.0 mg, 0.2 mmol), mesityl(2-(methoxy carbonyl)phenyl)iodonium trifluoromethanesulfonate (127.2 mg, 0.24 mmol), copper(I) chloride (2 mg, 0.02 mmol) and 5 Å MS (20 mg) were added to a dried vial sequentially. The reaction was stirred at 70 °C after added anhydrous DCE (4 mL). The reaction mixture was stirred until complete consumption of starting material was observed by TLC. Then the reaction mixture was concentrated in vacuum and separated on silica gel by PE/EA gradient (5:1) to get the target product as a white solid (54.5 mg, 88% yield, regioselectivity > 20/1). (Regioselectivity was determined by crude sample NMR.)

$R_f$ 0.23 (Petroleum ether/EtOAc = 5/1).

**$^1$H NMR** (400 MHz, Chloroform-*d*) δ 8.34 (dd, *J* = 8.0, 1.6 Hz, 1H), 7.79 – 7.75 (m, 1H), 7.68 (d, *J* = 8.4 Hz, 1H), 7.61 – 7.53 (m, 3H), 6.98 – 6.95 (m, 2H), 3.87 (s, 3H), 3.76 (s, 3H).

**$^{13}$C NMR** (101 MHz, Chloroform-*d*) δ 167.4, 161.6, 161.3, 155.3, 135.4, 135.0, 130.0, 129.8, 128.6, 124.9, 124.0, 119.6, 114.1, 109.6, 55.5, 52.8.

**HRMS** (ESI) m/z: $[M + H]^+$ Calcd for $C_{18}H_{15}O_5^+$ 311.0914; Found 311.0919.

**Methyl 3-(3,4-dimethoxyphenyl)-1-*oxo*-1*H*-isochromene-4-carboxylate (3x)**

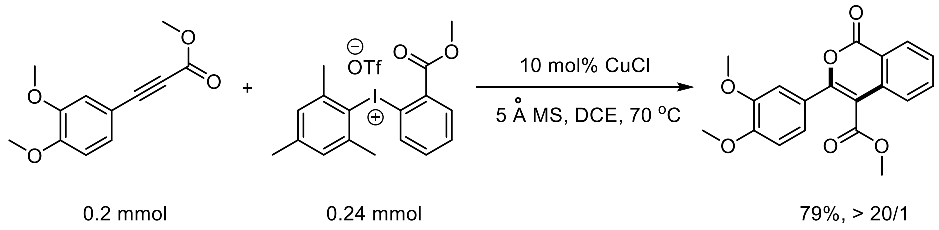

Methyl 3-(3,4-dimethoxyphenyl)propiolate (44.0 mg, 0.2 mmol), mesityl(2-(methoxy carbonyl)phenyl)iodonium trifluoromethanesulfonate (127.2 mg, 0.24 mmol), copper(I) chloride (2 mg, 0.02 mmol) and 5 Å MS (20 mg) were added to a dried vial sequentially. The reaction was stirred at 70 ºC after added anhydrous DCE (4 mL). The reaction mixture was stirred until complete consumption of starting material was observed by TLC. Then the reaction mixture was concentrated in vacuum and separated on silica gel by PE/EA gradient (2:1) to get the target product as a white solid (54 mg, 79% yield, regioselectivity > 20/1). (Regioselectivity was determined by crude sample NMR.)

$R_f$ 0.24 (Petroleum ether/EtOAc = 2/1).

**$^1$H NMR** (400 MHz, Chloroform-*d*) δ 8.34 (dd, *J* = 8.0, 1.2 Hz, 1H), 7.79 – 7.75 (m, 1H), 7.65 (d, *J* = 8.0 Hz, 1H), 7.57 – 7.53 (m, 1H), 7.23 – 7.19 (m, 2H), 6.91 (d, *J* = 8.0 Hz, 1H), 3.94 (s, 3H), 3.93 (s, 3H), 3.76 (s, 3H).

**$^{13}$C NMR** (101 MHz, Chloroform-*d*) δ 167.4, 161.3, 155.0, 151.2, 149.1, 135.4, 134.9, 130.0, 128.7, 125.0, 124.0, 121.5, 119.6, 110.8, 110.7, 110.0, 56.2, 56.1, 52.9.

**HRMS** (ESI) m/z: $[M + H]^+$ Calcd for $C_{19}H_{17}O_6^+$ 341.1020; Found 341.1011.

## Scope of the aryl(mesityl)iodonium salts

### 4-Butyl-8-methoxy-3-(4-methoxyphenyl)-1*H*-isochromen-1-one (3aa)

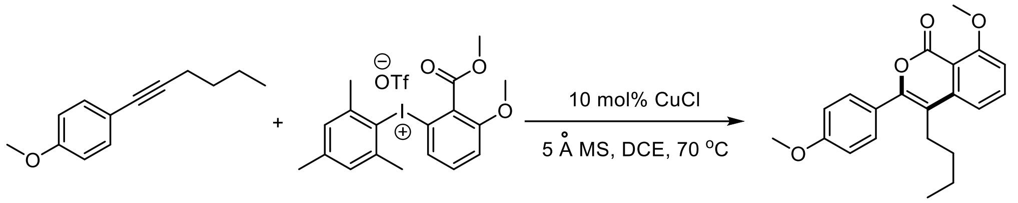


1-(Hex-1-yn-1-yl)-4-methoxybenzene (37.6 mg, 0.2 mmol), mesityl(3-methoxy-2-(methoxycarbonyl)phenyl)iodonium trifluoromethanesulfonate (134.4 mg, 0.24 mmol), copper(I) chloride (2 mg, 0.02 mmol) and 5 Å MS (20 mg) were added to a dried vial sequentially. The reaction was stirred at 70 °C after added anhydrous DCE (4 mL). The reaction mixture was stirred until complete consumption of starting material was observed by TLC. Then the reaction mixture was concentrated in vacuum and separated on silica gel by PE/EA gradient (5:1) to get the target product as a white solid ( 54 mg, 79% yield, regioselectivity > 20/1). (Regioselectivity was determined by crude sample NMR.)

$R_f$ 0.31 (Petroleum ether/EtOAc = 2/1).

**$^1$H NMR** (400 MHz, Chloroform-*d*) 7.69 – 7.66 (m, *J* = 8.0 Hz, 1H), 7.49 – 7.45 (m, 2H), 7.15 (dd, *J* = 0.8, 8.0 Hz, 1H), 6.97 – 6.92 (m, 3H), 4.00 (s, 3H), 3.83 (s, 3H), 2.62 – 2.58 (m, 2H), 1.62 – 1.54 (m, 2H), 1.38 – 1.29 (m, 2H), 0.87 (t, *J* = 7.6 Hz, 3H).

**$^{13}$C NMR** (101 MHz, Chloroform-*d*) δ 162.0, 160.3, 159.4, 152.0, 141.1, 135.5, 130.5, 126.0, 115.4, 113.6, 113.0, 110.0, 109.6, 56.4, 55.4, 32.0, 27.1, 22.8, 13.8.

**HRMS** (ESI) m/z: [M + H]$^+$ Calcd for $C_{21}H_{23}O_4^+$ 339.1591; Found 339.1592.

**Methyl 4-butyl-3-(4-methoxyphenyl)-1-*oxo*-1*H*-isochromene-8-carboxylate (3ab)**

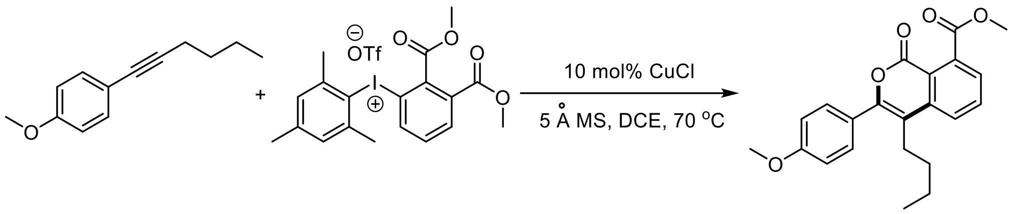


1-(Hex-1-yn-1-yl)-4-methoxybenzene (37.6 mg, 0.2 mmol), (2,3-bis(methoxy carbonyl)phenyl)(mesityl)iodonium trifluoromethanesulfonate (141.0 mg, 0.24 mmol), copper(I) chloride (2 mg, 0.02 mmol) and 5 Å MS (20 mg) were added to a dried vial sequentially. The reaction was stirred at 70 °C after added anhydrous DCE (4 mL). The reaction mixture was stirred until complete consumption of starting material was observed by TLC. Then the reaction mixture was concentrated in vacuum and separated on silica gel by PE/EA gradient (5:1) to get the target product as a white solid ( 55 mg, 75% yield, regioselectivity > 20/1). (Regioselectivity was determined by crude sample NMR.)

$R_f$ 0.69 (Petroleum ether/EtOAc = 2/1).

**$^1$H NMR** (400 MHz, Chloroform-*d*) δ 7.78 (dd, *J* = 8.0, 7.2 Hz, 1H), 7.68 (dd, *J* = 8.4, 1.2 Hz, 1H), 7.48 – 7.45 (m, 3H), 6.98 – 6.95 (m, 2H), 3.99 (s, 3H), 3.85 (s, 3H), 2.69 – 2.65 (m, 2H), 1.62 – 1.55 (m, 2H), 1.40 – 1.31 (m, 2H), 0.89 (t, *J* = 7.2 Hz, 3H).

**$^{13}$C NMR** (101 MHz, Chloroform-*d*) δ 169.9, 160.6, 160.5, 152.2, 138.9, 136.6, 134.2, 130.5, 126.1, 125.5, 124.9, 117.8, 113.8, 113.2, 55.4, 53.2, 32.1, 26.8, 22.7, 13.8.

**HRMS** (ESI) m/z: $[M + Na]^+$ Calcd for $C_{22}H_{22}O_5Na^+$ 389.1359; Found 389.1359.

**4-Butyl-8-fluoro-3-(4-methoxyphenyl)-1*H*-isochromen-1-one (3ac)**

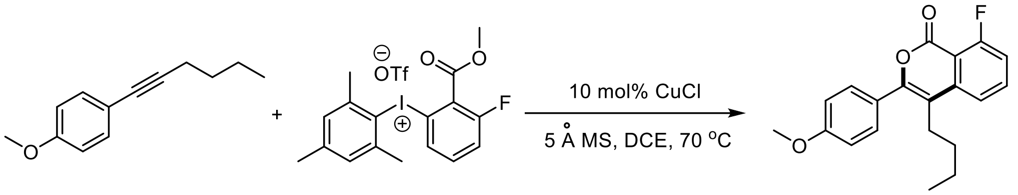


1-(Hex-1-yn-1-yl)-4-methoxybenzene (37.6 mg, 0.2 mmol), (3-fluoro-2-(methoxy carbonyl)phenyl)(mesityl)iodonium trifluoromethanesulfonate (130.6 mg, 0.24 mmol), copper(I) chloride (2 mg, 0.02 mmol) and 5 Å MS (20 mg) were added to a dried vial sequentially. The reaction was stirred at 70 °C after added anhydrous DCE (4 mL). The reaction mixture was stirred and added (3-fluoro-2-(methoxycarbonyl)phenyl) (mesityl)iodonium trifluoromethanesulfonate (16.4 mg, 0.03 mmol) until complete consumption of starting material was observed by TLC. Then the reaction mixture was concentrated in vacuum and separated on silica gel by PE/EA gradient (5:1) to get the target product (41 mg, 62% yield, regioselectivity > 20/1). (Regioselectivity was determined by crude sample NMR.)

$R_f$ 0.31 (Petroleum ether/EtOAc = 5/1).

**$^1$H NMR** (400 MHz, Chloroform-*d*) δ 7.75 – 7.70 (m, 1H), 7.50 – 7.46 (m, 2H), 7.39 (d, *J* = 8.0 Hz, 1H), 7.19 – 7.14 (m, 1H), 6.98 – 6.95 (m, 2H), 3.86 (s, 3H), 2.66 – 2.62 (m, 2H), 1.63 – 1.56 (m, 2H), 1.41 – 1.31 (m, 2H), 0.89 (t, *J* = 7.2 Hz, 3H).

**$^{13}$C NMR** (101 MHz, Chloroform-*d*) δ 163.3 (d, *J* = 266.4 Hz), 160.5, 158.2 (d, *J* = 5.1 Hz), 152.6, 140.8, 135.9 (d, *J* = 10.4 Hz), 130.5, 125.6, 119.3 (d, *J* = 4.5 Hz), 114.9 (d, *J* = 21.5 Hz), 113.8, 113.0, 110.1 (d, *J* = 6.7 Hz), 55.4, 32.0, 27.1, 22.8, 13.8.

**$^{19}$F NMR** (376 MHz, $CDCl_3$) $\delta$ -106.7 – -106.8 (m, 1F)

**HRMS** (ESI) m/z: $[M + H]^+$ Calcd for $C_{20}H_{20}FO_3^+$ 327.1391; Found 327.1391.

**4-Butyl-3-(4-methoxyphenyl)-7-methyl-1*H*-isochromen-1-one (3ad)**

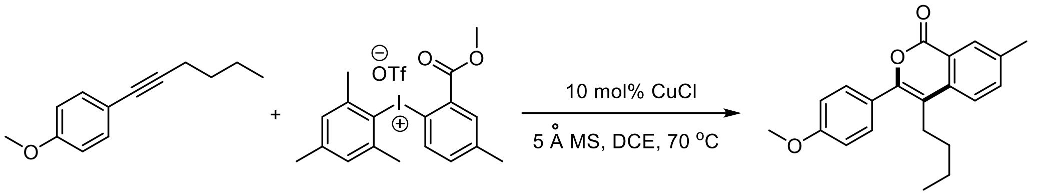


1-(Hex-1-yn-1-yl)-4-methoxybenzene (37.6 mg, 0.2 mmol), mesityl(2-(methoxy carbonyl)-4-methylphenyl)iodonium trifluoromethanesulfonate (130.6 mg, 0.24 mmol), copper(I) chloride (2 mg, 0.02 mmol) and 5 Å MS (20 mg) were added to a dried vial sequentially. The reaction was stirred at 70 °C after added anhydrous DCE (4 mL). The reaction mixture was stirred until complete consumption of starting material was observed by TLC. Then the reaction mixture was concentrated in vacuum and separated on silica gel by PE/EA gradient (8:1) to get the target product (55 mg, 86% yield, regioselectivity > 20/1). (Regioselectivity was determined by crude sample NMR.)

$R_f$ 0.30 (Petroleum ether/EtOAc = 8/1).

**$^1$H NMR** (400 MHz, Chloroform-*d*) δ 8.17 (s, 1H), 7.60 – 7.56 (dd, *J* = 8.0 Hz, 1.6 Hz, 1H), 7.52 (d, *J* = 8.4 Hz, 1H), 7.49 – 7.47 (m, 2H), 6.98 – 6.94 (m, 2H), 3.85 (s, 3H), 2.67 – 2.63 (m, 2H), 2.47 (s, 3H), 1.65 – 1.57 (m, 2H), 1.41 – 1.32 (m, 2H), 0.89 (t, *J* = 7.2 Hz, 3H).

**$^{13}$C NMR** (101 MHz, Chloroform-*d*) δ 162.9, 160.2, 150.6, 137.8, 136.0, 135.7, 130.5, 129.7, 126.1, 123.5, 121.0, 113.7, 113.6, 55.4, 32.3, 26.8, 22.8, 21.3, 13.9.

**HRMS** (ESI) m/z: $[M + H]^+$ Calcd for $C_{21}H_{23}O_3^+$ 323.1642; Found 323.1640 .

**4-Butyl-7-methoxy-3-(4-methoxyphenyl)-1*H*-isochromen-1-one (3ae)**

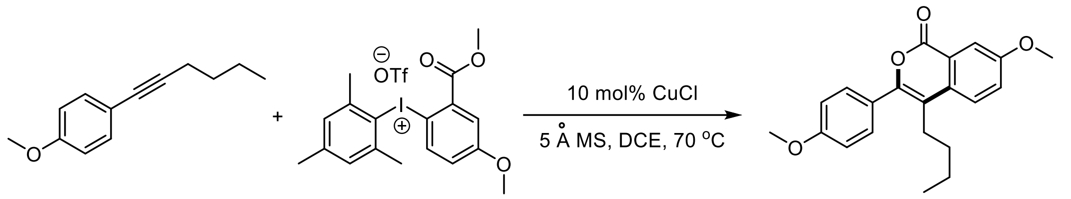


1-(Hex-1-yn-1-yl)-4-methoxybenzene (37.6 mg, 0.2 mmol), mesityl(4-methoxy-2-(methoxycarbonyl)phenyl)iodonium trifluoromethanesulfonate (134.4 mg, 0.24 mmol), copper(I) chloride (2 mg, 0.02 mmol) and 5 Å MS (20 mg) were added to a dried vial sequentially. The reaction was stirred at 70 °C after added anhydrous DCE (4 mL). The reaction mixture was stirred until complete consumption of starting material was observed by TLC. Then the reaction mixture was concentrated in vacuum and separated on silica gel by PE/EA gradient (5:1) to get the target product (48 mg, 71% yield, regioselectivity > 20/1). (Regioselectivity was determined by crude sample NMR.)

$R_f$ 0.30 (Petroleum ether/EtOAc = 5/1).

**$^1$H NMR** (400 MHz, Chloroform-*d*) δ 7.78 (d, *J* = 2.8 Hz, 1H), 7.54 (d, *J* = 8.8 Hz, 1H), 7.48 (d, *J* = 8.8 Hz, 2H), 7.36 (dd, *J* = 9.2, 3.6 Hz, 1H), 6.96 (d, *J* = 8.8 Hz, 2H), 3.92 (s, 3H), 3.86 (s, 3H), 2.69 – 2.61 (m, 2H), 1.65 – 1.57 (m, 2H), 1.41 – 1.32 (m, 2H), 0.89 (t, *J* = 7.2 Hz, 3H).

**$^{13}$C NMR** (101 MHz, Chloroform-*d*) δ 162.9, 160.2, 159.1, 149.6, 131.9, 130.6, 126.1, 125.2, 124.4, 122.4, 113.7, 113.5, 110.3, 55.9, 55.4, 32.4, 26.9, 22.8, 13.9.

**HRMS** (ESI) m/z: $[M + H]^+$ Calcd for $C_{21}H_{23}O_4^+$ 339.1591; Found 339.1588.

**4-Butyl-7-fluoro-3-(4-methoxyphenyl)-1*H*-isochromen-1-one (3af)**

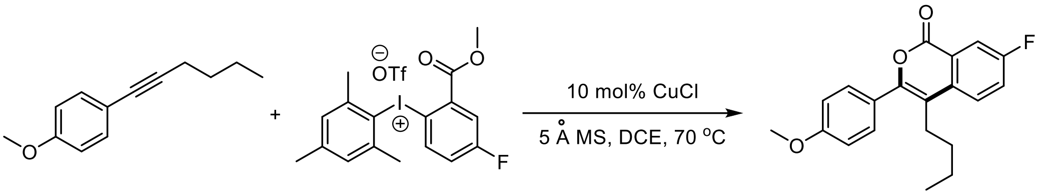


1-(Hex-1-yn-1-yl)-4-methoxybenzene (37.6 mg, 0.2 mmol), (4-fluoro-2-(methoxy carbonyl)phenyl)(mesityl)iodonium trifluoromethanesulfonate (130.6 mg, 0.24 mmol), copper(I) chloride (2 mg, 0.02 mmol) and 5 Å MS (20 mg) were added to a dried vial sequentially. The reaction was stirred at 70 °C after added anhydrous DCE (4 mL). The reaction mixture was stirred until complete consumption of starting material was observed by TLC. Then the reaction mixture was concentrated in vacuum and separated on silica gel by PE/EA gradient (5:1) to get the target product as a white solid (61 mg, 94% yield, regioselectivity > 20/1). (Regioselectivity was determined by crude sample NMR.)

$R_f$ 0.30 (Petroleum ether/EtOAc = 5/1).

**$^1$H NMR** (400 MHz, Chloroform-*d*) δ 8.02 (dd, *J* = 8.4, 2.8 Hz, 1H), 7.63 (dd, *J* = 8.8, 4.8 Hz, 1H), 7.50 – 7.46 (m, 3H), 7.00 – 6.96 (m, 2H), 3.87 (s, 3H), 2.69 – 2.65 (m, 2H), 1.65 – 1.57 (m, 2H), 1.42 – 1.33 (m, 2H), 0.90 (t, *J* = 7.6 Hz, 3H).

**$^{13}$C NMR** (101 MHz, Chloroform-*d*) δ 161.6 (d, *J* = 250.7 Hz), 161.8 (d, *J* = 3.6 Hz), 160.4, 151.0, 134.7, 130.5, 126.0 (d, *J* = 7.7 Hz), 125.7, 123.0 (d, *J* = 23.0 Hz), 122.9 (d, *J* = 7.9 Hz), 115.4 (d, *J* = 23.0 Hz), 113.8, 113.1, 55.4, 32.2, 26.9, 22.8, 13.9.

**$^{19}$F NMR** (376 MHz, $CDCl_3$) *δ* -111.7 – -111.9 (m, 1F)

**HRMS** (ESI) m/z: $[M + H]^+$ Calcd for $C_{20}H_{20}FO_3^+$ 327.1391; Found 327.1388.

**4-Butyl-7-chloro-3-(4-methoxyphenyl)-1*H*-isochromen-1-one (3ag)**

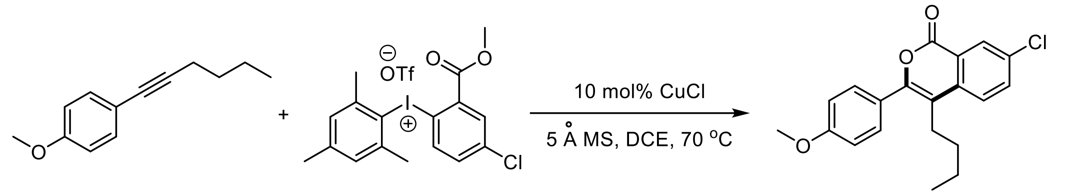


1-(Hex-1-yn-1-yl)-4-methoxybenzene (37.6 mg, 0.2 mmol), (4-chloro-2-(methoxy carbonyl)phenyl)(mesityl)iodonium trifluoromethanesulfonate (135.4 mg, 0.24 mmol), copper(I) chloride (2 mg, 0.02 mmol) and 5 Å MS (20 mg) were added to a dried vial sequentially. The reaction was stirred at 70 °C after added anhydrous DCE (4 mL). The reaction mixture was stirred until complete consumption of starting material was observed by TLC. Then the reaction mixture was concentrated in vacuum and separated on silica gel by PE/EA gradient (5:1) to get the target product as a white solid ( 56 mg, 82% yield, regioselectivity > 20/1). (Regioselectivity was determined by crude sample NMR.)

$R_f$ 0.32 (Petroleum ether/EtOAc = 8/1).

**$^1$H NMR** (400 MHz, Chloroform-*d*) δ 8.29 (d, *J* = 2.4 Hz, 1H), 7.68 (dd, *J* = 2.4, 8.8 Hz, 1H), 7.54 (d, *J* = 8.8 Hz,, 1H), 7.48 – 7.44 (m, 2H), 6.97 – 6.93 (m, 2H), 3.84 (s, 3H), 2.66 – 2.62 (m, 2H), 1.62 – 1.54 (m, 2H), 1.40 – 1.31 (m, 2H), 0.88 (t, *J* = 7.2 Hz, 3H).

**$^{13}$C NMR** (101 MHz, Chloroform-*d*) δ 161.5, 160.5, 151.8, 136.6, 135.0, 133.5, 130.5, 129.3, 125.6, 125.2, 122.4, 113.8, 113.1, 55.4, 32.1, 26.8, 22.8, 13.9.

**HRMS** (ESI) m/z: $[M + H]^+$ Calcd for $C_{20}H_{20}ClO_3^+$ 343.1096; Found 343.1088.

**7-Bromo-4-butyl-3-(4-methoxyphenyl)-1*H*-isochromen-1-one (3ah)**

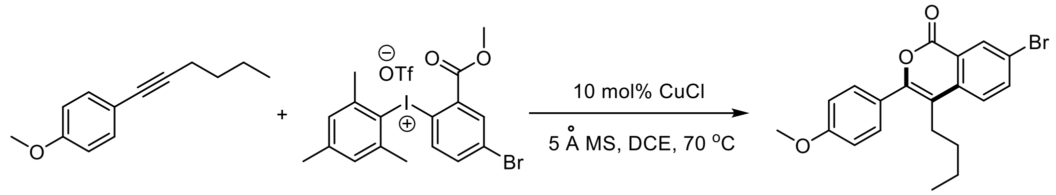


1-(Hex-1-yn-1-yl)-4-methoxybenzene (37.6 mg, 0.2 mmol), (4-bromo-2-(methoxy carbonyl)phenyl)(mesityl)iodonium trifluoromethanesulfonate (145.9 mg, 0.24 mmol), copper(I) chloride (2 mg, 0.02 mmol) and 5 Å MS (20 mg) were added to a dried vial sequentially. The reaction was stirred at 70 °C after added anhydrous DCE (4 mL). The reaction mixture was stirred until complete consumption of starting material was observed by TLC. Then the reaction mixture was concentrated in vacuum and separated on silica gel by PE/EA gradient (5:1) to get the target product as a white solid (59 mg, 76% yield, regioselectivity > 20/1). (Regioselectivity was determined by crude sample NMR.)

$R_f$ 0.29 (Petroleum ether/EtOAc = 8/1).

**$^1$H NMR** (400 MHz, Chloroform-*d*) δ 8.46 (d, *J* = 2.0 Hz, 1H), 7.84 (dd, *J* = 8.8, 2.4 Hz, 1H), 7.49 – 7.45 (m, 3H), 6.98 – 6.94 (m, 2H), 3.85 (s, 3H), 2.67 – 2.62 (m, 2H), 1.62 – 1.55 (m, 2H), 1.41 – 1.32 (m, 2H), 0.89 (t, *J* = 7.2 Hz, 3H).

**$^{13}$C NMR** (101 MHz, Chloroform-*d*) δ 161.4, 160.5, 151.9, 137.7, 137.0, 132.4, 130.5, 125.6, 125.4, 122.6, 121.2, 113.8, 113.2, 55.4, 32.1, 26.7, 22.8, 13.9.

**HRMS** (ESI) m/z: $[M + H]^+$ Calcd for $C_{20}H_{20}BrO_3^+$ 387.0590; Found 387.0590.

**4-Butyl-6-chloro-3-(4-methoxyphenyl)-1*H*-isochromen-1-one (3ai)**

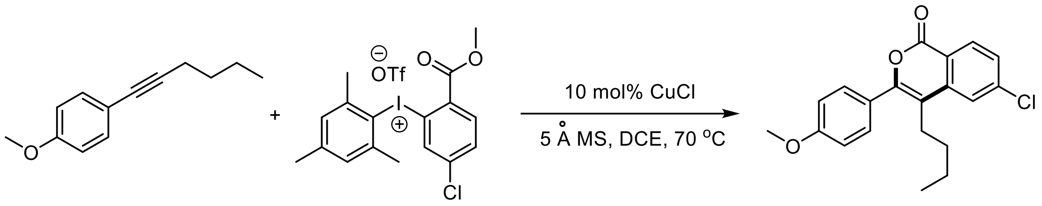


1-(Hex-1-yn-1-yl)-4-methoxybenzene (37.6 mg, 0.2 mmol), (5-chloro-2-(methoxy carbonyl)phenyl)(mesityl)iodonium trifluoromethanesulfonate (135.3 mg, 0.24 mmol), copper(I) chloride (2 mg, 0.02 mmol) and 5 Å MS (20 mg) were added to a dried vial sequentially. The reaction was stirred at 70 °C after added anhydrous DCE (4 mL). The reaction mixture was stirred until complete consumption of starting material was observed by TLC. Then the reaction mixture was concentrated in vacuum and separated on silica gel by PE/EA gradient (5:1) to get the target product as a yellow solid ( 53 mg, 78% yield, regioselectivity > 20/1). (Regioselectivity was determined by crude sample NMR.)

$R_f$ 0.30 (Petroleum ether/EtOAc = 8/1).

**$^1$H NMR** (400 MHz, Chloroform-*d*) δ 8.27 (d, *J* = 8.4 Hz, 1H), 7.55 (d, *J* = 2.0 Hz, 1H), 7.49 – 7.43 (m, 3H), 6.98 – 6.94 (m, 2H), 3.85 (s, 3H), 2.64 – 2.60 (m, 2H), 1.63 – 1.55 (m, 2H), 1.41 – 1.32 (m, 2H), 0.90 (t, *J* = 8.0 Hz, 3H).

**$^{13}$C NMR** (101 MHz, Chloroform-*d*) δ 161.9, 160.5, 152.8, 141.5, 139.7, 131.6, 130.5, 128.1, 125.6, 123.3, 119.5, 113.8, 112.8, 55.4, 32.0, 26.6, 22.7, 13.8.

**HRMS** (ESI) m/z: $[M + H]^+$ Calcd for $C_{20}H_{20}ClO_3^+$ 343.1096; Found 343.1092.

**6-Bromo-4-butyl-3-(4-methoxyphenyl)-1H-isochromen-1-one (3aj)**

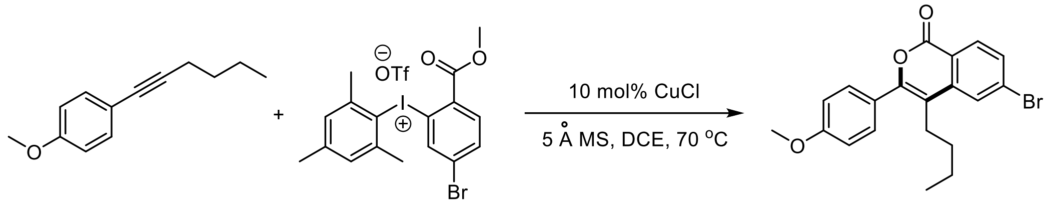


1-(Hex-1-yn-1-yl)-4-methoxybenzene (37.6 mg, 0.2 mmol), (5-bromo-2-(methoxy carbonyl)phenyl)(mesityl)iodonium trifluoromethanesulfonate (145.9 mg, 0.24 mmol), copper(I) chloride (2 mg, 0.02 mmol) and 5 Å MS (20 mg) were added to a dried vial sequentially. The reaction was stirred at 70 ºC after added anhydrous DCE (4 mL). The reaction mixture was stirred until complete consumption of starting material was observed by TLC. Then the reaction mixture was concentrated in vacuum and separated on silica gel by PE/EA gradient (5:1) to get the target product as a white solid ( 58 mg, 75% yield, regioselectivity > 20/1). (Regioselectivity was determined by crude sample NMR.)

$R_f$ 0.31 (Petroleum ether/EtOAc = 8/1).

**$^1$H NMR** (400 MHz, Chloroform-*d*) δ 8.19 (d, *J* = 8.4 Hz, 1H), 7.73 (d, *J* = 2.0 Hz, 1H), 7.62 – 7.59 (dd, *J* = 1.6, 8.4 Hz, 1H), 7.49 – 7.46 (m, 2H), 6.98 – 6.95 (m, 2H), 3.85 (s, 3H), 2.65 – 2.61 (m, 2H), 1.63 – 1.56 (m, 2H), 1.42 – 1.32 (m, 2H), 0.90 (t, *J* = 7.2 Hz, 3H).

**$^{13}$C NMR** (101 MHz, Chloroform-*d*) δ 162.0, 160.5, 152.8, 139.8, 131.6, 131.0, 130.5, 130.4, 126.4, 125.6, 119.9, 113.8, 112.7, 55.4, 32.0, 26.6, 22.7, 13.8.

**HRMS** (ESI) m/z: $[M + H]^+$ Calcd for $C_{20}H_{20}BrO_3^+$ 387.0590; Found 387.0590.

**4,5-Diphenyl-7*H*-thieno[2,3-c]pyran-7-one (3ak)**

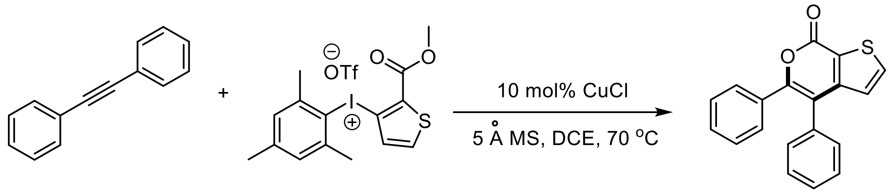


1,2-Diphenylethyne (17.8 mg, 0.1 mmol), mesityl(2-(methoxycarbonyl)thiophen-3-yl) iodonium trifluoromethanesulfonate (64.3 mg, 0.12 mmol), copper(I) chloride (1 mg, 0.01 mmol) and 5 Å MS (20 mg) were added to a dried vial sequentially. The reaction was stirred at 70 °C after added anhydrous DCE (2 mL). The reaction mixture was stirred until complete consumption of starting material was observed by TLC. Then the reaction mixture was concentrated in vacuum and separated on silica gel by PE/EA gradient (5:1) to get the target product as a white solid ( 26 mg, 85% yield).

$R_f$ 0.40 (Petroleum ether/EtOAc = 5/1).

**$^1$H NMR** (400 MHz, Chloroform-*d*) δ 7.78 (d, *J* = 5.2 Hz, 1H), 7.41 – 7.34 (m, 5H), 7.30 – 7.19 (m, 5H), 6.96 (d, *J* = 5.2 Hz, 1H).

The titled compound is known. It’s $^1$H-NMR is in accordance with the known literature.[2]

**5,6-Diphenyl-2*H*-pyran-2-one (3am)**

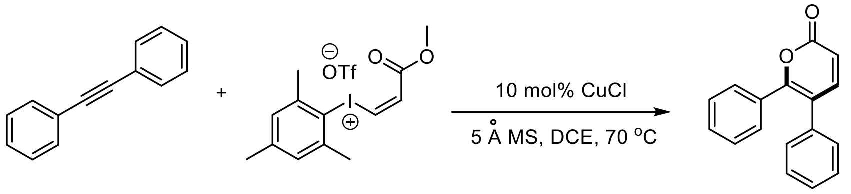


1,2-Diphenylethyne (35.6 mg, 0.2 mmol), (*Z*)-mesityl (3-methoxy-3-oxoprop-1-en-1-yl)iodonium trifluoromethanesulfonate (115.2 mg, 0.24 mmol), copper(I) chloride (2 mg, 0.02 mmol) and 5 Å MS (20 mg) were added to a dried vial sequentially. The reaction was stirred at 70 °C after added anhydrous DCE (4 mL). The reaction mixture was stirred until complete consumption of starting material was observed by TLC. Then the reaction mixture was concentrated in vacuum and separated on silica gel by PE/EA gradient (5:1) to get the target product as a white solid ( 40 mg, 81% yield).

$R_f$ 0.20 (Petroleum ether/EtOAc = 5/1).

**$^1$H NMR** (400 MHz, Chloroform-*d*) δ 7.47 (d, *J* = 9.2 Hz, 1H), 7.38 – 7.29 (m, 6H), 7.26 – 7.21 (m, 2H), 7.20 – 7.15 (m, 2H), 6.37 (d, *J* = 9.2 Hz, 1H).

The titled compound is known. It's $^1$H-NMR is in accordance with the known literature.[7]

**3-(4-Methoxyphenyl)-4-phenyl-1*H*-isochromen-1-one (3am)**

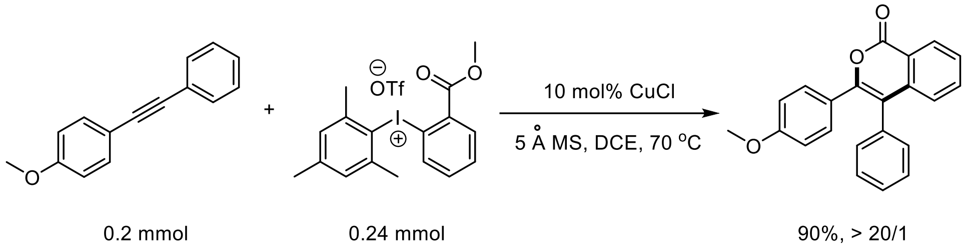


1-Methoxy-4-(phenylethynyl)benzene (41.6 mg, 0.2 mmol), mesityl(2-(methoxy carbonyl)phenyl)iodonium trifluoromethanesulfonate (127.2 mg, 0.24 mmol), copper(I) chloride (2 mg, 0.02 mmol) and 5 Å MS (20 mg) were added to a dried vial sequentially. The reaction was stirred at 70 °C after added anhydrous DCE (4 mL). The reaction mixture was stirred until complete consumption of starting material was observed by TLC. Then the reaction mixture was concentrated in vacuum and separated on silica gel by PE/EA gradient (5:1) to get the target product as a white solid ( 59 mg, 90% yield, regioselectivity > 20/1). (Regioselectivity was determined by crude sample NMR.)

$R_f$ 0.39 (Petroleum ether/EtOAc = 5/1).

**$^1$H NMR** (400 MHz, Chloroform-*d*) δ 8.39 (d, *J* = 8.0 Hz, 1H), 7.63 – 7.59 (m, 1H), 7.51 – 7.38 (m, 4H), 7.29 – 7.26 (m, 4H), 7.16 (d, *J* = 8.0 Hz, 1H), 6.71 (d, *J* = 8.4 Hz, 2H), 3.76 (s, 3H).

**$^{13}$C NMR** (101 MHz, Chloroform-*d*) δ 162.5, 160.1, 151.0, 139.3, 134.8, 134.7, 131.4, 130.8, 129.6, 129.3, 128.2, 127.9, 125.4, 125.2, 120.3, 115.9, 113.4, 55.3.

**HRMS** (ESI) m/z: $[M+H]^+$ Calcd for $C_{22}H_{17}O_3^+$ 329.1172; Found 329.1175.

**3-(4-Methoxyphenyl)-6-methyl-4-phenyl-1H-isochromen-1-one (3an)**

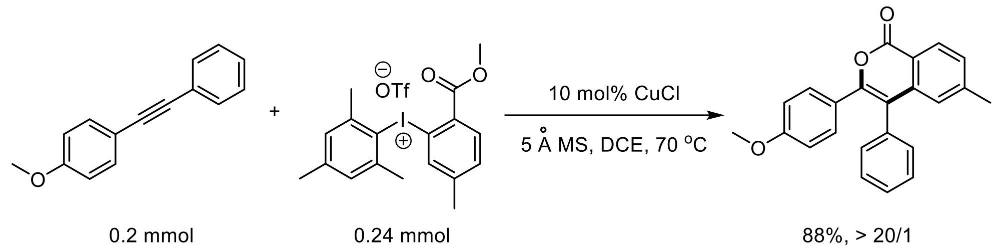


Methoxy-4-(phenylethynyl)benzene (41.6 mg, 0.2 mmol), mesityl(2-(methoxy carbonyl)-5-methylphenyl)iodonium trifluoromethanesulfonate (130.6 mg, 0.24 mmol), copper(I) chloride (2 mg, 0.02 mmol) and 5 Å MS (20 mg) were added to a dried vial sequentially. The reaction was stirred at 70 °C after added anhydrous DCE (4 mL). The reaction mixture was stirred until complete consumption of starting material was observed by TLC. Then the reaction mixture was concentrated in vacuum and separated on silica gel by PE/EA gradient (5:1) to get the target product as a white solid (60 mg, 88% yield, regioselectivity > 20/1). (Regioselectivity was determined by crude sample NMR.)

$R_f$ 0.40 (Petroleum ether/EtOAc = 5/1).

**$^1$H NMR** (400 MHz, Chloroform-*d*) δ 8.28 (d, *J* = 8.0 Hz, 1H), 7.47 – 7.38 (m, 3H), 7.31 (dd, *J* = 8.0, 2.0 Hz, 1H), 7.27 – 7.24 (m, 4H), 6.93 (q, *J* = 1.6 Hz, 1H), 6.72 – 6.68 (m, 2H), 3.76 (s, 2H), 2.36 (s, 3H).

**$^{13}$C NMR** (101 MHz, Chloroform-*d*) δ 162.6, 160.0, 151.0, 145.8, 139.3, 134.9, 131.4, 130.8, 129.7, 129.3, 129.2, 128.1, 125.5, 125.2, 117.9, 115.9, 113.4, 55.3, 22.3.

The titled compound is known. It's $^1$H-NMR is in accordance with the known literature.[8]

**3-(4-Methoxyphenyl)-9-methyl-4-phenylpyrano[3,4-b]indol-1(9H)-one (3ao)**

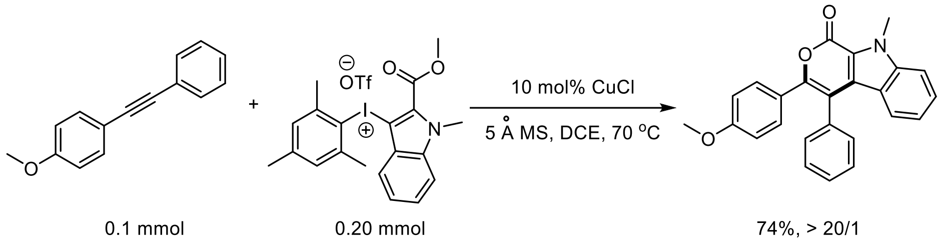


1-Methoxy-4-(phenylethynyl)benzene (20.8 mg, 0.1 mmol), mesityl(2-(methoxy carbonyl)-1-methyl-1H-indol-3-yl)iodonium trifluoromethanesulfonate (70 mg, 0.12 mmol), copper(I) chloride (2 mg, 0.02 mmol) and 5 Å MS (20 mg) were added to a dried vial sequentially. The reaction was stirred at 70 °C after added anhydrous DCE (2 mL). Diaryliodonium salt was consumed quickly and needed to replenish twice (0.8 mmol). The reaction mixture was stirred until complete consumption of the alkyne was observed by TLC. Then the reaction mixture was concentrated in vacuum and separated on silica gel by PE/EA gradient (5:1) to get the target product as a white solid (28 mg, 74% yield, regioselectivity > 20/1). (Regioselectivity was determined by crude sample NMR.)

$R_f$ 0.38 (Petroleum ether/EtOAc = 5/1).

**$^1$H NMR** (400 MHz, Chloroform-*d*) δ 7.45 (d, *J* = 3.6 Hz, 1H), 7.43 – 7.38 (m, 2H), 7.32 – 7.29 (m, 2H), 7.24 – 7.20 (m, 3H), 7.02 – 6.97 (m, 3H), 6.84 (m, 1H), 4.28 (s, 3H), 3.91 (s, 3H).

**$^{13}$C NMR** (101 MHz, Chloroform-*d*) δ 159.7, 157.2, 149.3, 141.7, 133.2, 131.9, 129.0, 128.3, 128.0, 127.5, 127.3, 126.8, 123.4, 121.8, 121.0, 120.8, 116.0, 114.6, 110.5, 55.4, 31.5.

**HRMS** (ESI) m/z: $[M + H]^+$ Calcd for $C_{25}H_{20}NO_3^+$ 382.1438; Found 382.1434.

**3-(4-Methoxyphenyl)-4-(p-tolyl)-1H-isochromen-1-one (3op)**

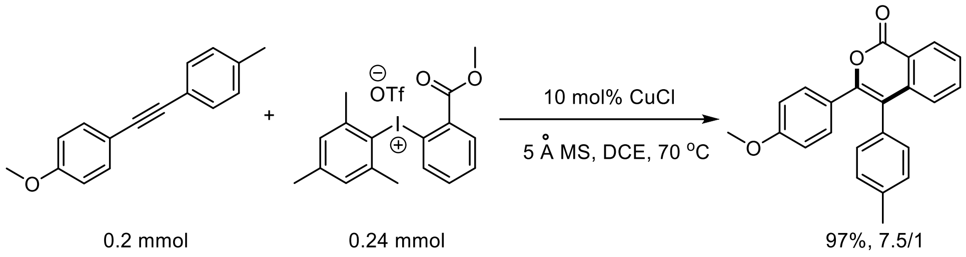


1-Methoxy-4-(*p*-tolylethynyl)benzene (44.4 mg, 0.2 mmol), mesityl(2-(methoxy carbonyl)phenyl)iodonium trifluoromethanesulfonate (127.2 mg, 0.24 mmol), copper(I) chloride (2 mg, 0.02 mmol) and 5 Å MS (20 mg) were added to a dried vial sequentially. The reaction was stirred at 70 °C after added anhydrous DCE (4 mL). The reaction mixture was stirred until complete consumption of starting material was observed by TLC. Then the reaction mixture was concentrated in vacuum and separated on silica gel by PE/EA gradient (5:1) to get the target product as a white solid (66.4 mg, 97% yield, regioselectivity = 7.5/1). (Regioselectivity was determined by crude sample NMR.)

$R_f$ 0.40 (Petroleum ether/EtOAc = 5/1).

**$^1$H NMR** (400 MHz, Chloroform-*d*) δ 8.38 (d, *J* = 8.0 Hz, 1H), 7.63 – 7.58 (m, 1H), 7.50 – 7.46 (m, 1H), 7.32 – 7.28 (m, 2H), 7.24 (d, *J* = 7.6 Hz, 2H), 7.19 – 7.13 (m, 3H), 6.74 – 6.70 (m, 2H), 3.77 (s, 3H), 2.42 (s, 3H).

**$^{13}$C NMR** (101 MHz, Chloroform-*d*) δ 162.6, 160.0, 150.9, 139.5, 137.9, 134.6,131.6, 131.2, 130.8, 130.0, 129.6, 127.8, 125.6, 125.3, 120.3, 115.8, 113.4, 55.3, 21.5.

The titled compound is known. It's $^1$H-NMR is in accordance with the known literature.[9]

## The crude reaction $^{1}$H NMR of 1-Methoxy-4-(*p*-tolylethynyl)benzene

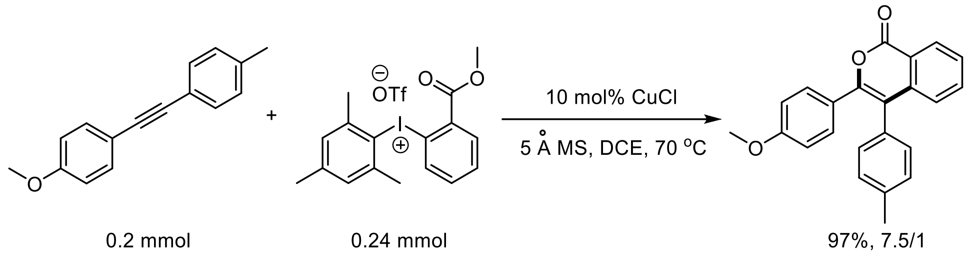


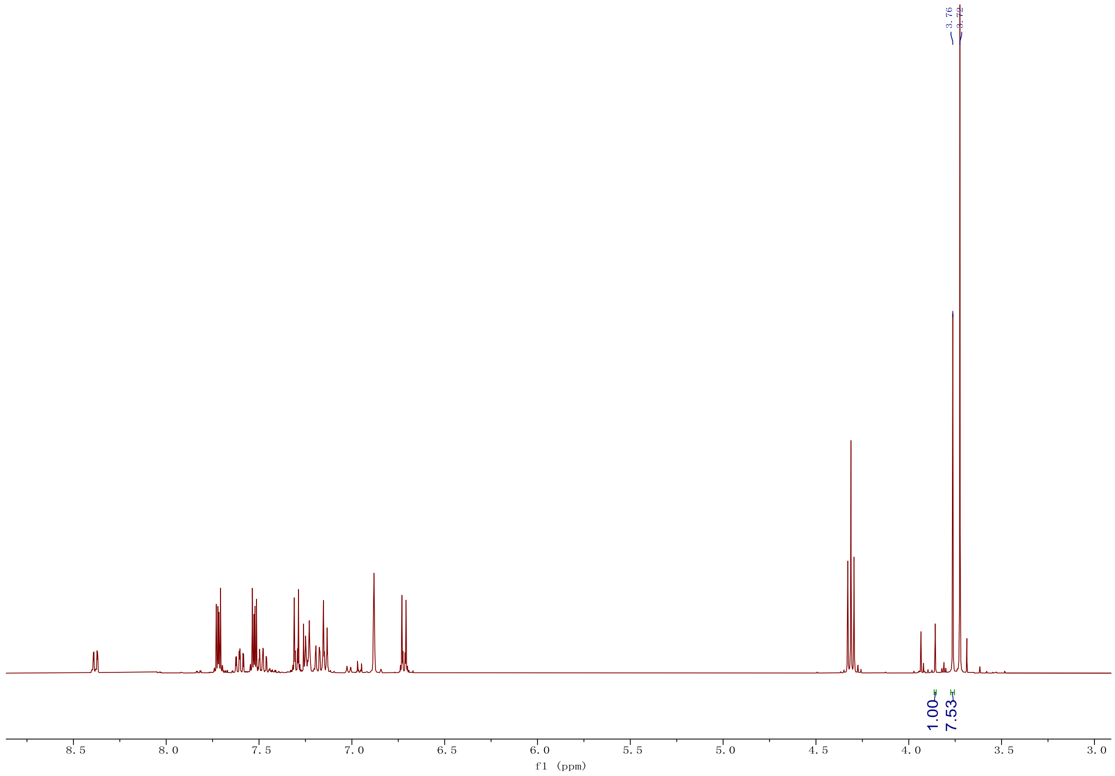

**Ethyl4-(1-oxo-3-phenyl-1*H*-isochromen-4-yl)benzoate (3aq)**

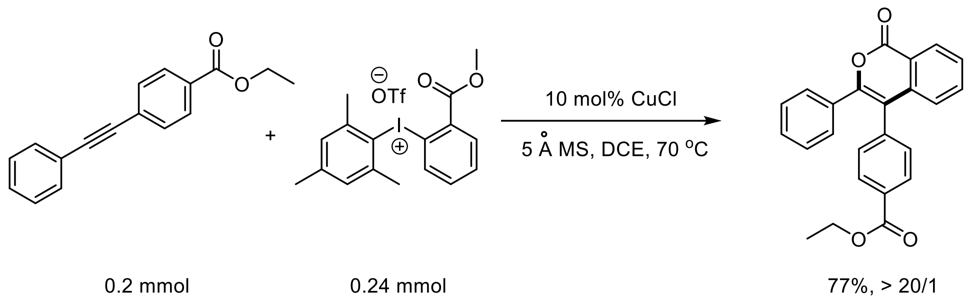


Ethyl 4-(phenylethynyl)benzoate (50.0 mg, 0.2 mmol), mesityl(2-(methoxy carbonyl)phenyl)iodonium trifluoromethanesulfonate (127.2 mg, 0.24 mmol), copper(I) chloride (2 mg, 0.02 mmol) and 5 Å MS (20 mg) were added to a dried vial sequentially. The reaction was stirred at 70 °C after added anhydrous DCE (4 mL). The reaction mixture was stirred until complete consumption of starting material was observed by TLC. Then the reaction mixture was concentrated in vacuum and separated on silica gel by PE/EA gradient (5:1) to get the target product as a white solid (57 mg, 77% yield, regioselectivity > 20/1). (Regioselectivity was determined by crude sample NMR.)

$R_f$ 0.39 (Petroleum ether/EtOAc = 5/1).

**$^1$H NMR** (400 MHz, Chloroform-*d*) δ 8.42 (dd, *J* = 8.0, 1.6 Hz, 1H), 8.12 – 8.09 (m, 2H), 7.67 – 7.63 (m, 1H), 7.57 – 7.53 (m, 1H), 7.39 – 7.14 (m, 8H), 4.42 (q, *J* = 7.2 Hz, 2H), 1.42 (t, *J* = 7.2 Hz, 3H).

**$^{13}$C NMR** (101 MHz, Chloroform-*d*) δ 166.3, 162.1, 151.4, 139.4, 138.3, 134.9, 132.7, 131.5, 130.3, 129.9, 129.4, 128.5, 128.2, 125.1, 120.5, 116.2, 61.3, 14.5.

**HRMS** (ESI) m/z: $[M + H]^+$ Calcd for $C_{24}H_{19}O_4^+$ 371.1278; Found 371.1271.

## Synthetic applications

### 3,4-Diphenyl-1H-isochromen-1-one (3m)

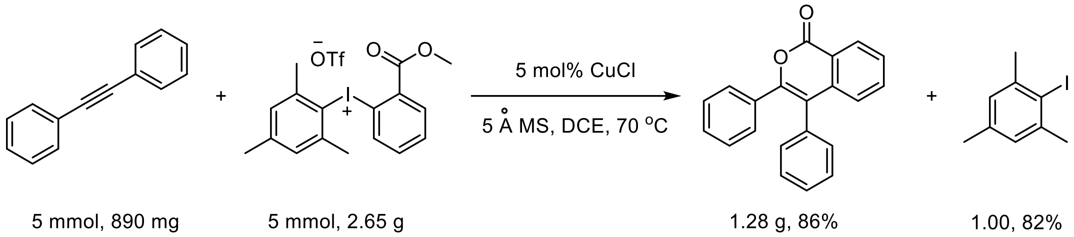


1,2-Diphenylethyne (890 mg, 5 mmol), mesityl (2-(methoxycarbonyl) phenyl)iodonium trifluoromethanesulfonate (2.65 g, 5 mmol), copper(I) chloride (24.8 mg, 0.25 mmol) and 5 Å MS (200 mg) were added to a dried vial sequentially. The reaction was stirred at 70 ºC after added anhydrous DCE (40 mL). The reaction mixture was stirred until complete consumption of starting material was observed by TLC. Then the reaction mixture was concentrated in vacuum and separated on silica gel by PE/EA gradient (10:1) to get the target product (1.28 mg, 100% yield) and recover 2,4,6-Trimethyliodobenzene (1.0 g, 82%).

$R_f$ 0.35 (Petroleum ether/EtOAc = 10/1).

**$^1$H NMR** (400 MHz, Chloroform-*d*) δ 8.42 (dd, *J* = 1.6, 8.0 Hz, 1H), 7.67 – 7.63 (m, 1H), 7.55 – 7.51 (m, 1H), 7.48 – 7.39 (m, 3H), 7.36 – 7.33 (m, 2H), 7.29 – 7.18 (m, 6H).

**3-(4-Methoxyphenyl)-1H-isochromen-1-one (6)**

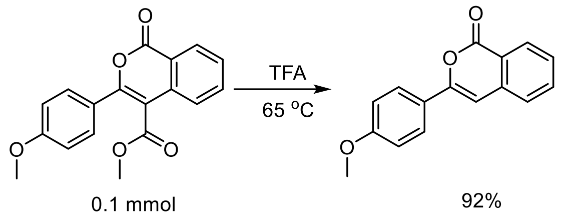

A 5.0 mL screw-cap vial was added methyl 3-(4-methoxyphenyl)-1-oxo-1H-isochromene-4-carboxylate (31 mg, 0.1 mmol) and TFA (0.8 mL) under air. After stirring at 65 °C for 6 hours, the reaction was quenched with aqueous $H_2O$ and extracted with ethyl acetate (8 mL x 3). The organic layer was washed with brine, dried over $Na_2SO_4$, and filtered through a sand core funnel. The solvent was removed under reduced pressure and the residue was separated on silica gel by PE/EA gradient (5:1) to get the target product as a white solid (15 mg, 92% yield).

$R_f$ 0.19 (Petroleum ether/EtOAc = 10/1).

**$^1$H NMR** (400 MHz, Chloroform-*d*) δ 8.29 (d, *J* = 8.8 Hz, 1H), 7.86 – 7.80 (m, 2H), 7.72 – 7.68 (m, 1H), 7.48 – 7.44 (m, 2H), 7.01 – 6.95 (m, 2H), 6.84 (s, 1H), 3.86 (s, 3H).

The titled compound is known. It's $^1$H-NMR is in accordance with the known literature.[10]

.

**3-Benzoyl-3-phenylisobenzofuran-1(3H)-one (7)**

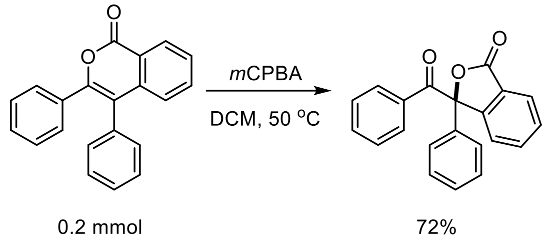


To a 5.0 mL screw-cap vial was added 3, 4-Diphenyl-1*H*-isochromen-1-one (59.6 mg, 0.2 mmol), *m*CPBA (121.8 mg, 0.6 mmol, 85% purity), and dichloromethane (3 mL) under air. After stirring at room temperature under air for 6 hours, *p*-toluenesulfonic acid (3.8 mg, 0.02 mmol) was added and the reaction was stirred at 50 °C overnight. The reaction was quenched with aqueous $Na_2S_2O_3$ at 0 °C and extracted with dichloromethane. The organic layer was washed with brine, dried over $Na_2SO_4$, and filtered through a sand core funnel. The solvent was removed under reduced pressure and the residue was separated on silica gel by PE/EA gradient (5:1) to get the target product as a white solid (45 mg, 72% yield).

$R_f$ 0.50 (Petroleum ether/EtOAc = 5/1).

**$^1$H NMR** (400 MHz, Chloroform-*d*) δ 8.02 – 7.98 (m, 2H), 7.87 (dd, *J* = 7.6 Hz, 2H), 7.70 (t, *J* = 7.6 Hz, 1H), 7.61 – 7.49 (m, 4H), 7.43 – 7.33 (m, 5H).

**$^{13}$C NMR** (101 MHz, Chloroform-*d*) δ 193.5, 169.4, 149.9, 137.6, 134.6, 134.2, 133.7, 131.0, 129.8, 129.6, 129.1, 128.5, 126.0, 125.6, 124.2, 123.8, 92.6.

**HRMS** (ESI) m/z: $[M + Na]^+$ Calcd for $C_{21}H_{14}O_3Na^+$ 337.0835; Found 337.0837.

## Oospolactone

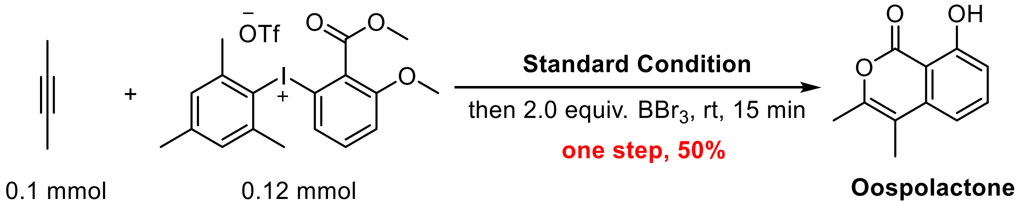


2-Butyne (37.6 mg, 7.8 uL, 0.1 mmol), mesityl(3-methoxy-2-(methoxycarbonyl) phenyl)iodonium trifluoromethanesulfonate (67.2 mg, 0.12 mmol), copper(I) chloride (1 mg, 0.01 mmol) and 5 Å MS (20 mg) were added to a dried vial sequentially. The reaction was stirred at 70 ºC after added anhydrous DCE (2 mL). The reaction mixture was stirred until complete consumption of starting material was observed by TLC. $BBr_3$ (0.2 mL, 1 M DCM solution) was added to the reaction mixture and stirred at room temperature for 15 minutes. Then the reaction was quenched with $H_2O$ and extracted with ethyl acetate (15 mL x 3). The organic layer was washed with brine, dried over $Na_2SO_4$, and filtered through a sand core funnel. Then the reaction mixture was concentrated in vacuum and the residue was separated on silica gel by PE/EA gradient (4:1) to get the target product as a white solid ( 9.5 mg, 50% yield).

$R_f$ 0.50 (Petroleum ether/EtOAc = 4/1).

**$^1$H NMR** (400 MHz, Chloroform-*d*) δ 11.31 (s, 1H), 7.62 (t, *J* = 8.0 Hz, 1H), 6.96 – 6.92 (m, 2H), 2.32 (d, *J* = 0.8 Hz, 3H), 2.14 (d, *J* = 1.2 Hz, 3H).

The titled compound is known. It's $^1$H-NMR is in accordance with the known literature.[11]

**Methyl3-(3,4-dimethoxyphenyl)-8-methoxy-1-*oxo*-1*H*-isochromene-4-carboxylate (3ar)**

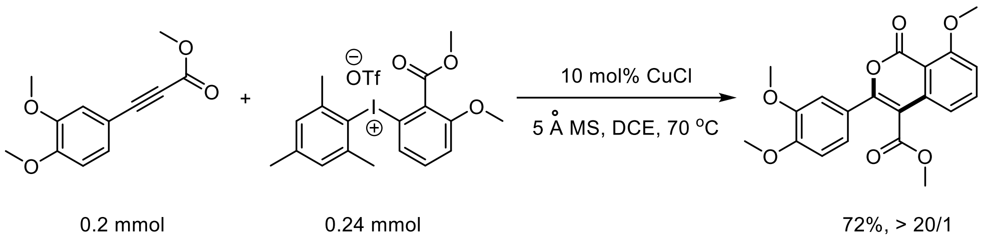


Methyl 3-(3,4-dimethoxyphenyl)propiolate (44.0 mg, 0.2 mmol), mesityl(3-methoxy-2-(methoxycarbonyl)phenyl)iodonium trifluoromethanesulfonate (134.4 mg, 0.24 mmol), copper(I) chloride (2 mg, 0.02 mmol) and 5 Å MS (20 mg) were added to a dried vial sequentially. The reaction was stirred at 70 °C after added anhydrous DCE (4 mL). The reaction mixture was stirred until complete consumption of starting material was observed by TLC. Then the reaction mixture was concentrated in vacuum and separated on silica gel by PE/EA gradient (1:1) to get the target product as a white solid (53 mg, 72% yield, regioselectivity > 20/1). (Regioselectivity was determined by crude sample NMR.)

$R_f$ 0.23 (Petroleum ether/EtOAc = 1/1).

**$^1$H NMR** (400 MHz, Chloroform-*d*) δ 7.67 (t, *J* = 8.4 Hz, 1H), 7.24 – 7.14 (m, 3H), 7.01 (d, *J* = 7.6 Hz, 1H), 6.90 (d, *J* = 8.4 Hz, 1H), 4.03 (s, 3H), 3.93 (s, 6H), 3.75 (s, 3H).

**$^{13}$C NMR** (101 MHz, Chloroform-*d*) δ 167.8, 162.0, 157.8, 155.1, 151.2, 149.0, 137.7, 136.4, 124.9, 121.5, 115.7, 110.8, 110.7, 110.6, 109.8, 108.4, 56.6, 56.2, 56.1, 52.9.

**HRMS** (ESI) m/z: $[M + H]^+$ Calcd for $C_{20}H_{19}O_7^+$ 371.1125; Found 371.1121.

**Thunberginol A**

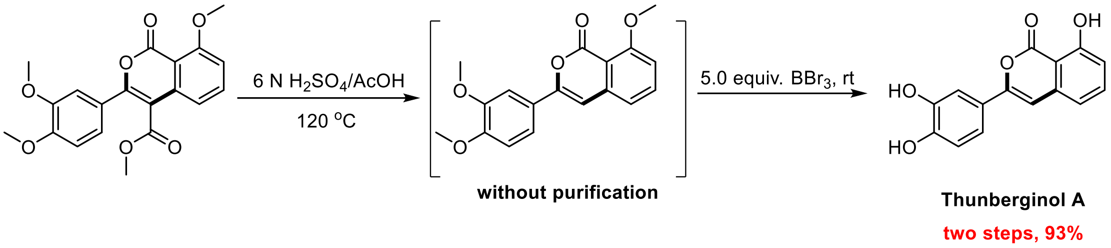


A mixture of 3-(3,4-dimethoxyphenyl)-8-methoxy-1-*oxo*-1*H*-isochromene-4-carboxylate (23 mg, 0.06 mmol) , 0.5 mL of glacial acetic acid and 0.5 mL of 6 N sulfuric acid were taken in a 5.0 mL screw-cap vial. The reaction mixture was heated to 120 °C for 4 h. The reaction mixture was cooled, diluted with water and extracted with ethyl acetate (8 mL*3). The organic layer was washed with brine, dried over $Na_2SO_4$, and filtered through a sand core funnel. The extracting mixture was concentrated under vacuum and used to the next step without further purification.

The above mixture and anhydrous DCM (1 mL) was added a dried vial. Then $BBr_3$ (0.3 mL, 1 M DCM solution) was added to the reaction mixture and stirred at room temperature until complete consumption of starting material was observed by TLC. Then the reaction was quenched with $H_2O$ and extracted with ethyl acetate (10 mL * 5). The organic layer was washed with brine, dried over $Na_2SO_4$, and filtered through a sand core funnel. Then the reaction mixture was concentrated in vacuum and the residue was separated on silica gel by PE/EA gradient (2:1) to get the target product as a brown solid ( 15.0 mg, 93% yield).

$R_f$ 0.25 (Petroleum ether/EtOAc = 1/1).

**$^1$H NMR** (400 MHz, DMSO-$d_6$) δ 7.67 (t, *J* = 8.0 Hz, 1H), 7.35 – 7.22 (m, 3H), 7.08 (d, *J* = 7.6 Hz, 1H), 6.92 (d, *J* = 8.0 Hz, 1H), 6.87 (d, *J* = 8.0 Hz, 1H).

The titled compound is known. It's $^1$H-NMR is in accordance with the known literature.[12]

# References

1. Kenji Ueura, Tetsuya Satoh, Masahiro Miura, An Efficient Waste-Free Oxidative Coupling via Regioselective C–H Bond Cleavage: Rh/Cu-Catalyzed Reaction of Benzoic Acids with Alkynes and Acrylates under Air. *Org. Lett.* **2007**, *9,* 1407 – 1409.

2. Jiayu Mo, Lianhui Wang, Xiuling Cui, Rhodium(III)-Catalyzed C–H Activation/Alkyne Annulation by Weak Coordination of Peresters with O–O Bond as an Internal Oxidant. *Org. Lett.* **2015**, *17,* 4960 – 4963.

3. Venkataraman Subramanian, Venkateswara Rao Batchu, Deepak Barange, Manojit Pal, Synthesis of Isocoumarins via Pd/C mediated Reaction of *o*-Iodobenzoic acid with Terminal alkynes. *J. Org. Chem.* **2005**, *70,* 4778 – 4783.

4. Wei Li, Mario P. Wiesenfeldt, and Frank Glorius, Ruthenium–NHC–Diamine Catalyzed Enantioselective Hydrogenation of Isocoumarins. *J. Am. Chem. Soc.* **2017**, *139,* 2585 – 2588.

5. Jong Hyub Park, Sachin V. Bhilare, So Won Youn, NHC-Catalyzed Oxidative Cyclization Reactions of 2-Alkynylbenzaldehydes under Aerobic Conditions: Synthesis of O-Heterocycles. *Org. Lett.* **2011**, *13,* 2228 – 2231.

6. Hong-Yueh Liao, Chien-Hong Cheng, Synthesis of Isocoumarins from o-Iodobenzoic Acid and Terminal Acetylenes Mediated by Palladium Complexes and Zinc Chloride. *J. Org. Chem.* **1995**, *60,* 3711 – 3716.

7. Qi-Liang Yang, Yi-Kang Xing, Xiang-Yang Wang, Hong-Xing Ma, Xin-Jun Weng, Xiang Yang, Hai-Ming Guo, Tian-Sheng Mei, Electrochemistry-Enabled Ir-Catalyzed Vinylic C−H Functionalization. *J. Am. Chem. Soc.* **2019**, *141*, 18970–18976.

8. Ting Han, Haiqin Deng, Chris Y. Y. Yu, Chen Gui, Zhegang Song,Ryan T. K. Kwok, Jacky W. Y. Lam, Ben Zhong Tang, Functional isocoumarin-containing polymerssynthesized by rhodium-catalyzed oxidativepolycoupling of aryl diacid and internal diyne. *Polym. Chem*. **2016**, *7*, 2501 – 2510.

9. Rashmi Prakash, Kommuri Shekarrao, Sanjib Gogoi, Romesh C. Boruah. Ruthenium-catalyzed decarbonylative addition reaction of anhydrides with alkynes: a facile synthesis of isocoumarins and *α*-pyrones. *Chem. Commun.*, **2015**, *51*, 9972 – 9974.

10. Guangbin Jiang, Jianxiao Li, Chuanle Zhu, Wanqing Wu, Huanfeng Jiang, Palladium-Catalyzed Sequential Nucleophilic Addition/Oxidative Annulation of Bromoalkynes with Benzoic Acids To Construct Functionalized Isocoumarins. *Org. Lett.* **2017**, *19*, 4440 – 4443.

11. George Majetich, Jeremy L. Grove, Synthesis of 8-hydroxyisochromenes and 8-hydrooxy isocoumarins from 3-ethoxycyclohex-2-en-1-one. *Heterocycles* **2012**, *84*, 983 – 1012.

12. Ai Kurume, Yasuhiro Kamata, Masayuki Yamashita, Qilong Wang, Hisashi Matsuda, Masayuki Yoshikawa, Ikuo Kawasaki, Shunsaku Ohta, Synthesis of 3-Substituted Isocoumarins and Their Inhibitory Effects on Degranulation of RBL-2H3 Cells Induced by Antigen, *Chem. Pharm. Bull.* **2008**, *56*, 1264 – 1269.
