# Supplementary material for: Design, development and applications of copper-catalyzed regioselective (4 + 2) annulations between diaryliodonium salts and alkynes
Source: Commun Chem. 2022 Nov 8;5:145. doi: 10.1038/s42004-022-00768-3 (PMC9814649; doi:10.1038/s42004-022-00768-3)
Supplement: Supplementary file 2 — Description of Additional Supplementary Files [file 42004_2022_768_MOESM2_ESM.docx]

Description of Additional Supplementary Files

**File name:** Supplementary Data 1

**Description**: Spectra of substrates and products
