# Supplementary material for: Design, development and applications of copper-catalyzed regioselective (4 + 2) annulations between diaryliodonium salts and alkynes
Source: Commun Chem. 2022 Nov 8;5:145. doi: 10.1038/s42004-022-00768-3 (PMC9814649; doi:10.1038/s42004-022-00768-3)

Spectra of substrates and products

^{1}H NMR of **2aa** ($CDCl_3$, 400 M)

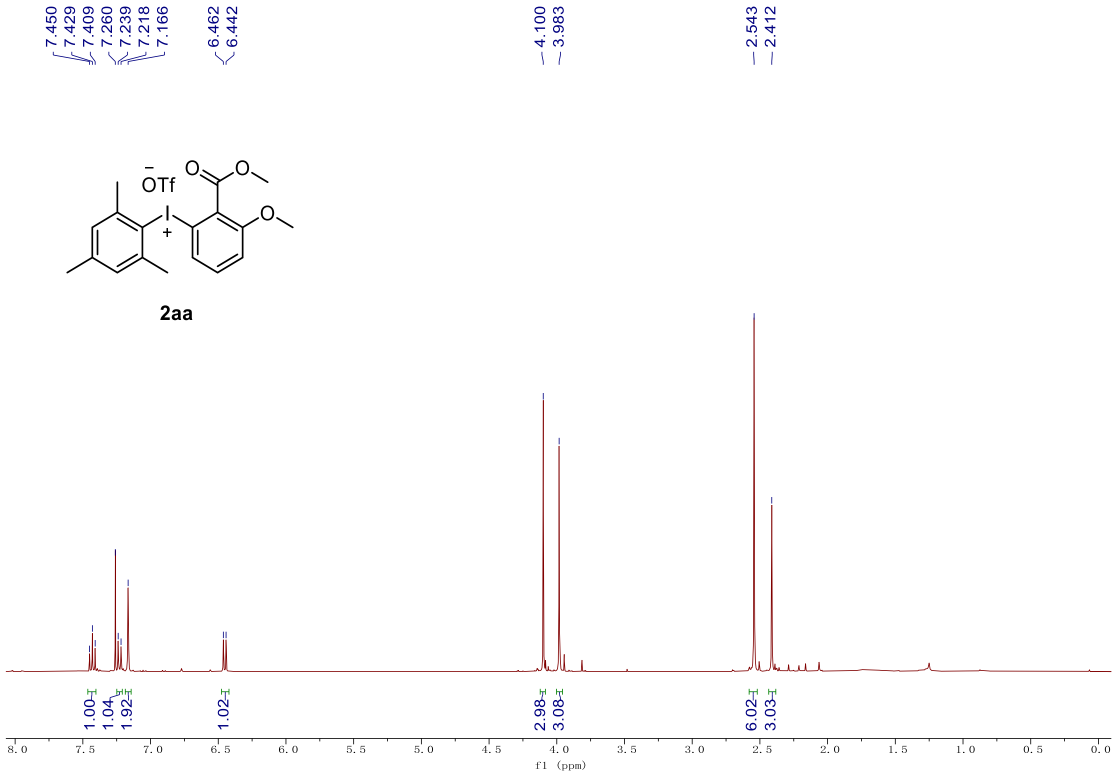

^{13}C NMR of **2aa** ($CDCl_3$, 101 M)

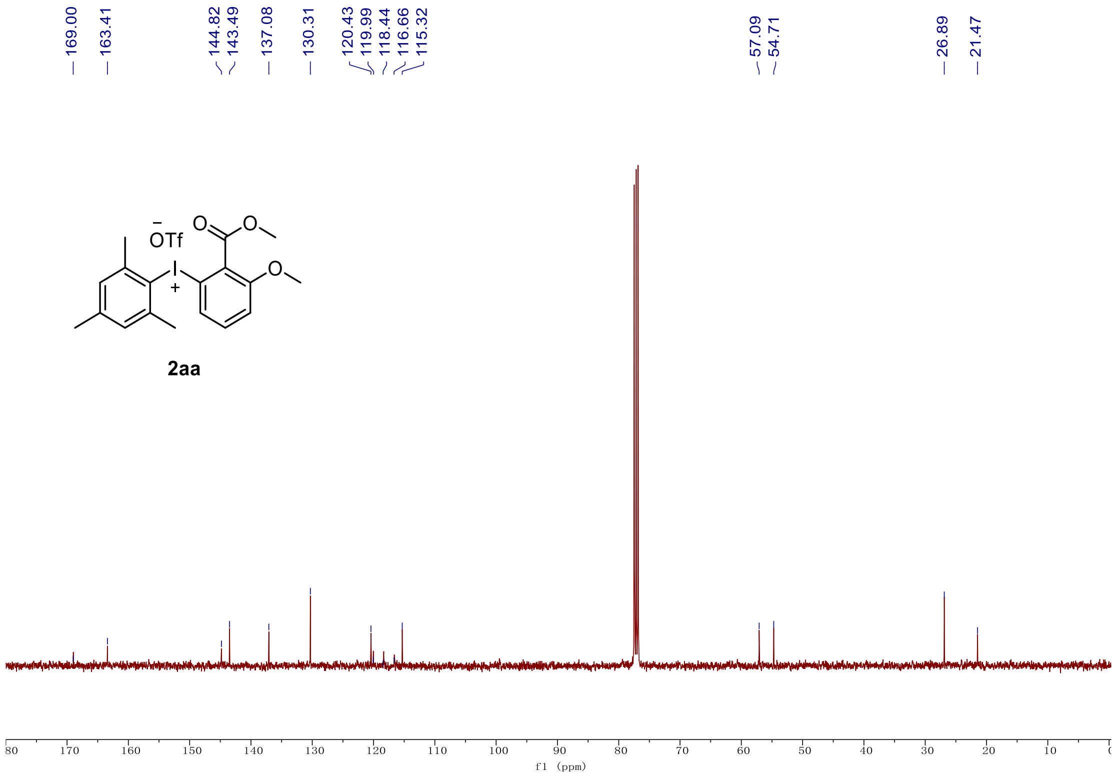

^{19}F NMR of **2aa** ($CDCl_3$, 376 M)

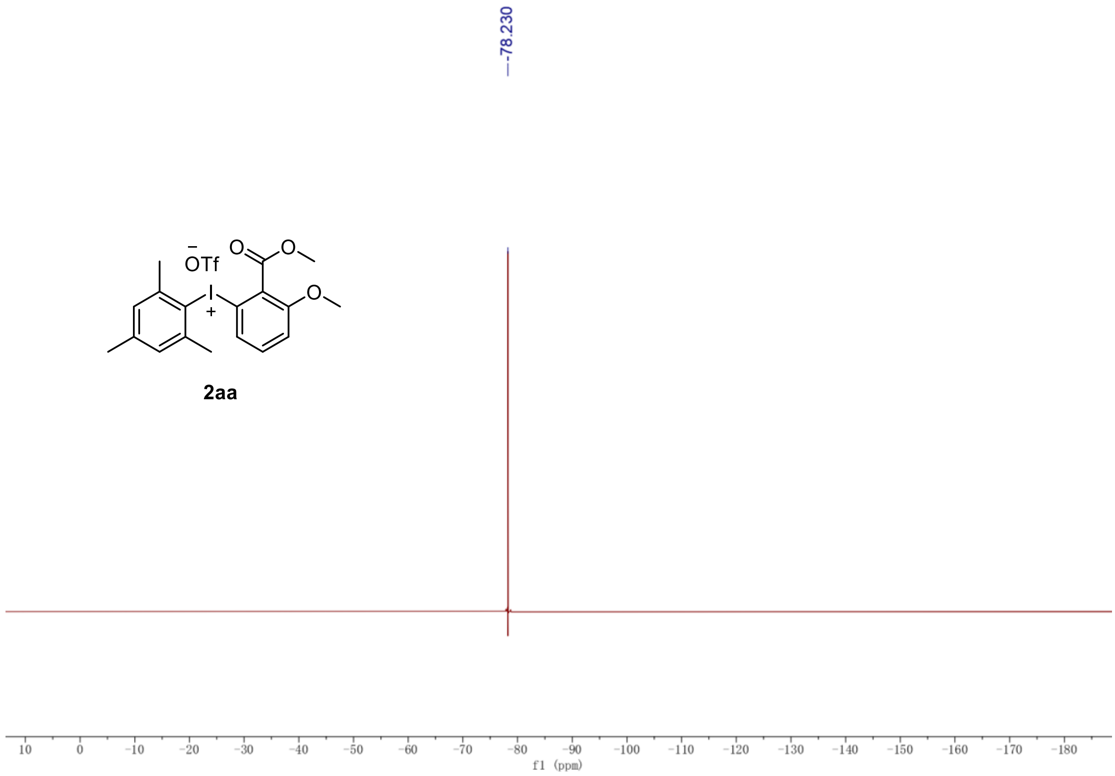

^{1}H NMR of **2ab** ($CDCl_3$, 400 M)

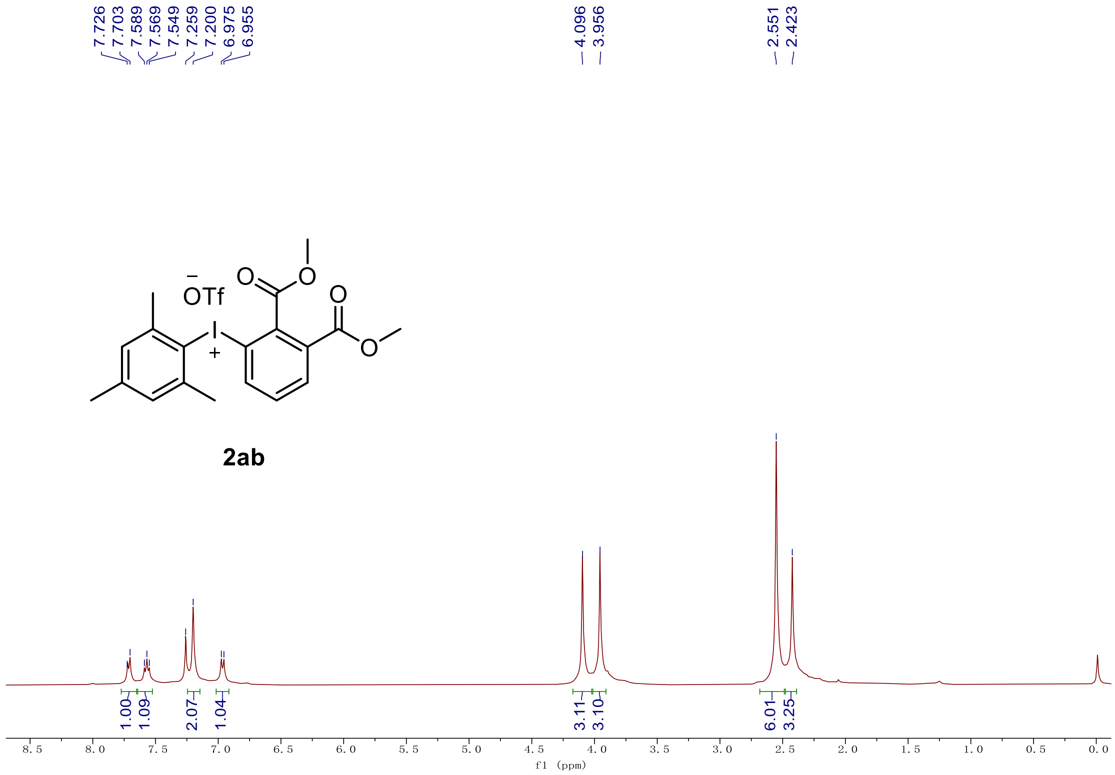

^{13}C NMR of **2ab** ($CDCl_3$, 101 M)

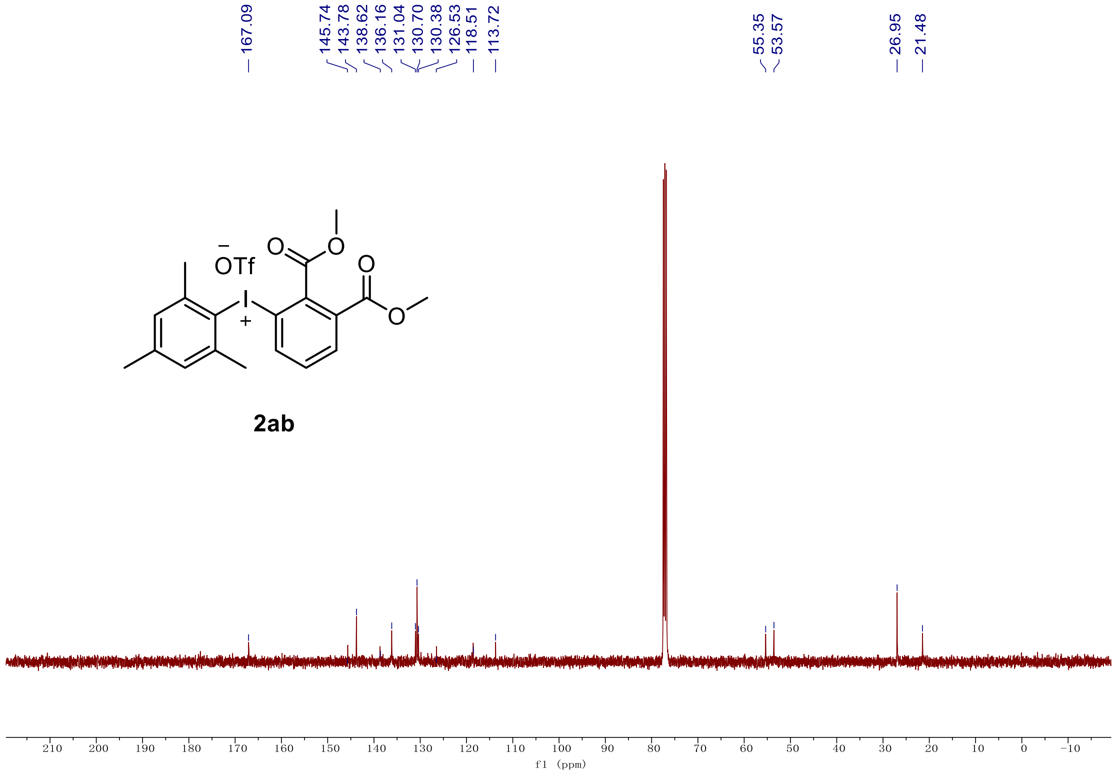

^{19}F NMR of **2ab** ($CDCl_3$, 376 M)

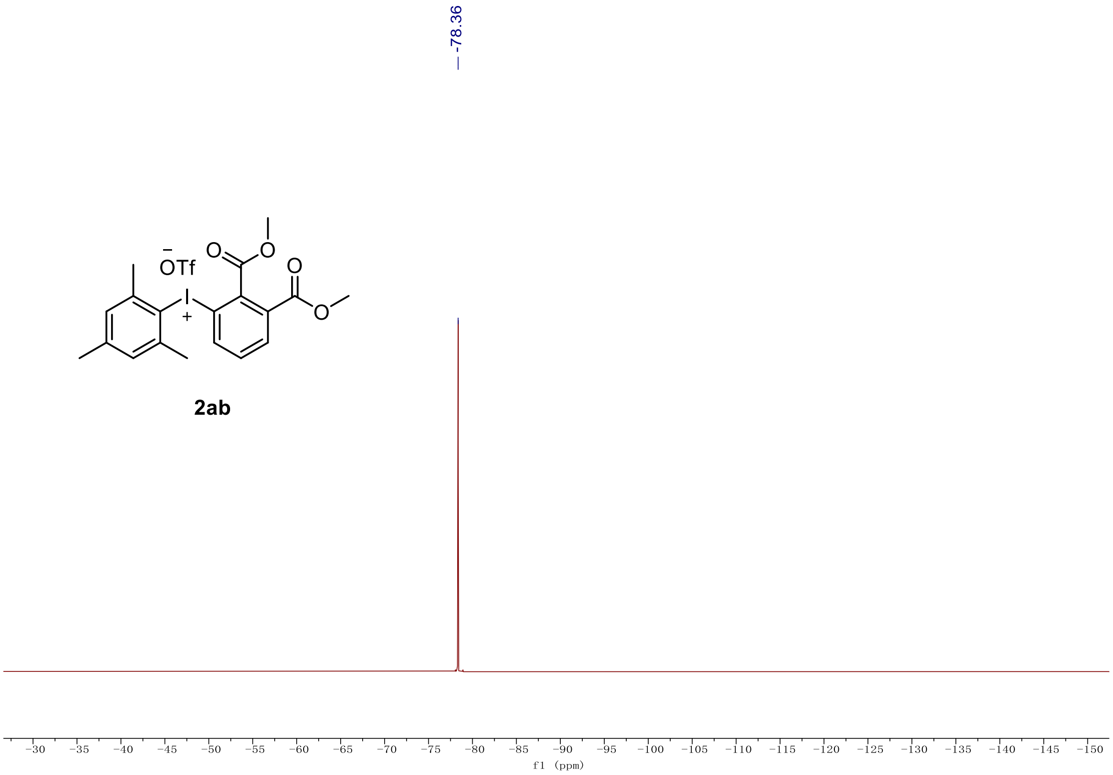

^{1}H NMR of **2ac** ($CDCl_3$, 400 M)

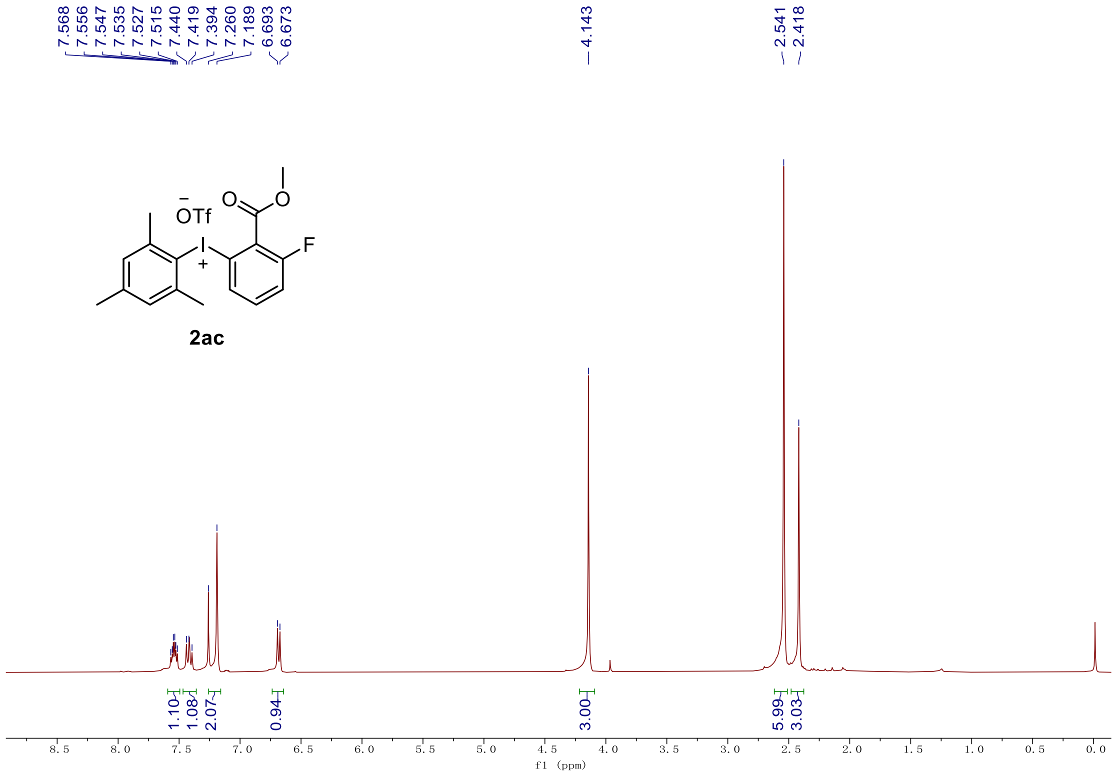

^{13}C NMR of **2ac** ($CDCl_3$, 101 M)

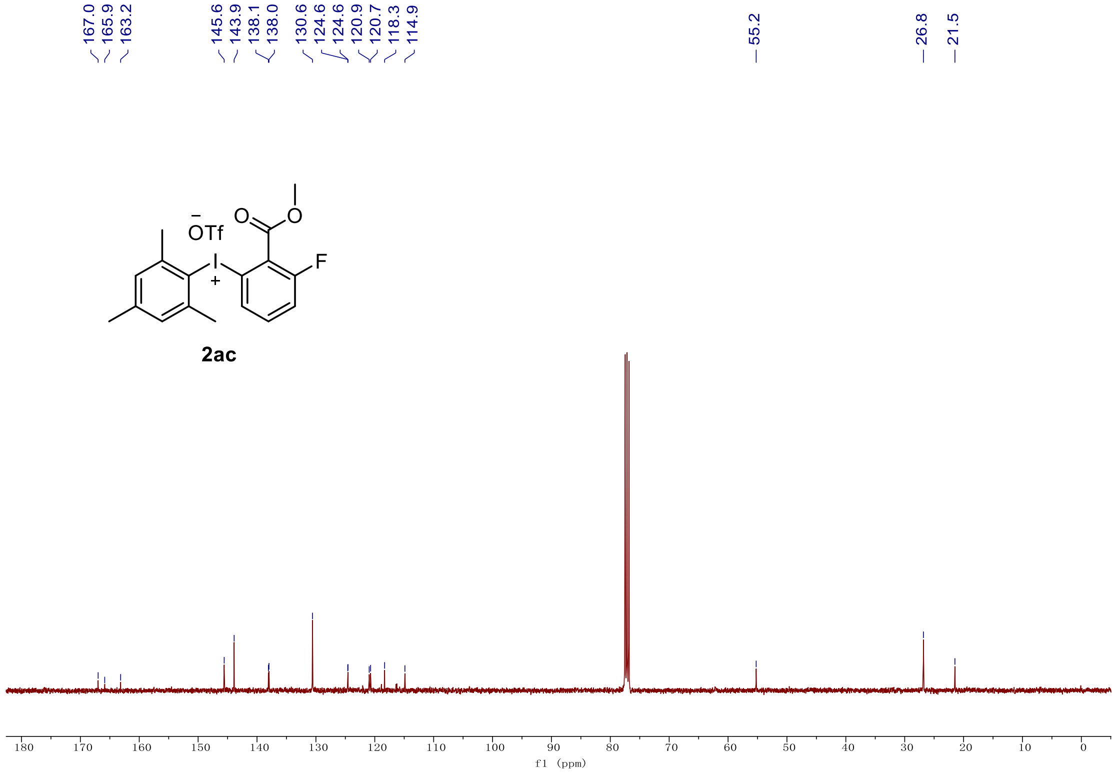

^{19}F NMR of **2ac** ($CDCl_3$, 376 M)

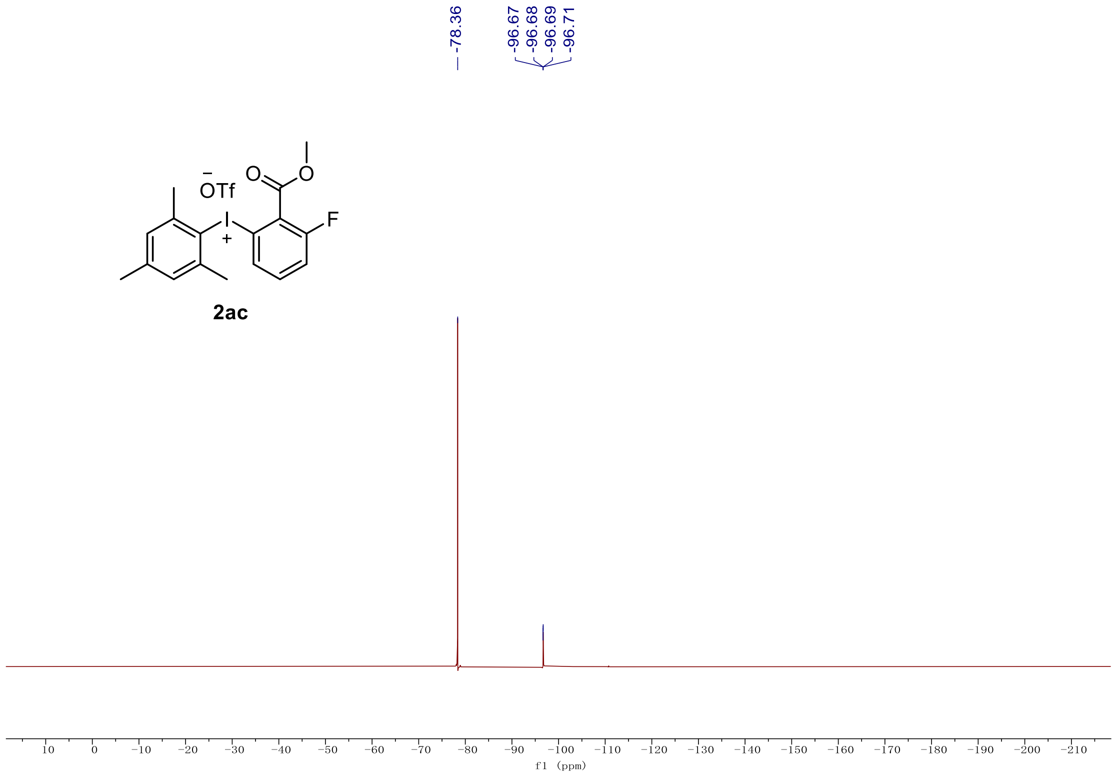

^{1}H NMR of **2ad** ($CDCl_3$, 400 M)

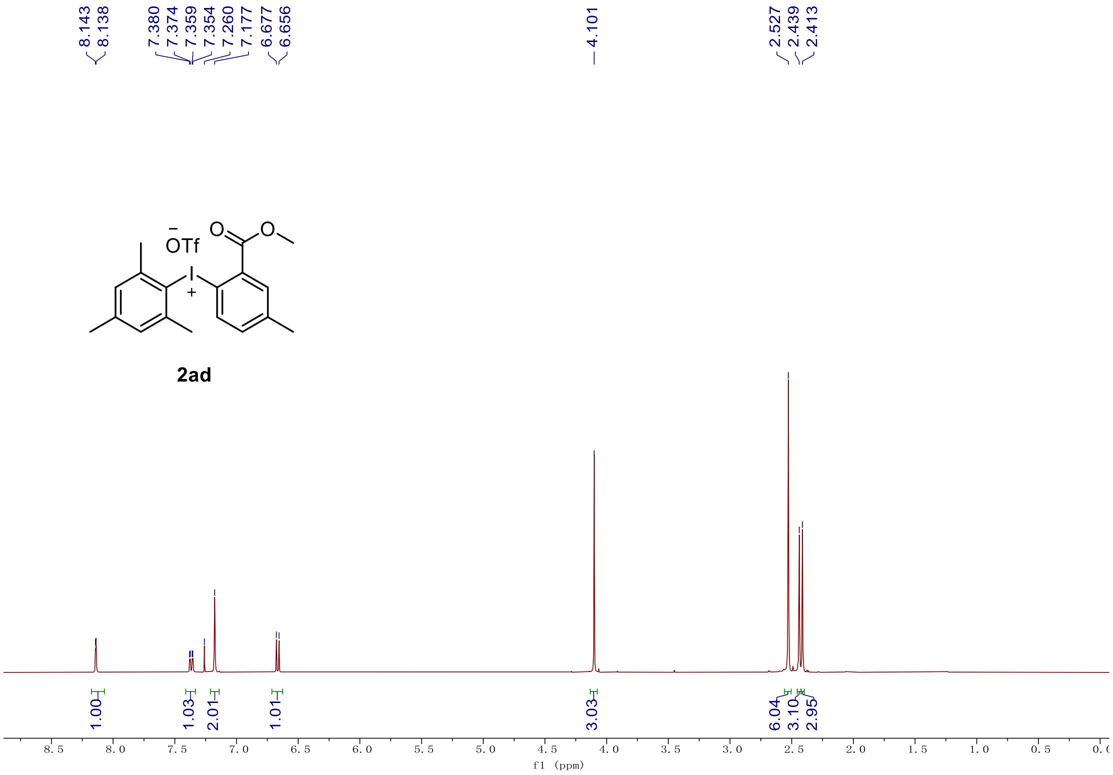

^{13}C NMR of **2ad** ($CDCl_3$, 101 M)

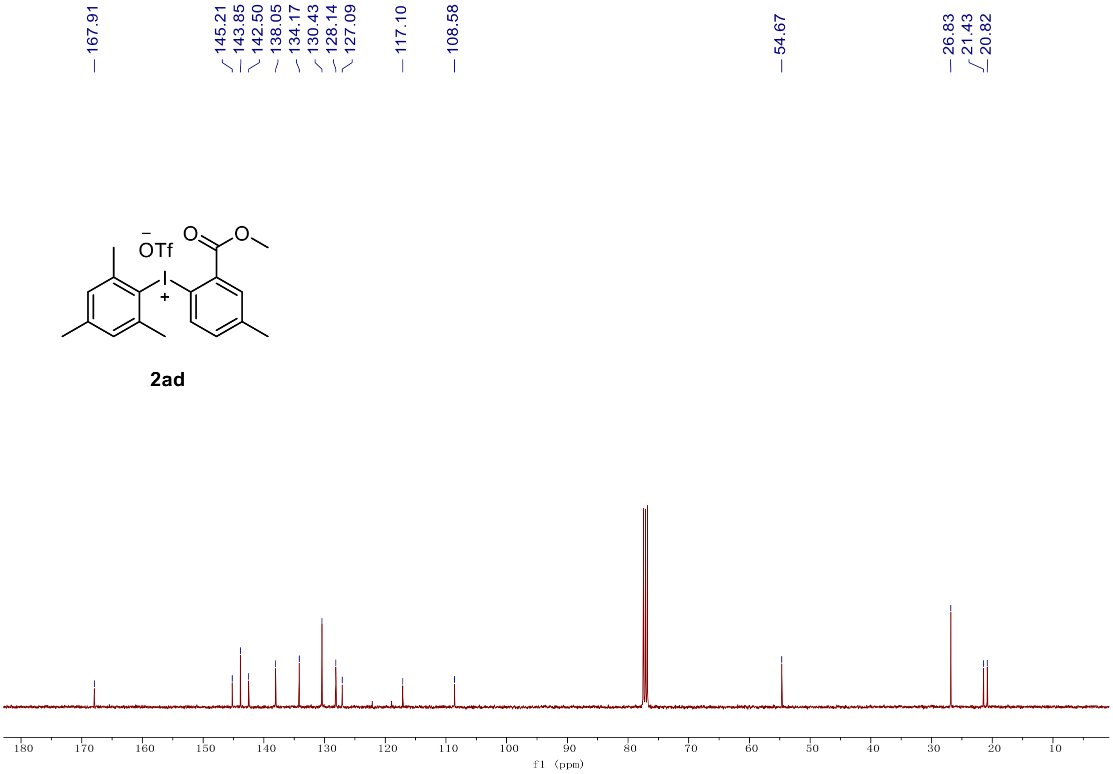

^{19}F NMR of **2ad** ($CDCl_3$, 376 M)

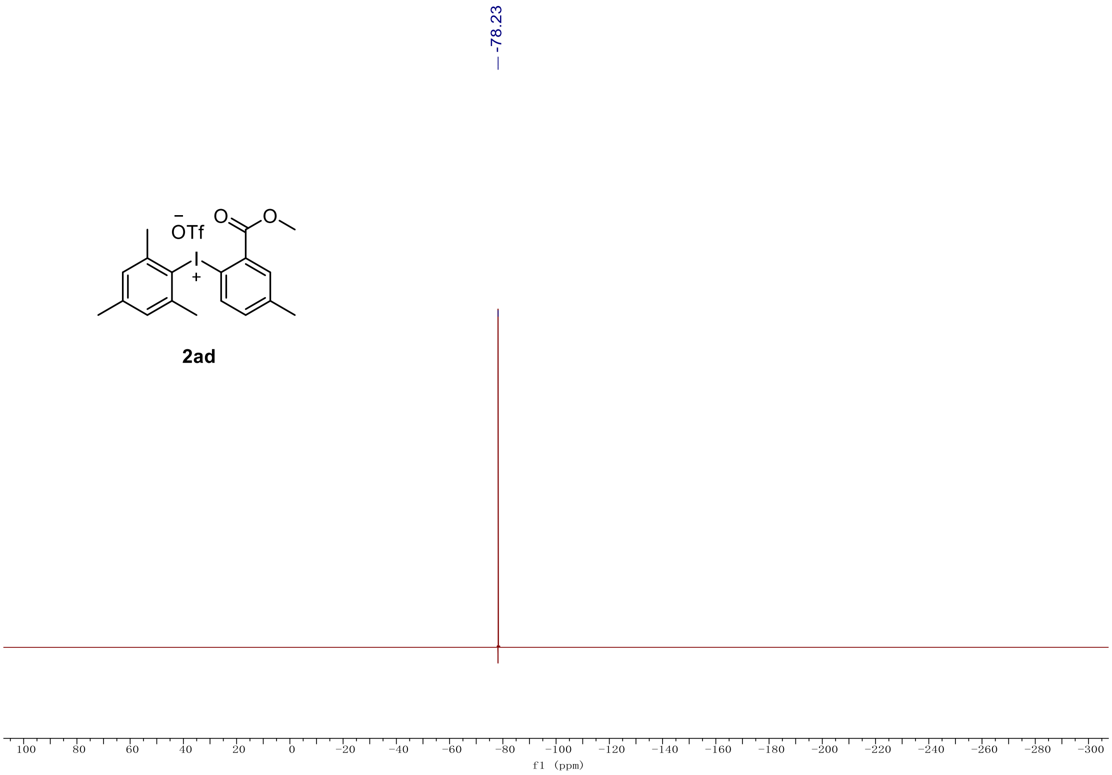

^{1}H NMR of **2ae** ($CDCl_3$, 400 M)

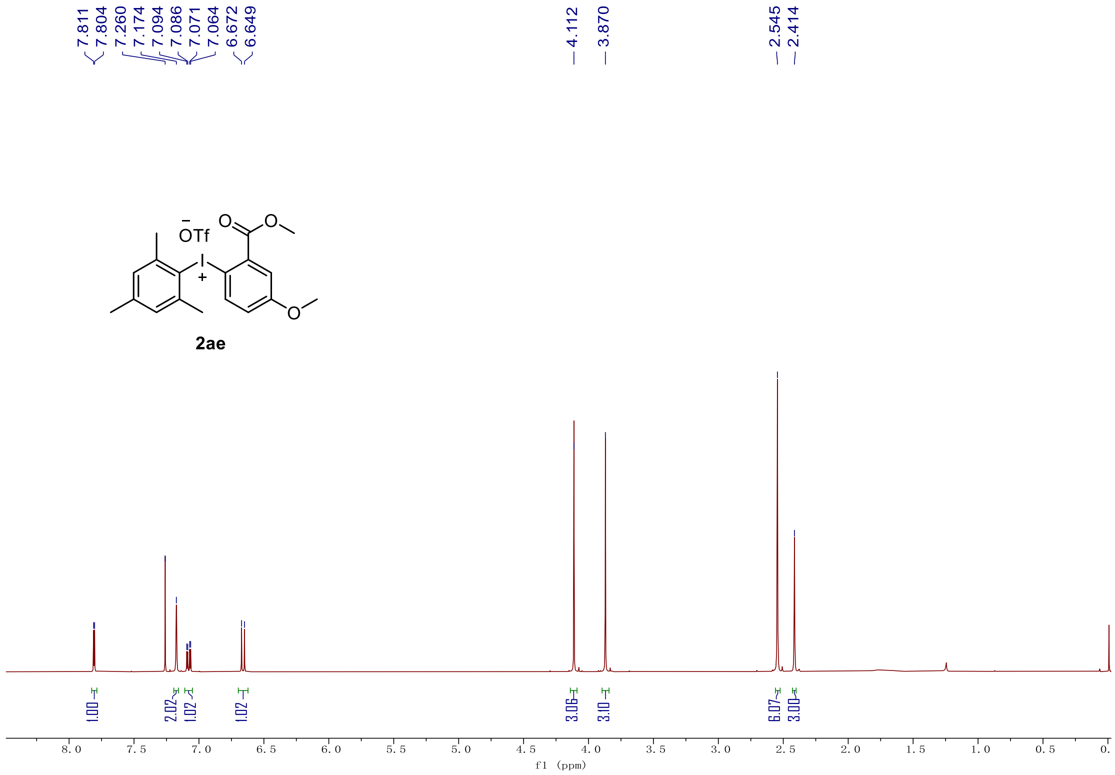

^{13}C NMR of **2ae** ($CDCl_3$, 101 M)

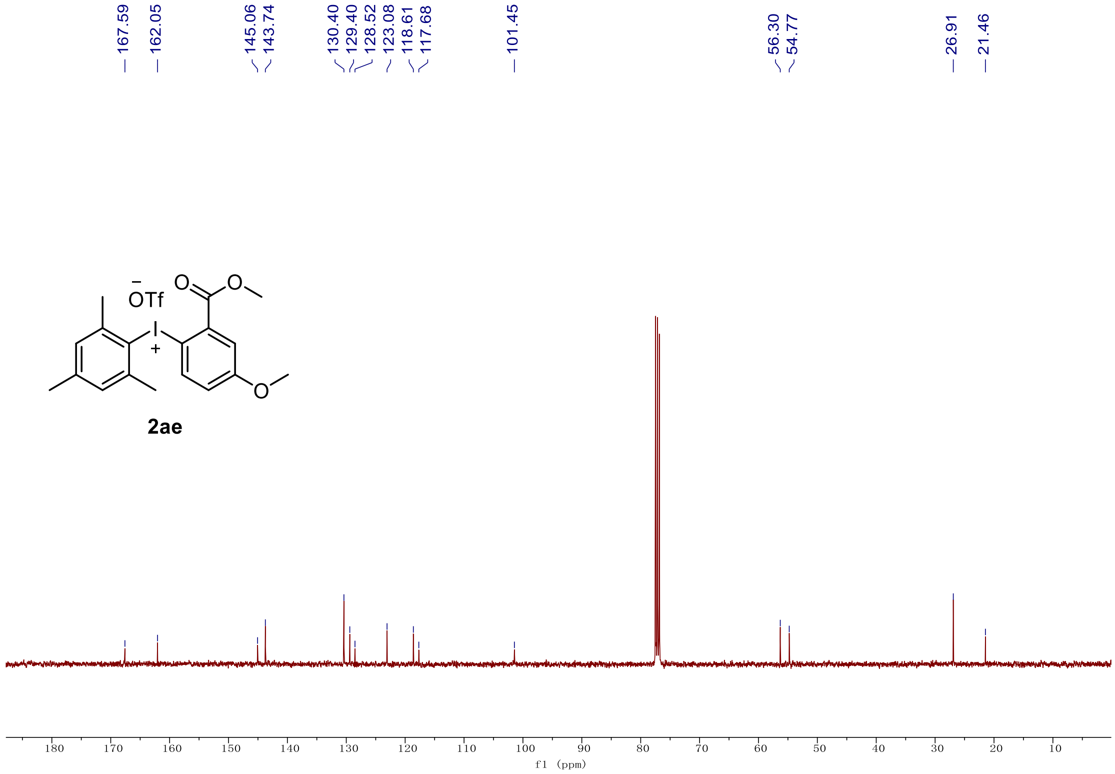

^{19}F NMR of **2ae** ($CDCl_3$, 376 M)

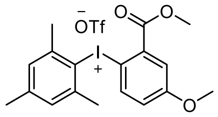

2ae

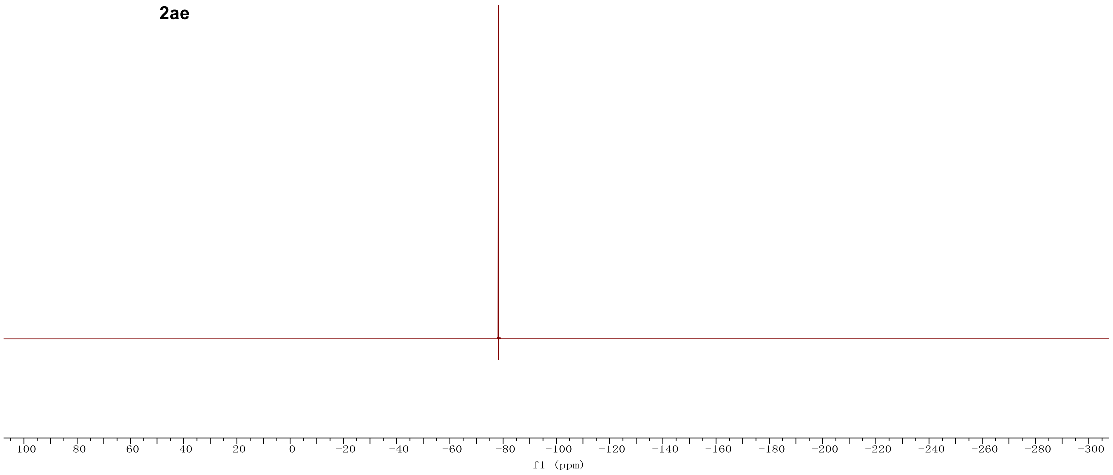

^{1}H NMR of **2af** ($CDCl_3$, 400 M)

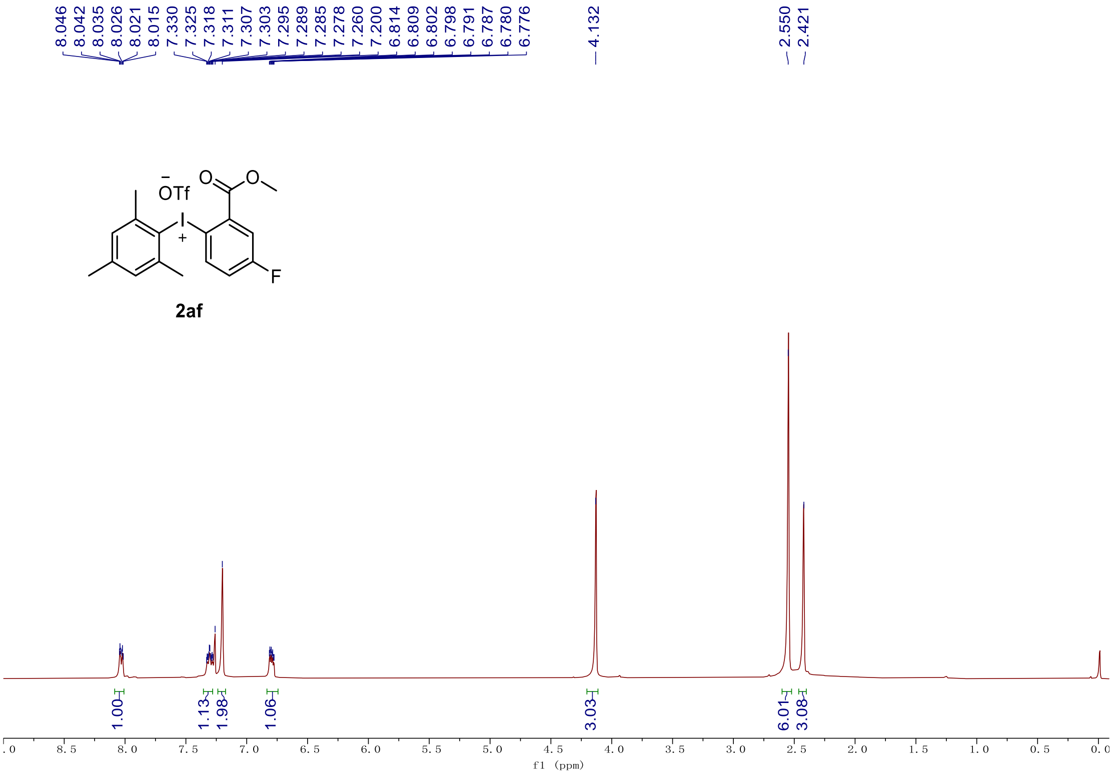

^{13}C NMR of **2af** ($CDCl_3$, 101 M)

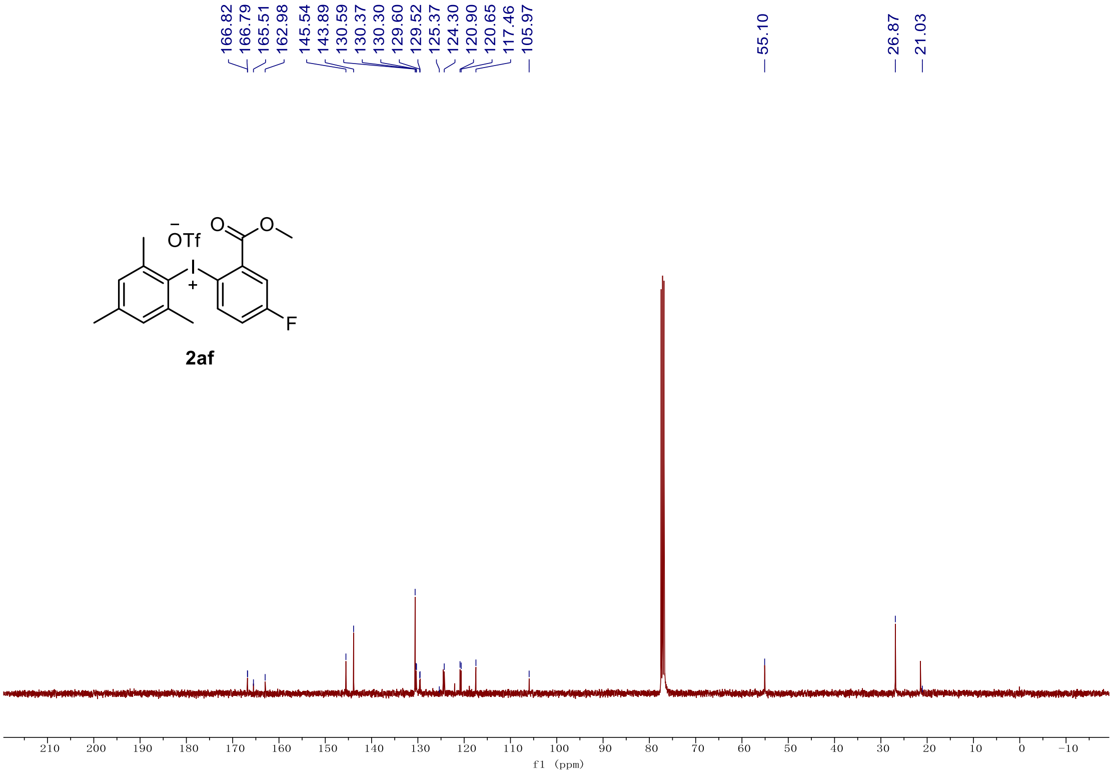

^{19}F NMR of **2af** ($CDCl_3$, 376 M)

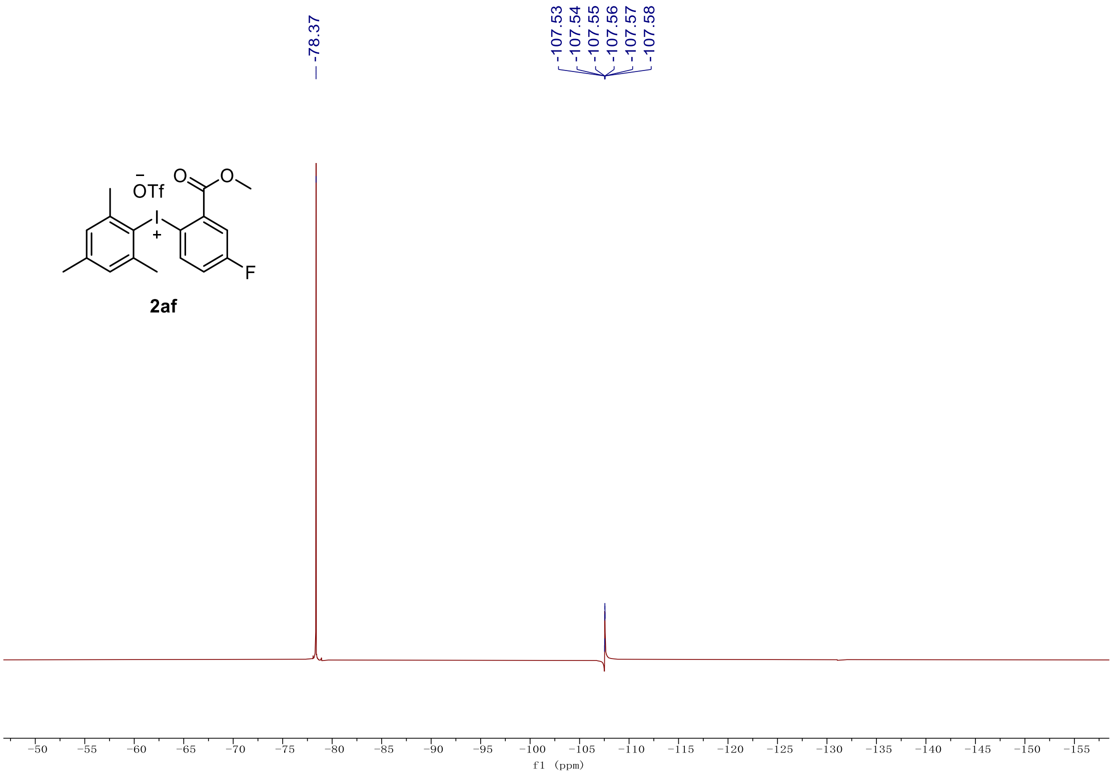

^{1}H NMR of **2ag** ($CDCl_3$, 400 M)

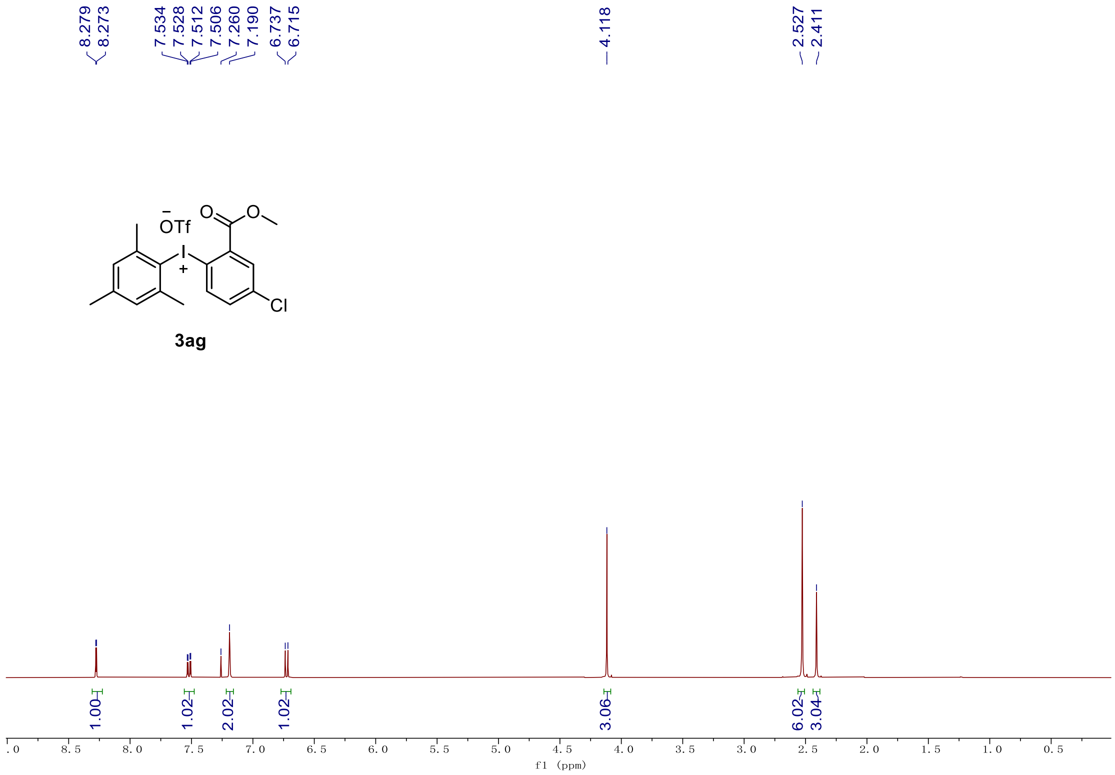

^{13}C NMR of **2ag** ($CDCl_3$, 101 M)

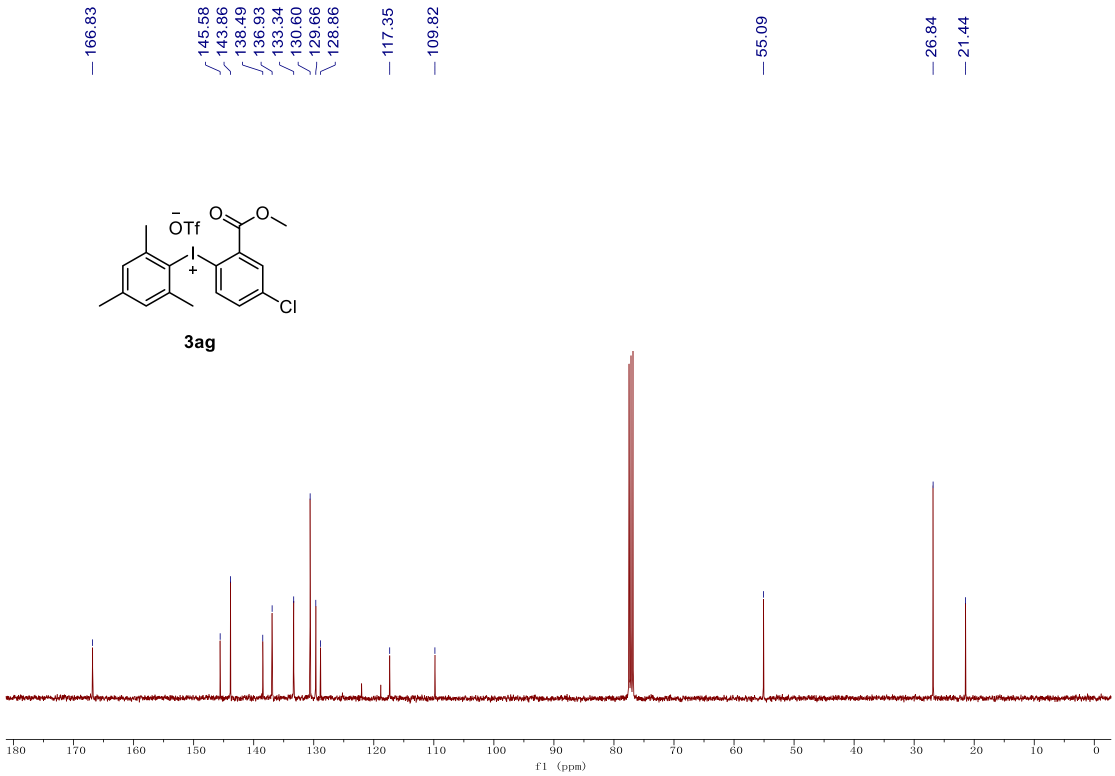

^{19}F NMR of **2ag** ($CDCl_3$, 376 M)

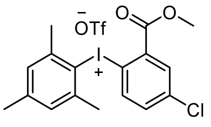

3ag

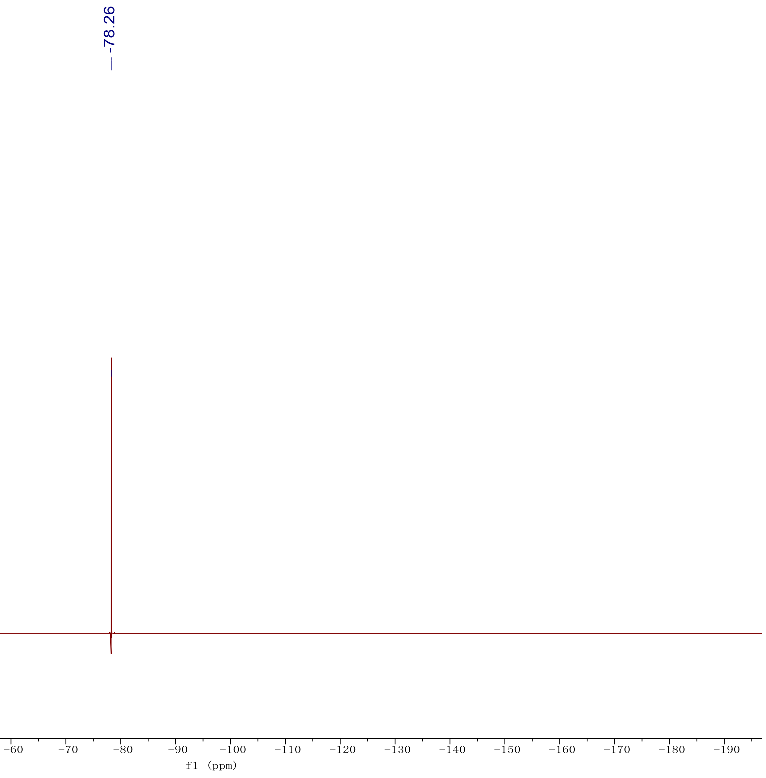

^{1}H NMR of **2ah** ($CDCl_3$, 400 M)

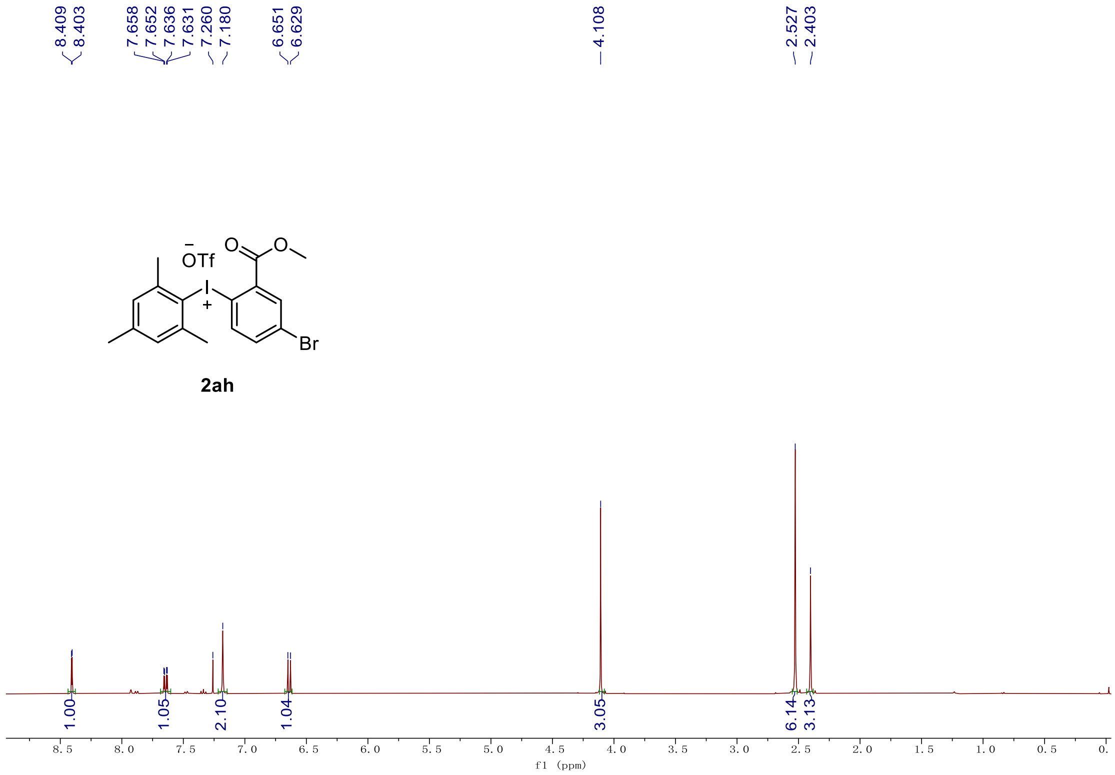

^{13}C NMR of **2ah** ($CDCl_3$, 101 M)

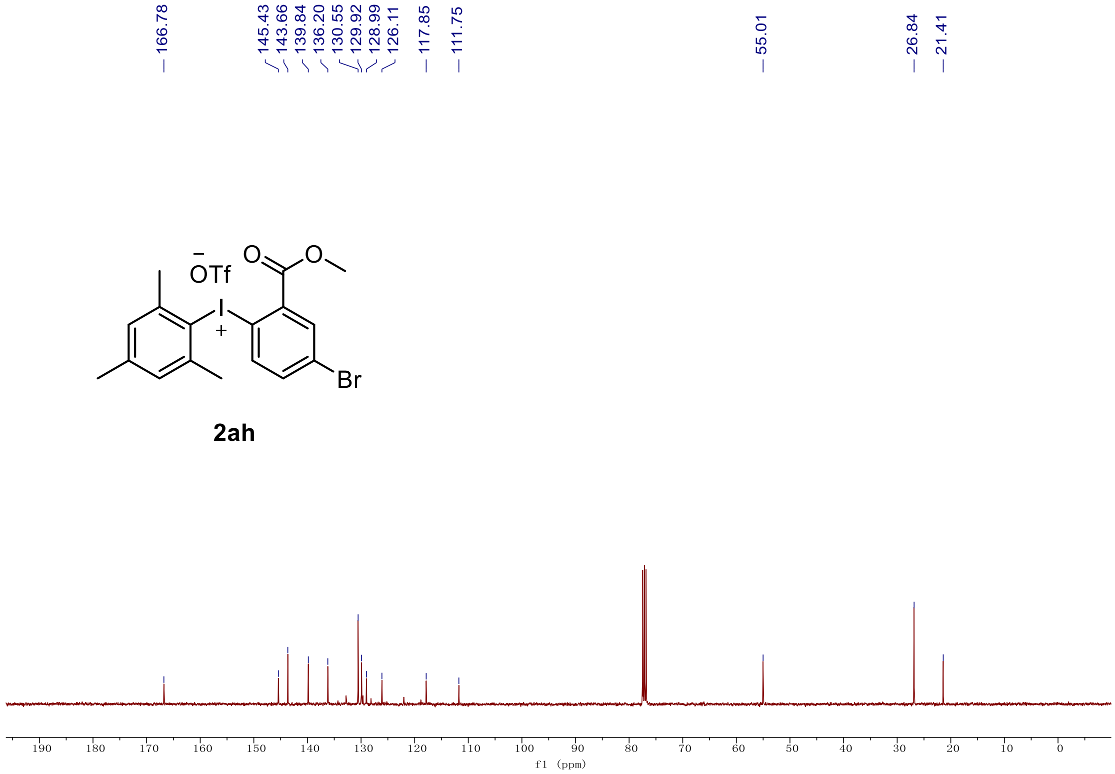

^{19}F NMR of **2ah** ($CDCl_3$, 376 M)

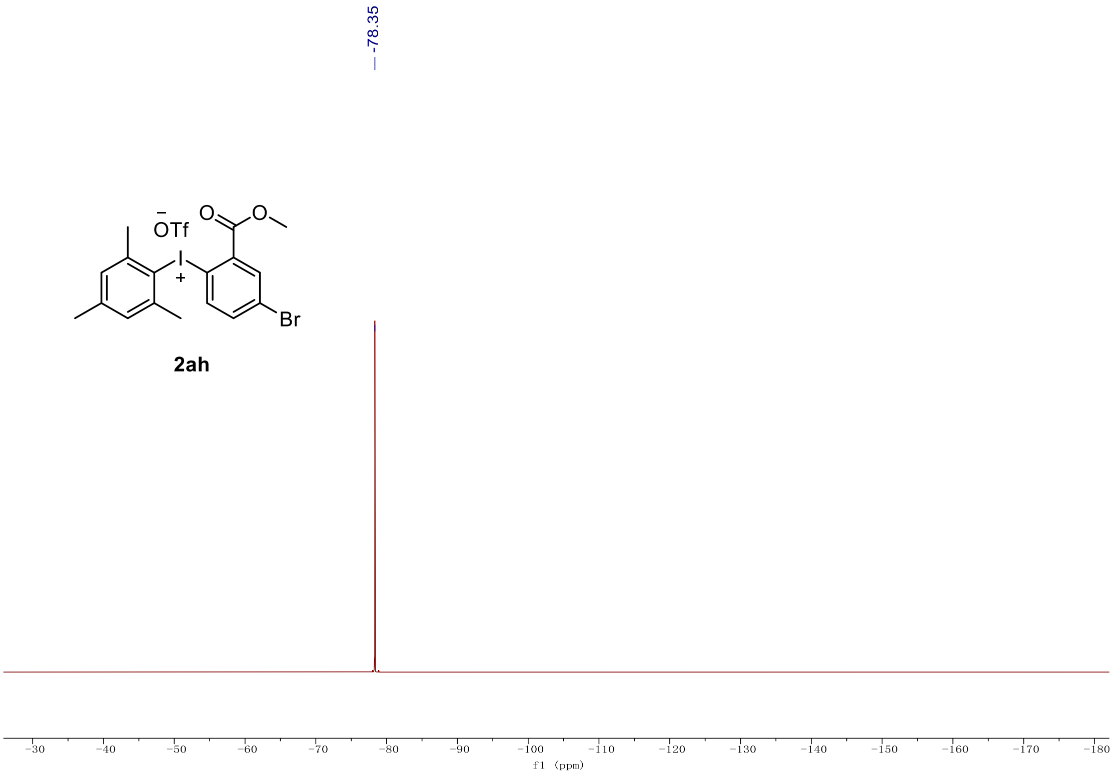

^{1}H NMR of **2ai** ($CDCl_3$, 400 M)

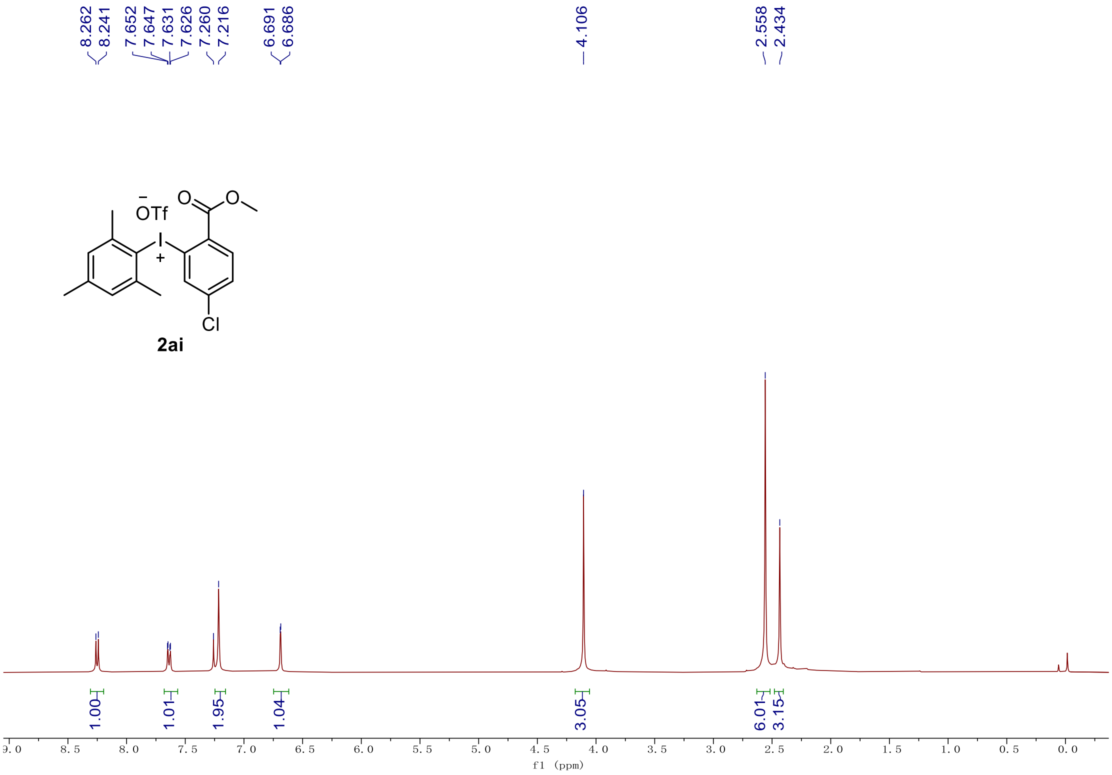

^{13}C NMR of **2ai** ($CDCl_3$, 101 M)

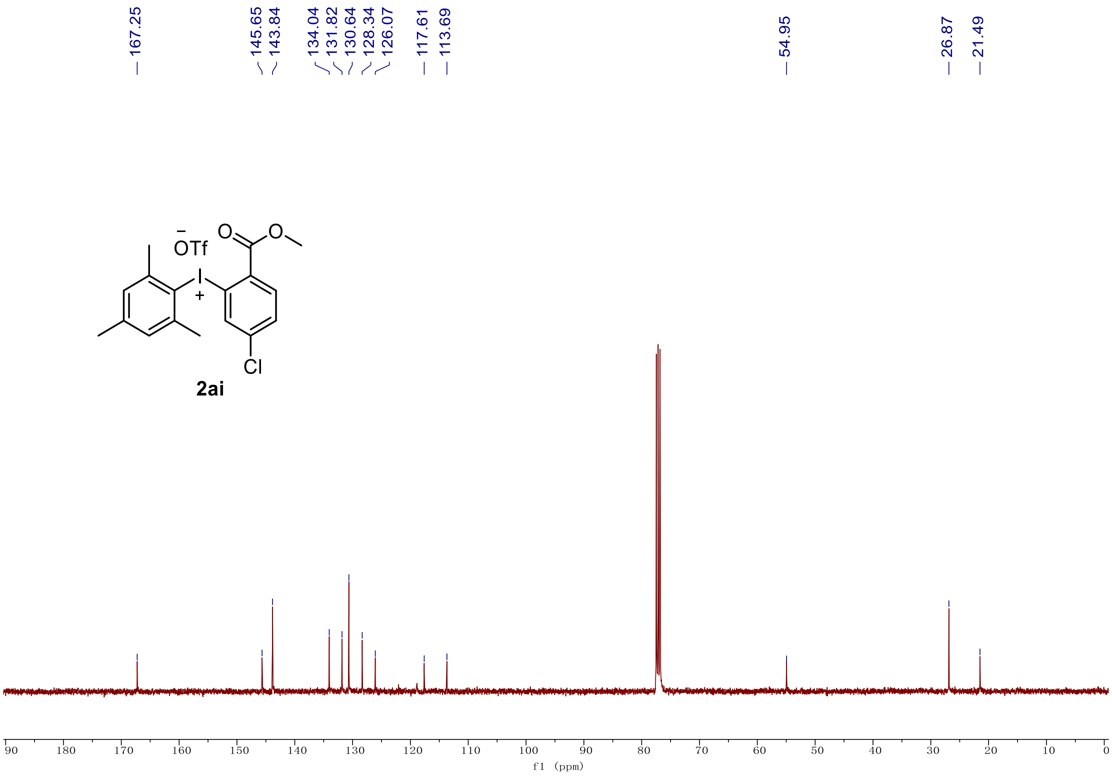

^{19}F NMR of **2ai** ($CDCl_3$, 376 M)

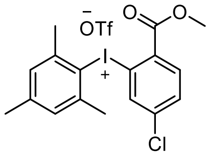

2ai

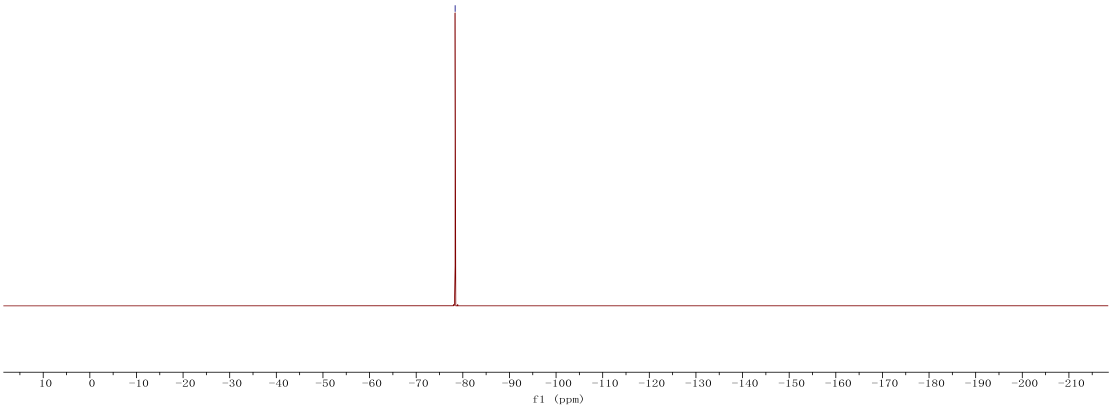

^{1}H NMR of **2aj** ($CDCl_3$, 400 M)

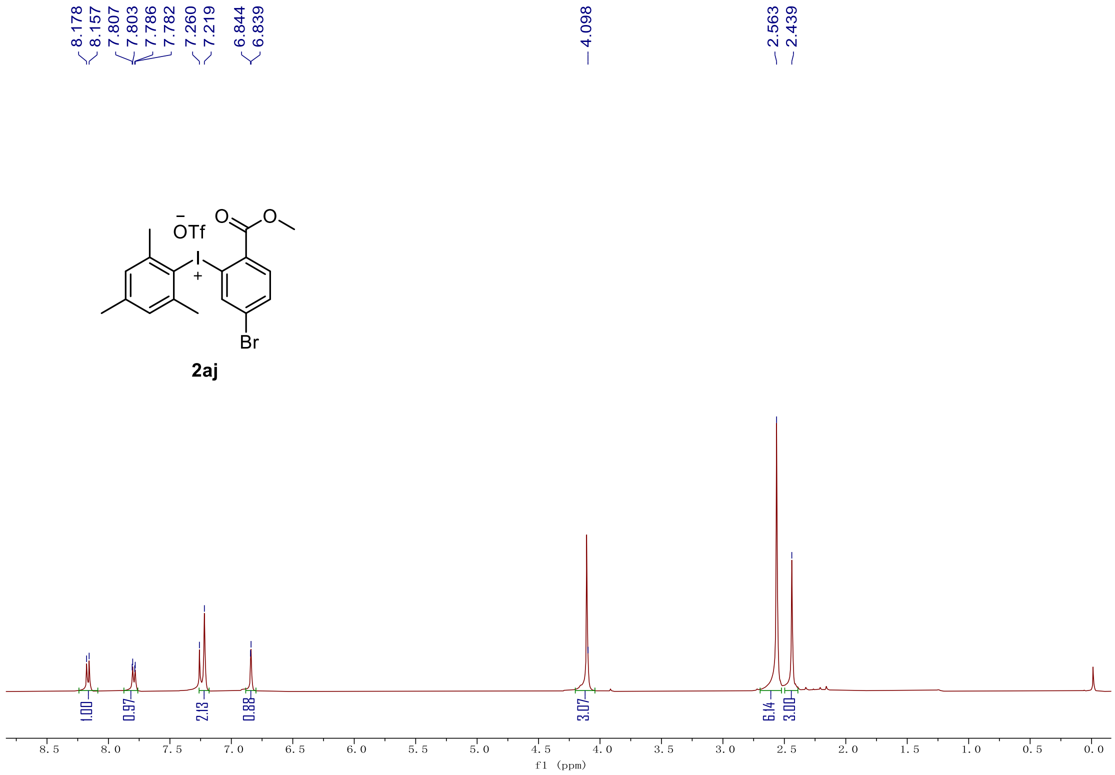

^{13}C NMR of **2aj** ($CDCl_3$, 101 M)

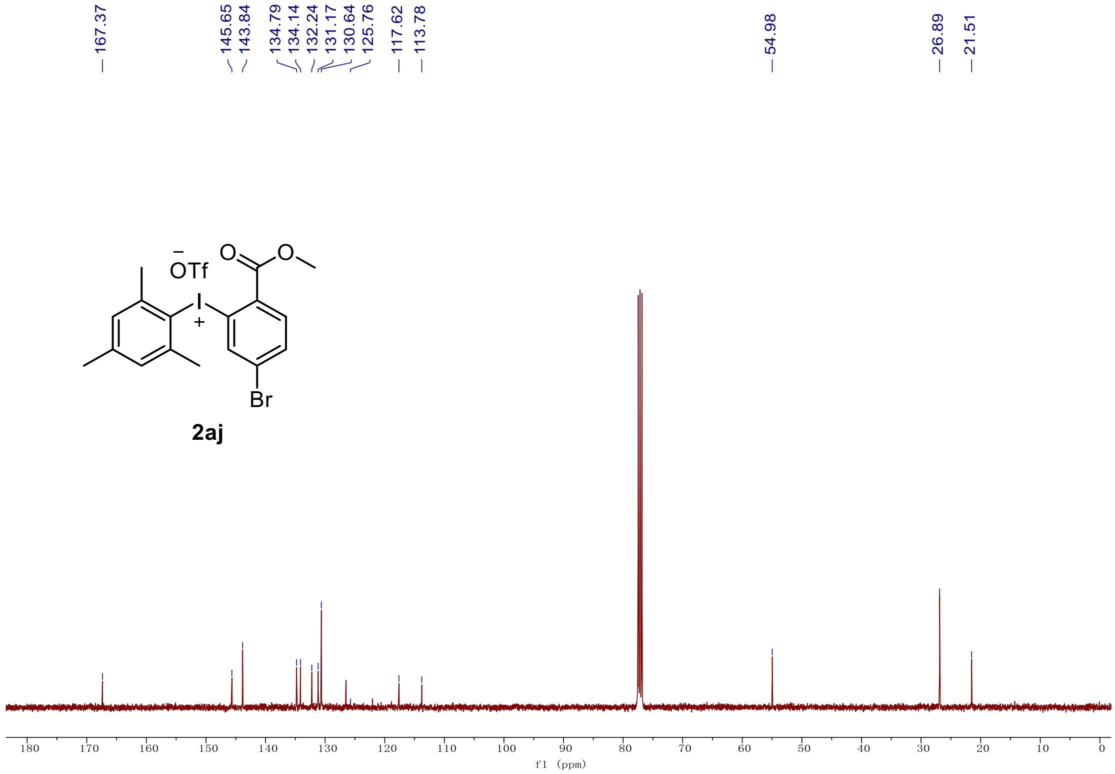

^{19}F NMR of **2aj** ($CDCl_3$, 376 M)

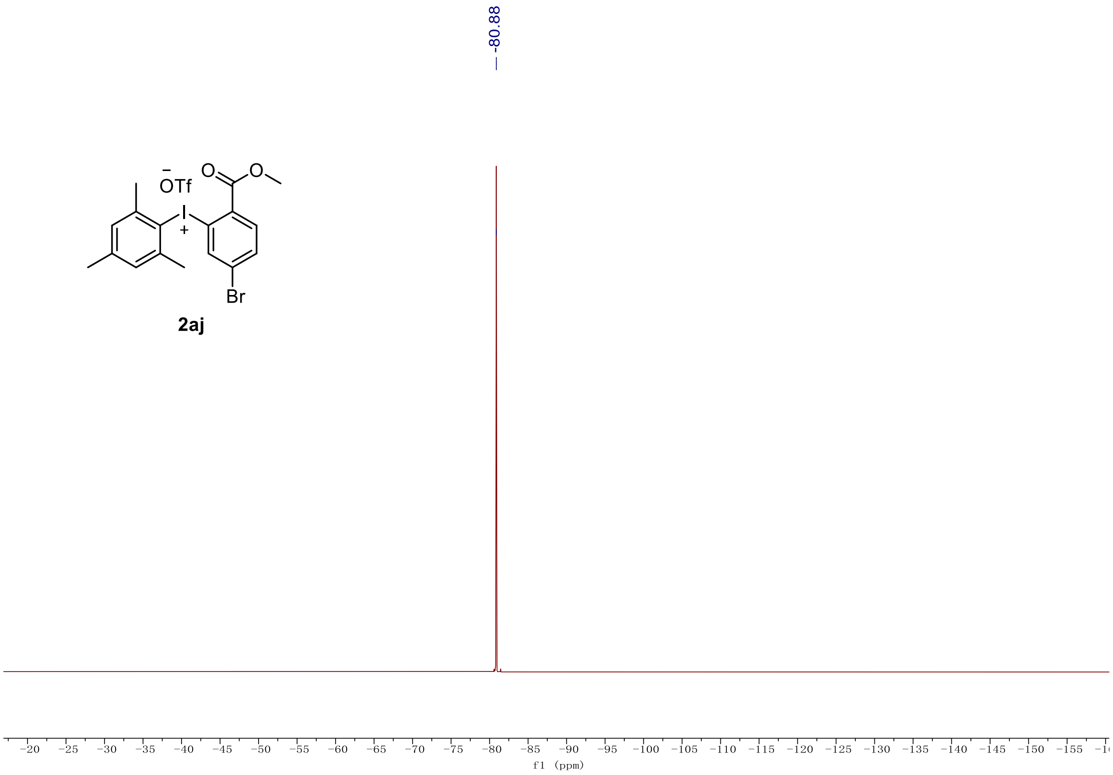

2aj

^{1}H NMR of **2ak** ($CDCl_3$, 400 M)

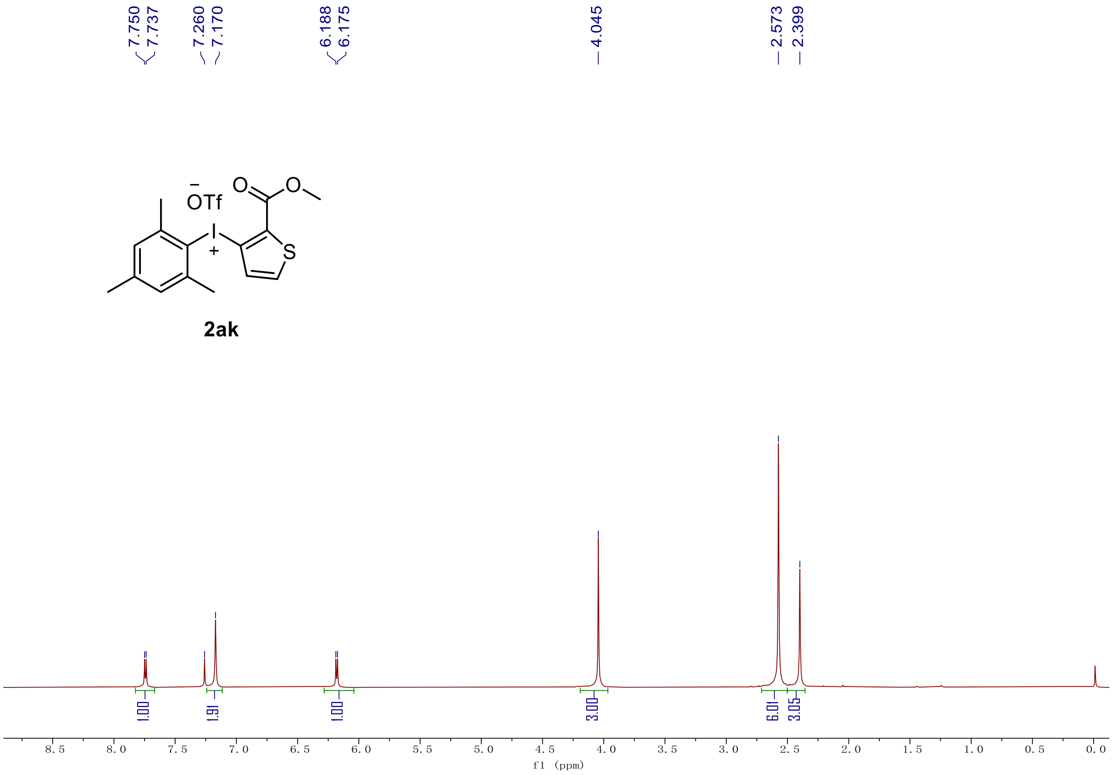

^{13}C NMR of **2ak** ($CDCl_3$, 101 M)

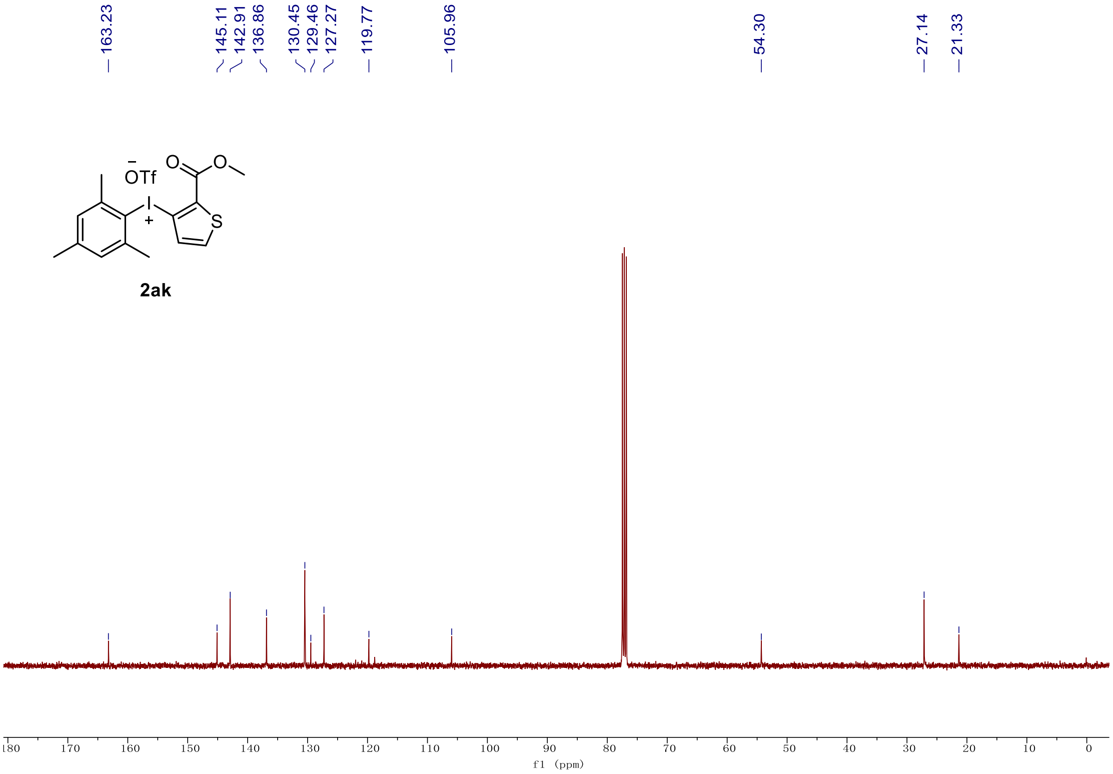

^{19}F NMR of **2ak** ($CDCl_3$, 376 M)

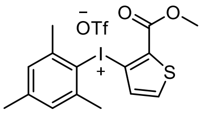

2ak

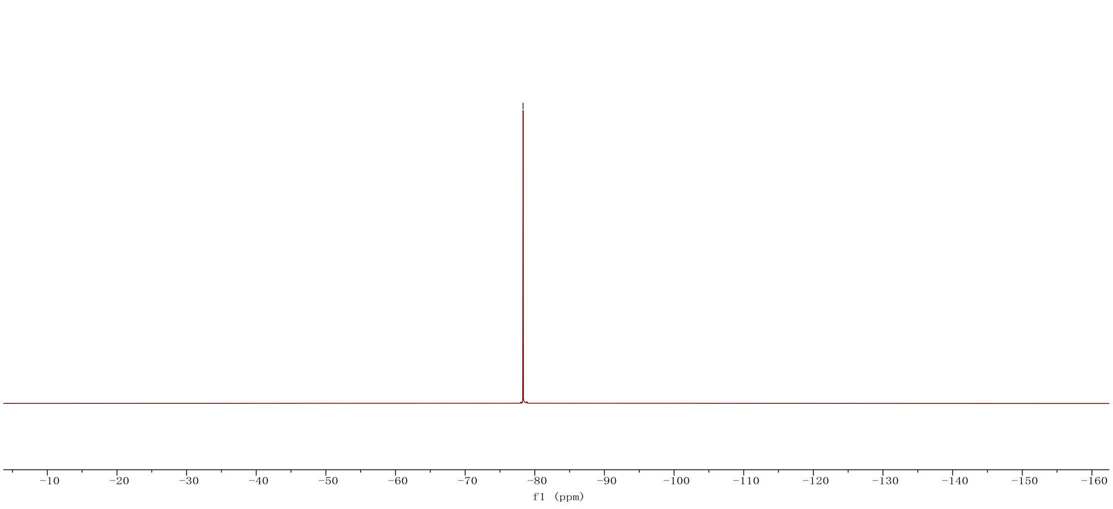

^{1}H NMR of **2al** ($CDCl_3$, 400 M)

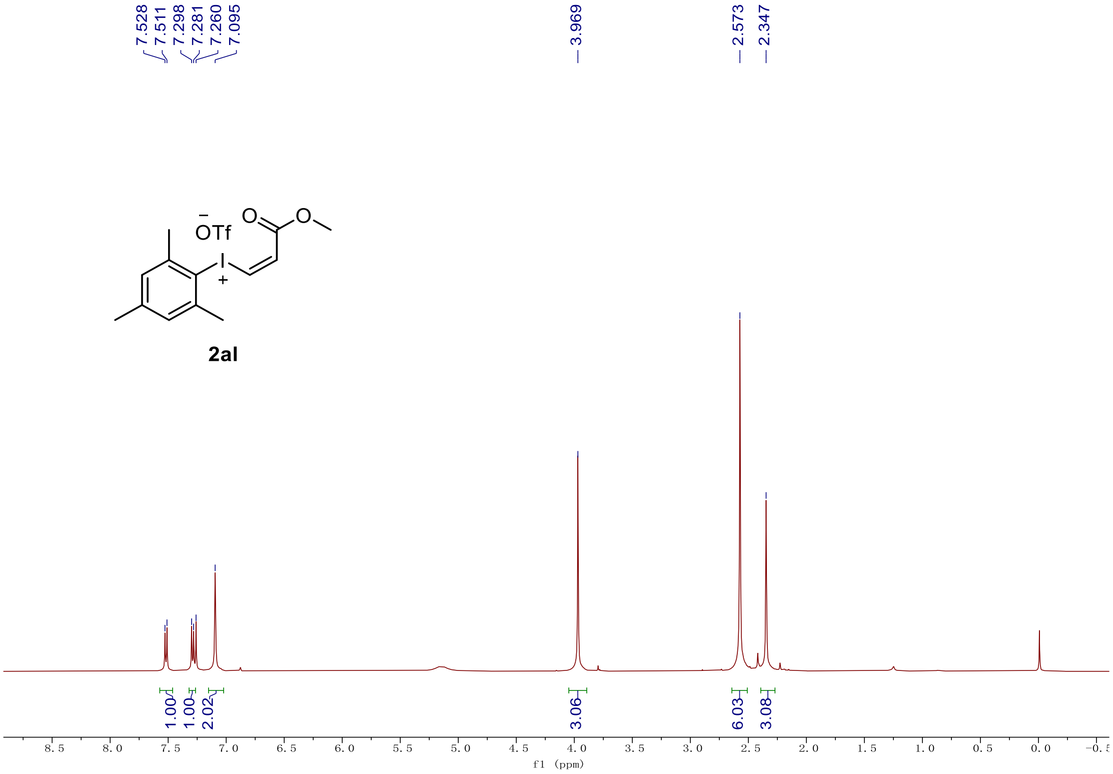

^{13}C NMR of **2al** ($CDCl_3$, 101 M)

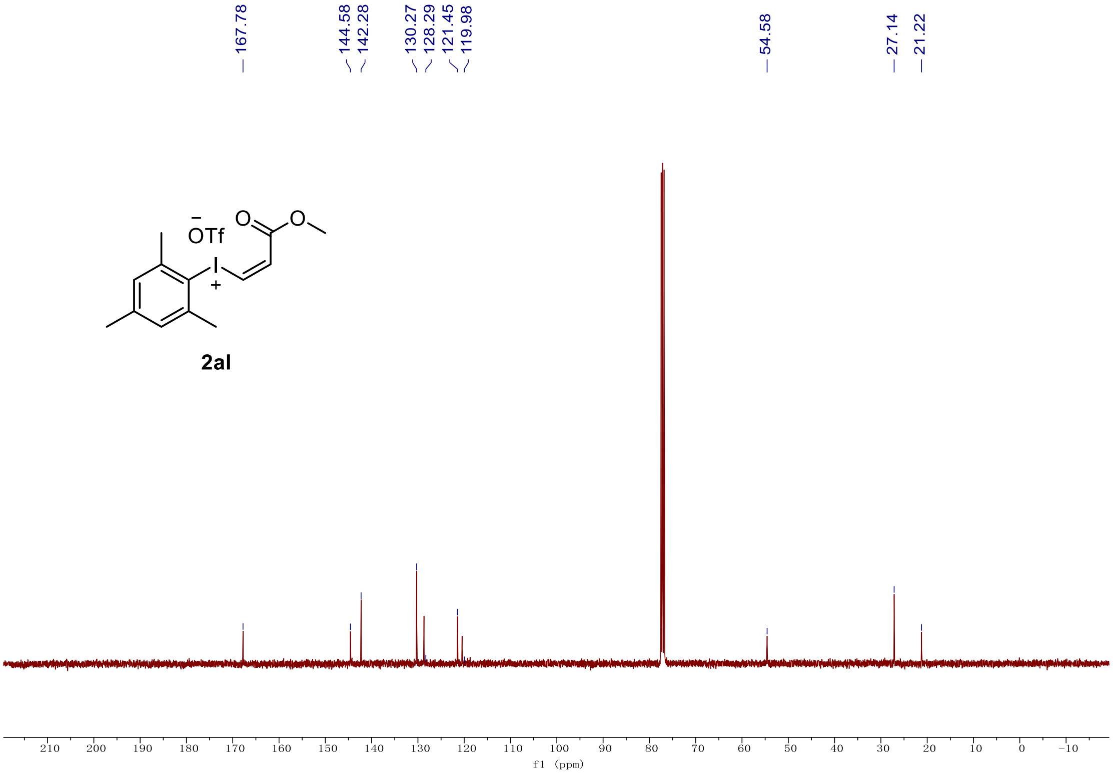

^{19}F NMR of **2al** ($CDCl_3$, 376 M)

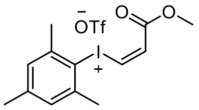

2al

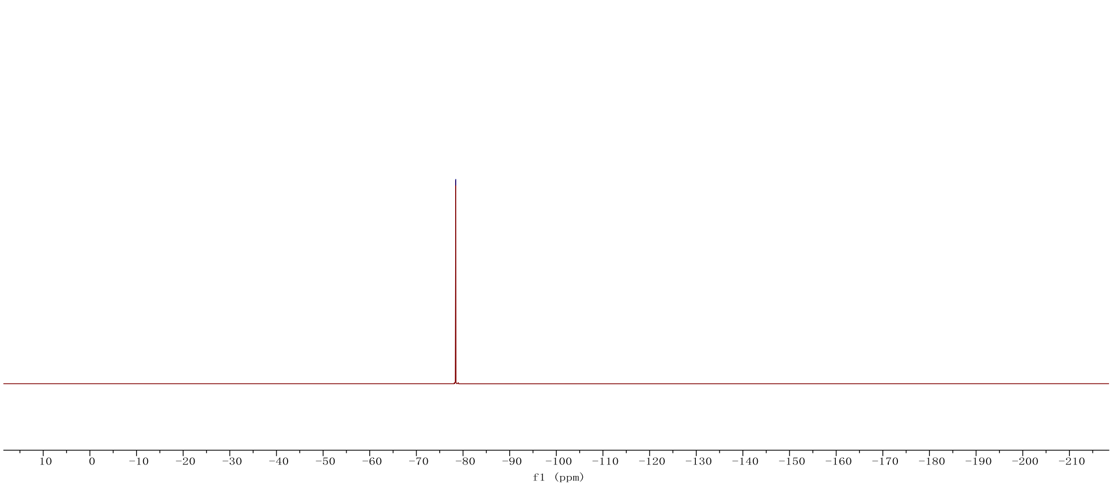

^{1}H NMR of **2ao** ($CDCl_3$, 400 M)

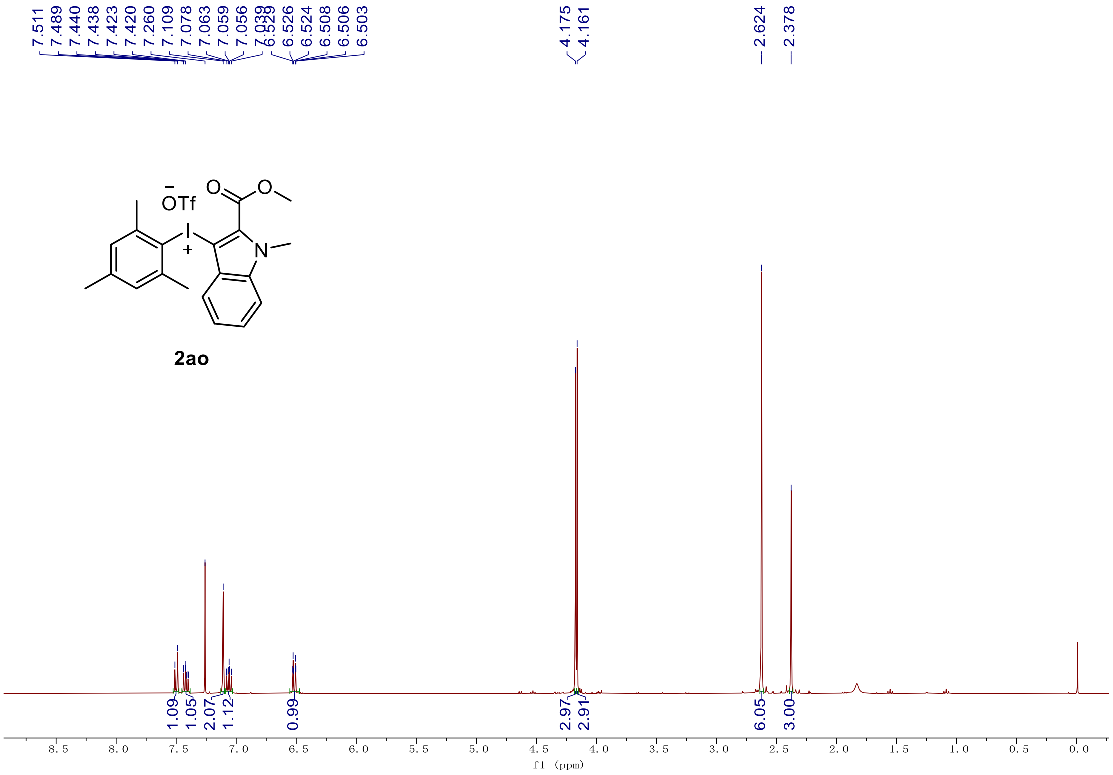

^{13}C NMR of **2ao** ($CDCl_3$, 101 M)

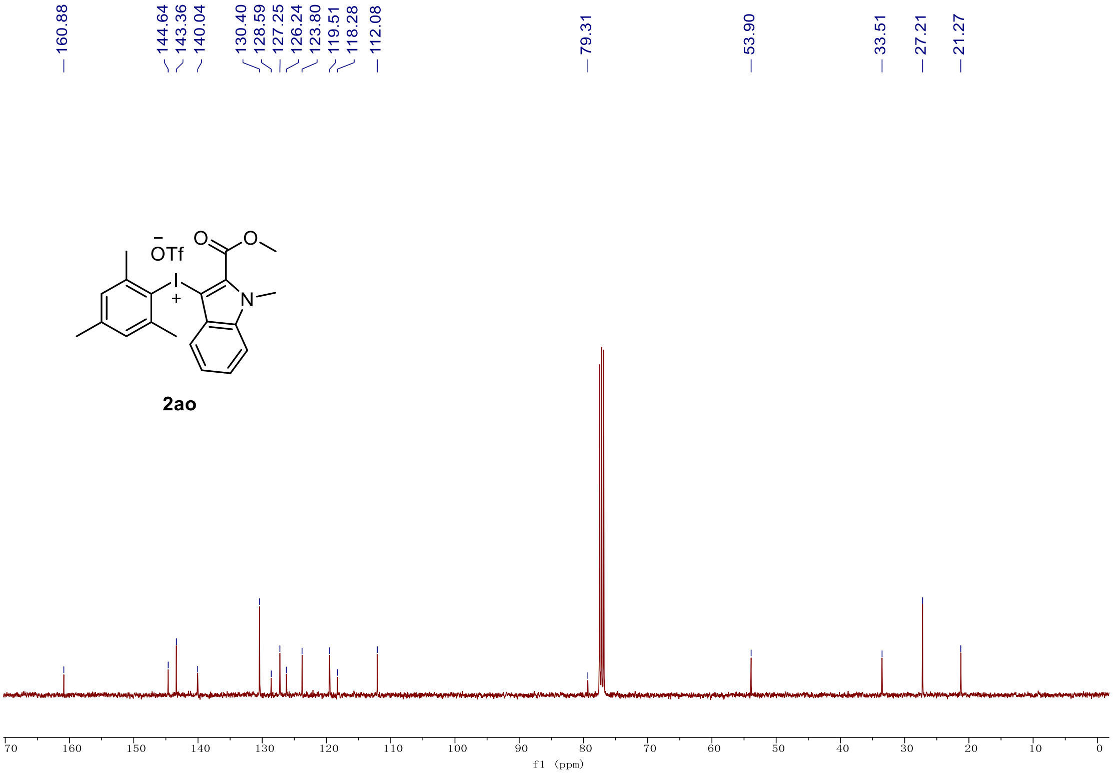

^{19}F NMR of **2ao** ($CDCl_3$, 376 M)

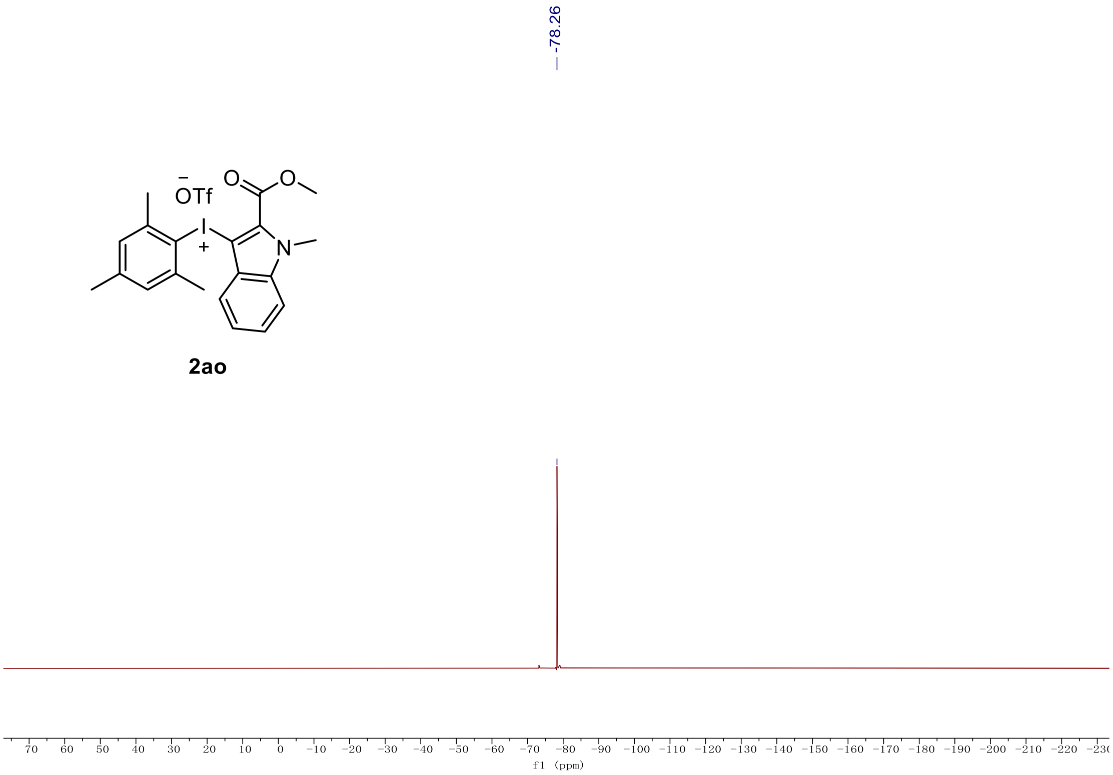

^{1}H NMR of **3b** ($CDCl_3$, 400 M)

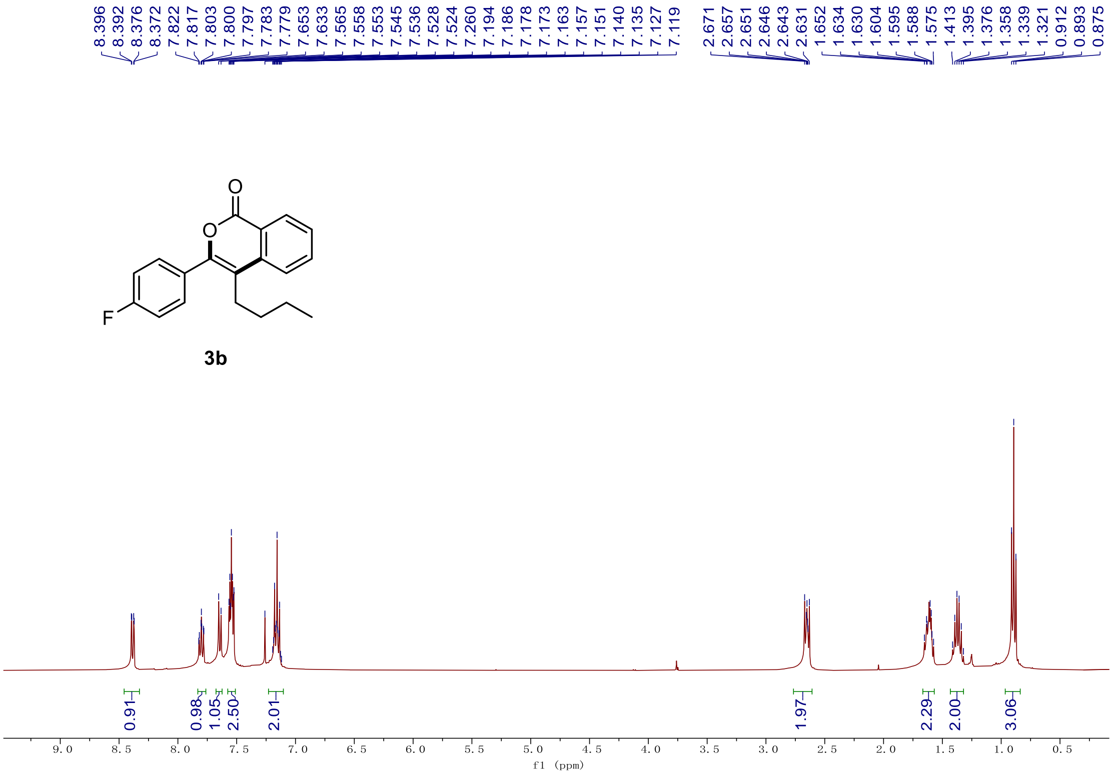

^{13}C NMR of **3b** ($CDCl_3$, 101 M)

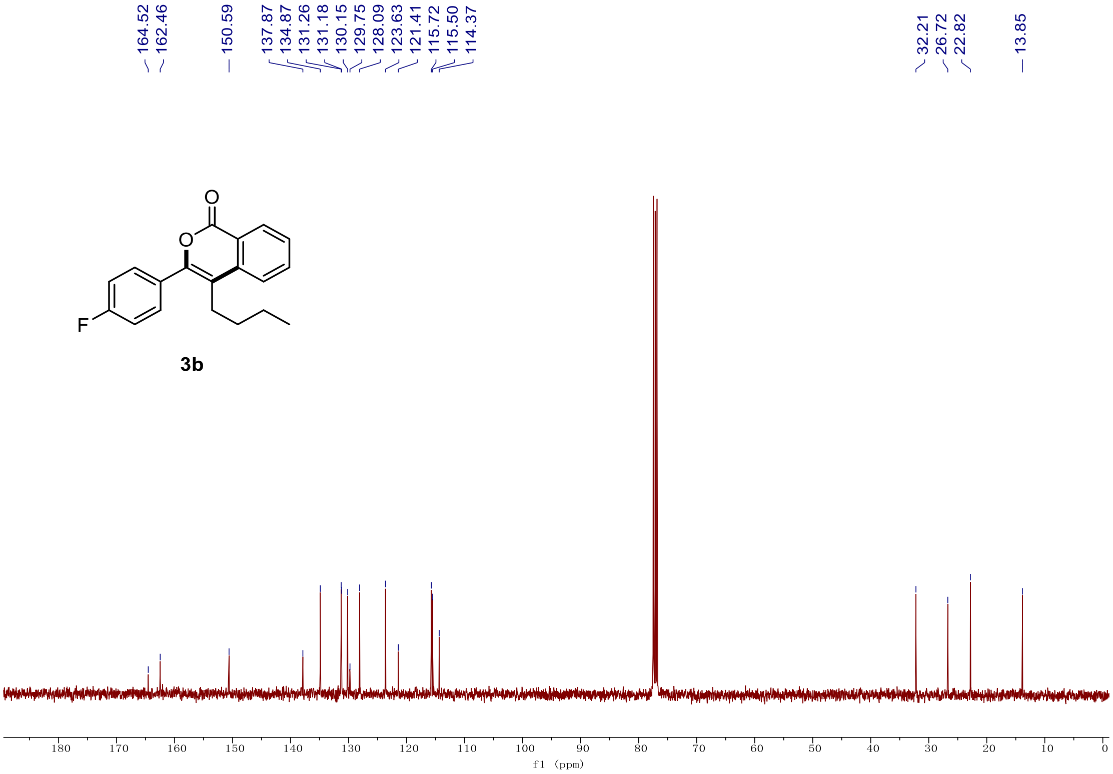

^{19}F NMR of **3b** ($CDCl_3$, 376 M)

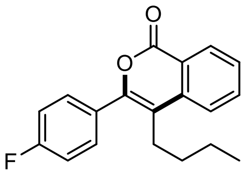

3b

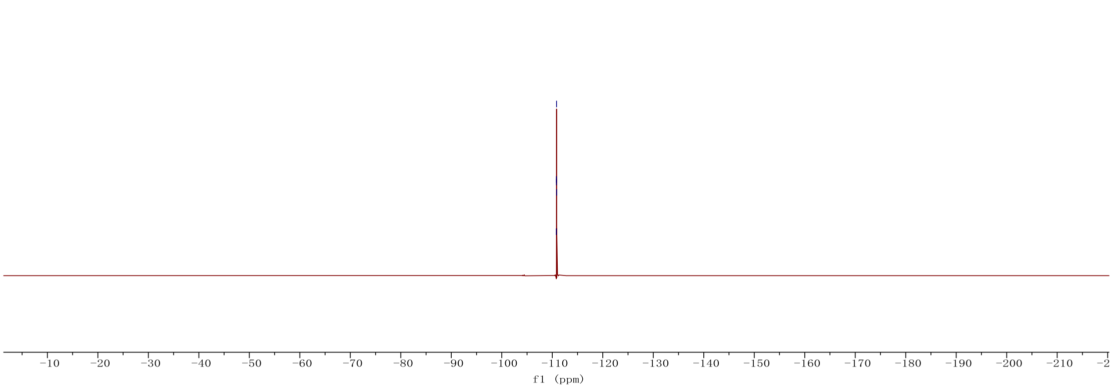

^{1}H NMR of **3c** ($CDCl_3$, 400 M)

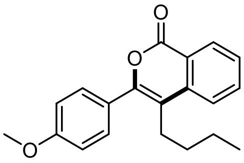

3c

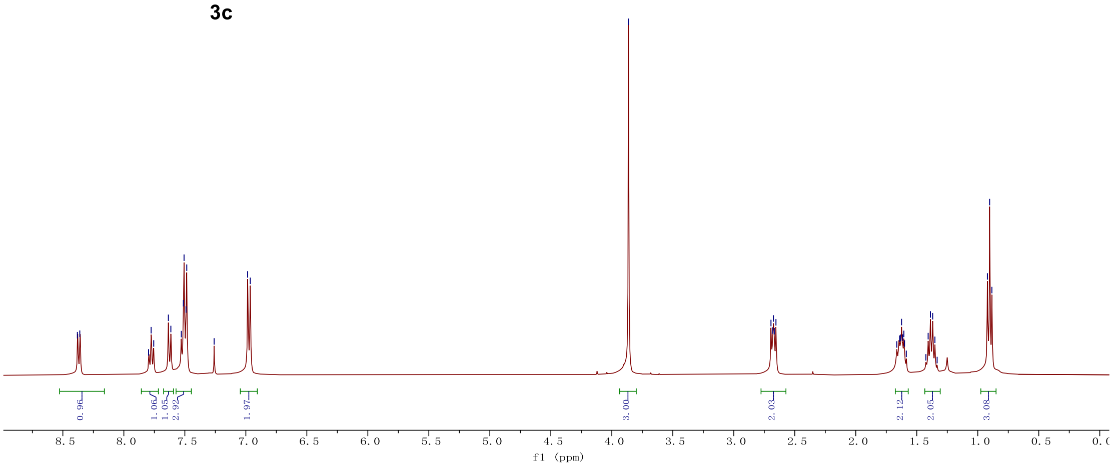

^{13}C NMR of **3c** ($CDCl_3$, 101 M)

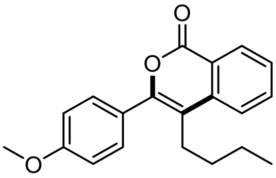

3c

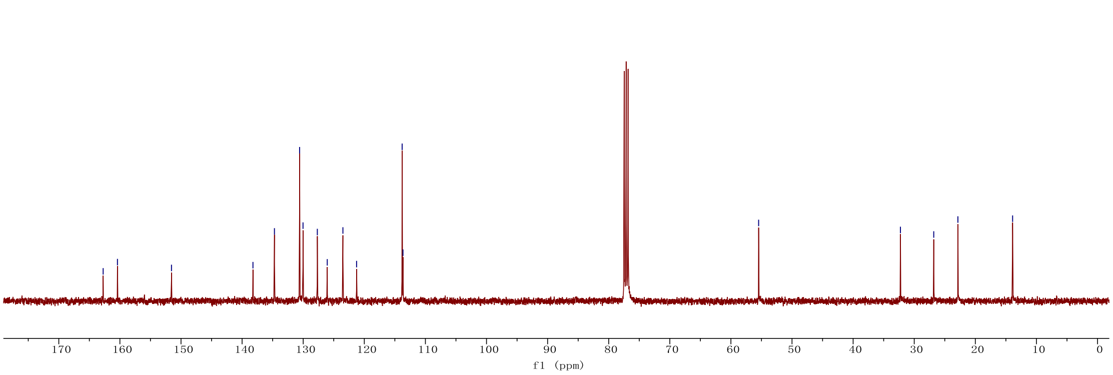

^{1}H NMR of **3d** ($CDCl_3$, 400 M)

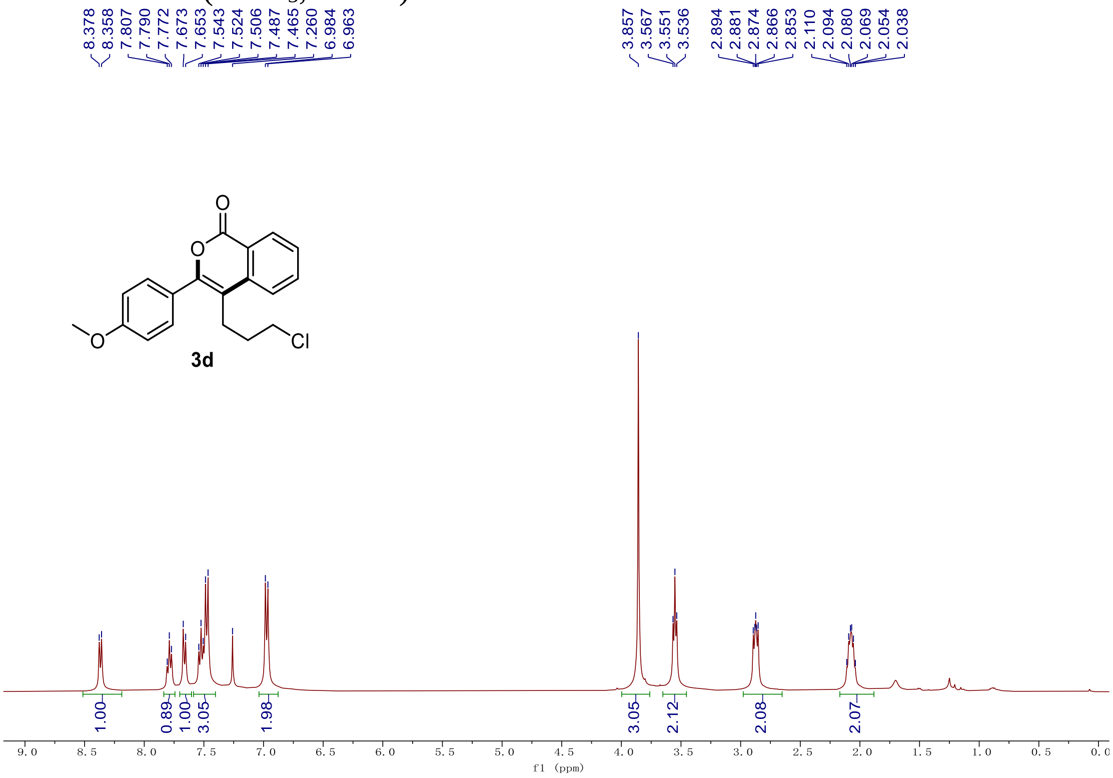

^{13}C NMR of **3d** ($CDCl_3$, 101 M)

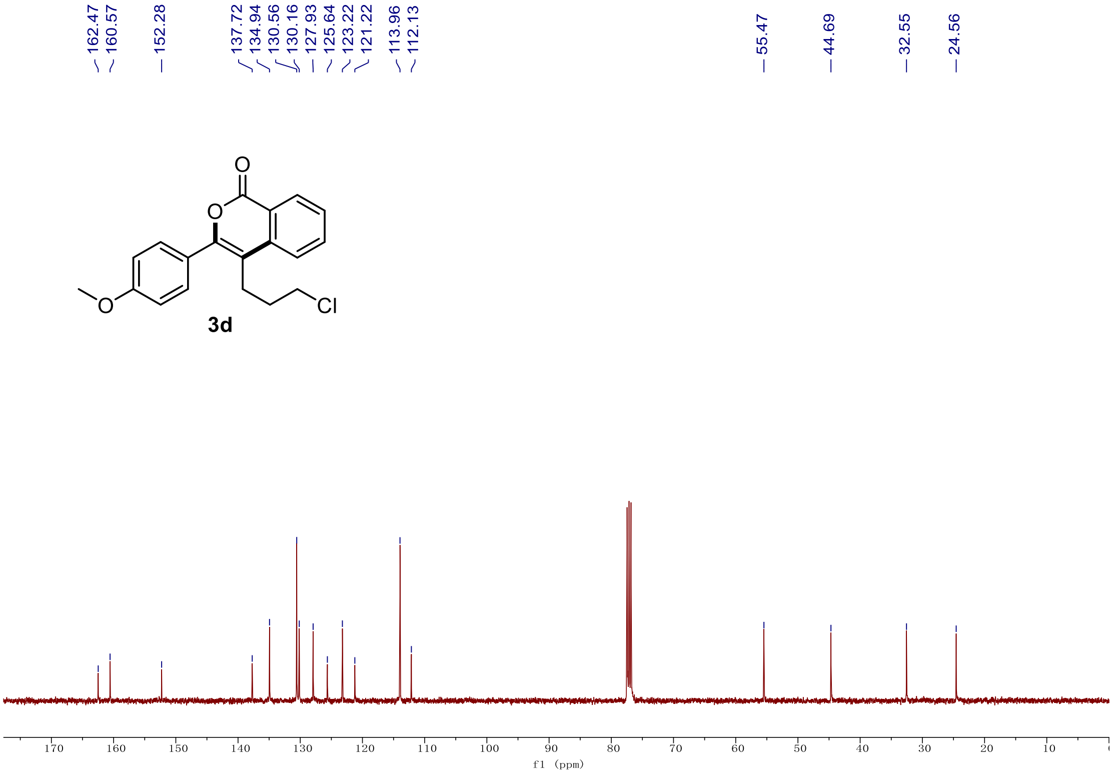

^{1}H NMR of **3e** ($CDCl_3$, 400 M)

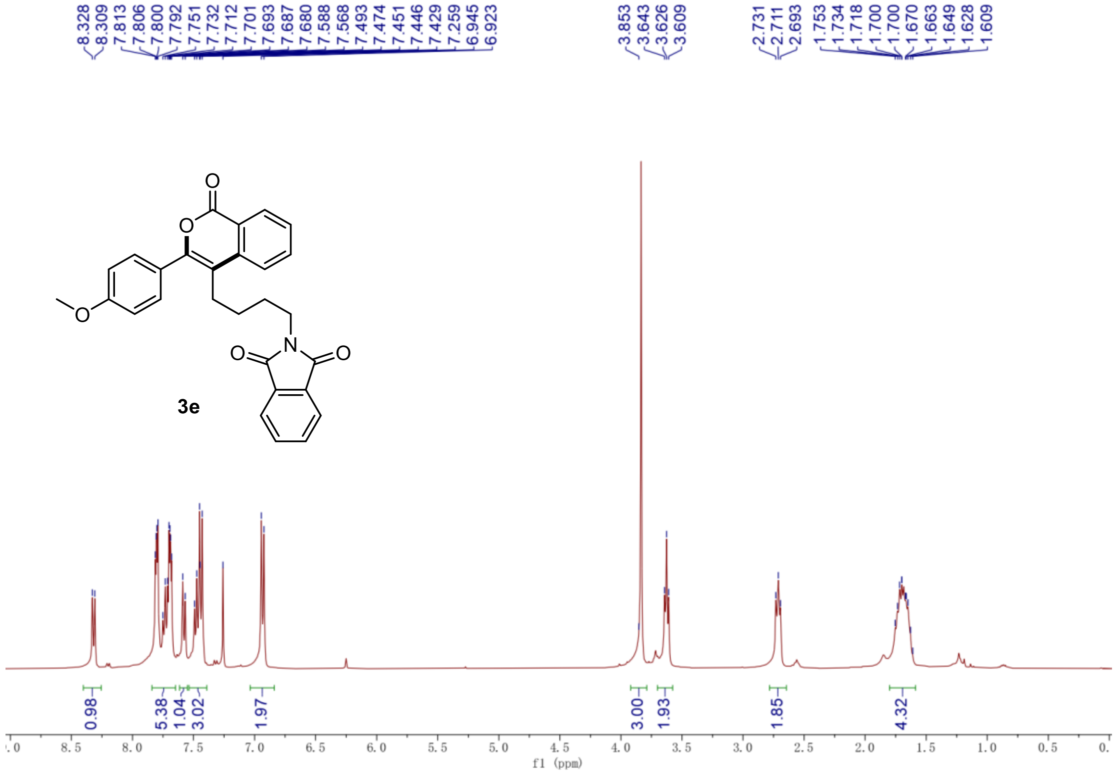

^{13}C NMR of **3e** ($CDCl_3$, 101 M)

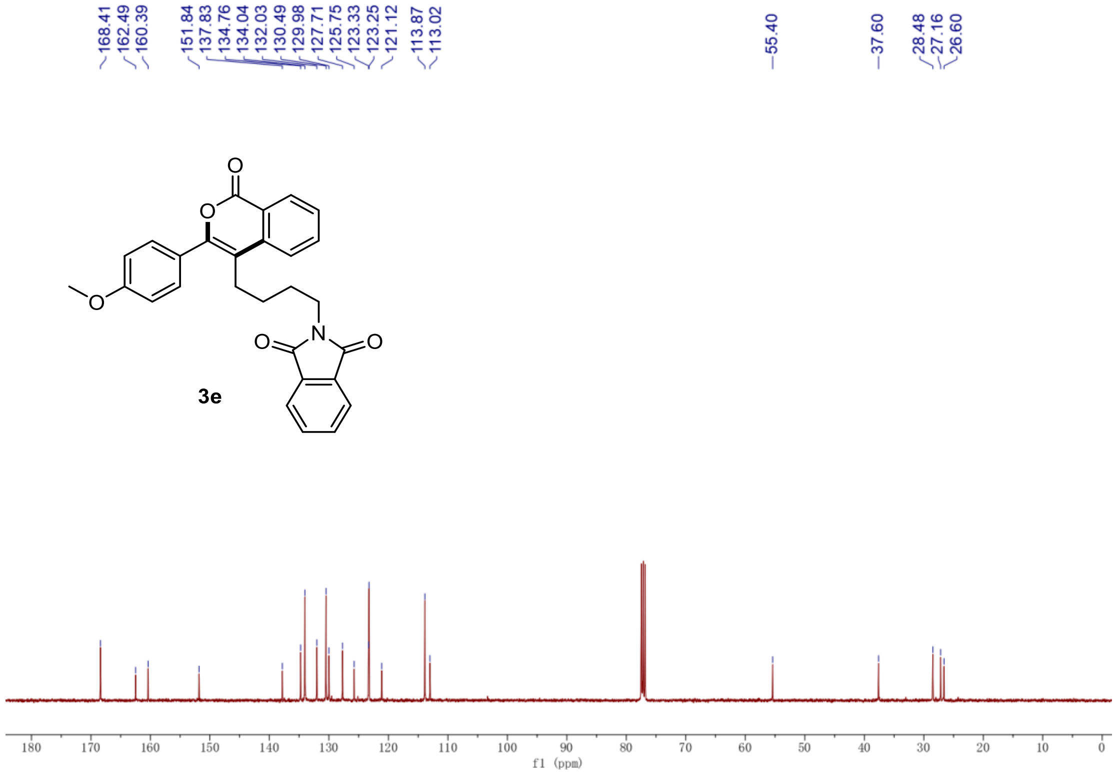

^{1}H NMR of **3f** ($CDCl_3$, 400 M)

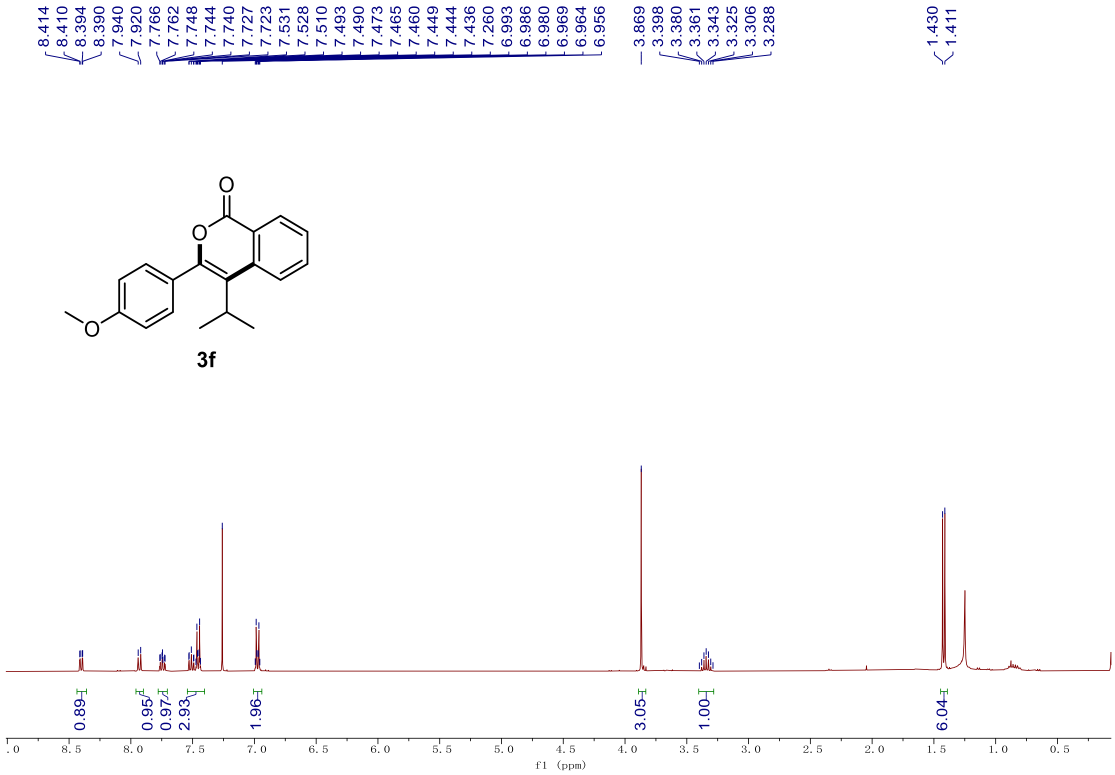

^{13}C NMR of **3f** ($CDCl_3$, 101 M)

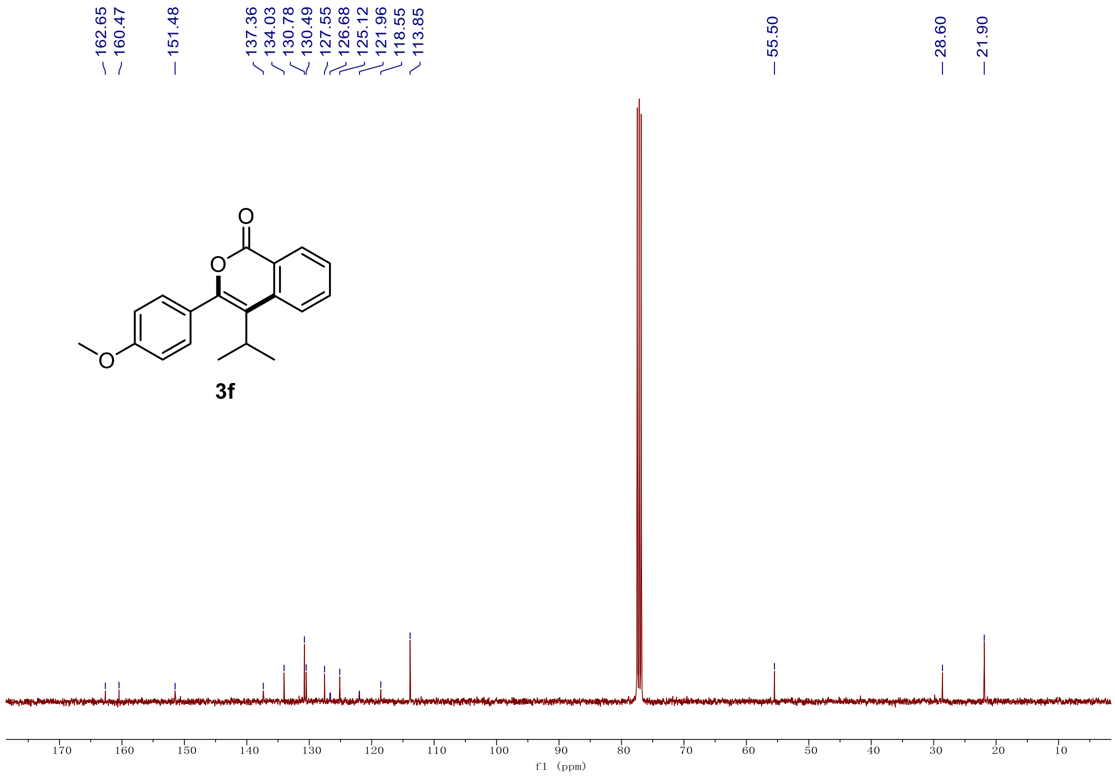

^{1}H NMR of **3g** ($CDCl_3$, 400 M)

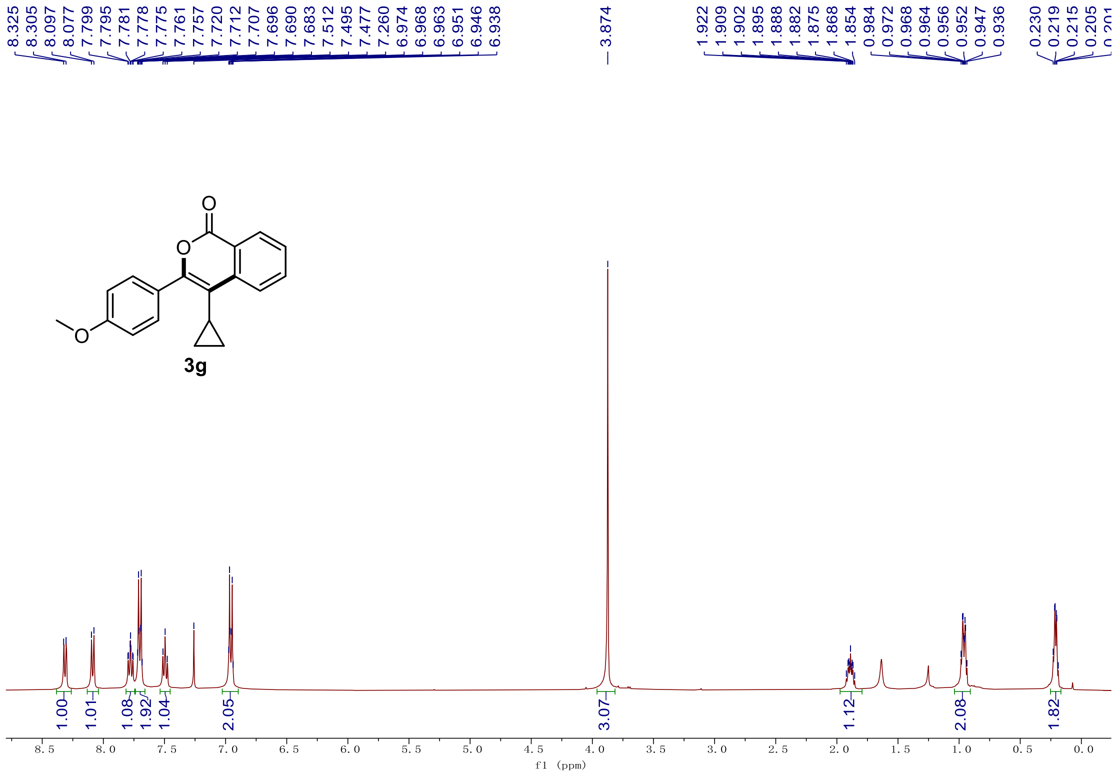

^{13}C NMR of **3g** ($CDCl_3$, 101 M)

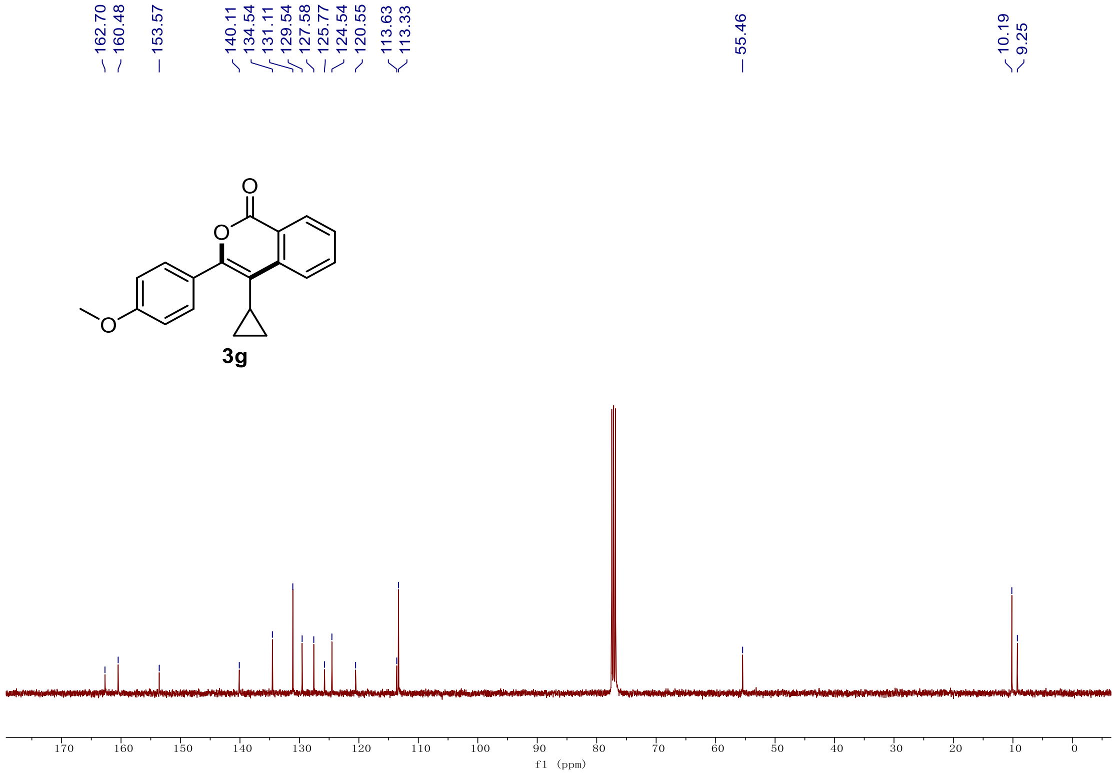

^{1}H NMR of **3h** ($CDCl_3$, 400 M)

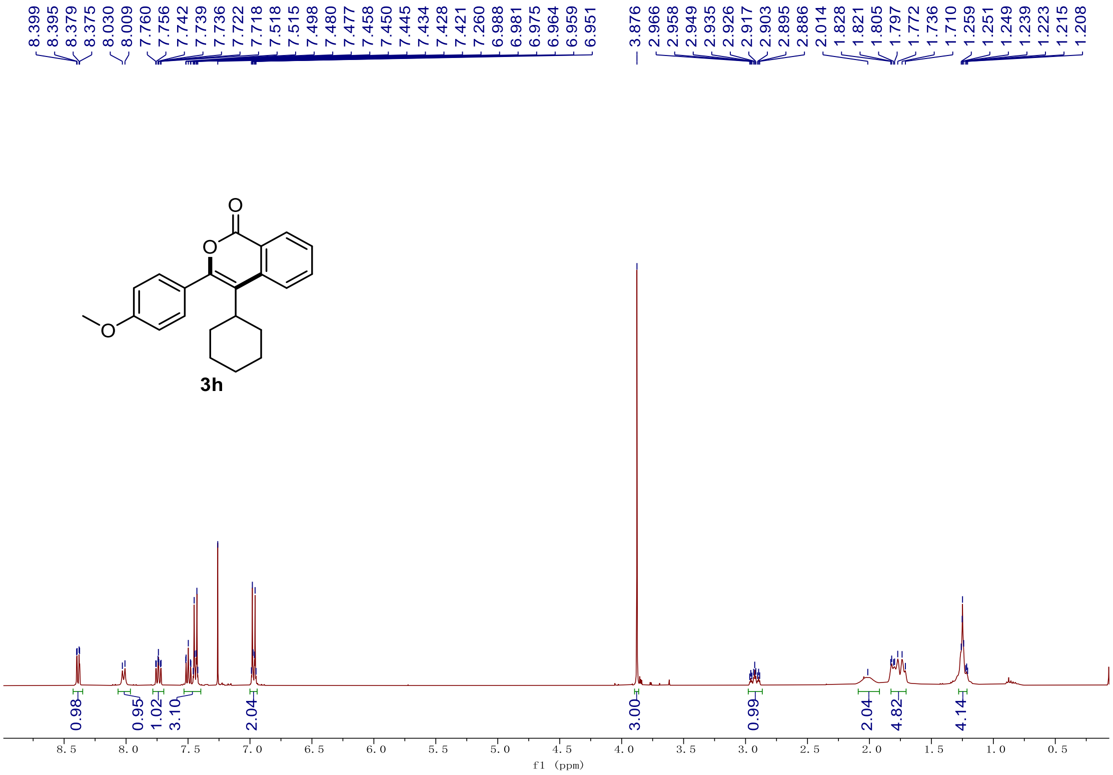

^{13}C NMR of **3h** ($CDCl_3$, 101 M)

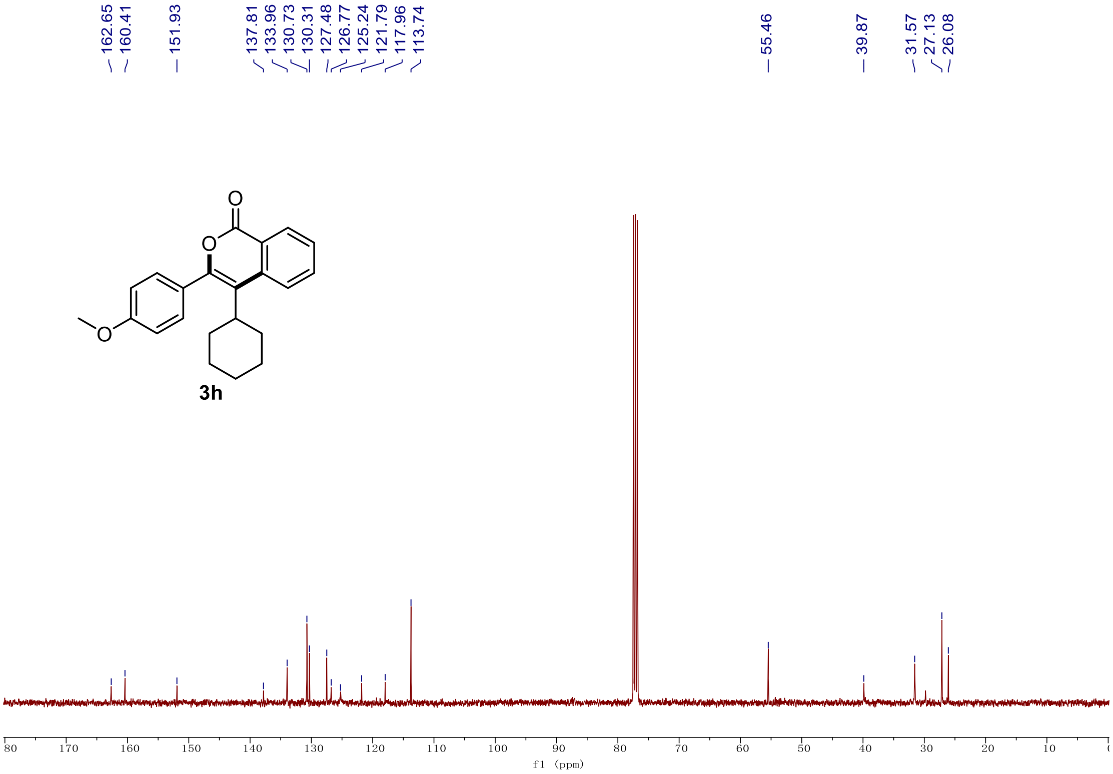

^{1}H NMR of **3i** ($CDCl_3$, 400 M)

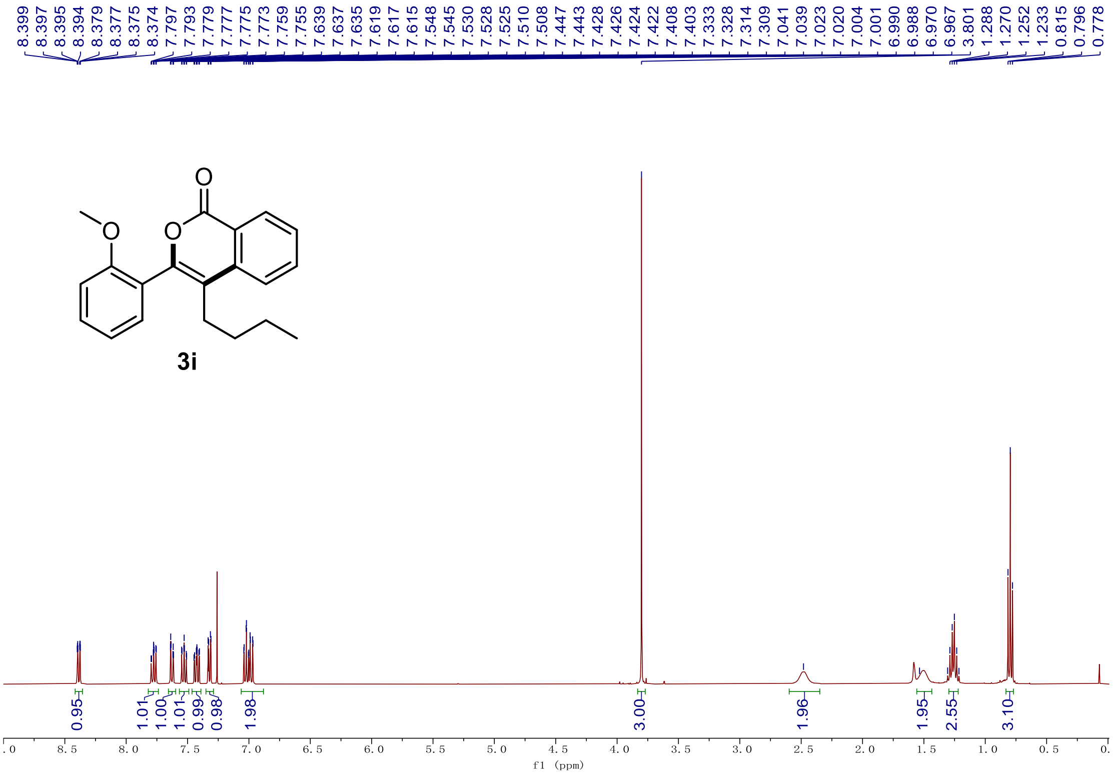

^{13}C NMR of **3i** ($CDCl_3$, 101 M)

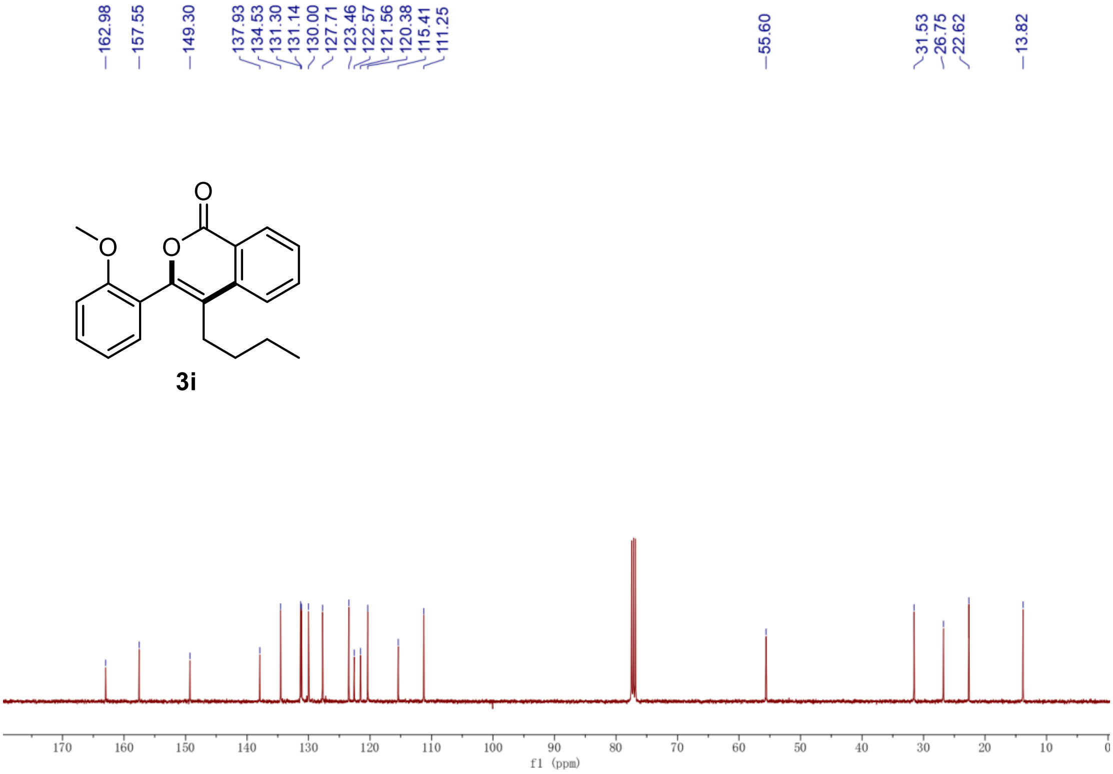

^{1}H NMR of **3j** ($CDCl_3$, 400 M)

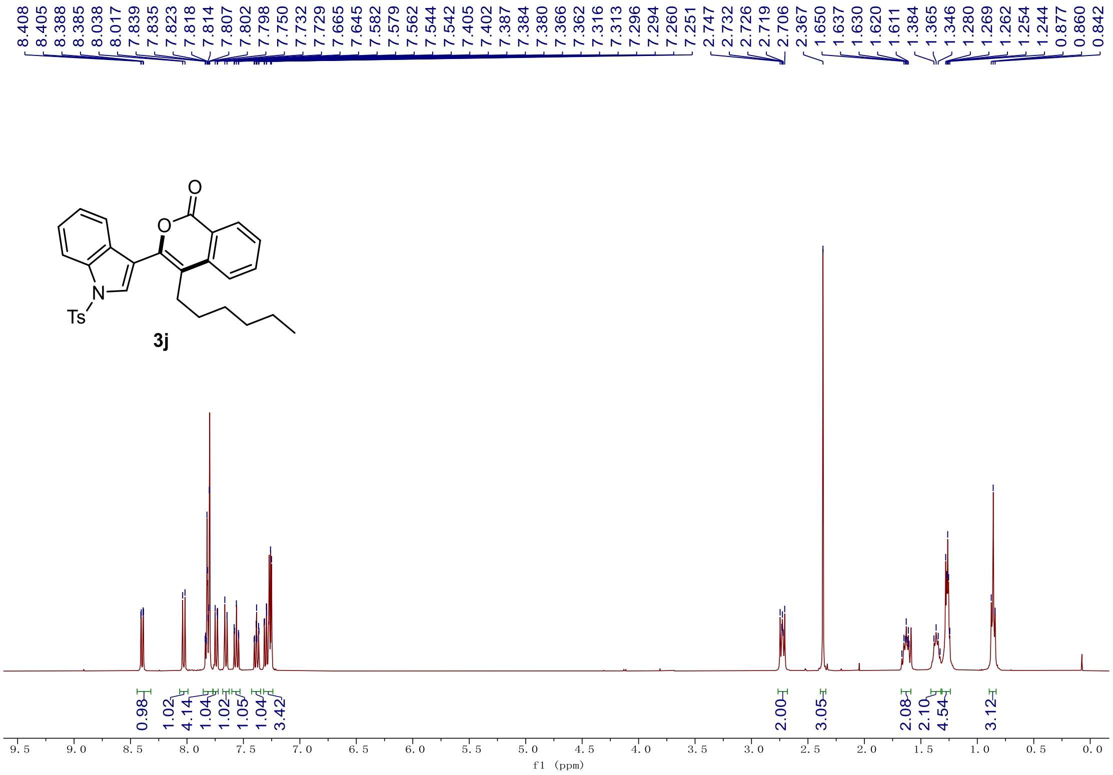

^{13}C NMR of **3j** ($CDCl_3$, 101 M)

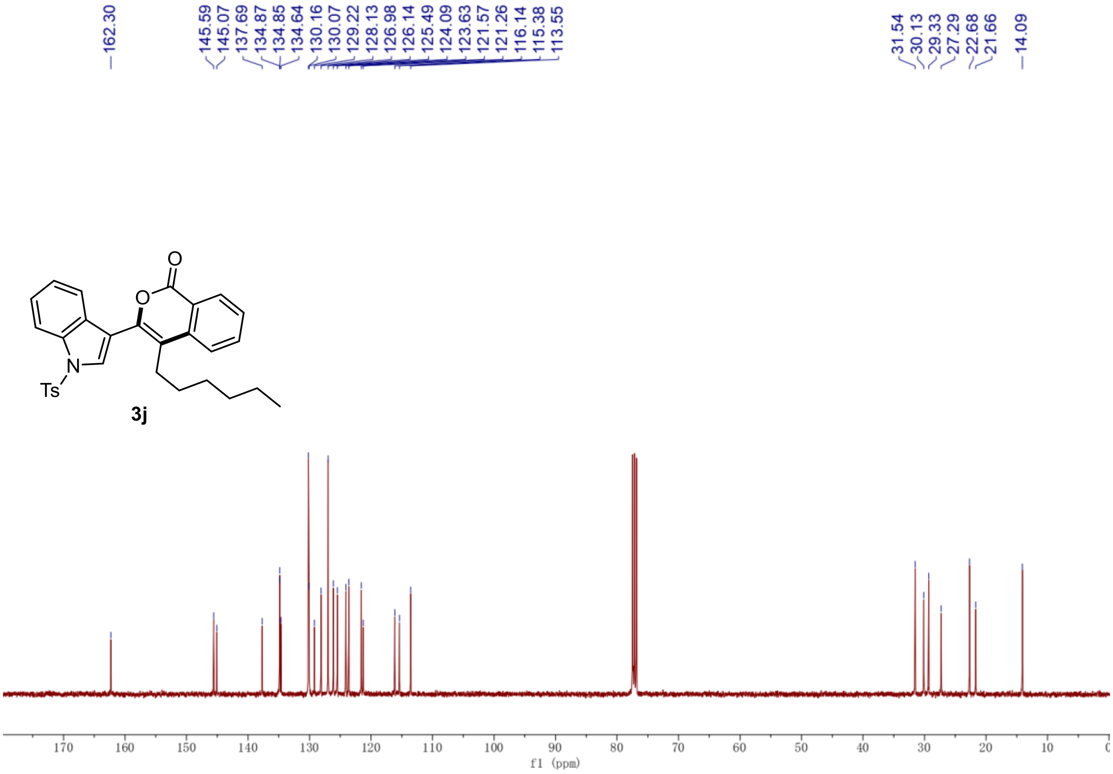

^{1}H NMR of **3k** ($CDCl_3$, 400 M)

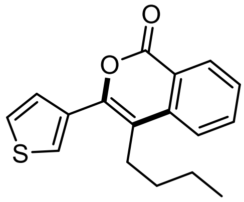

3k

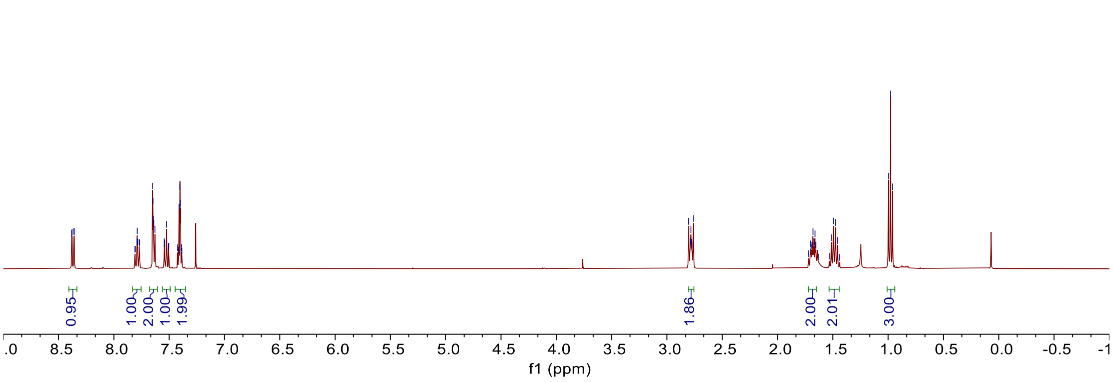

^{13}C NMR of **3k** ($CDCl_3$, 101 M)

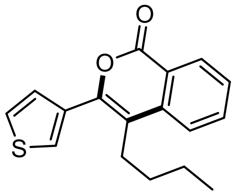

3k

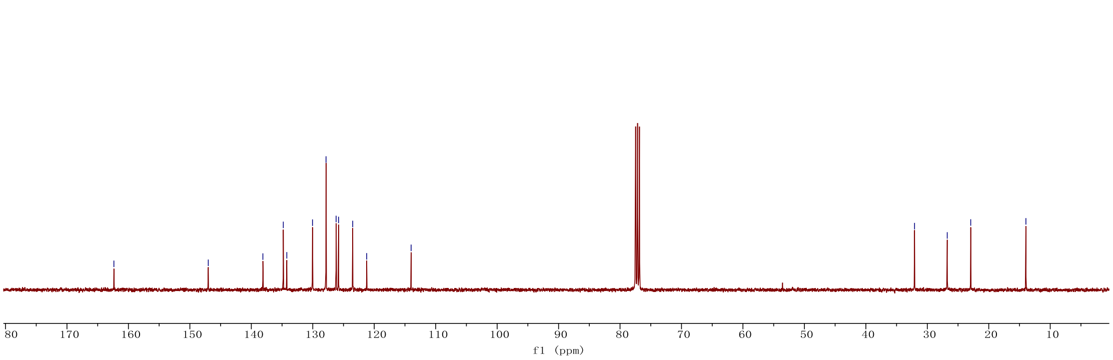

^{1}H NMR of **3l** ($CDCl_3$, 400 M)

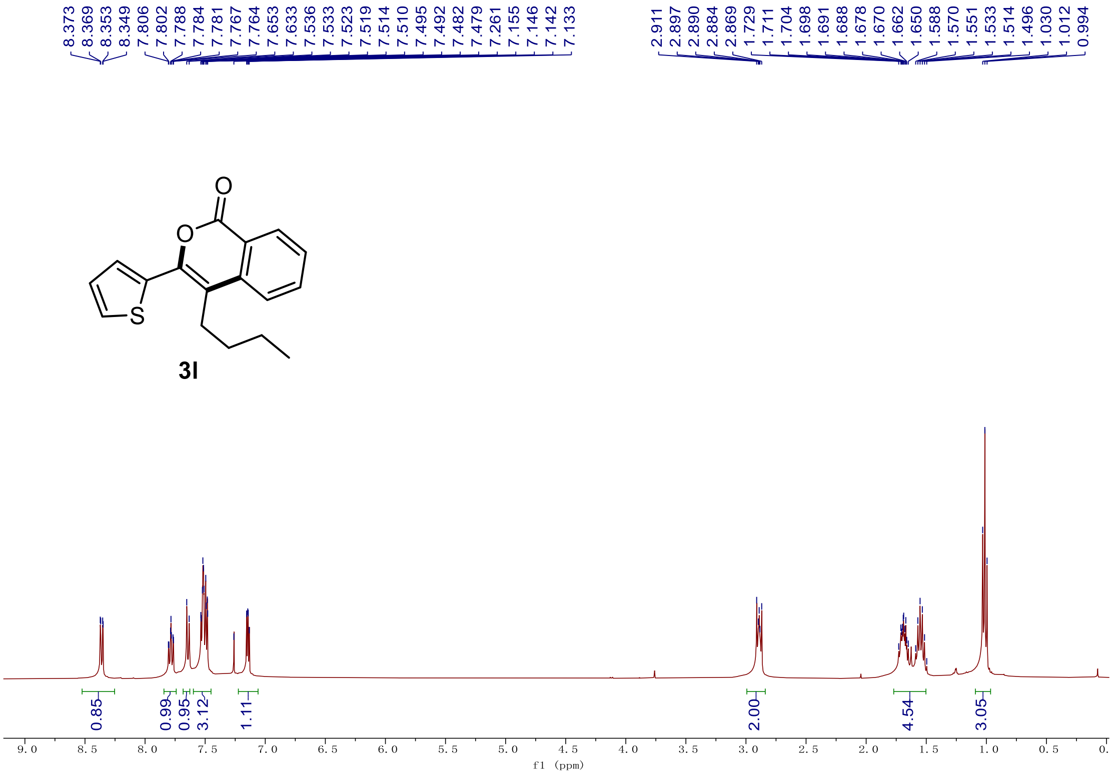

^{13}C NMR of **3l** ($CDCl_3$, 101 M)

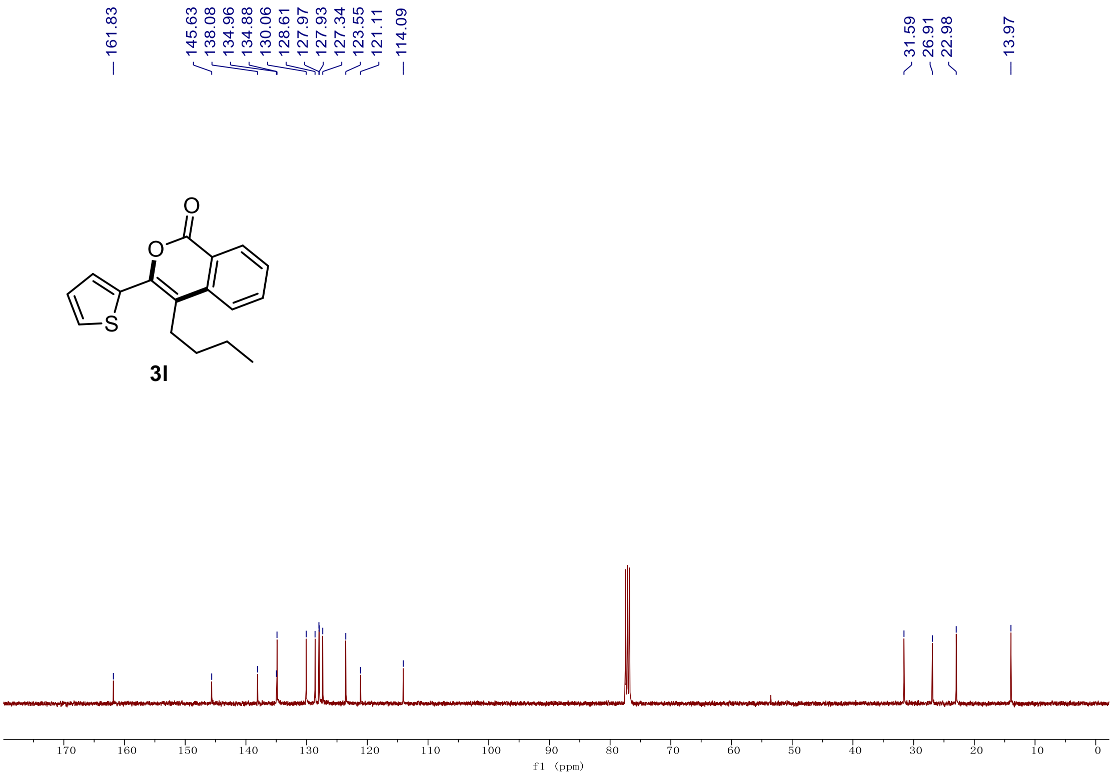

^{1}H NMR of **3q** ($CDCl_3$, 400 M)

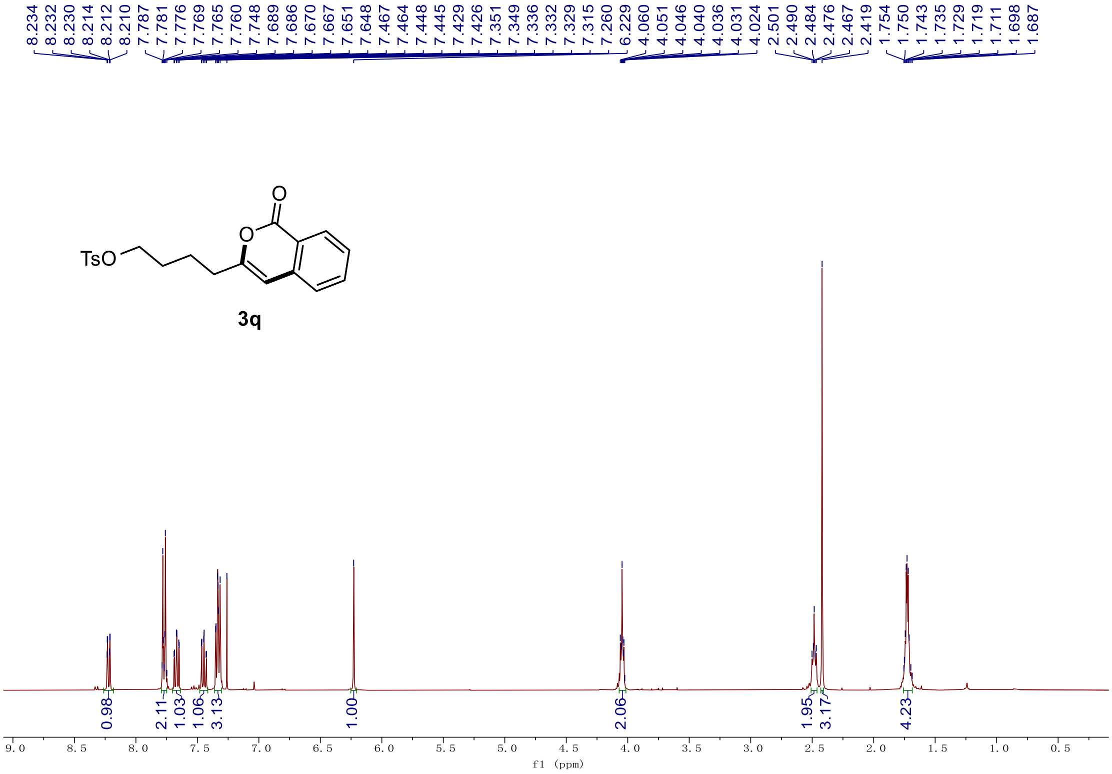

^{13}C NMR of **3q** ($CDCl_3$, 101 M)

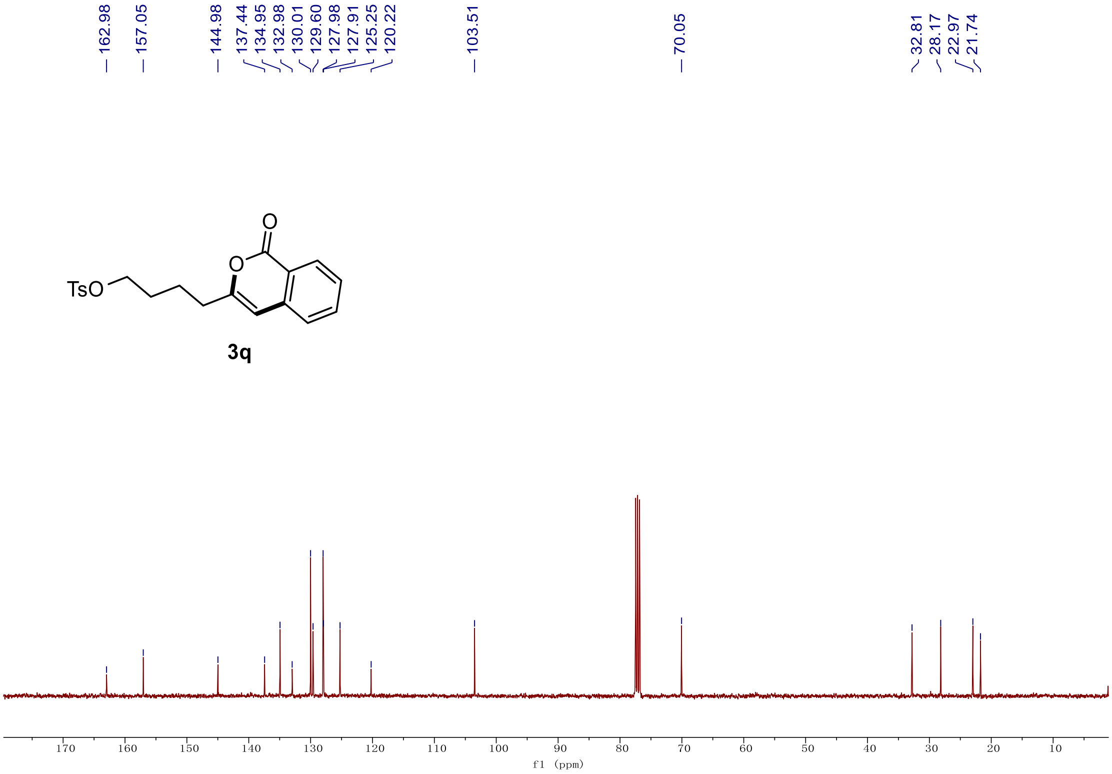

^{1}H NMR of **3r** ($CDCl_3$, 400 M)

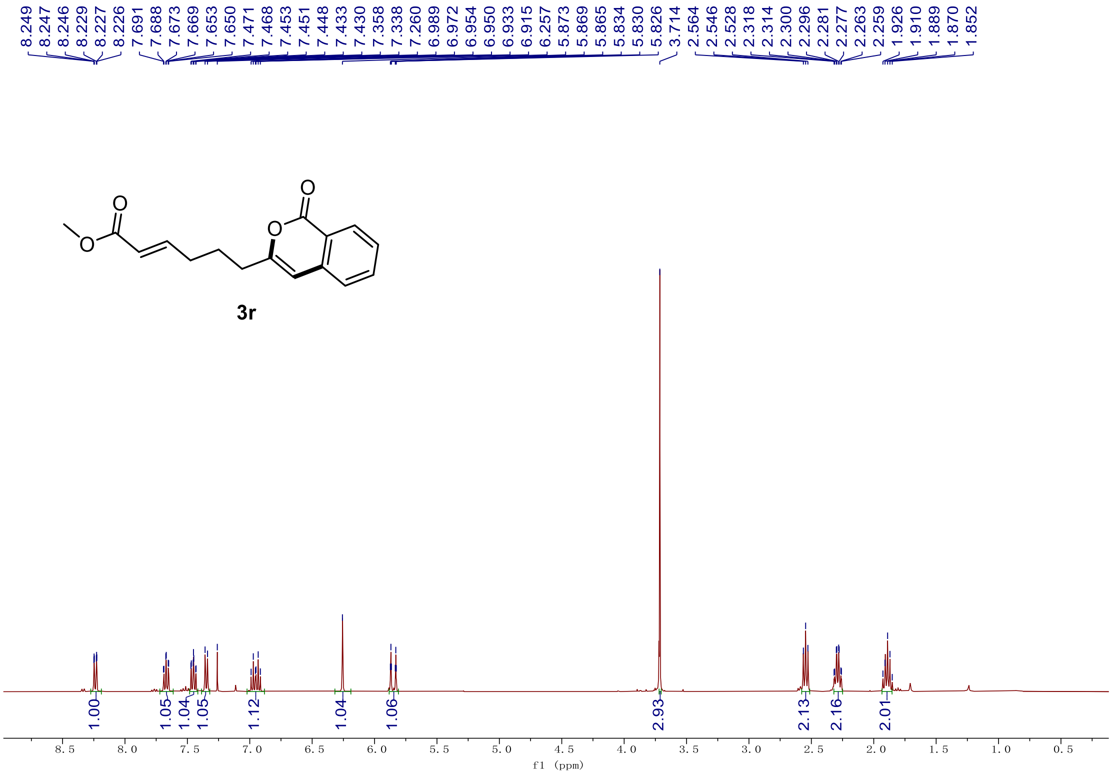

^{13}C NMR of **3r** ($CDCl_3$, 101 M)

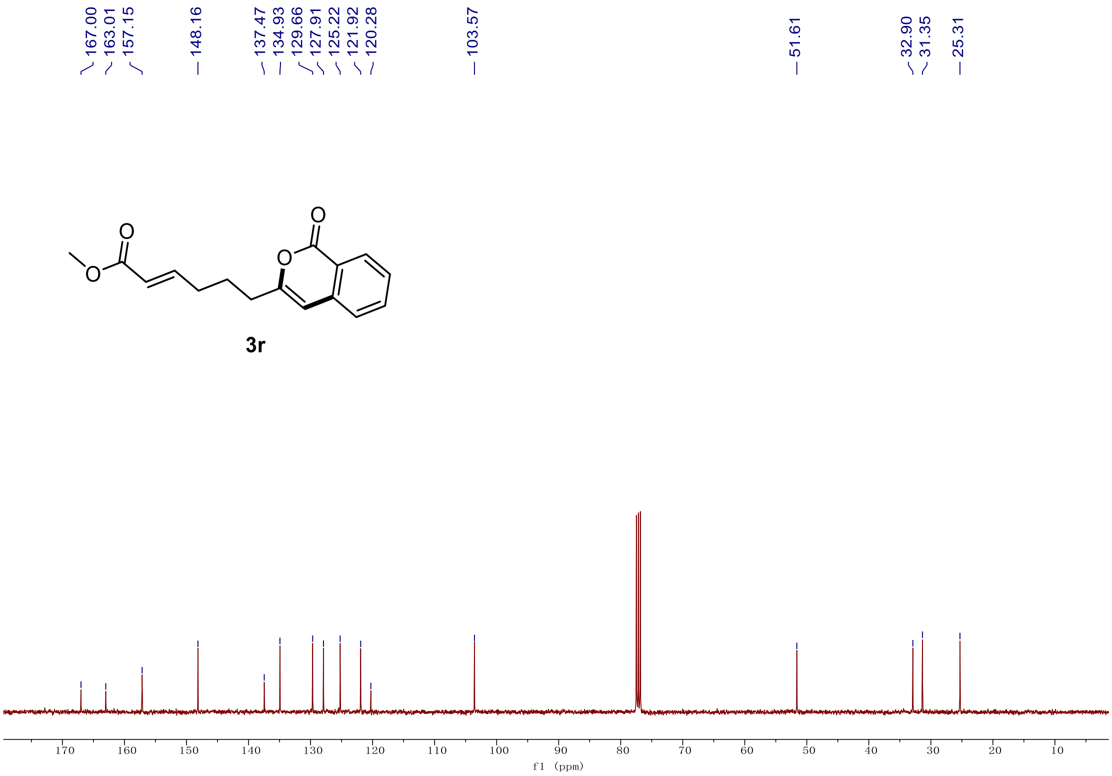

^{1}H NMR of **3v** ($CDCl_3$, 400 M)

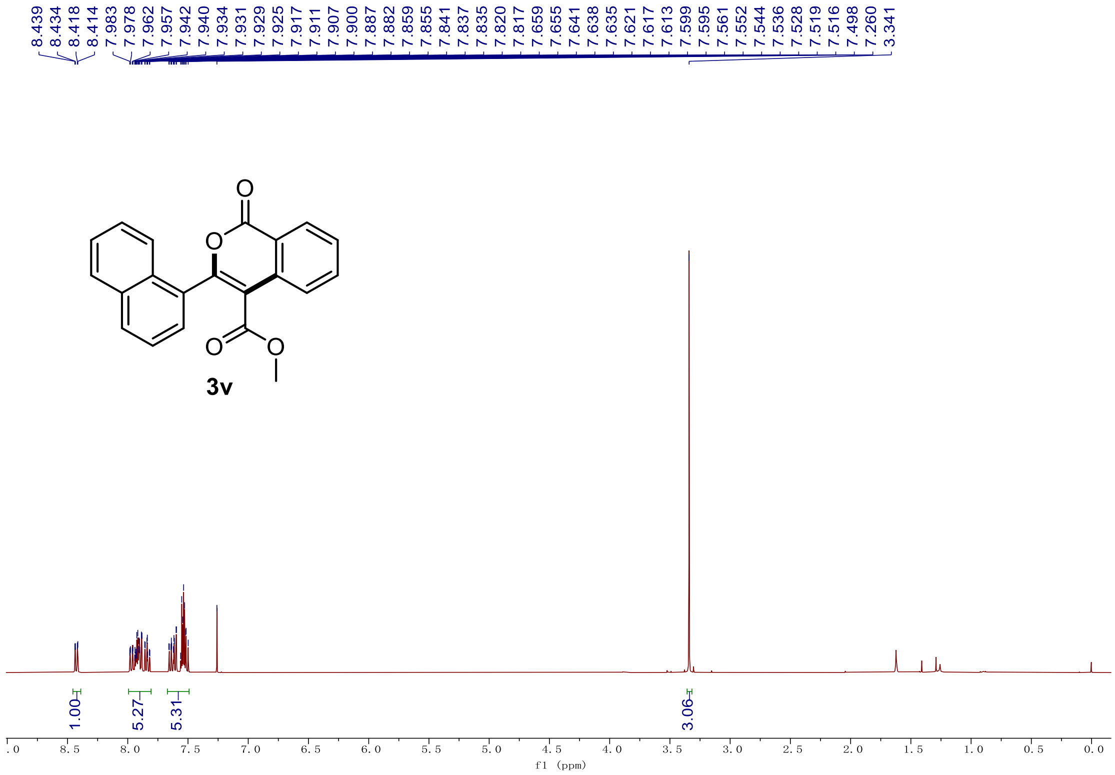

^{13}C NMR of **3v** ($CDCl_3$, 101 M)

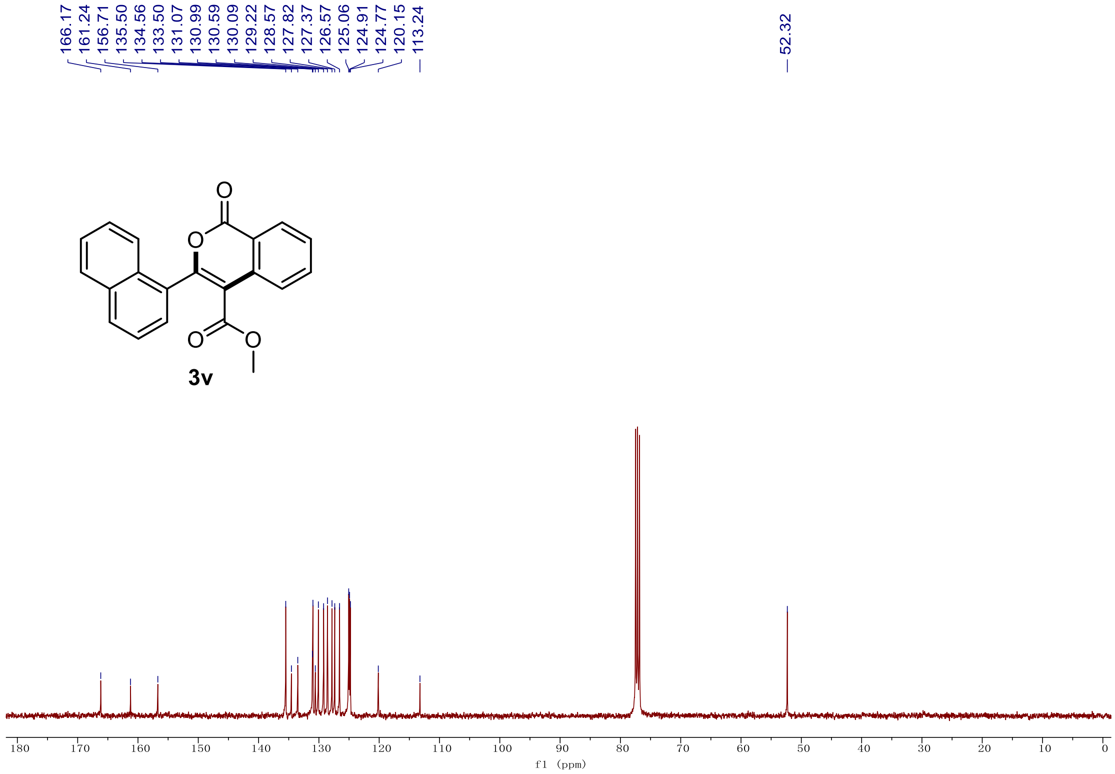

^{1}H NMR of **3w** ($CDCl_3$, 400 M)

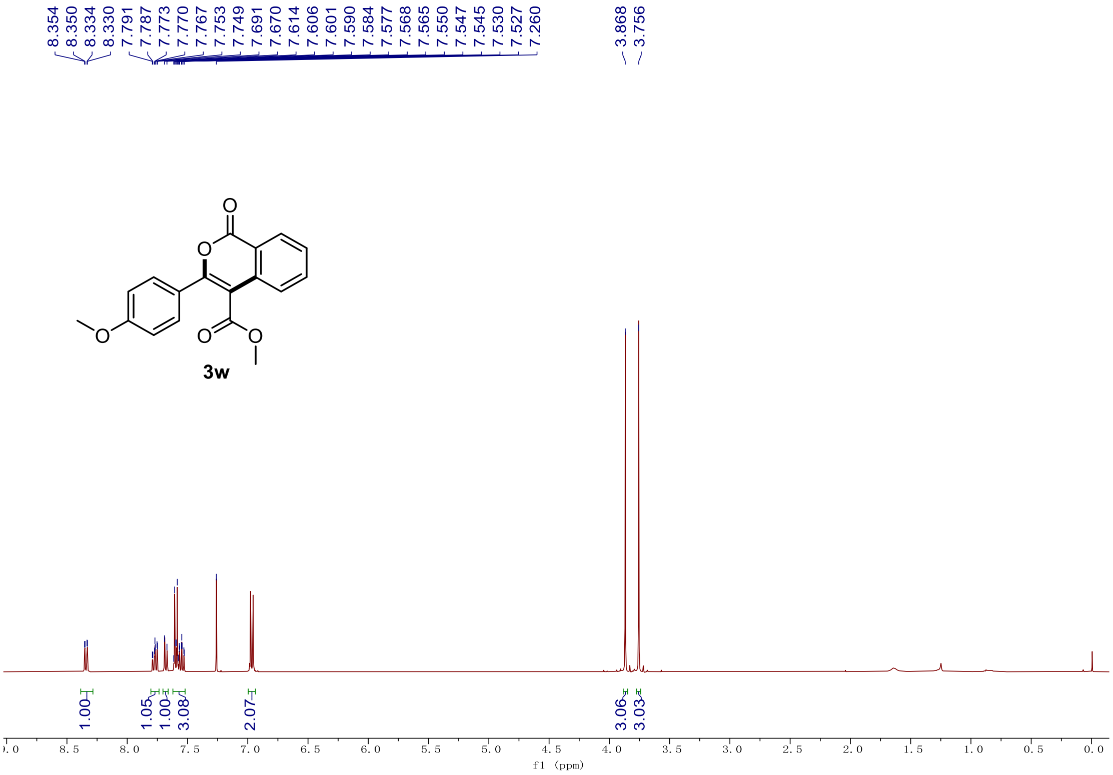

^{13}C NMR of **3w** ($CDCl_3$, 101 M)

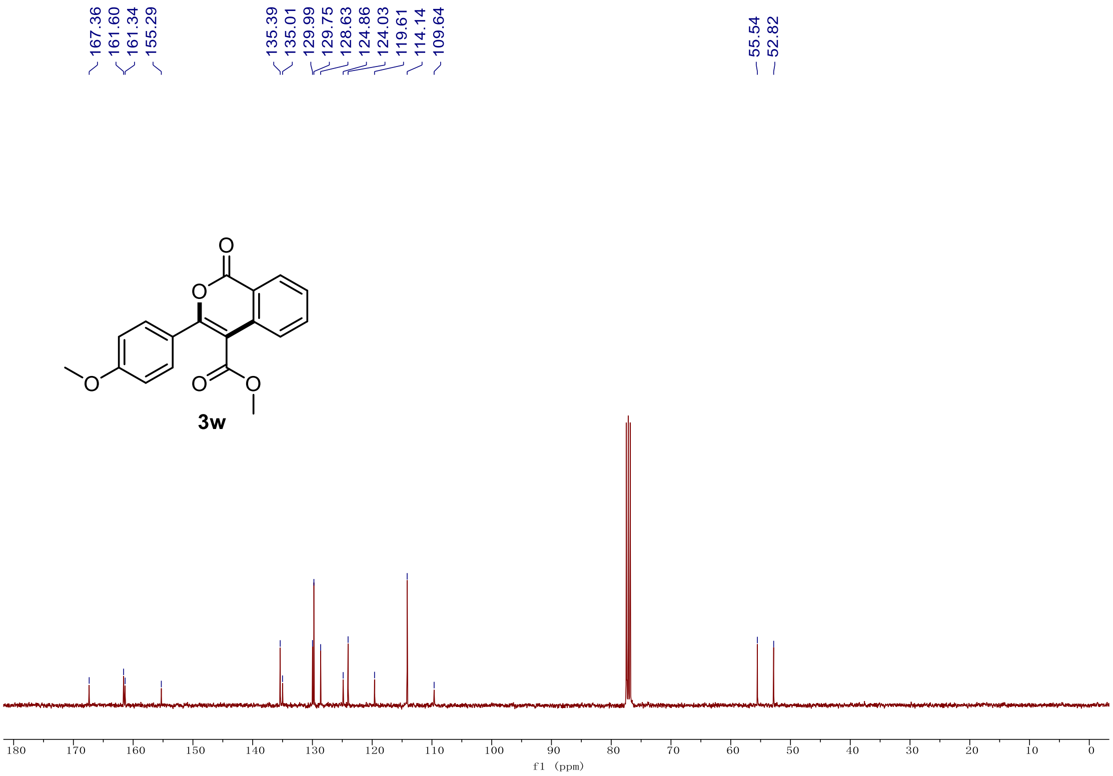

^{1}H NMR of **3x** ($CDCl_3$, 400 M)

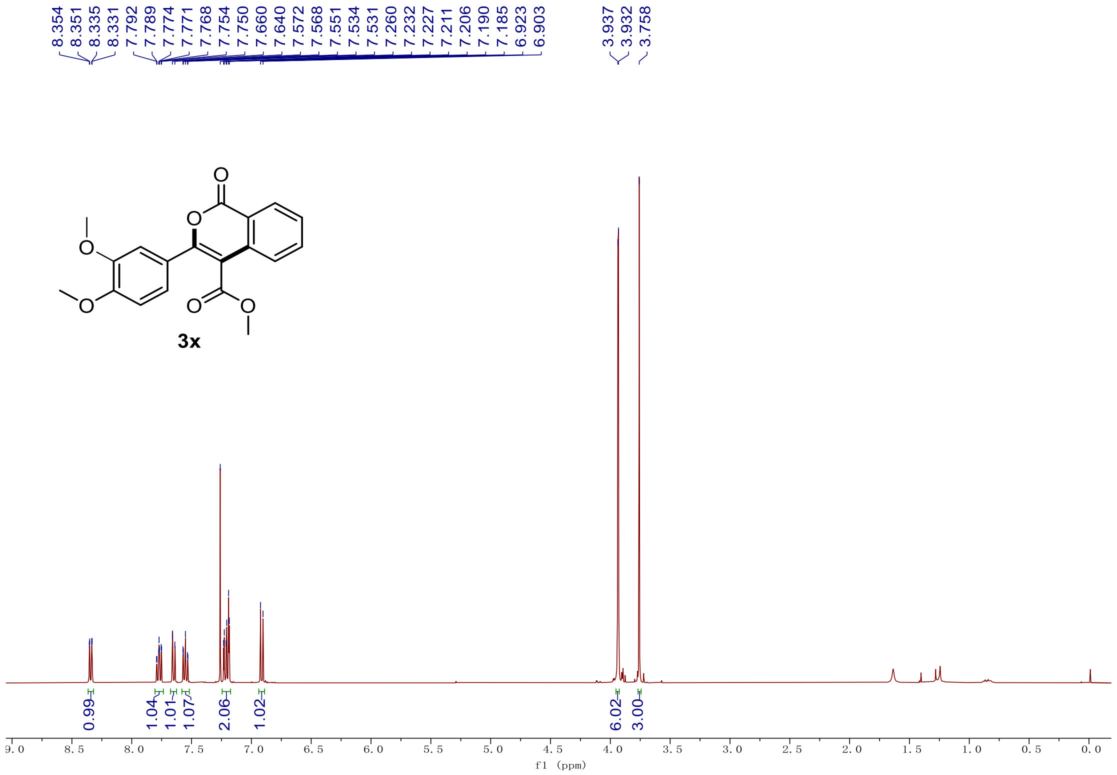

^{13}C NMR of **3x** ($CDCl_3$, 101 M)

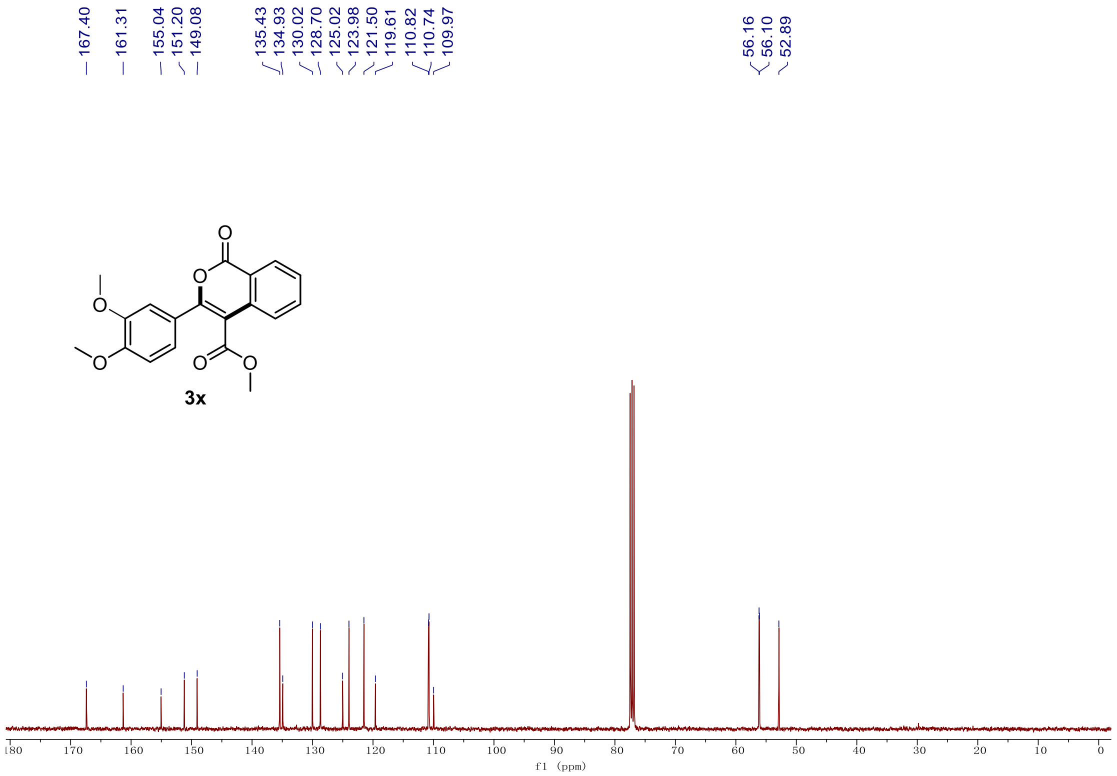

^{1}H NMR of **3aa** ($CDCl_3$, 400 M)

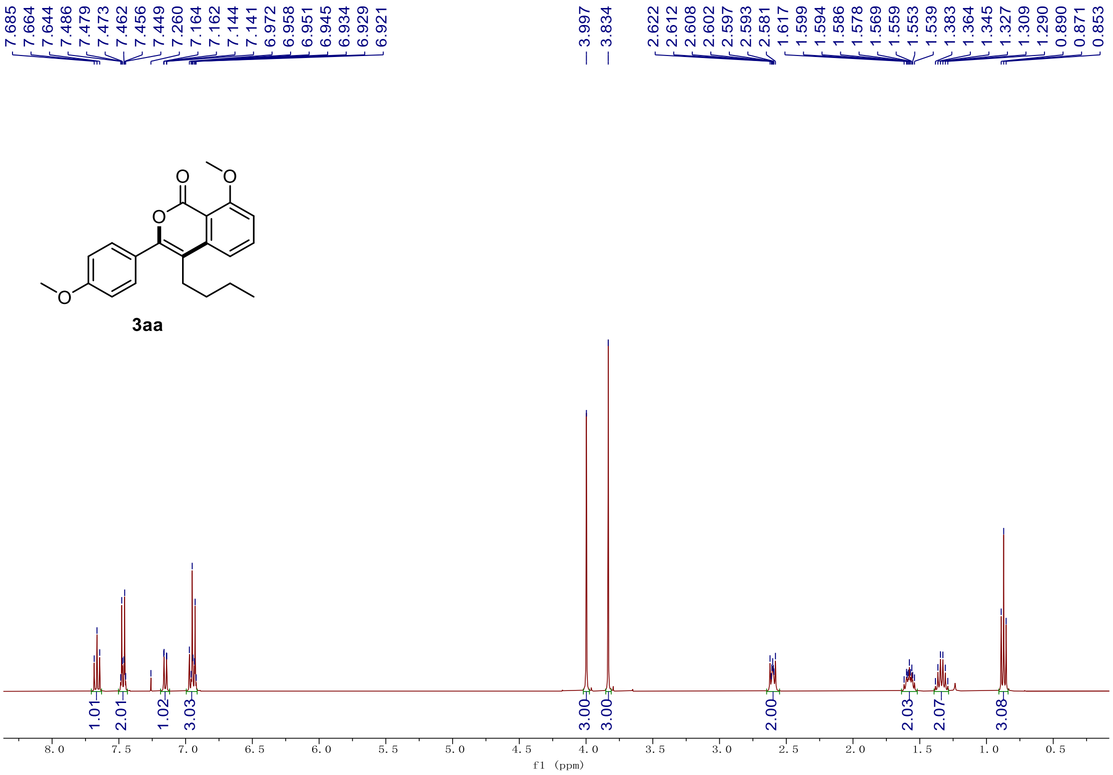

^{13}C NMR of **3aa** ($CDCl_3$, 101 M)

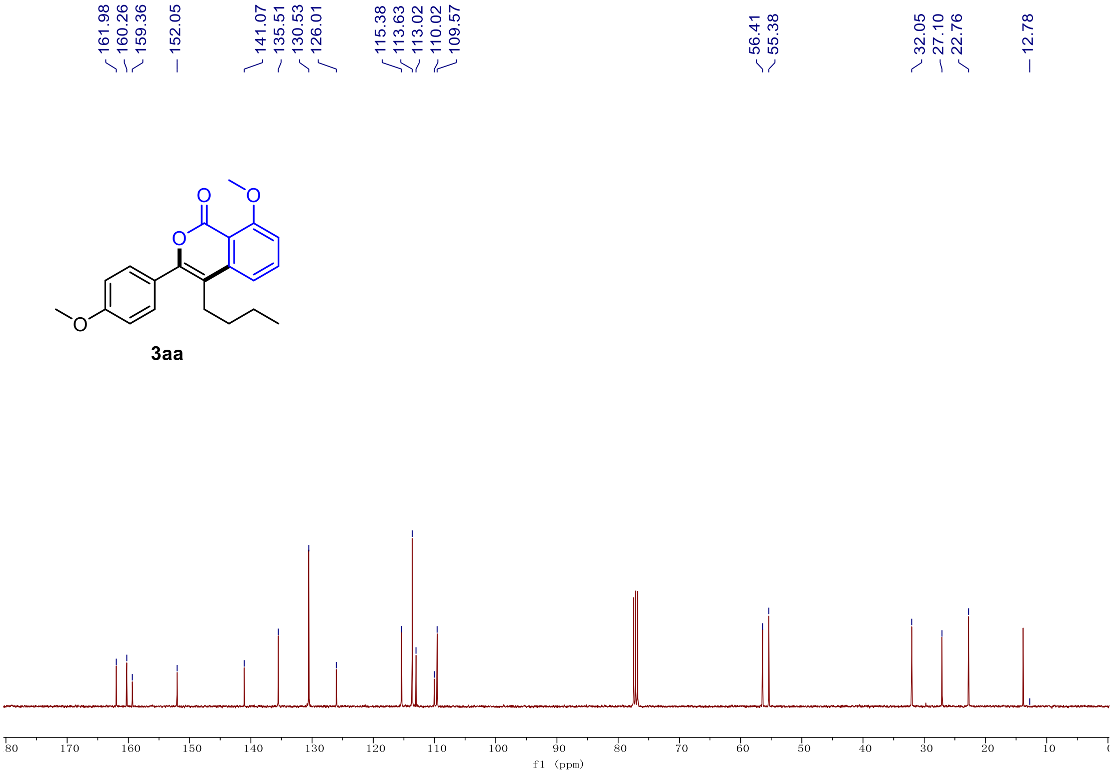

^{1}H NMR of **3ab** ($CDCl_3$, 400 M)

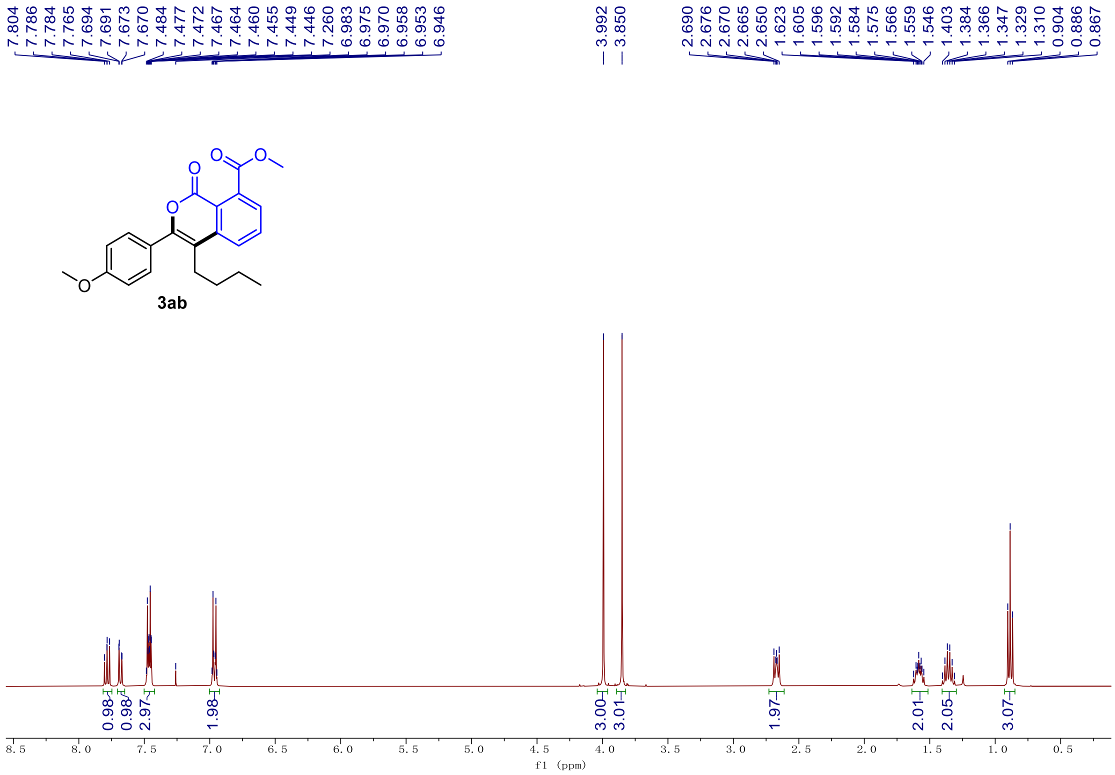

^{13}C NMR of **3ab** ($CDCl_3$, 101 M)

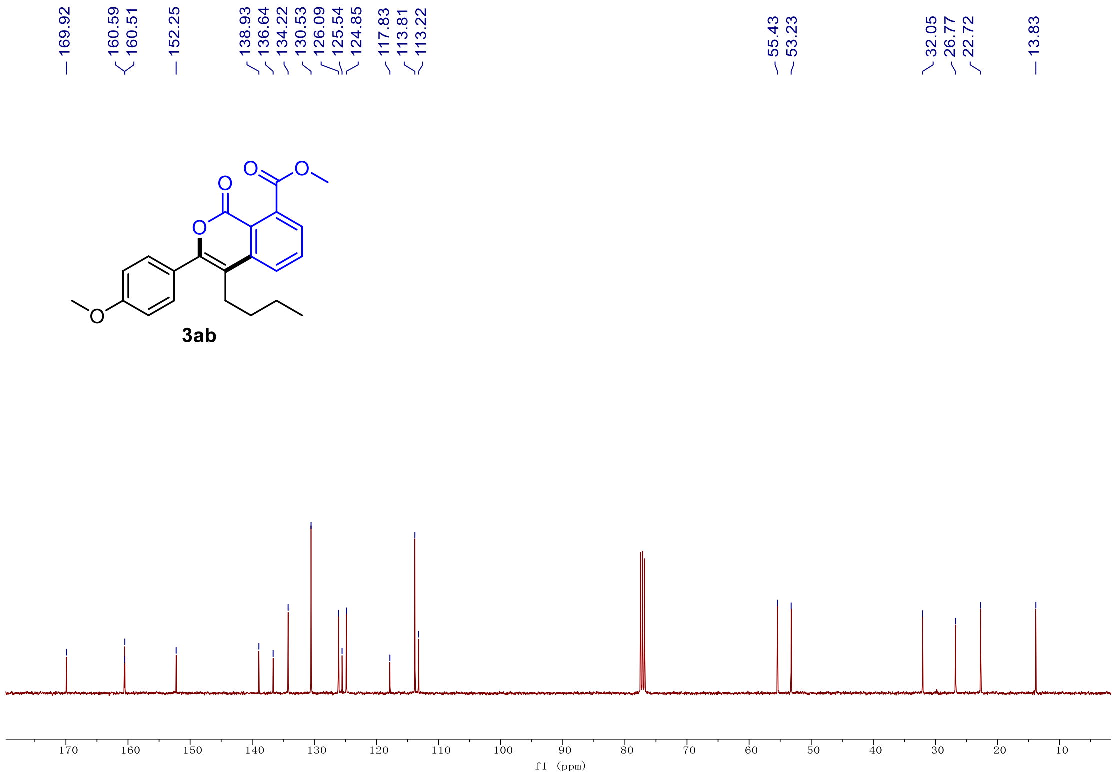

^{1}H NMR of **3ac** ($CDCl_3$, 400 M)

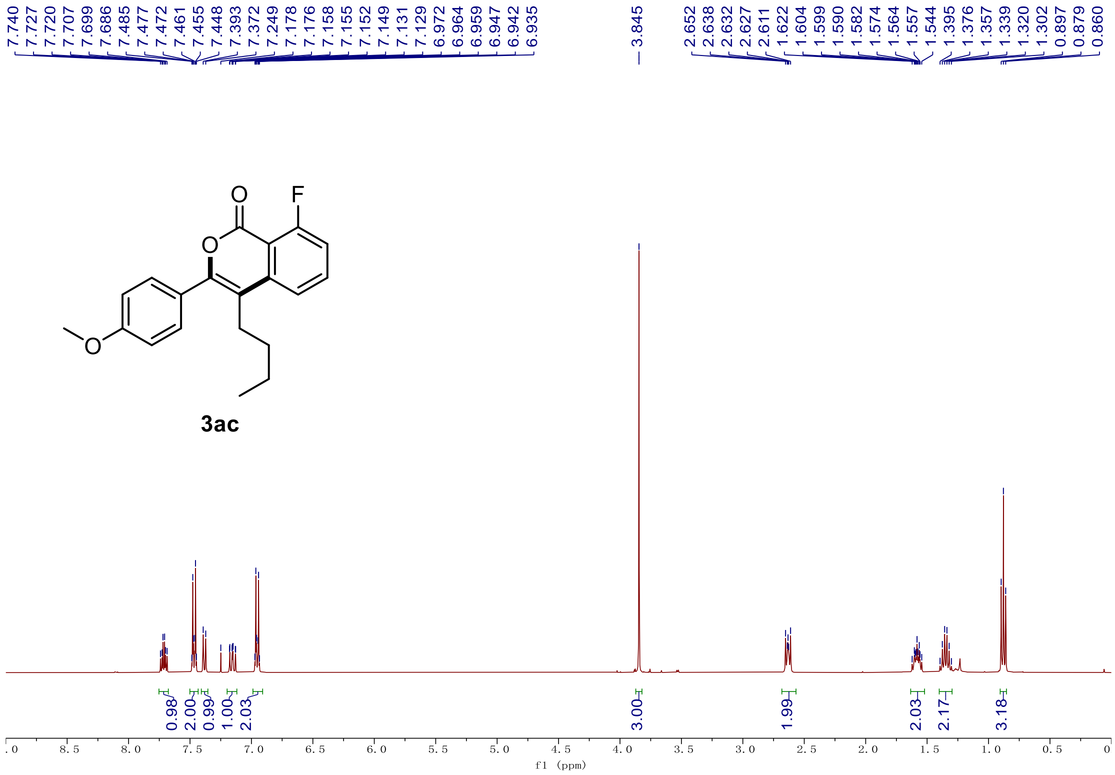

^{13}C NMR of **3ac** ($CDCl_3$, 101 M)

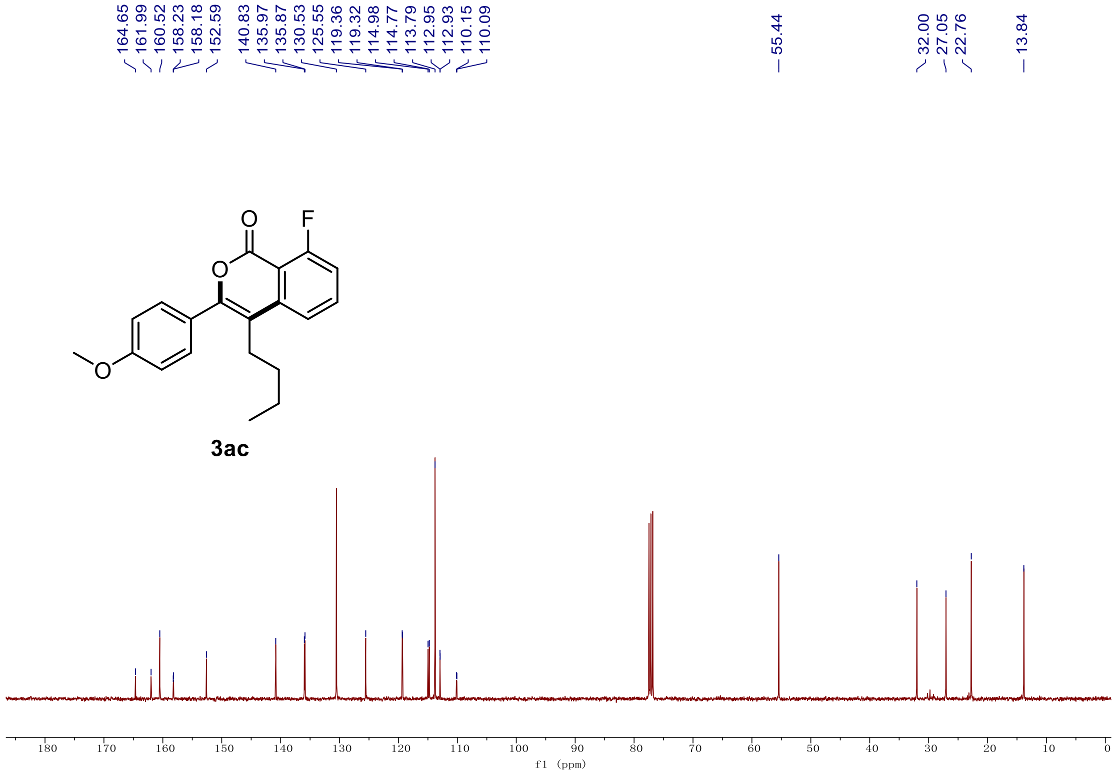

^{19}F NMR of **3ac** ($CDCl_3$, 376 M)

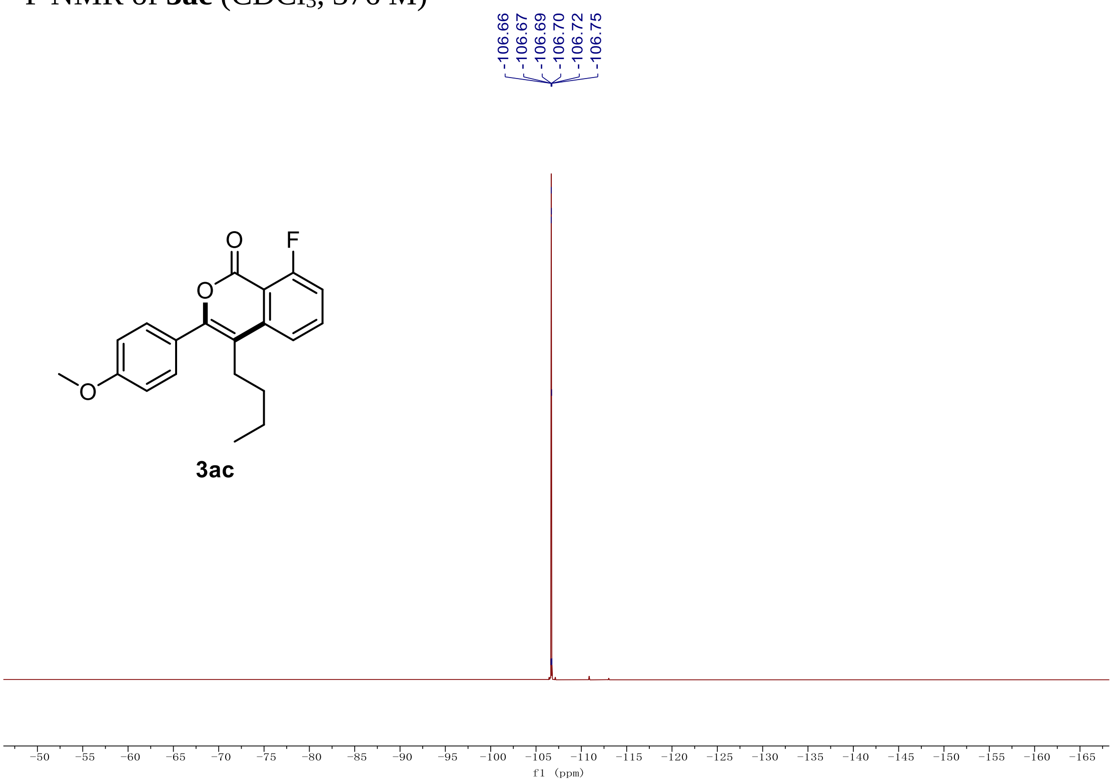

^{1}H NMR of **3ad** ($CDCl_3$, 400 M)

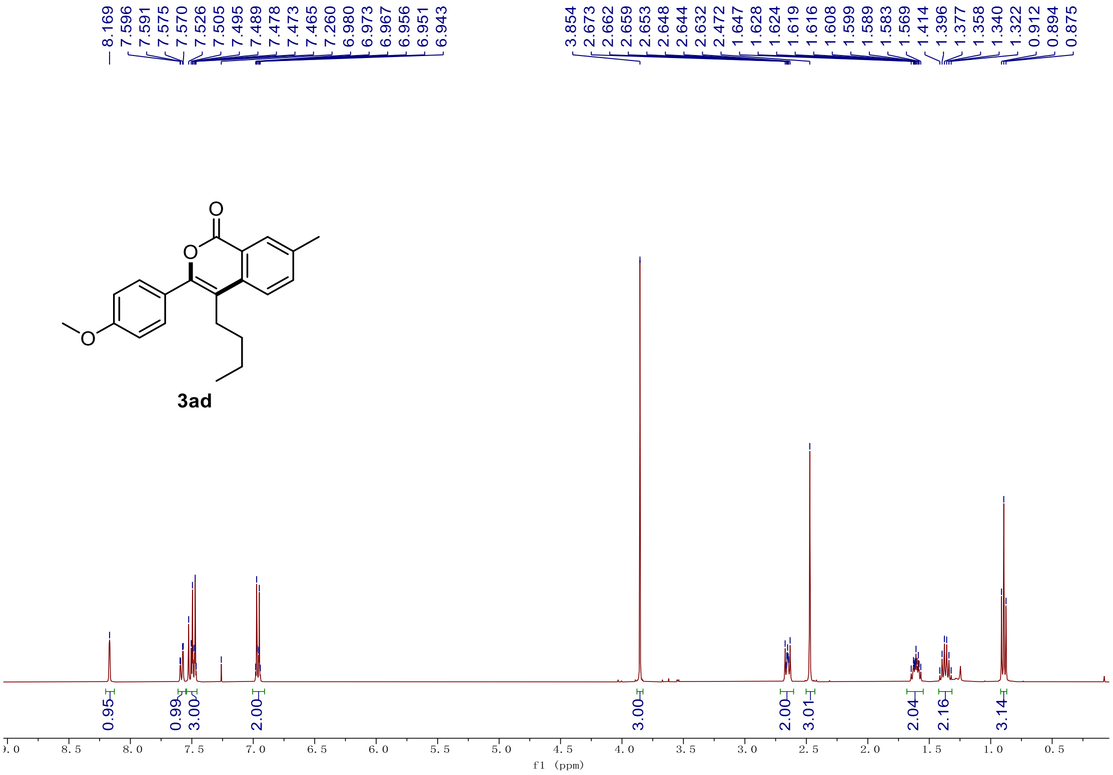

^{13}C NMR of **3ad** ($CDCl_3$, 101 M)

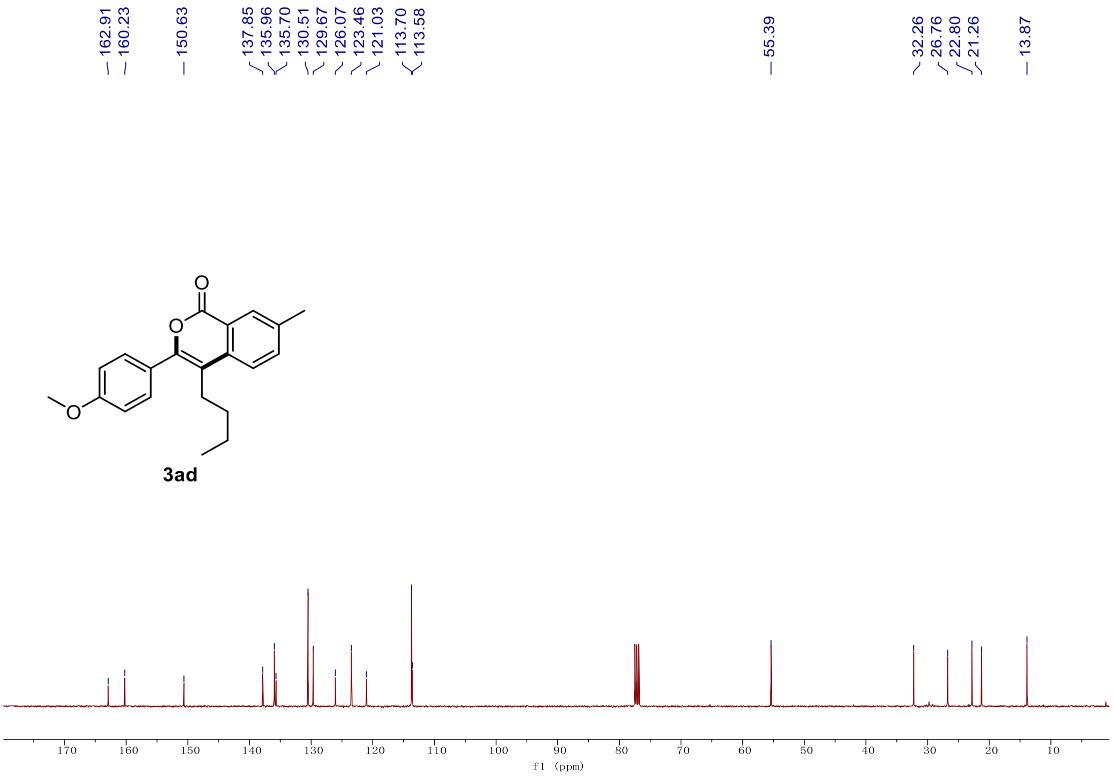

^{1}H NMR of **3ae** ($CDCl_3$, 400 M)

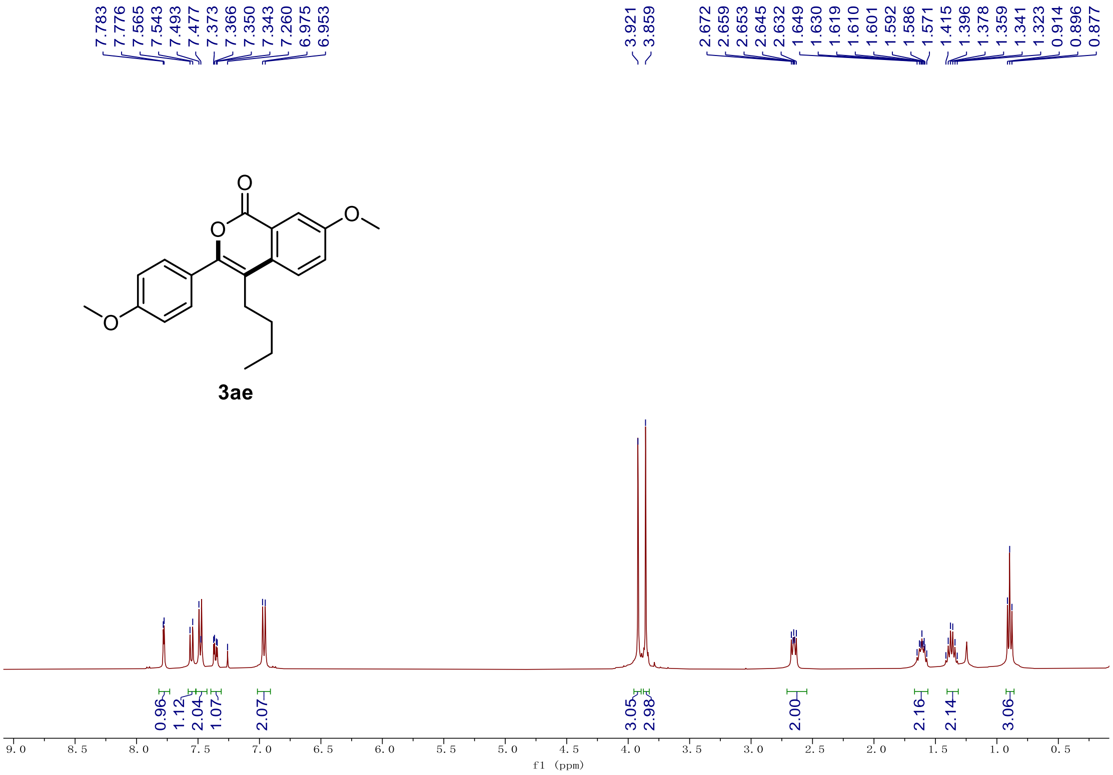

^{13}C NMR of **3ae** ($CDCl_3$, 101 M)

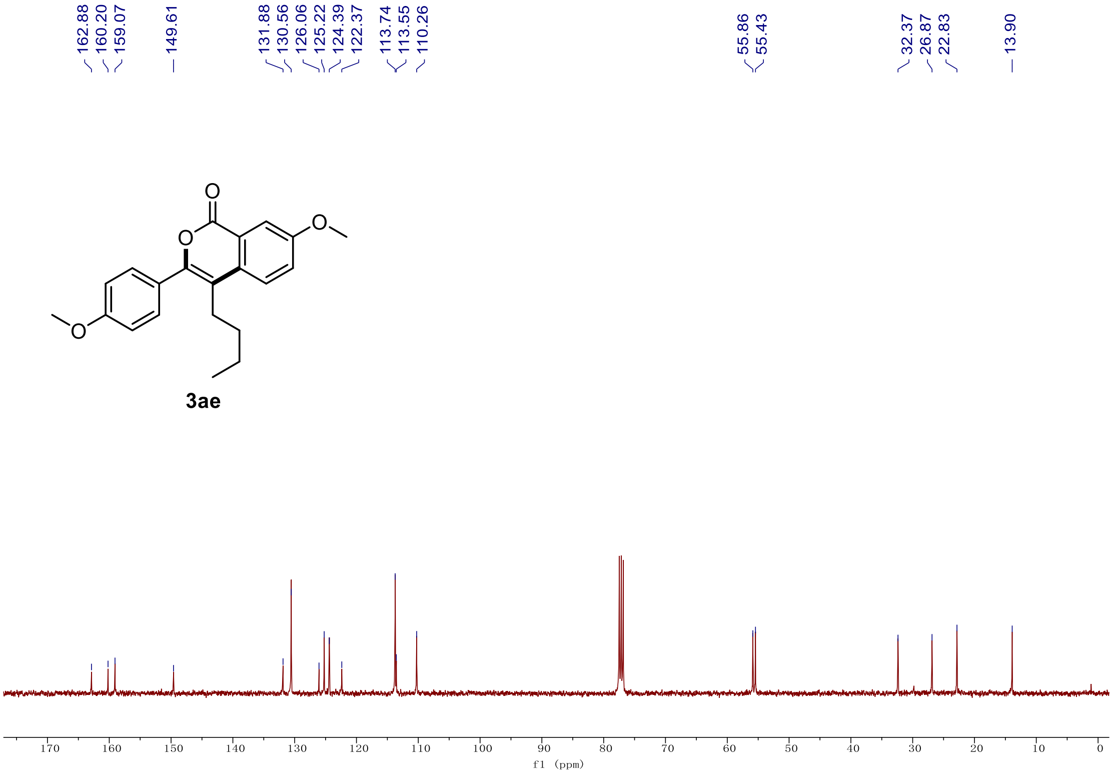

^{1}H NMR of **3af** ($CDCl_3$, 400 M)

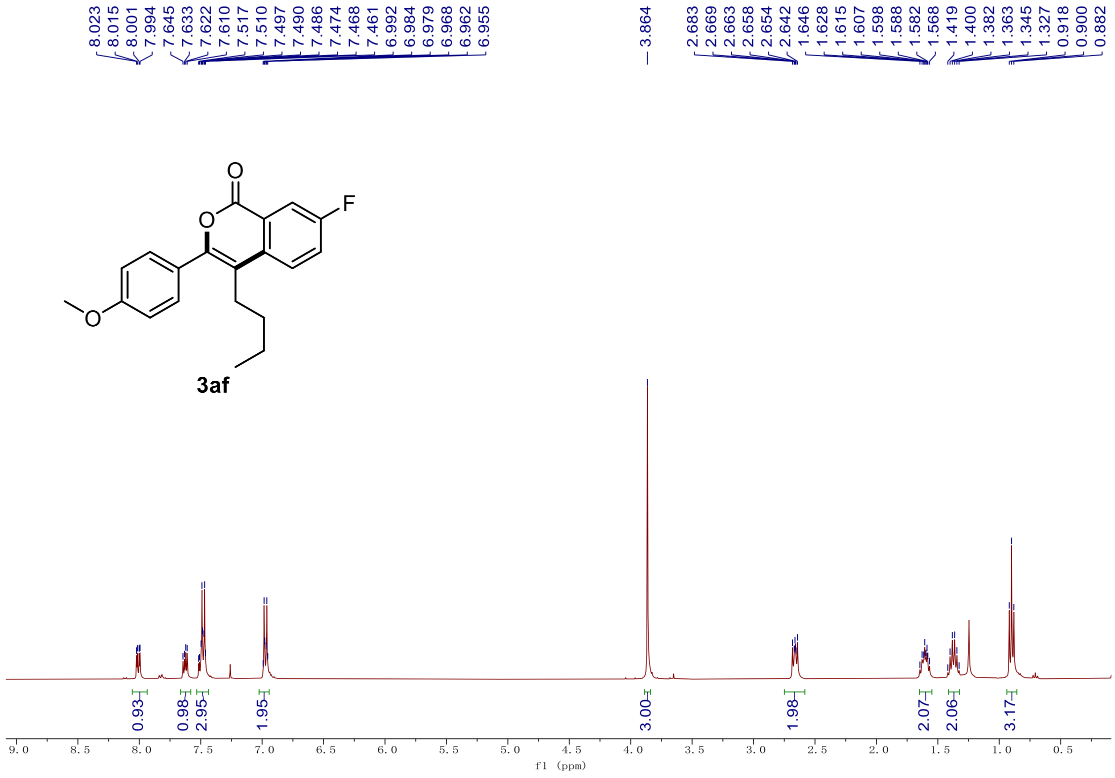

^{13}C NMR of **3af** ($CDCl_3$, 101 M)

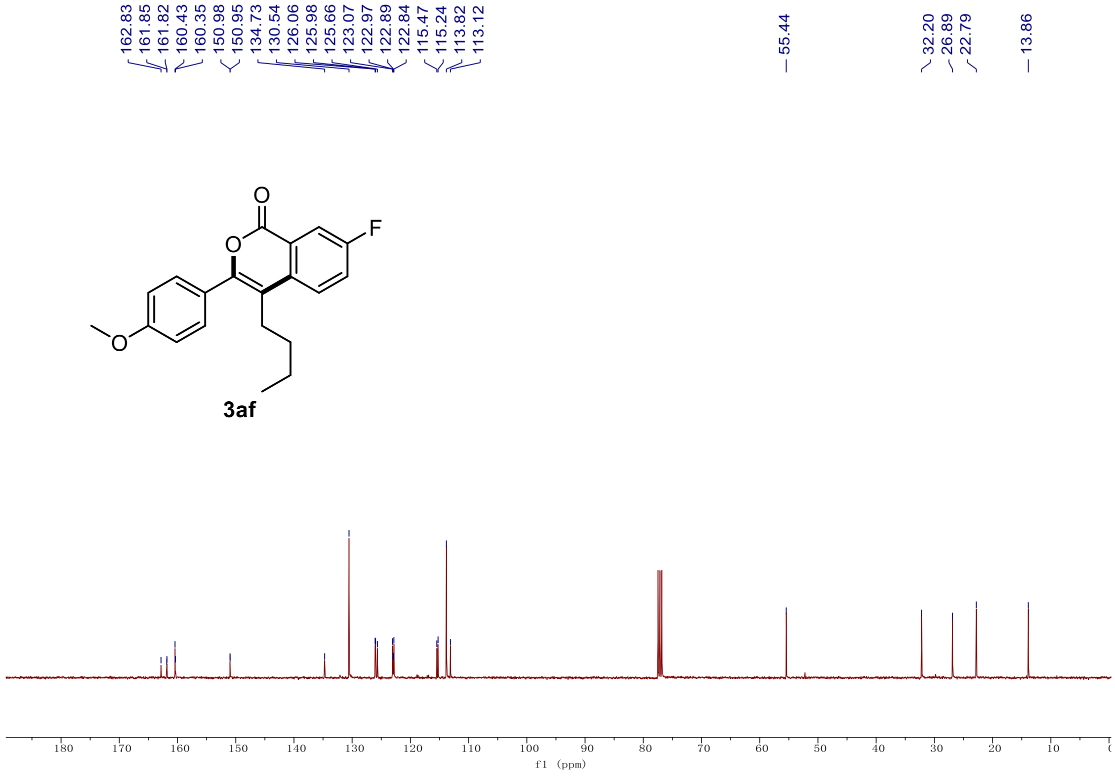

^{19}F NMR of **3af** ($CDCl_3$, 376 M)

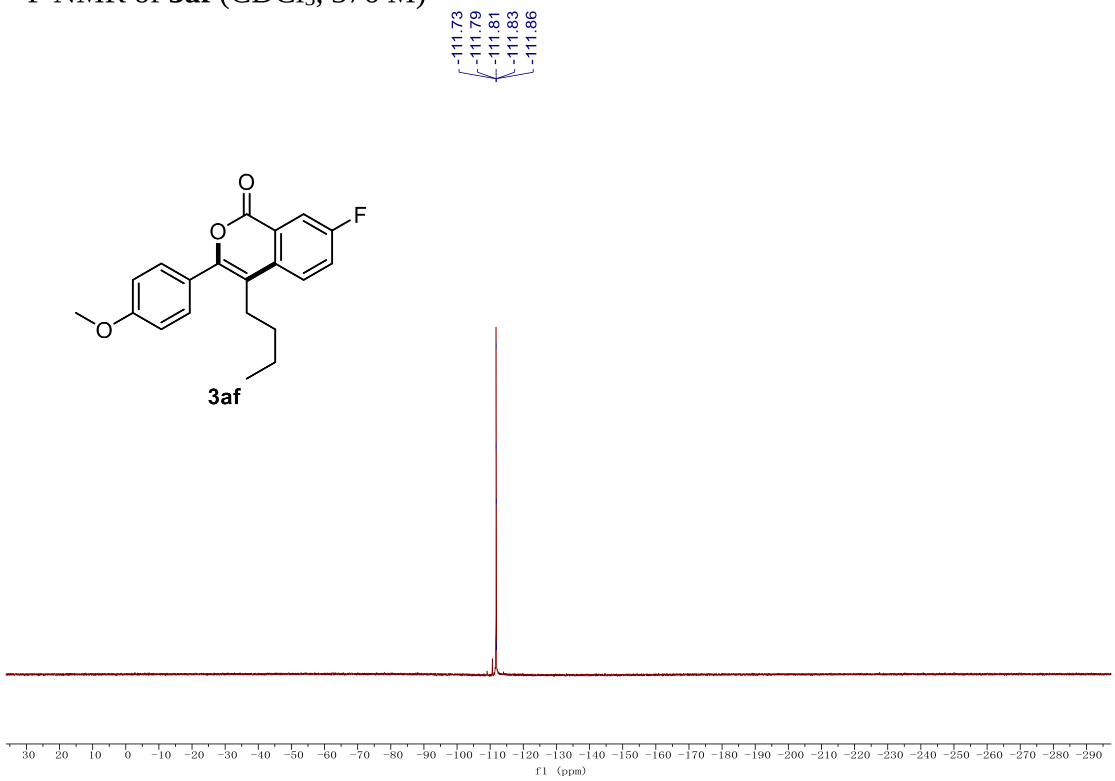

^{1}H NMR of **3ag** ($CDCl_3$, 400 M)

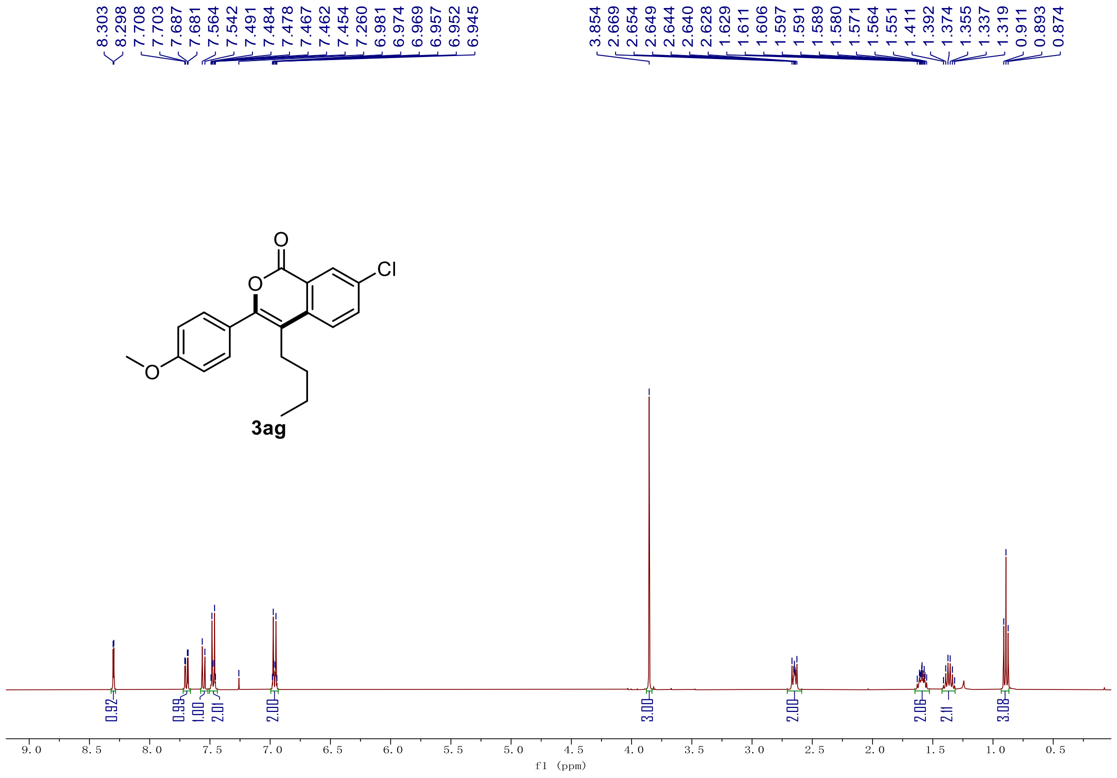

^{13}C NMR of **3ag** ($CDCl_3$, 101 M)

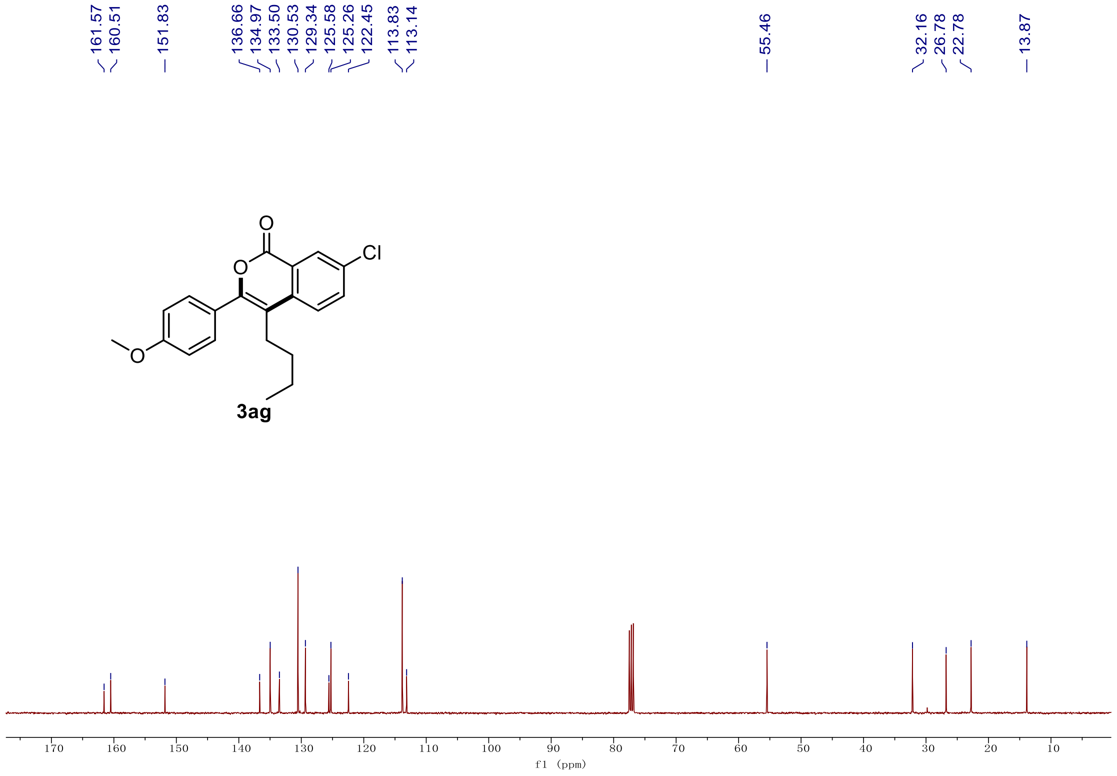

^{1}H NMR of **3ah** ($CDCl_3$, 400 M)

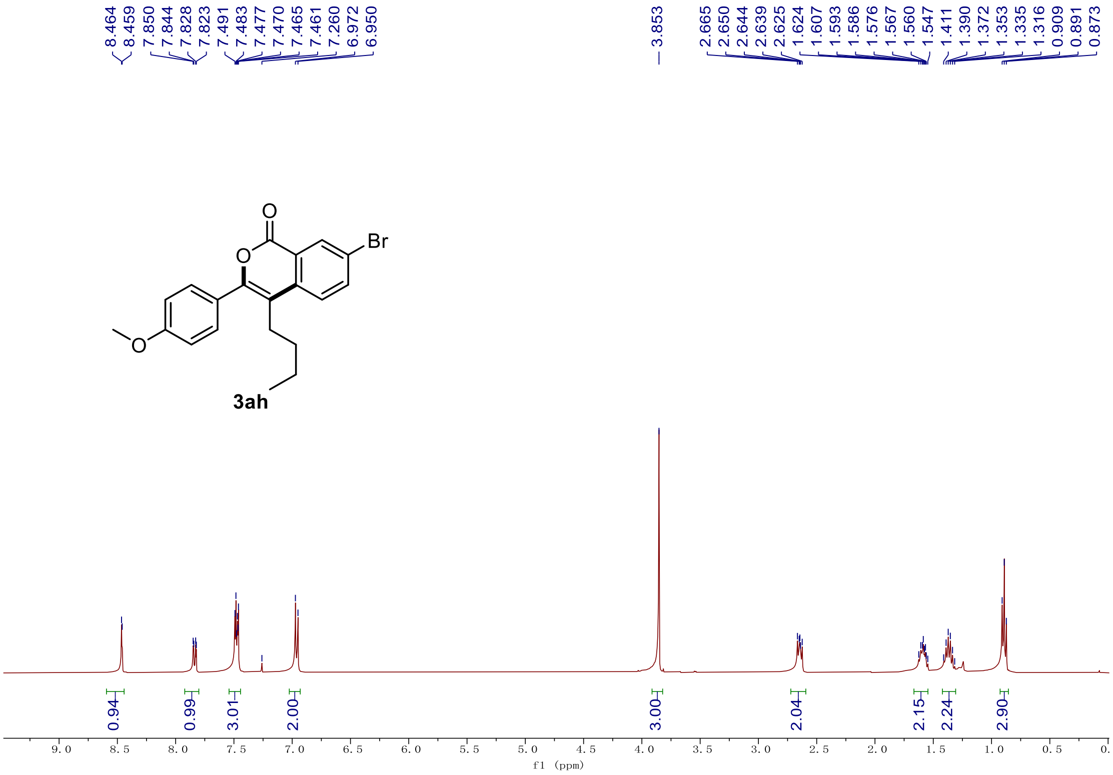

^{13}C NMR of **3ah** ($CDCl_3$, 101 M)

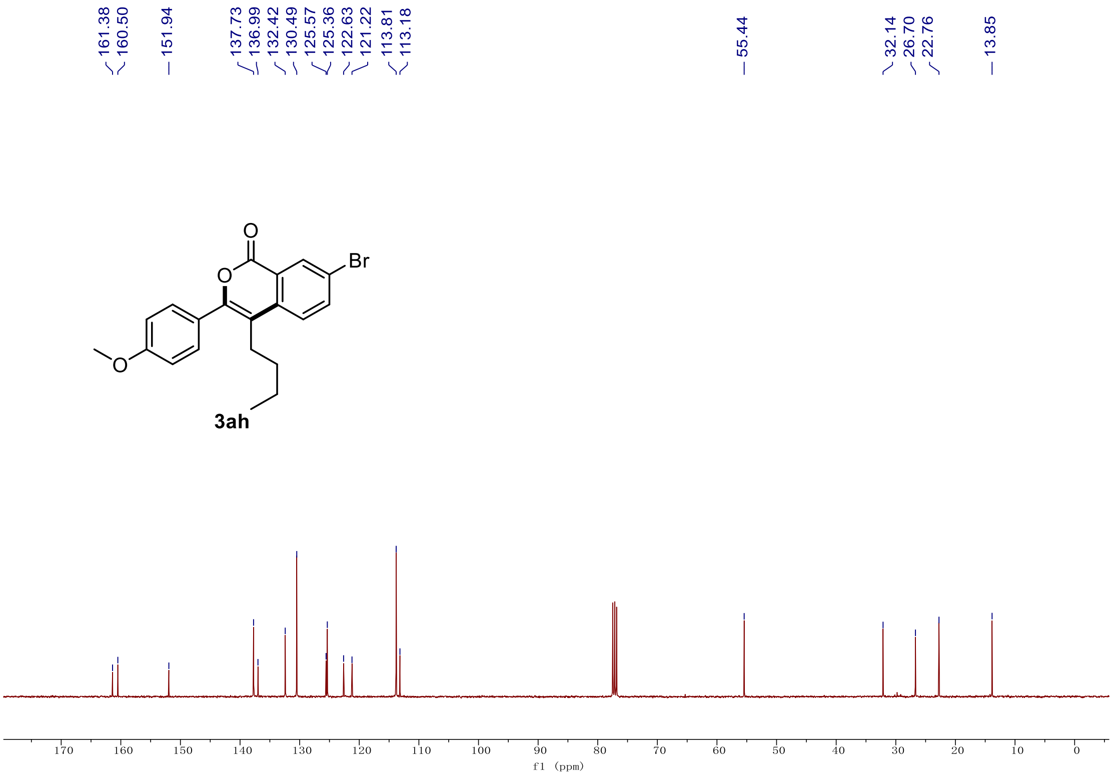

^{1}H NMR of **3ai** ($CDCl_3$, 400 M)

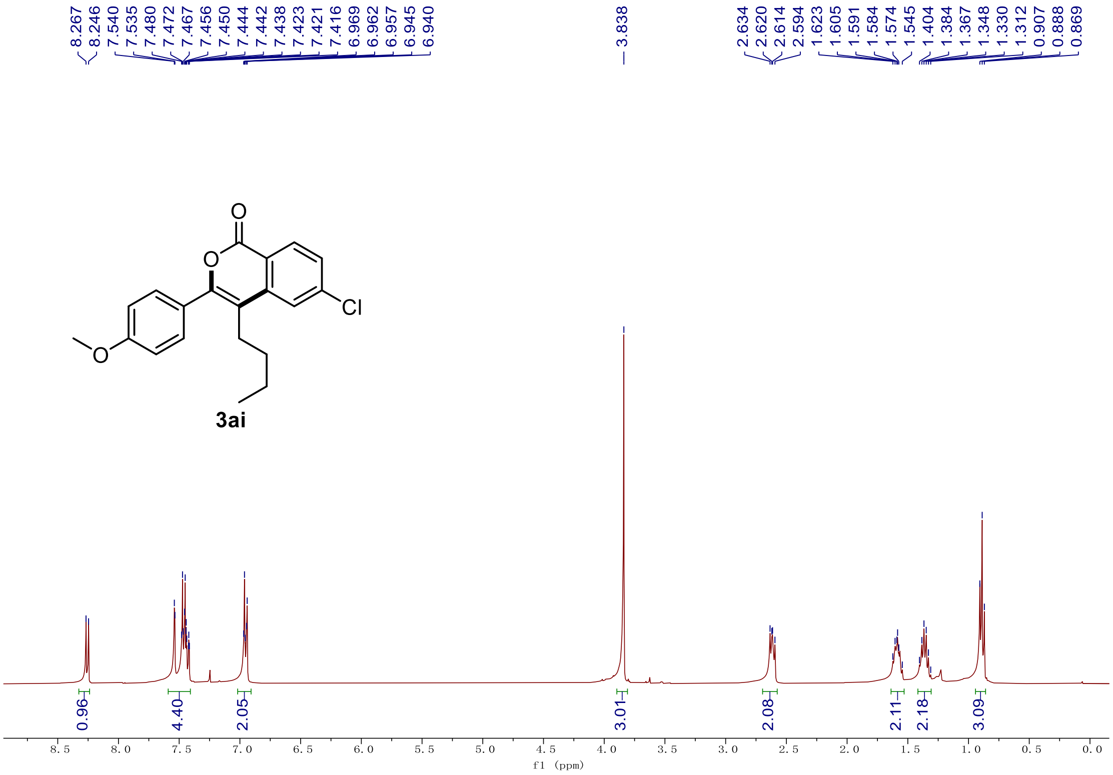

^{13}C NMR of **3ai** ($CDCl_3$, 101 M)

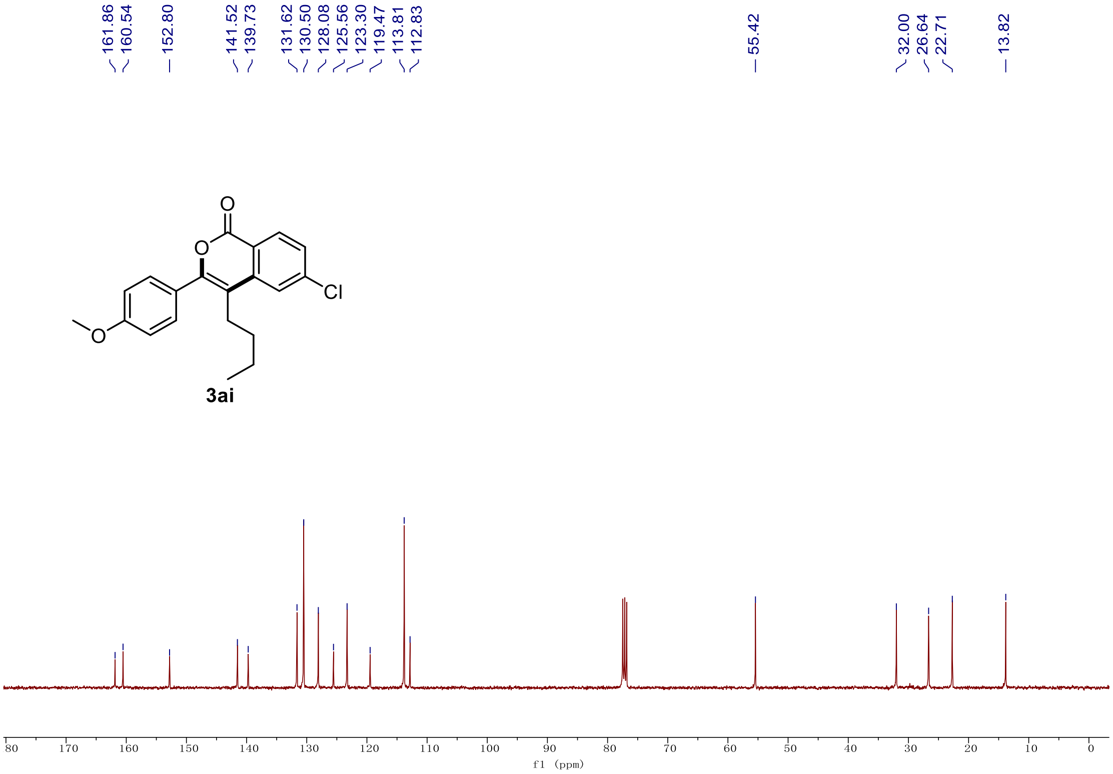

^{1}H NMR of **3aj** ($CDCl_3$, 400 M)

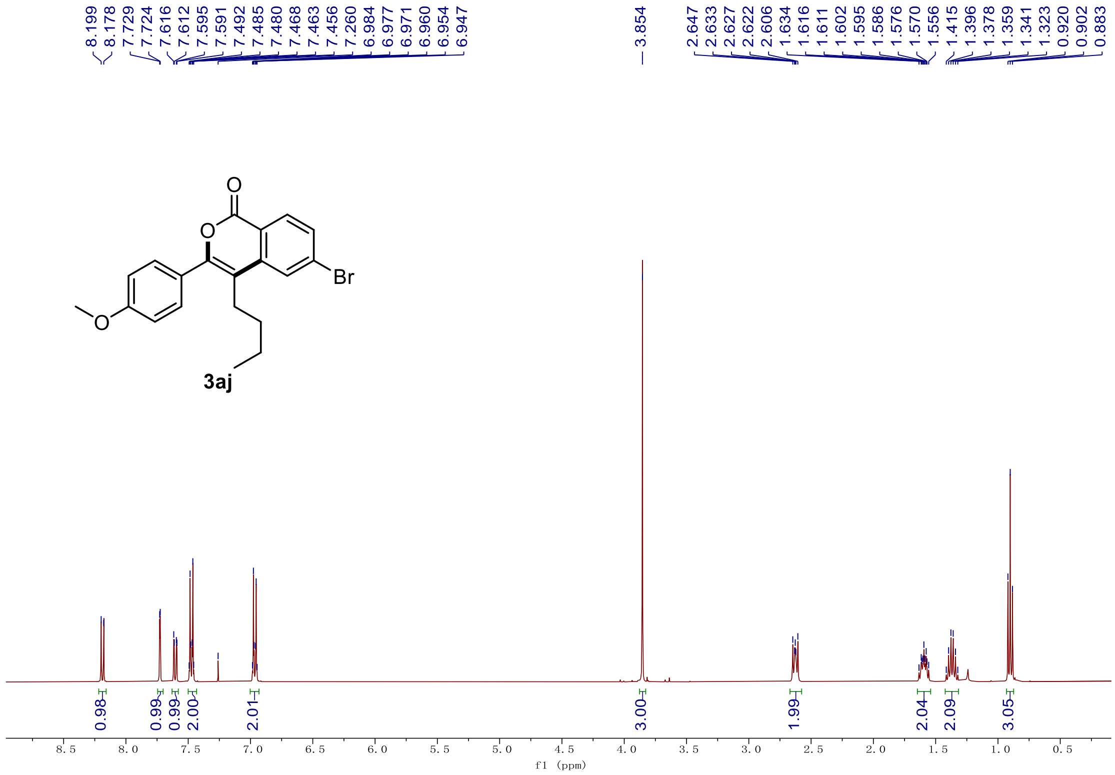

^{13}C NMR of **3aj** ($CDCl_3$, 101 M)

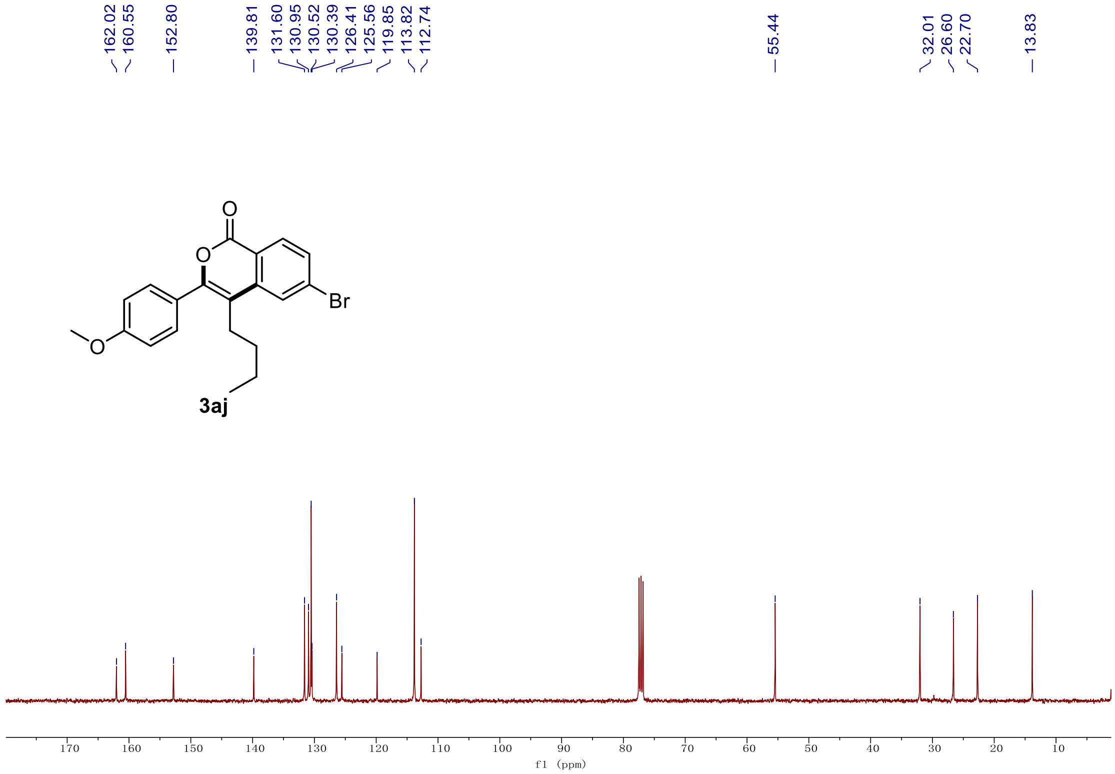

^{1}H NMR of **3am** ($CDCl_3$, 400 M)

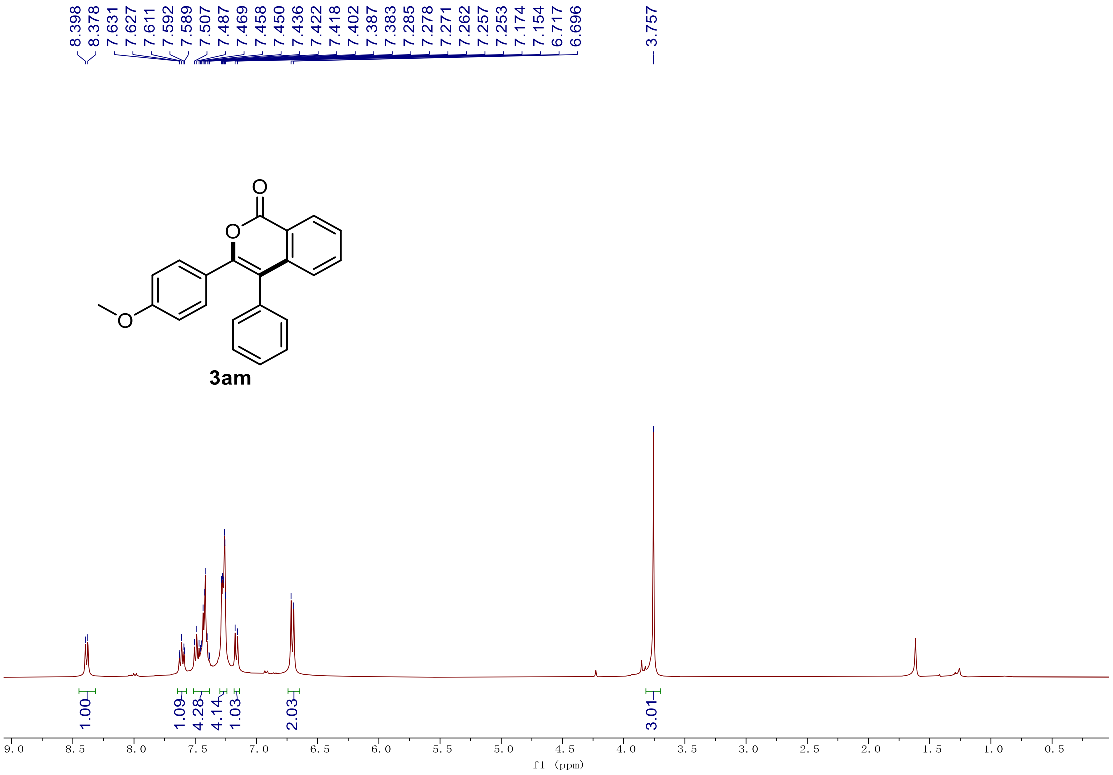

^{13}C NMR of **3am** ($CDCl_3$, 101 M)

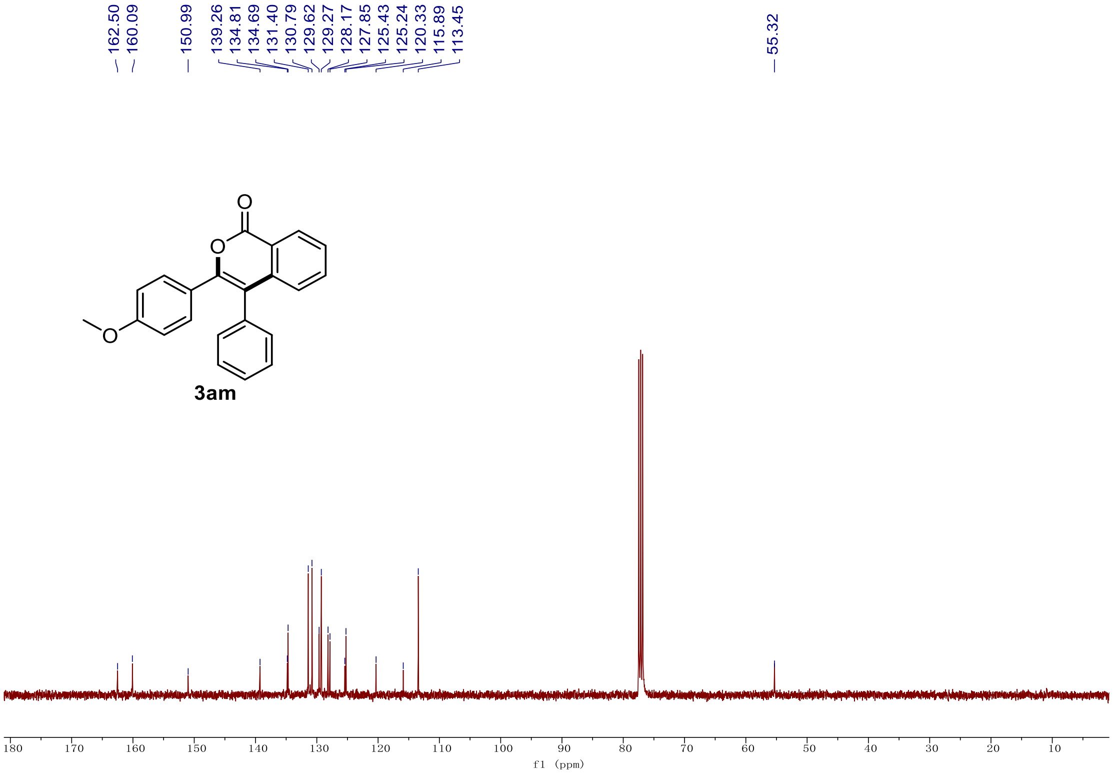

^{1}H NMR of **3ao** ($CDCl_3$, 400 M)

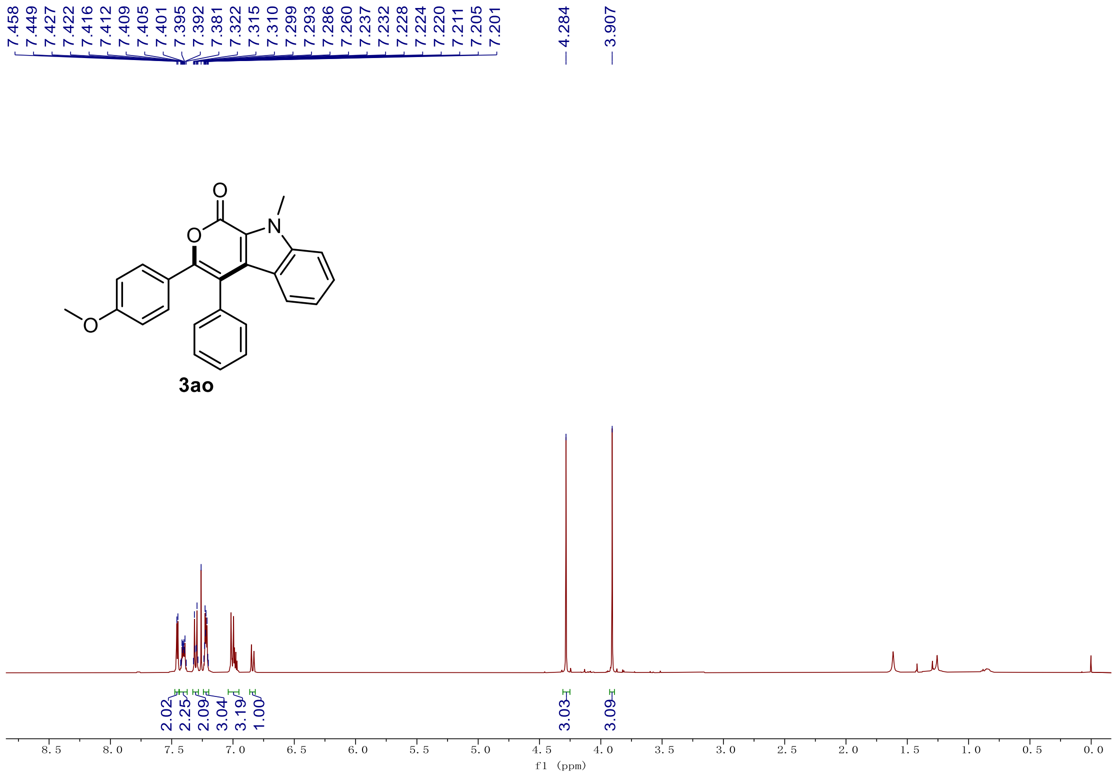

^{13}C NMR of **3ao** ($CDCl_3$, 101 M)

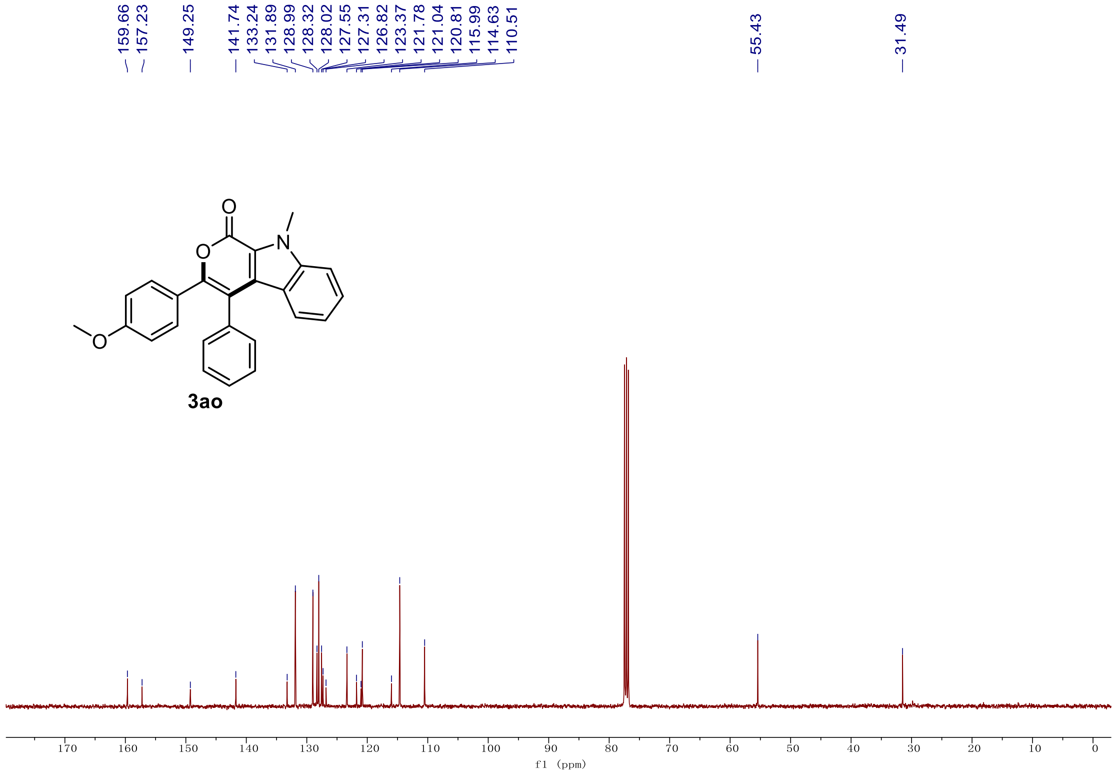

^{1}H NMR of **3aq** ($CDCl_3$, 400 M)

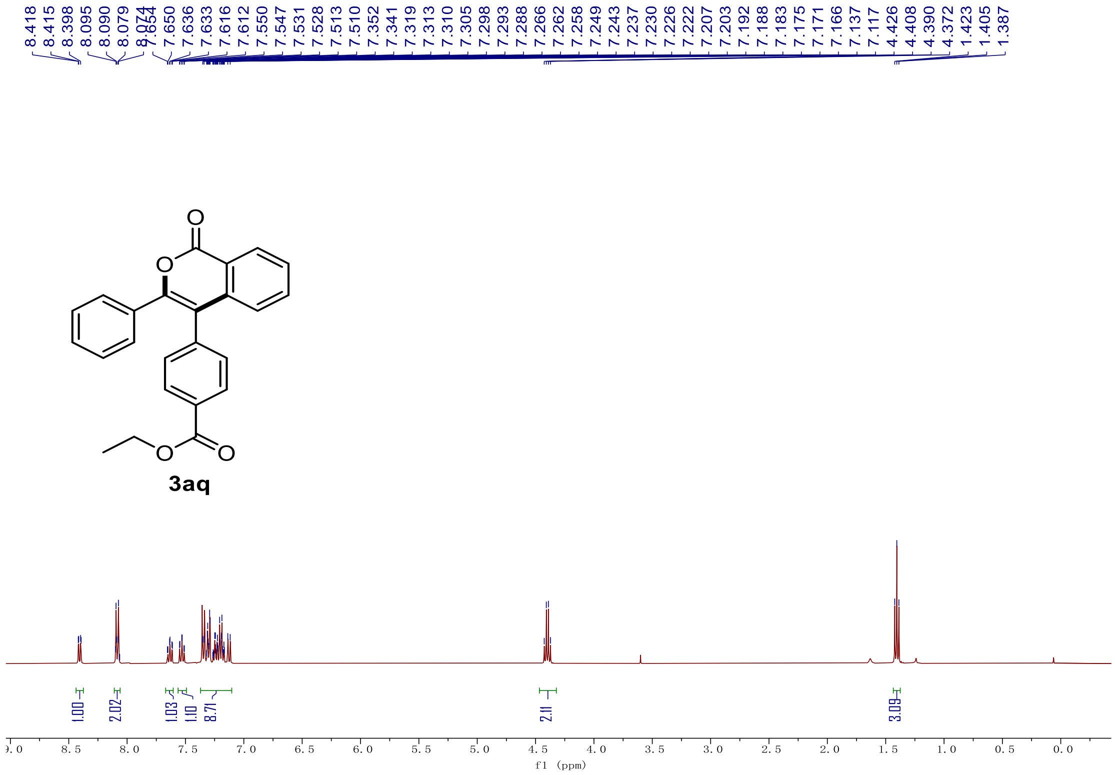

^{13}C NMR of **3aq** ($CDCl_3$, 101 M)

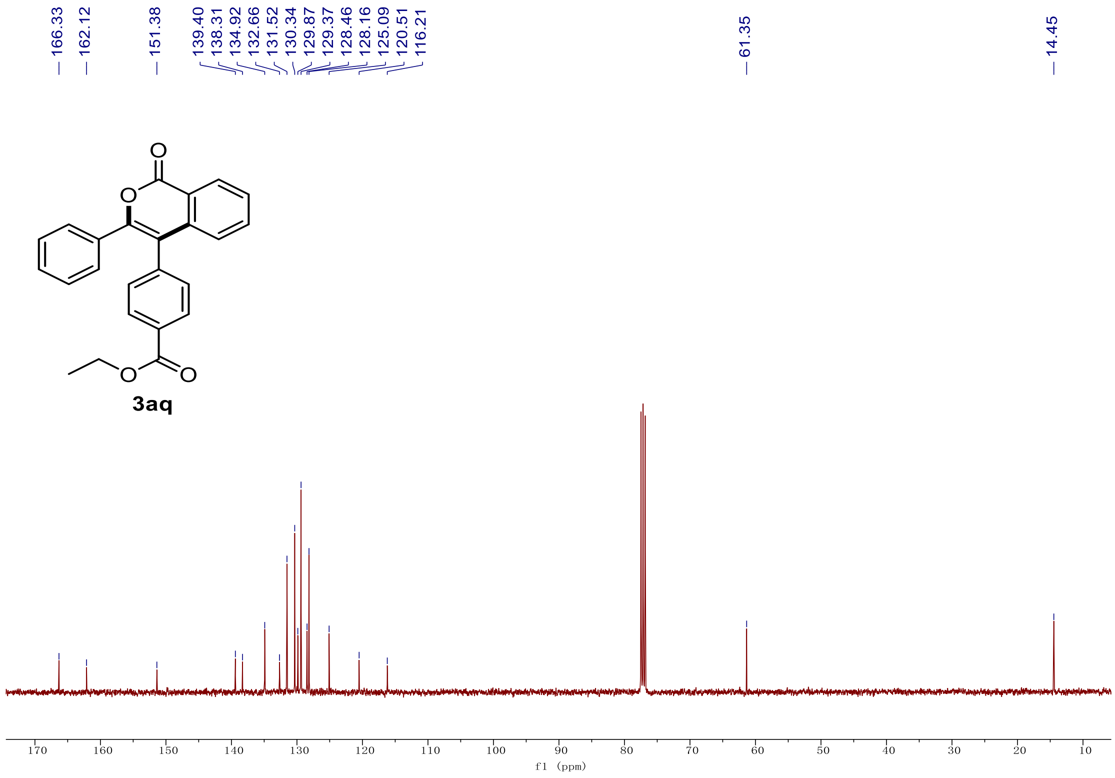

^{1}H NMR of **7** ($CDCl_3$, 400 M)

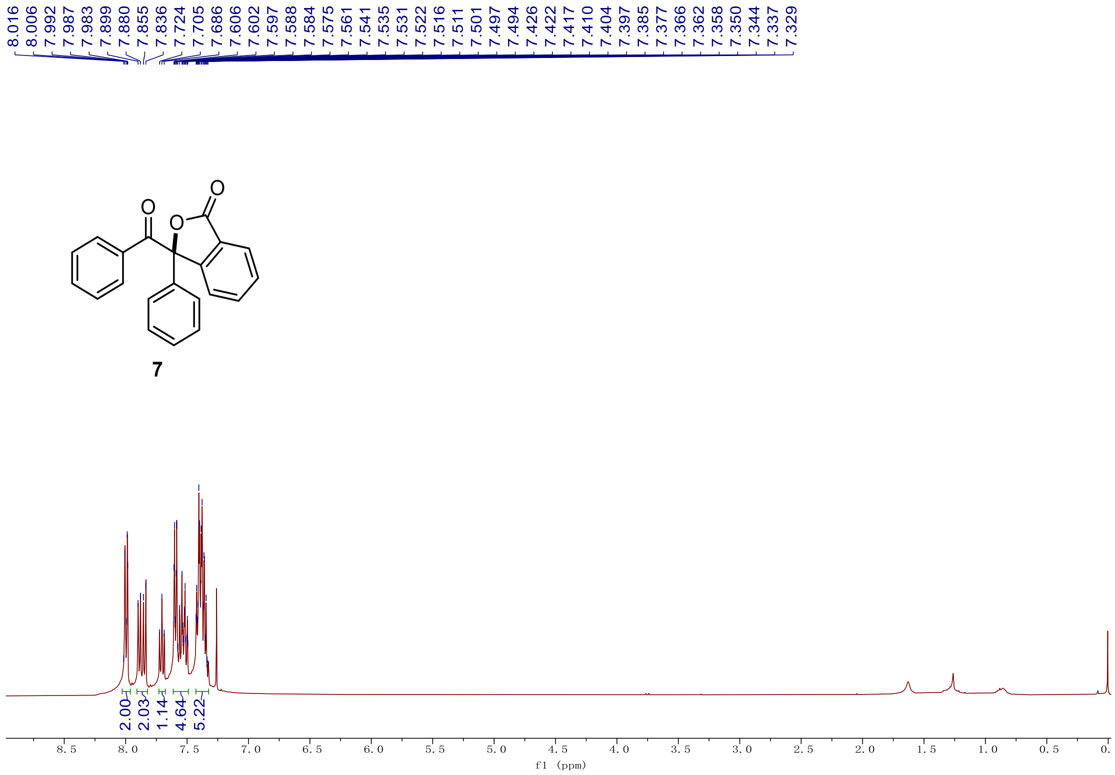

^{13}C NMR of **6** ($CDCl_3$, 101 M)

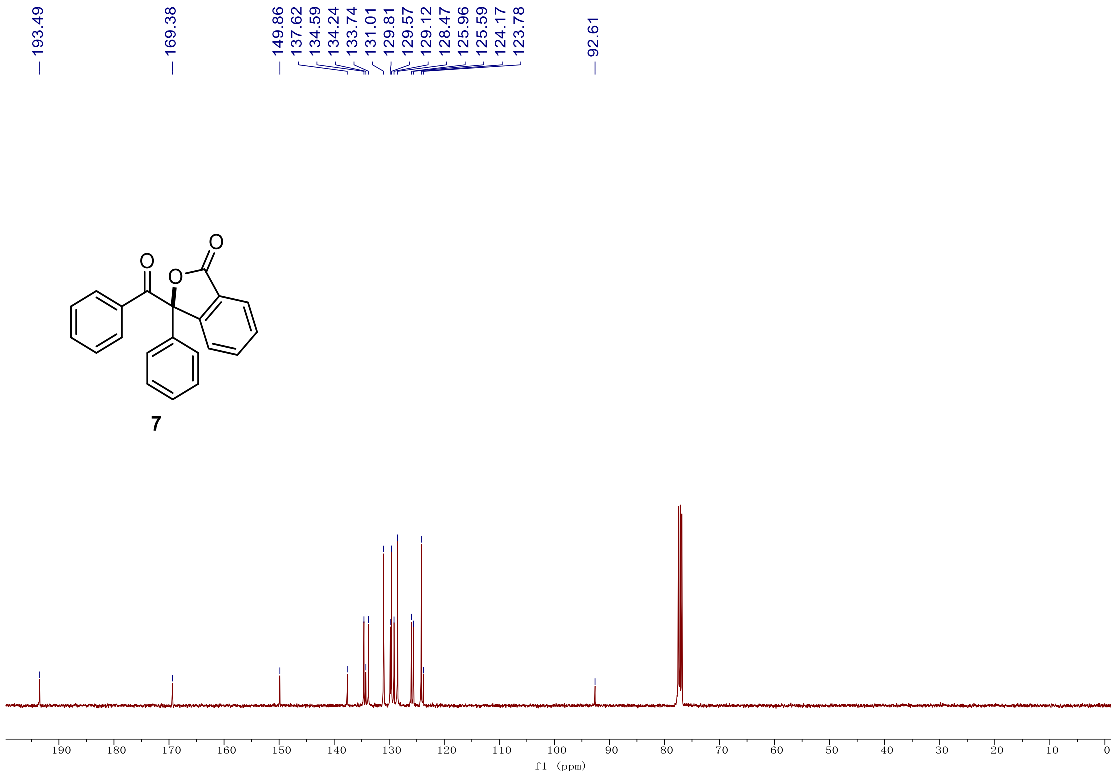

^{1}H NMR of **3ar** ($CDCl_3$, 400 M)

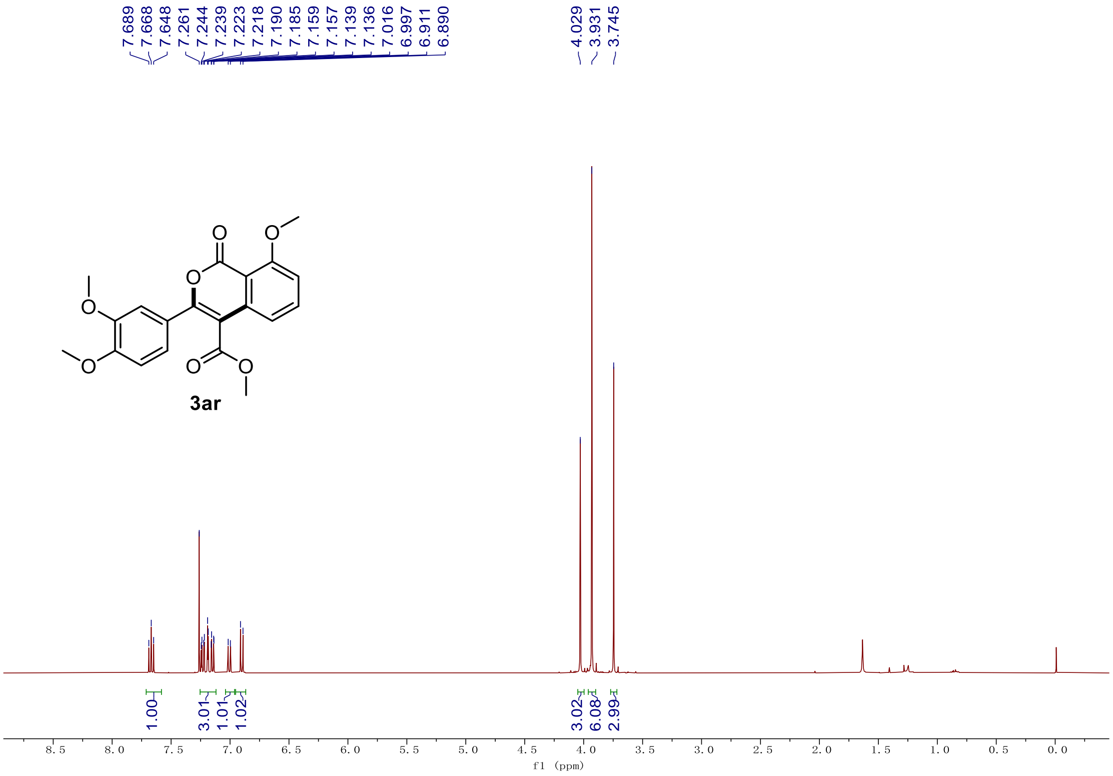

^{13}C NMR of **3ar** ($CDCl_3$, 101 M)

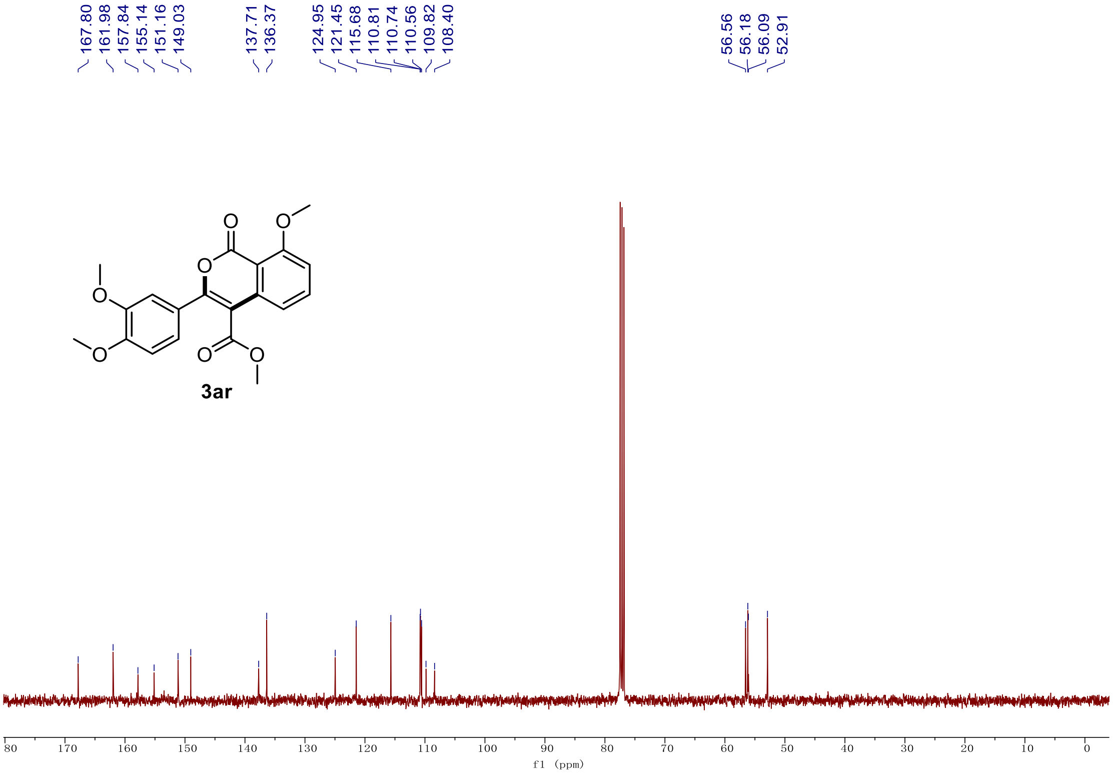

^{1}H NMR of Thunberginol A (DMSO, 400 M)

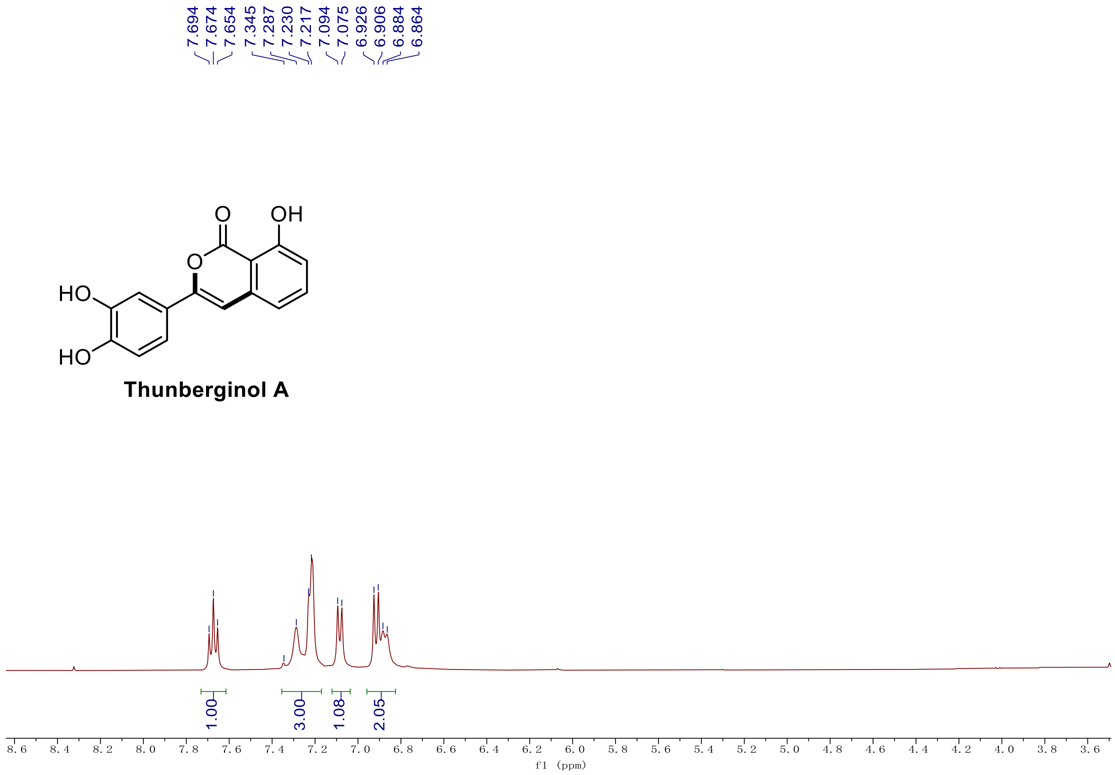

^{13}C NMR of Thunberginol A (DMSO, 101 M)

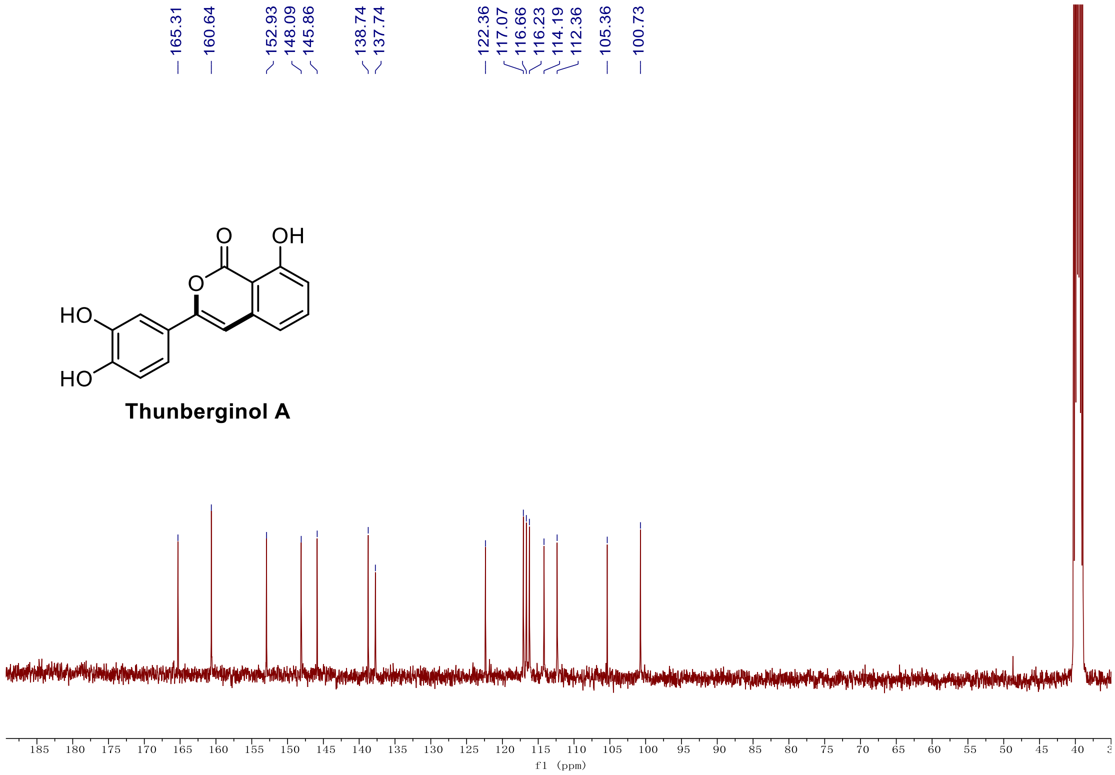

Supplement: Supplementary file 3 — Supplementary Data 1 [file 42004_2022_768_MOESM3_ESM.pdf]
